# Genome-Wide Identification and Expression Analysis of Homeodomain Leucine Zipper Subfamily IV (HDZ IV) Gene Family from *Musa accuminata*

**DOI:** 10.3389/fpls.2016.00020

**Published:** 2016-02-01

**Authors:** Ashutosh Pandey, Prashant Misra, Anshu Alok, Navneet Kaur, Shivani Sharma, Deepika Lakhwani, Mehar H. Asif, Siddharth Tiwari, Prabodh K. Trivedi

**Affiliations:** ^1^Department of Biotechnology, National Agri-Food Biotechnology InstituteMohali, India; ^2^CSIR-National Botanical Research InstituteLucknow, India

**Keywords:** banana, homeodomain zipper transcription factors, gene family, differential expression, abiotic stress

## Abstract

The homeodomain zipper family (HD-ZIP) of transcription factors is present only in plants and plays important role in the regulation of plant-specific processes. The subfamily IV of HDZ transcription factors (HD-ZIP IV) has primarily been implicated in the regulation of epidermal structure development. Though this gene family is present in all lineages of land plants, members of this gene family have not been identified in banana, which is one of the major staple fruit crops. In the present work, we identified 21 HDZIV encoding genes in banana by the computational analysis of banana genome resource. Our analysis suggested that these genes putatively encode proteins having all the characteristic domains of HDZIV transcription factors. The phylogenetic analysis of the banana HDZIV family genes further confirmed that after separation from a common ancestor, the banana, and poales lineages might have followed distinct evolutionary paths. Further, we conclude that segmental duplication played a major role in the evolution of banana HDZIV encoding genes. All the identified banana *HDZIV* genes expresses in different banana tissue, however at varying levels. The transcript levels of some of the banana *HDZIV* genes were also detected in banana fruit pulp, suggesting their putative role in fruit attributes. A large number of genes of this family showed modulated expression under drought and salinity stress. Taken together, the present work lays a foundation for elucidation of functional aspects of the banana HDZIV encoding genes and for their possible use in the banana improvement programs.

## Introduction

Gene regulation ensures precise control over various biological processes including growth, development, and metabolism (Nath et al., 2005). Transcription factors are regulatory proteins, which play key role in transcriptional regulation of gene expression. They bind to specific *cis*-acting elements present over the promoter region of the target genes and modulate their expression at transcription level (Ramirez and Basu, 2009). For obvious reasons, transcription factors are central to the regulation of different biological processes and are important molecular components in cellular signal transduction machinery. Plants being sessile have evolved elaborate mechanism to adapt under varying sets of conditions. In this regard, transcription factors are instrumental in controlling and fine-tuning plant growth and development under favorable and adverse conditions (Seo and Choi, 2015). Thus, investigation of functional aspects of transcription factor genes is desirable for gaining fundamental knowledge about molecular basis of biological processes. On the basis of conserved DNA binding domains, transcription factors are differentiated into families (Agarwal et al., 2015). Certain transcription factor families are specific to plant lineage suggesting their importance in plant specific processes. Homeodomain-zipper (HDZ) family of transcription factors are exclusively restricted to plant lineage, and characterized by the presence of conserved DNA binding domains namely homeodomain (HD) and a leucine zipper domain (LZ), required for mediating protein-protein interaction (Ariel et al., 2007). Based on the presence and absence of additional conserved domains, the HDZ family has been further classified into four subfamilies from I to IV. The members of subfamilies III and IV of HDZ transcription factors possess additional conserved domains namely the steroidogenic acute regulatory protein-related lipid transfer (START) domain and the START adjacent domain (SAD). Another additional conserved domain named as MEKHLA domain is present only in the members of HDZ III subfamily.

Genome-wide identification of HD-ZIP IV genes has been carried out in different plants including *Arabidopsis*, rice, maize, soybean, and cucumber (Nakamura et al., 2006; Jain et al., 2008; Javelle et al., 2011; Fu et al., 2013; Zalewski et al., 2013; Belamkar et al., 2014). These genes primarily express in the outer most layer of shoot apical meristem and in epidermal cells of plant organs (Javelle et al., 2011; Chew et al., 2013). The functional characterization and expression analysis suggested that HD-ZIP IV transcription factors play major role in developmental regulation of epidermal structures such as trichomes, stomata and root hairs as well as in cuticle development (Ariel et al., 2007). In *Arabidopsis*, two closely related and functionally redundant HDZIV genes, namely *PDF2* and *ML1* have been implicated in regulation of embryo and epidermis development as well as in determination of floral identity (Kamata et al., 2013; Ogawa et al., 2015). The *Arabidopsis* HD-ZIP IV transcription factor, GL2 controls trichome development and root hair patterning (Wang et al., 2007). AtANL2, another Arabidopsis HD-ZIP IV transcription factor regulates anthocyanin deposition in sub-epidermal cells, epidermal cell proliferation, and root development (Kubo et al., 1999). Heterologous and homologous overexpression of Arabidopsis *AtHDG11* gene led to the drought tolerant phenotype, which was attributed to improved root system and reduced stomatal density in transgenic plants (Yu et al., 2008, 2013). Functional analysis of some of the HD-ZIP IV genes has also been carried out in other plants such as maize and tomato. In maize, cuticle deposition and kernel development were reported to be regulated by HD-ZIP IV transcription factor, ZmOCL1 (HD-ZIP IV1), while the ZmOCL4 (HD-ZIP IV4) was implicated in regulation of development of anther wall as well as trichomes (Vernoud et al., 2009; Javelle et al., 2010). Based on the aforementioned, it can be concluded that HD-ZIP IV family transcription factors play key role in regulation of plant-specific developmental aspects and, therefore deserves thorough investigation for their functional attributes in different crop plants.

Banana is a staple fruit crop for a major world population, especially in developing countries (Bapat et al., 2010). Certain agronomic traits such as fruit quality and stress resistance are of great importance in this plant species. For various reasons, banana improvement through breeding exercises has been challenging. Therefore, genetic engineering based manipulations holds great promise for the crop improvement however, genes required for this purpose needs to be identified from banana. In past, a number of genes with modulated expression during banana fruit ripening were identified (Kesari et al., 2007), however none of these genes belonged to the analysis of gene families. Recent release of banana genome sequence provides useful resource to understand functional genomics of useful agronomic traits and to identify candidate genes to be utilized in banana improvement programme (D'Hont et al., 2012). Despite the availability of comprehensive genome resource of banana, there are limited reports concerning with gene family characterizations. To the best of our knowledge, as of now, exhaustive identification of gene families in banana has been limited to MAPK genes (Asif et al., 2014), genes involved in ethylene biosynthesis and perception (Jourda et al., 2014), NAC domain transcription factors (Cenci et al., 2014), and AP2/ERF transcription factors (Lakhwani et al., 2016). In the present work, we have carried out genome-wide identification of HD-ZIP subfamily IV (HD-ZIP IV) transcription factors in banana. Furthermore, we have also studied expression profile of individual genes of this family during plant growth development, fruit ripening as well as during stress response. Taken together, in this study, we have developed a comprehensive resource of HD-ZIP IV gene family in banana, which could be used as a foundation for elucidation of their functions and for deciphering their regulatory networks.

## Materials and methods

### Computational identification and analysis of banana HDZIV genes

The proteins corresponding to the HDZ IV genes of maize, rice and Arabidopsis, as described in Javelle et al. (2011) were used as query in BLAST P searches in banana genome database (http://banana-genome.cirad.fr/) (D'Hont et al., 2012). The banana proteins, resulting from each blast search (E-value, 10^−5^) were pooled and redundant sequences were removed. The protein sequences, thus retrieved were aligned with that of maize, rice and Arabidopsis. Protein sequences containing MEKHLA domain were removed. Further refinement of gene models of banana HDZIV proteins was carried out by retrieving corresponding genomic sequences and by using Fgenesh programme (www.omictools.com/fgenesh-s1037.html). Finally, manual inspection of intron and exon boundaries was carried out. The prediction of molecular weight and PI was done by using tools available at Expasy bioinformatics resource portal. The prediction of *Arabidopsis* homologs of banana HDZIV genes was carried out by BLAST P searches in The Arabidopsis Information Resource (TAIR; http://www.arabidopsis.org; TAIR10). Identification of conserved motif among banana HDZIV proteins was done by using MEME tool (http://meme-suite.org/tools/meme). The identified motifs, as represented by logos were manually inspected for presence of elements, representing conserved domains of HDZIV proteins.

### Intron- exon composition and identification of conserved motifs in 3′UTR

The analysis of intron and exon composition was carried out by using tool hosted at http://gsds.cbi.pku.edu.cn/. For identification of conserved motifs in 3′UTR regions (Javelle et al., 2011; Zalewski et al., 2013) of banana HDZIV genes, a region of 500 nucleotides, downstream to the stop codons of HDZIV ORF, was considered as putative 3′UTR. This region was scanned for the presence of conserved motifs of 19 and 21 nucleotides. The corresponding sequences were retrieved and used for development of sequence logos by using web logo tool hosted at http://weblogo.berkeley.edu/logo.cgi.

### Alignment, phylogenetic analysis, chromosomal localization, and gene duplication

Multiple sequence alignment was performed by using Clustal X programme. Individual domains in the protein sequences were identified and manually highlighted. The phylognetic tree was constructed by neighbor joining method with 1000 bootstrap values. For the purpose, Mega 5 program was used with Dayhoff model and pairwise deletion (Tamura et al., 2011). The chromosomal mapping of individual HDZIV genes was carried out by performing BLASTX searches in a local database of banana genome sequence. In order to identify segmental and tendem duplications, synteny analysis among banana chromosomes was carried out by using SynMap tool available at CoGe server (https://genomevolution.org/CoGe/SynMap.pl) (Lyons and Freeling, 2008). The syntenic chromosome regions were manually searched for presence of HDZIV genes. Gene duplication events were concluded if closely related banana HDZIV genes were found to be located within syntenic regions.

### Prediction of *cis*-regulatory elements

For identification of *cis*-regulatory elements in the banana HDZIV genes, a 1.5 kb region upstream to the translation start codon was considered as proximal promoter. In the case of HDZIV5 (locus id GSMUA_Achr3P23090_001), genomic information was not available for the upstream region, and therefore was not included in the analysis. Further, in the case of MaHDZIV1 (locus id GSMUA_Achr1P18760_001), the genomic information was available for 1056 bps only. The upstream regions were analyzed using Plant Care database and *cis*-regulatory elements were retrieved (Lescot, 2002).

### Plant material and various treatments

Dessert banana (*M. acuminate*, AAA genome) was used for gene expression analysis in the present study. The vegetative tissues such as different developmental stages of leaves, different layers of pseudostem, bract, fruit fingers, fruit peel, and fruit pulp etc. were harvested from field grown banana plants and stored at −80°C, following freezing in liquid nitrogen. The *in-vitro* tissues (non-embryogeneic callus culture and somatic embryo) were developed using male flowers as explants and as per previously described protocol (Côte et al., 1996). For imposition of water stress, *in vitro* grown banana plantlets were taken out of culture vessels, kept in filter paper and exposed to air. The plantlets were collected at varying time points of stress treatment and used for RNA isolation. NaCl (250 mM) was used for evoking salinity stress in banana plantlets as described by Sreedharan et al. (2012). In each case of stress treatment, samples were collected in triplicates.

### Total RNA isolation, cDNA synthesis, and gene expression analysis

Total RNA was isolated from banana tissues according to the previously described protocol (Asif et al., 2006). Each RNA sample was treated with DNase I Digest kit (Sigma-Aldrich, USA) to eliminate DNA contamination. The integrity and size distribution of total RNA was analyzed by agarose gel electrophoresis. A NanoQuant (Infinite® 200 PRO NanoQuant, Austria) was used for RNA quantification. DNA-free RNA (5 μg) was used for synthesis of first strand of cDNA by using Revert Aid First Strand cDNA synthesis Kit (Fermentas, USA) as per manufacturer's recommendations.

The quantitative real-time PCR expression was carried out with an ABI 7700 Sequence Detector (Applied Biosystems, USA). The transcripts were quantified by SYBR Green chemistry. The amount of cDNA was normalized by using amplification of housekeeping banana *actin* as an internal control. The data from real-time PCR amplification was estimated in terms of comparative fold expression following 2^−ΔΔct^ method (Livak and Schmittgen, 2001). The list of different primers used in the study is given in the Supplementary Table 1. Each reaction was performed in 20 μl (total volume) and consisted of 1X SYBR Green Master mix (Applied Biosystems, USA), 5 pmol of each primer, 1 μl cDNA template and sterile H_2_O. The steps performed during real-time PCR experiment were as follows: step (1) 50°C, 2 min; step (2) 95°C, 10 min; step (3) (95°C, 0.15 min; 60°C, 1 min) × 40 cycles.

## Results

### Identification of HDZ subfamily IV genes in banana

To comprehensively identify banana HDZIV genes, blast searches in banana genome database was carried out using known HDZIV proteins from Arabidopsis (16 genes), maize (17 genes), and rice (11 genes) as query sequences. The banana protein sequences, thus retrieved were analyzed by carrying out multiple sequence alignment with other known HDZIP proteins. However, some of the sequences were found to have deleted regions of varying amino acid length suggesting requirement of further refinement in gene model prediction. Therefore, corresponding genomic sequences were retrieved and gene models were modified using online tool (Fgenesh program) followed by manual analysis of intron–exon boundaries (Supplementary File S1). The deduced amino acid sequences from these refined gene models displayed all conserved domain and exhibited good alignment with other known HDZIV proteins. Following this, a total of 21 genes encoding putative HDZIP subfamily IV transcription factors in banana were identified. These HDZIV proteins contained characteristic domains namely HD, LZ, START, and SAD domains, and were devoid of HDZ sub family III specific MEKHLA domain (Supplementary Figure S1). These putative banana HDZIV proteins were named as MaHDZIV1 to HDZIV21, based on their positioning on banana linkage groups. Various features of identified banana HDZIV genes such as locus ids, chromosomal coordinates, predicted protein molecular weight and their closest Arabidopsis homologs are summarized in the Table 1.

**Table 1:** **Structural features of *MaHDZIV* genes in banana**.

**Genome sequence locus id**	**Name**	**Start**	**End**	**Amino acid**	**MW (kDa)**	**PI**	**Best hit**
GSMUA_Achr1P18760_001	*MaHDZIV1*	14012496	14015584	725	79.16	5.58	AT4G00730 (ANL2)
GSMUA_Achr2P15140_00	*MaHDZIV2*	16568570	16574422	706	78	5.86	AT1G73360 (HDG11)
GSMUA_Achr3P12410_001	*MaHDZIV3*	9200250	9203613	805	87	6.18	AT3G61150 (HDG1)
GSMUA_Achr3P15400_001	*MaHDZIV4*	16230400	16235306	742	82.5	6.03	AT1G79840 (GL2)
GSMUA_Achr3P23090_001	*MaHDZIV5*	23918711	23921733	683	74.5	5.8	AT4G04890 (PDF2)
GSMUA_Achr4P19010_001	*MaHDZIV6*	20045866	20050132	800	88	5.7	AT5G46880 (HDG5)
GSMUA_Achr5P10510_001	*MaHDZIV7*	7520973	7525132	803	86	6.2	AT4G00730 (ANL2)
GSMUA_Achr5P14100_001	*MaHDZIV8*	10146248	10149653	804	88.4	6	AT5G46880 (HDG5)
GSMUA_Achr5P20050_001	*MaHDZIV9*	21687311	21691138	804	86.3	6.4	AT4G00730 (ANL2)
GSMUA_Achr6P05990_001	*MaHDZIV10*	4042260	4045542	697	77	6.1	AT1G73360 (HDG11)
GSMUA_Achr7P00210_001	*MaHDZIV11*	204838	208547	716	79	5.2	AT4G04890 (PDF2)
GSMUA_Achr7P11310_001	*MaHDZIV12*	9019114	9023562	696	77	5.9	AT1G73360 (HDG11)
GSMUA_Achr8P07310_001	*MaHDZIV13*	4828124	4831306	757	82	5.7	AT4G04890 (PDF2)
GSMUA_Achr8P27340_001	*MaHDZIV14*	30544129	30548360	748	84	6.6	AT1G79840 (GL2)
GSMUA_Achr10P04900_001	*MaHDZIV15*	14541037	14544729	736	81	5.7	AT1G05230 (HDG2)
GSMUA_Achr10P17790_001	*MaHDZIV16*	24840086	24843716	696	77	7.5	AT1G73360 (HDG11)
GSMUA_Achr10P25900_001	*MaHDZIV17*	29702661	29705784	700	77	5.6	AT1G73360 (HDG11)
GSMUA_Achr11P03290_001	*MaHDZIV18*	2365748	2369916	802	86	6	AT4G00730 (ANL2)
GSMUA_Achr11P21700_001	*MaHDZIV19*	22201669	22205602	789	85	6	AT4G00730 (ANL2)
GSMUA_Achr11P25820_001	*MaHDZIV20*	24833194	24837205	771	84	5.6	AT4G04890 (PDF2)
GSMUA_AchrUn_randomP10540_001	*MaHDZIV21*	51152182	5156811	811	87	5.6	AT4G00730 (ANL2)

### Phylogenetic analysis of banana HDZIP IV proteins

**Figure 1:**
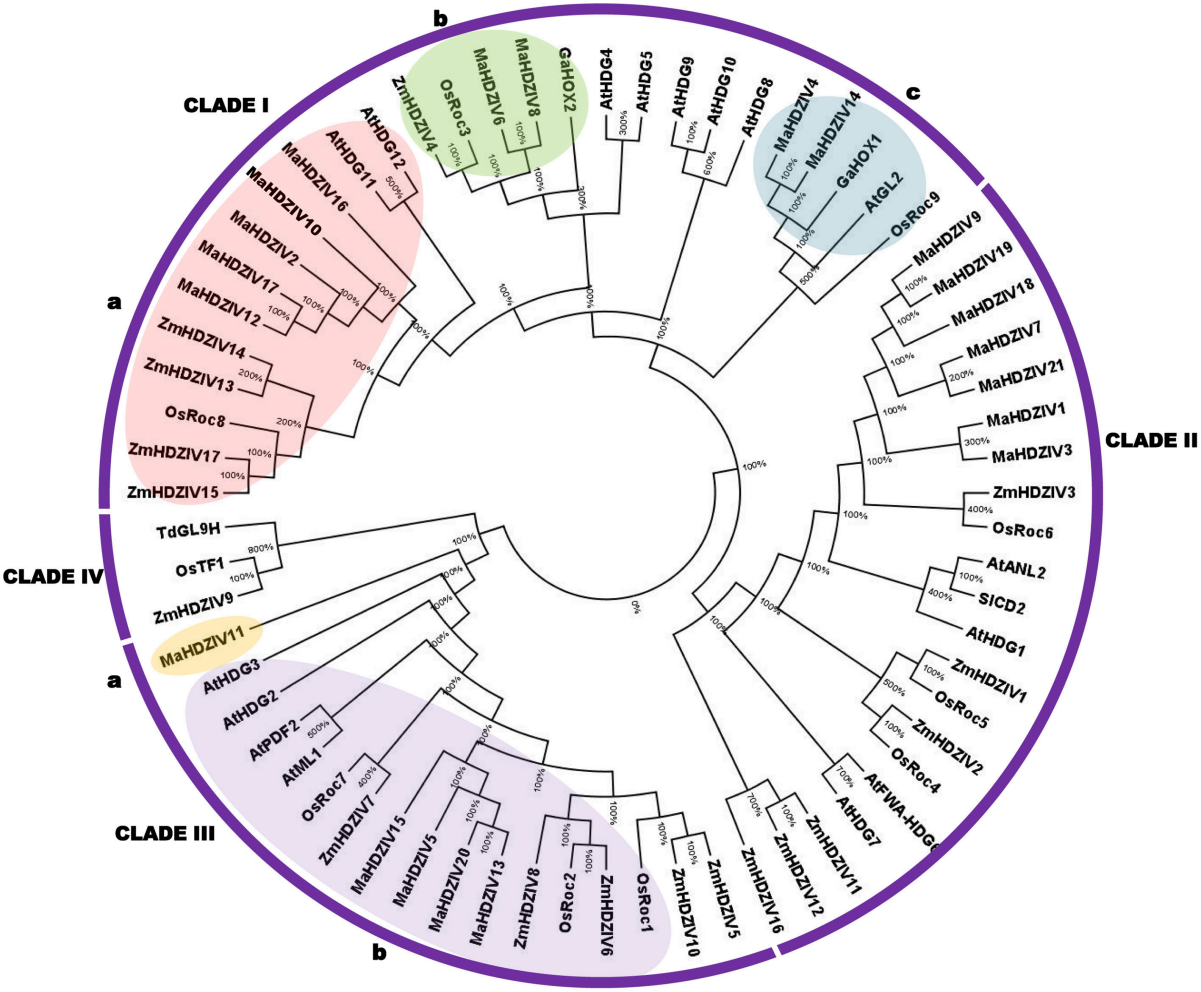
**Phylogenetic tree of HDZIV proteins**. The unrooted phylogenetic tree was constructed using NJ method and with 1000 bootstrap value. Within clades I and III, the discrete groups (a and b) of banana HDZIV proteins have been represented in boxes. The protein sequences corresponding to rice, maize and Arabidopsis were taken as described in Javelle et al. (2011). The proteins from other plant species are *Triticum turgidum* subsp. durum GL9H1 (TdGL9H1; accession number AEI99593), *Solanum lycopersicum* cutin deficient 2 (SlCD2; accession number NP_001234657), *Gossypium arboretum* HOX1 (GaHOX1 accession number; ABY41242), and *Gossypium arboretum* HOX2 (GaHOX2; accession number; ABY67263).

To gain knowledge about phylogenetic relationship of HDZIV proteins from banana and from other plants (rice, maize, and Arabidopsis), a phylogenetic tree was constructed using NJ method. Certain functionally characterized HDZIV proteins from tomato, cotton and wheat were also included in the analysis. The HDZIV proteins were grouped in four distinct clades, named as I to IV (Figure 1). Most of the HDZIV proteins from banana and other monocots got clustered together and thereby formed monocot-specific clusters in their respective clades. In these monocot-specific groups, whereas the HDZIV proteins from rice and maize were interspersed, the related banana HDZIP proteins grouped together and thereby formed distinct clusters within the groups. In certain groups, there were more paralogs for banana HDZIV genes as compared to that of other monocots. Interestingly, two banana HDZIV proteins, MaHDZIV4, and MaHDZIV14 grouped with dicot HDZIV proteins. In the clade III, MaHDZIV11 appears to be unique to banana as it is highly divergent from rest of the proteins in its respective clade. There was no representation from banana in the clade IV, which otherwise, seems to be a monocot-specific clade of HDZIV proteins.

### Conserved motifs in banana HDZ IV proteins

**Figure 2:**
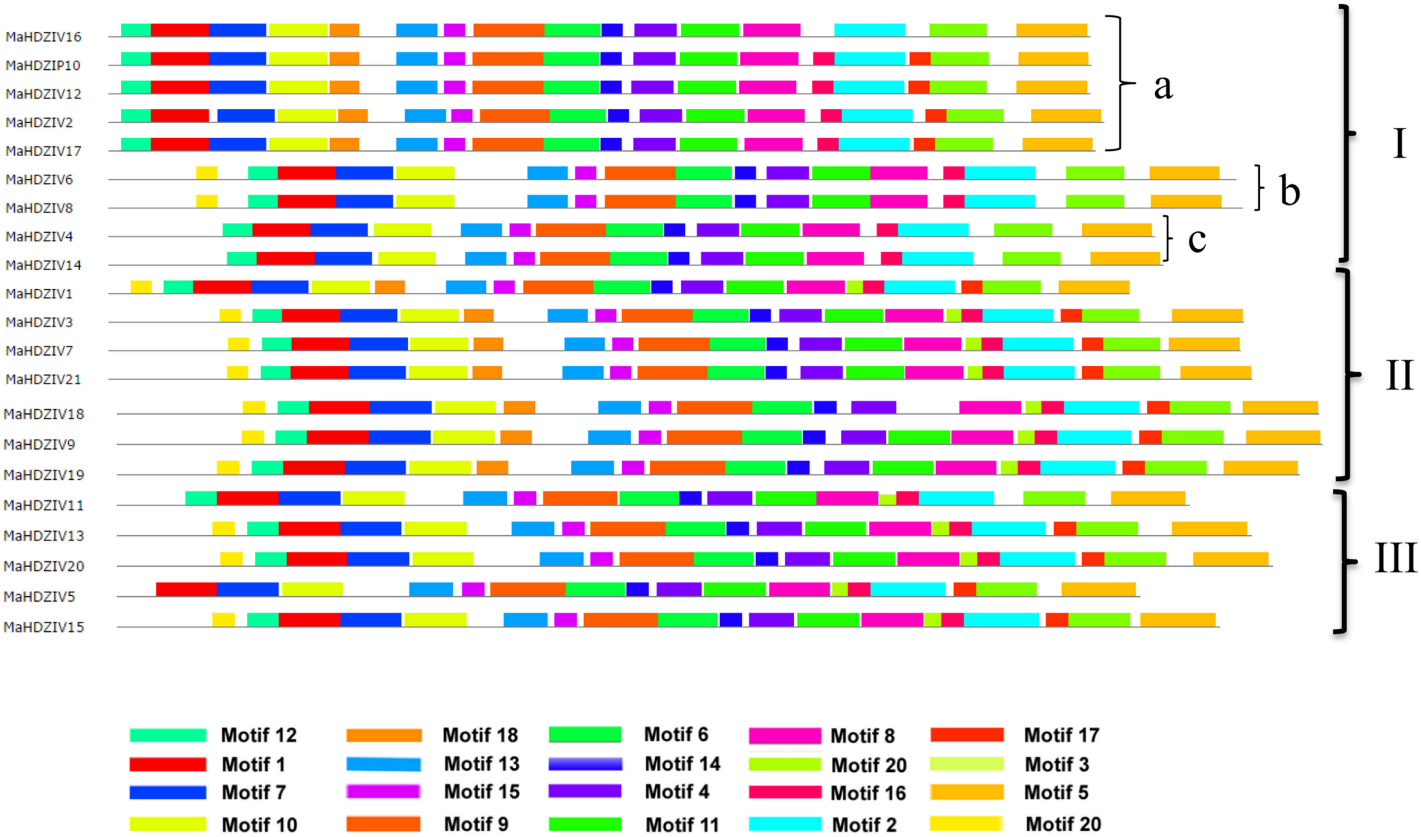
**Schematic representation of conserved motifs in banana HDZIV proteins**. A total of 20 conserved motifs were queried within all banana HDZIV proteins using MEME tool. The motifs have been represented in different colors. The banana HDZIV proteins are arranged according to their position in the phylogentic tree (Figure 1). The sequence logo corresponding to these conserved motifs are given in Supplementary Figure S2.

The deduced amino acid sequences corresponding to the banana HDZ proteins were further analyzed for comprehensive prediction of conserved motifs using MEME suite (Figure 2 and Supplementary Figure S2). A total of 20 conserved motifs were queried in 21 banana HDZIV proteins. Motifs 12 and 1 corresponded to the conserved HD, and motifs 7 and 10 belonged to the LZ domain. The START domain was represented by motifs 13, 15, 9, 6, 14, and 1. The rest of the predicted motifs were mostly associated with the SAD domain. While motifs representing HD, LZ, and START domains, the characteristic domains of HDZIV family proteins were present in all the 21 banana HDZ proteins, the SAD domain, in particular exhibited differences in terms of motif compositions in different HDZ proteins. We observed that closely related banana HDZ proteins displayed more or less similar compositions of motifs. For example, all banana HDZIP proteins, except for the MaHDZIV16, in cluster- a of the clade 1 have a total of 17 conserved motifs and thereby seem to be very similar.

### Gene structure and nucleotide conservation in 3′UTR region

**Figure 3:**
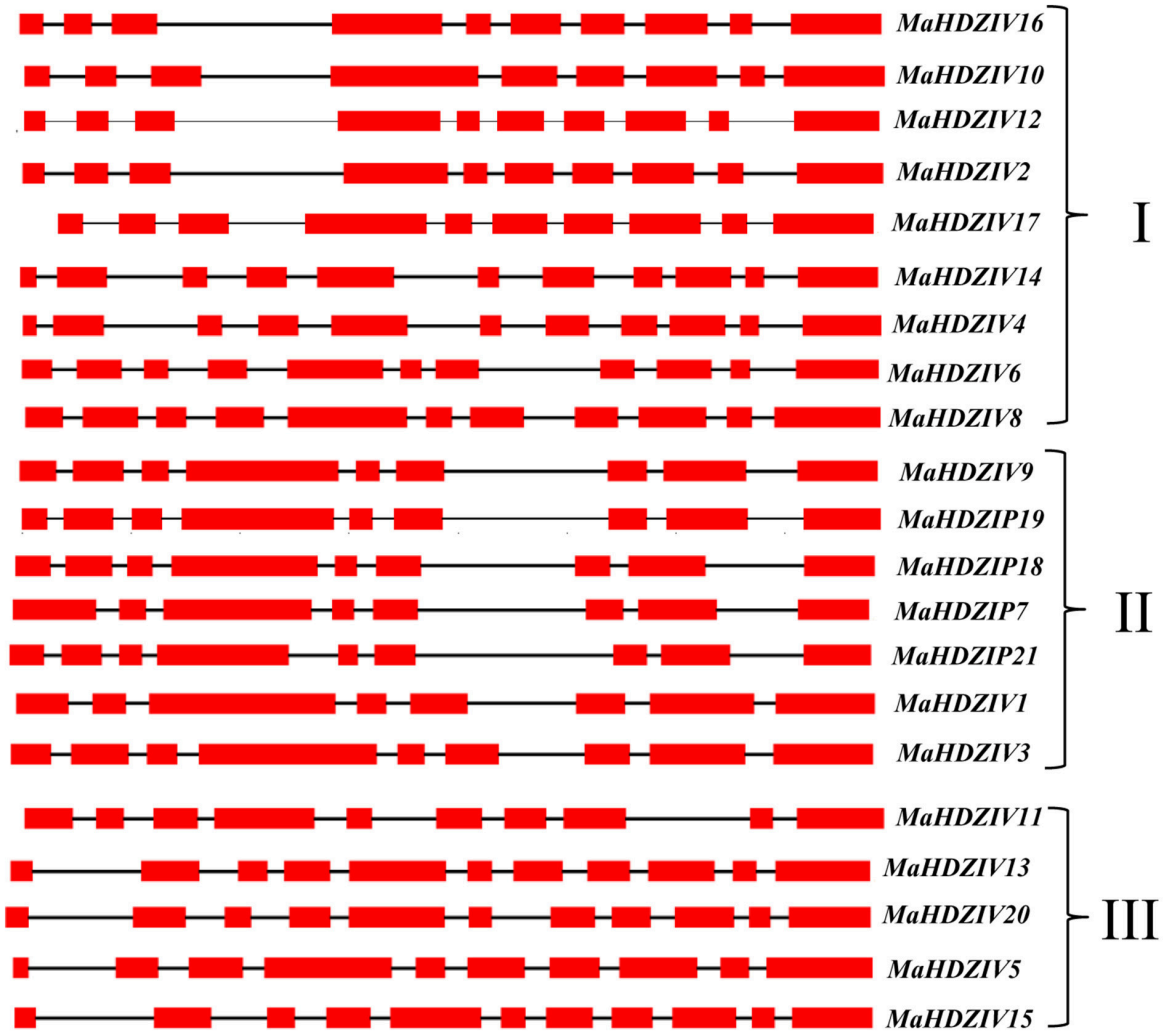
**Schematic representation of intron- exon composition of banana HDZIV genes**. The genes showing exons (in red boxes) and introns (in black lines) are grouped as per their positioning in the phylogentic tree. Complete coding region was used for the analysis. I, II, and III represent different clades.

In order to gain information regarding gene structure, genomic regions corresponding to banana HDZIV genes were analyzed for the architecture of introns and exons. The numbers of exons and introns among banana HDZIV genes varied between 8 to 11 and 7 to 10, respectively (Figure 3). Further, it was observed that most of the closely related HDZIV genes have similar profiles of introns and exons. Thus, phylogenetic grouping of HDZIV proteins is in accordance with exon- intron architectures of corresponding genes.

**Figure 4:**
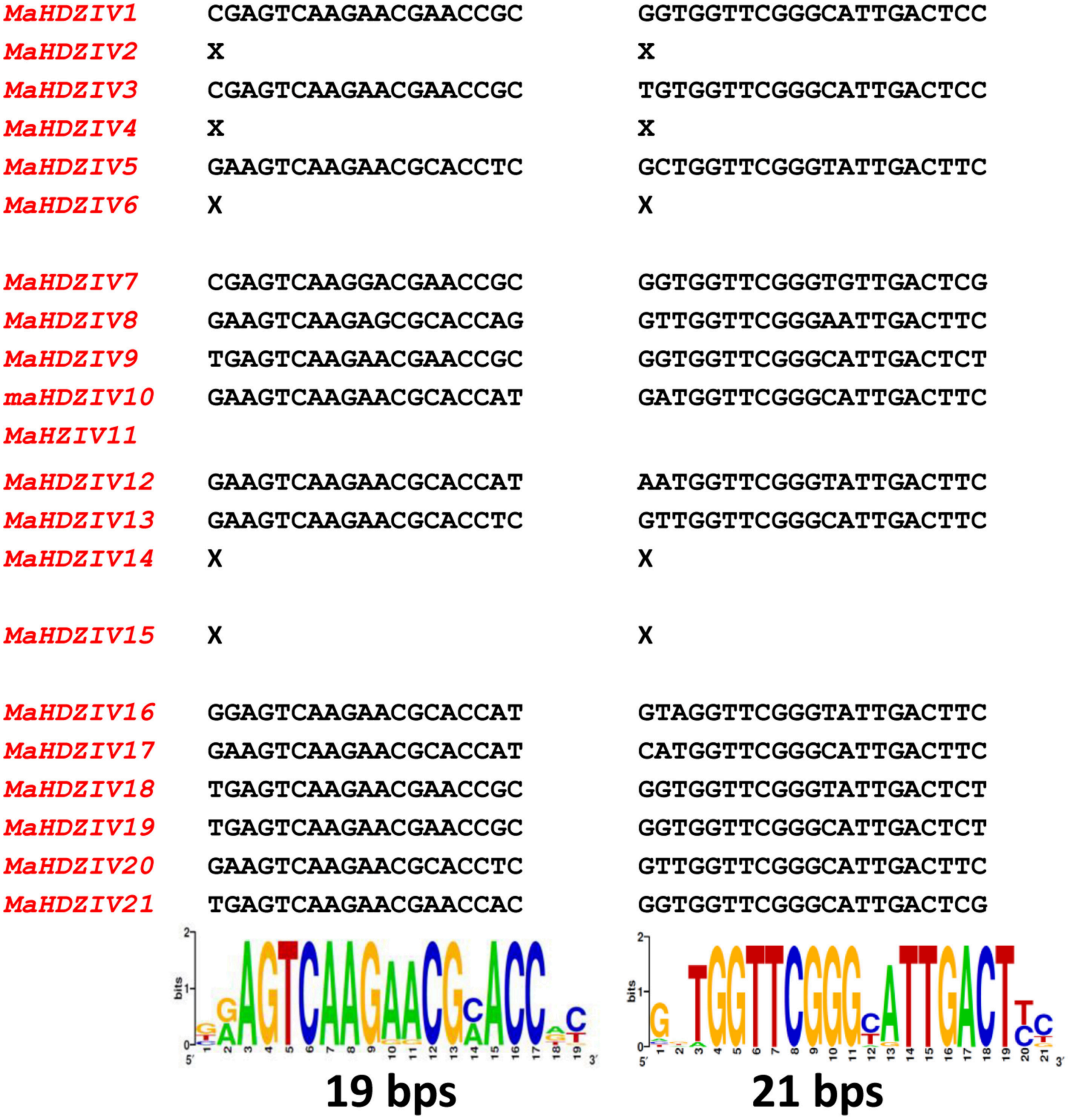
**Conserved motifs in 3′UTRs of banana HDZIV genes**. The conserved motifs of 19 and 21 bps are represented in the form of sequence logo. The X mark indicates the absence of conserved motifs in the respective banana HDZIV gene.

In earlier works, two short conserved regions have been reported to be present in 3′ UTRs of HDZ sub family IV genes (Javelle et al., 2011; Zalewski et al., 2013). Our analysis also revealed the presence of similar conserved motifs of 19 and 21 nucleotides in length in the 3′UTRs of all banana HDZ genes excluding the *MaHDZIV14, MaHDZIV15, MaHDZIV6, MaHDZIV11 MaHDZIV4*, and *MaHDZIV2* (Figure 4). Notably, both the motifs were either simultaneously present or altogether absent in some of these banana HDZ genes.

### Chromosomal localization and gene duplication

**Figure 5:**
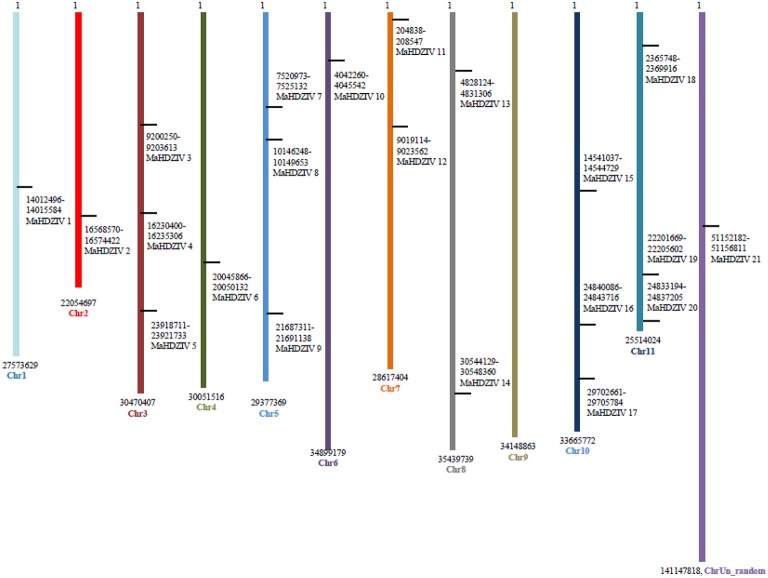
**Chromosomal localization of banana HDZIV genes**. The black lines on the chromosomes (vertical) indicate the positions of the respective genes. Numbers represent nucleotide base pair positions.

All *MaHDZIV* genes except for the *MaHDZIV21*, which is associated with an uncharacterized scaffold, could be mapped on banana chromosomes (Figure 5). No HDZIV gene was present on the chromosome 9. The each of the chromosomes 2, 5, 10, and 11 contained three HDZIV genes. There were two HDZIV genes on the chromosome 7 while each of the remaining chromosomes had one HDZIV gene. Further, by using CoGe Syn-Map tool, we assessed possibilities of tandem and segmental duplication in connection with the evolution of banana HDZ genes. Several closely related banana HDZIV genes were found to be located over syntenic regions of different chromosomes. For example, *MaHDZIV17, MaHDZIV2* and *MaHDZIV12* are localized on chromosomes 10, 2, and 7, respectively though chromosomal segments corresponding to these genes are syntenic to each another. However, no instance of tandem duplication associated with any of the closely related gene pair could be revealed. Thus, we conclude that evolution of banana HDZIV gene family could primarily be attributed to the process of segmental duplication.

### Differential gene expression

**Figure 6:**
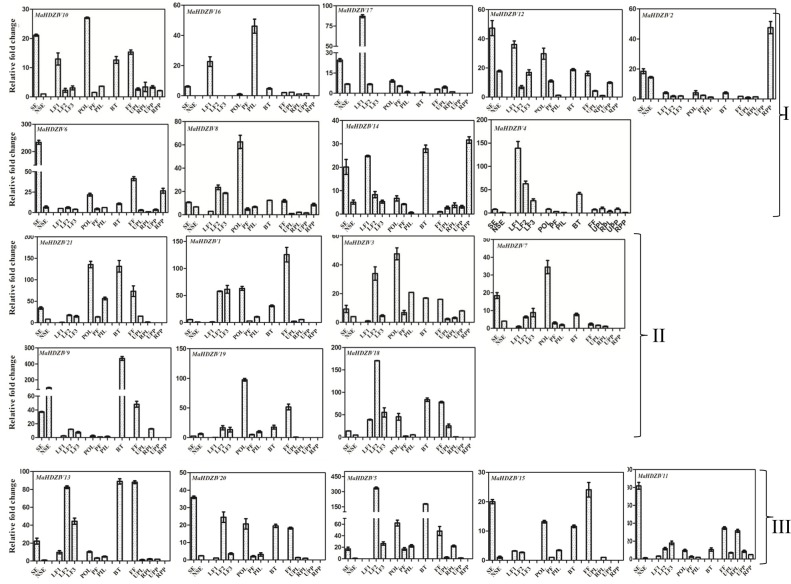
**Expression analysis of banana HDZIV genes in different tissues**. The different tissues are BT, Bract; FF, Fruit fingers; LF1, Leaf development stage 1; LF2, Leaf development stage 2; LF3, Leaf development stage 3; NSE, Non embryogenic callus; PIL, Pseudostem inner most tissue; PF, Pseudostem middle fibrous layer; POL, Pseudostem outer layer; RPL, Ripe banana peel; RPP, Ripe banana pulp; SE, Somatic embryo; UPL, Unripe banana peel; and UPP, Unripe banana pulp. In each case, expression level is expressed as relative fold change as compared to the tissue with the lowest expression level. The graphs are arranged as per the grouping in phylogentic tree.

Expression analysis of banana HDZIV genes in different plant parts such as leaves, pseudostem, and fruits as well as in *in vitro* tissue such as somatic embryos and non-embryogenic callus was carried out (Figure 6). All the banana HDZIV genes were expressed in different tissues at varying levels. Based on the expression profile in different leaf samples, two sets of banana HDZIV genes could be identified. In one set, the genes *MaHDZIV9, MaHDZIV8, MaHDZIV18, MaHDZIV7, MaHDZIV21, MaHDZIV*3, *MaHDZIV11, MaHDZIV13*, MaHDZIV*20*, and *MaHDZIV5* exhibited an up-regulation trend from young to mature leaves. Conversely, another set of genes consisting of *MaHDZIV14, MaHDZIV4, MaHDZIV17, MaHDZIV12, MaHDZIV10, and MaHDZIV16* displayed a down-regulation pattern during leaf maturation. The transcripts of several genes were also detected in different tissues corresponding to banana fruit; however, in most of the cases, it was not as pronounced as in other tissues. Among these genes, the *MaHDZIV14* and *MaHDZIV2* were remarkable as their transcript levels were reported to be highly abundant in ripe banana pulp suggesting that these might be involved in some aspects of fruit ripening. In the pseudostem, several genes were up-regulated in the outer layer as compared to the inner layers. The transcripts of most of the HDZIV genes could also be scored in somatic embryos and non embryogenic callus. Among these, most of the genes were found to be highly expressing in somatic embryos as compared to the non-embryogenic callus. Among these, the most significant were *MaHDZIV7, MaHDZIV11, MaHDZIV13, MaHDZ-IV20, MaHDZIV15, MaHDZIV14, MaHDZIV10, MaHDZIV16*, and *MaHDZIV6*, whose transcripts were highly abundant in somatic embryos. Furthermore, several closely related HDZIV genes showed similar expression patterns. For example, the expression pattern of phylogentically closely related genes, *MaHDZIV13, MaHDZIV20, MaHDZIV5, and MaHDZIV15* was similar to the some extent. Similar observations were made with the cluster consisting of *MaHDZIV7, MaHDZIV21, MaHDZIV19*, and *MaHDZIV9* genes as well as with the gene pair of *MaHDZIV6* and *MaHDZIV8*.

### Prediction of *cis*-acting elements in the proximal promoter

In order to get insight into the transcriptional regulation of banana HDZIV genes, *cis*-acting elements in their upstream regions were predicted (Table 2). The *cis*-elements involved in growth and development responses such as Skn-1 and GCN4 motifs, RY-elements etc. were detected in several banana HDZIV genes. Further, we observed that there was an overrepresentation of motifs involved in light regulated expression such as G-box, Sp1 element, TCC, GATA box etc. In addition, presence of several *cis* acting elements associated with biotic and abiotic stress response, e.g., MBS, ABRE, TCA, and TGA elements, GCC box etc. in the upstream regions of HDZ IV genes were also identified. Presence of a putative *cis*-acting elements known to participate in various functions in promoter regions of banana HDZIV genes suggest that they could be co-ordinately regulated by multiple transcription factors and appears to be modulated by multiple exogenous and endogenous factors.

**Table 2:** ***cis*-regulatory elements present in the promoters of *MaHDZIV* genes in banana**.

**Gene name**	**Motifs related to growth and development**	**Motifs related to light response**	**Motifs related to stress response**
*MaHDZIV1*	GCN4_motif, Skn-1_motif, Sp1	CATT-motif, GAG-motif, Gap-box, TCCC-motif,	GARE-motif, TCA-element, Box-W1, W box
*MaHDZIV2*	CAT-box, CCGTCC-box, as-2-box, O2-site	3-AF1 binding site, ACE, Box I, Box II, CATT-motif, G-box, GA-motif, I-box, Sp1, TCCC-motif, TGG-motif	MBS, TC-rich repeats, ABRE, CGTCA-motif, GARE-motif, P-box, TCA-element, TGA-element, TGACG-motif, ARE
*MaHDZIV3*		Sp1, TCCC-motif, TCT-motif	TCA-element, TGA-element, TGACG-motif, ARE, W box
*MaHDZIV4*	CAT-box, CCGTCC-box, GCN4_motif, RY-element, Skn-1_motif, circadian	AE-box, GAG-motif, GT1-motif, I-box, Sp1, TCCC-motif, Box I, GA-motif	Box-W1, MBS, TATC-box, TC-rich repeats, TCA-element, ARE, W box, box E
*MaHDZIV6*	CAT-box, CCGTCC-box, circadian, Skn-1_motif, O2-site,	AE-box, G-Box, I-box, MRE, Sp1, TCT-motif, box II, GC-motif,	LTR, MBS, TC-rich repeats, ABRE, CE3, CGTCA-motif, GARE-motif, TATC-box, TCA-element, TGACG-motif, ARE, Box-W1, W box
*MaHDZIV7*	CCGTCC-box, circadian, Skn-1_motif, GCN4_motif,	CATT-motif, G-box, GAG-motif, GT1-motif, sp1, MRE, GC-motif,	TC-rich repeats, ABRB, AuxRR-core, CGTCA-motif, P-box, TCA-element, TCCC-motif, TGA-element, TGACG-motif, W box, MBS, LTR, Box-W1, ARE
*MaHDZIV8*	CAT-box, Skn-1_motif, circadian, O2-site	ACE, Box 4, CATT-motif, G-Box, GAG-motif, Sp1, TCCC-motif, box II, TCCC-motif, TCT-motif	Box-W1, MBS, WUN-motif, TC-rich repeats, ABRE, CGTCA-motif, GARE-motif, SARE, TATC-box, TCA-element, TGA-element, TGACG-motif, TCA-element, TGACG-motif, W box, CE3
*MaHDZIV9*	CAT box, CCGTCC-box, circadian, O2-site,	AE-box, Box 4, CATT-motif, GA-motif, Sp1, TCCC-motif, rbcS-CMA7a, MRE,	GARE motif (-),CGTCA-motif, TCA-element, HSE, LTR, TC-rich repeats
*MaHDZIV10*	3-AF1 binding site, GCN4_motif, as-2-box, circadian	ATC-motif, ATCT-motif, Box 4, Box I, GAG-motif, GATA-motif, I-box, Sp1, TCCC-motif, TCT-motif, chs-CMA1a, GC-motif,	HSE, LTR, TGACG-motif, CGTCA-motif, P-box, TCA-element, ARE, EIRE
*MaHDZIV11*	CAT-box, GCN4_motif, RY-element, circadian, O2-site, AC-II,	ATCT-motif, CATT-motif, G-box, GAG-motif, I-box, Sp1, TCCC-motif, TCT-motif	Box-W1, WUN-motif, AuxRR-core, TCA-element, TGA-element, W box, GCC box,
*MaHDZIV12*	Skn-1_motif,	ACE, CATT-motif, G-Box, GAG-motif, GATA-motif, Sp1, TCCC-motif	Box-W1, LTR, MBS, ABRE, CGTCA-motif, GARE-motif, TCA-element, TGACG-motif, TGG-motif, W box, ARE, EIRE
*MaHDZIV13*	GCN4_motif, RY-element, Skn-1_motif, circadian, AC-I, O2-site	ATC-motif, Box 4, G-Box, GAG-motif, GATA-motif, I-box, Sp1	Box-W1, MBS, TC-rich repeats, ABRE, CGTCA-motif, TCA-element, TGA-element, TGACG-motif, ARE, W box
*MaHDZIV14*	GCN4_motif, Skn-1_motif	Box 4, Box I, G-Box, GT1-motif, MRE, Sp1, TCT-motif, Box III	MBS, ABRE, CGTCA-motif, GARE-motif, TGACG-motif, ARE
*MaHDZIV15*	RY-element, Skn-1_motif, O2-site	Box 4, GA-motif, GAG-motif, GATA-motif, I-box, Sp1, TCCC-motif, TCT-motif	HSE, MBS, TC-rich repeats, WUN-motif, CGTCA-motif, TCA-element, TCCACCT-motif, TGACG-motif
*MaHDZIV16*	Skn-1_motif, circadian	G-Box, GA-motif, GAG-motif, I-box, L-box, LAMP-element, Sp1, TCCC-motif, TCT-motif	Box-W1, MBS, ABRE, ARE, CGTCA-motif, P-box, TATC-box, TCA-element, TGACG-motif, W box
*MaHDZIV17*	CCGTCC-box, GCN4_motif, Skn-1_motif, circadian, O2-site	AE-box, ATC-motif, Box I, CATT-motif, G-box, GAG-motif, GATA-motif, Sp1, TCCC-motif	LTR, CGTCA-motif, P-box, TCA-element, TGACG-motif, ARE, box E
*MaHDZIV18*	CCGTCC-box, circadian, dOCT, AC-II,	AE-box, Box I, I-box, Sp1, TCCC-motif, chs-CMA1a, GC-motif,	Box-W1, HSE, MBS, CGTCA-motif, ERE, GARE-motif, TCA-element, TGA-element, TGACG-motif, W box
*MaHDZIV19*	CAT-box, Skn-1_motif, circadian, AC-I	AE-box, Box 4, G-box, GATA-motif, I-box, Sp1	Box-W1, HSE, LTR, WUN-motif, ABRE, CGTCA-motif, ARE,
*MaHDZIV20*	CAT-box, CCGTCC-box, Skn-1_motif	ATC-motif, CATT-motif, G-Box, GAG-motif, GATA-motif, Gap-box, I-box, MNF1, Sp1, TCCC-motif, TCT-motif	Box-W1, HSE, TC-rich repeats, CGTCA-motif, GARE-motif, TCA-element, TGACG-motif, W box
*MaHDZIV21*	3-AF1 binding site, GCN4_motif, Skn-1_motif, O2-site,	G-Box, GAG-motif, GATA-motif, Sp1, TCCC-motif, TCT-motif, GC-motif	TC-rich repeats, Box-W1, CGTCA-motif, TATC-box, TGACG-motif, ARE, W box

### Expression of genes under water and salinity stresses

The presence of several putative *cis*-acting elements involved in abiotic stress response (Table 2) in proximal promoter regions of banana HDZIV genes as well as reported function of *Arabidopsis* HDG11 in drought stress prompted us to carry out expression profiling of banana genes under abiotic stress (Yu et al., 2008, 2013).

**Figure 7:**
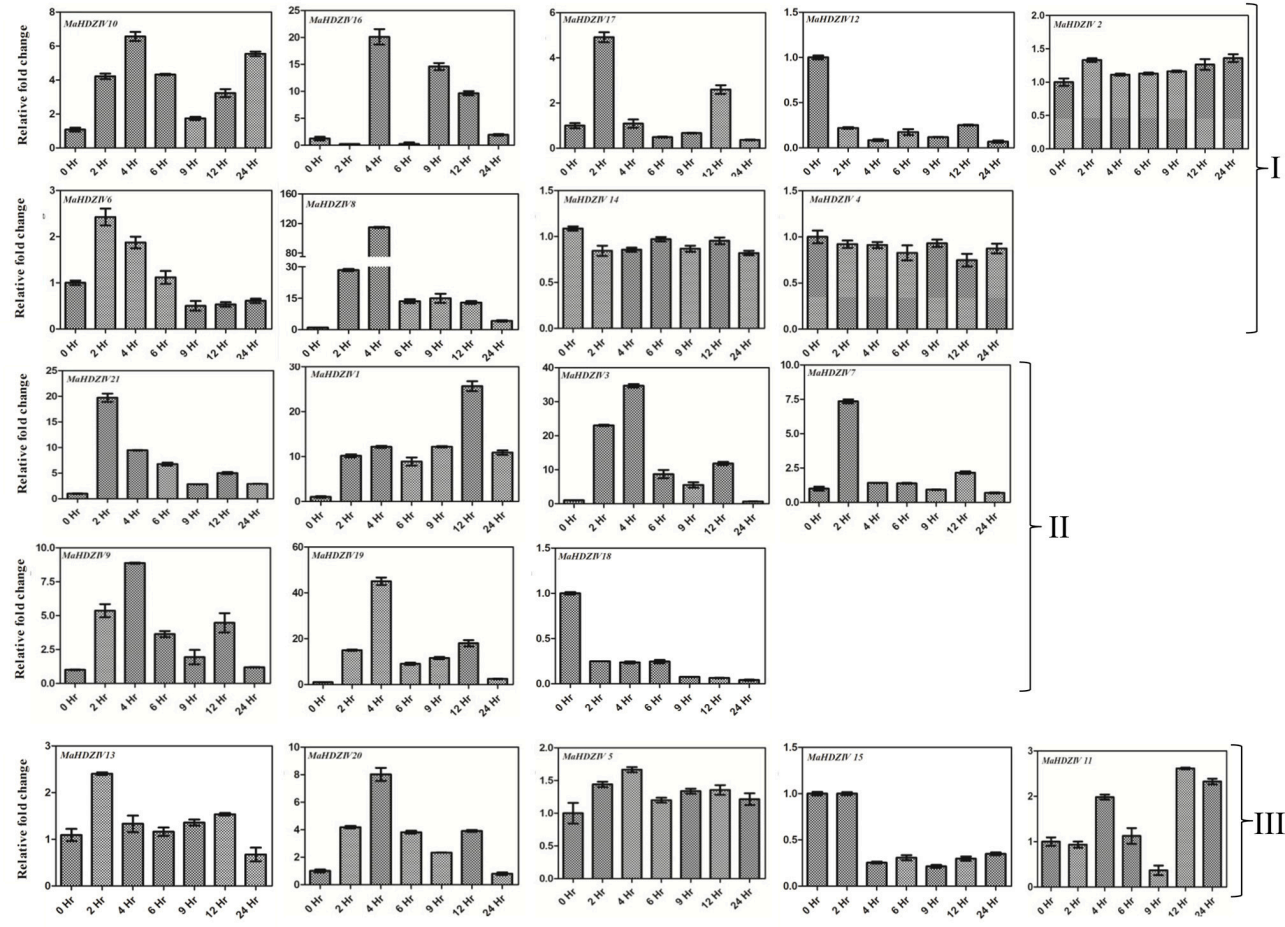
**Expression analysis of banana HDZIV genes following water stress treatment**. The water stress was imposed by sifting *in vitro* grown plantlet to the air. The samples were collected at different time points of stress imposition. The graphs are arranged as per the grouping in phylogentic tree.

Several banana *HDZIV* genes are differentially expressed at different time points of imposition of drought stress in banana (Figure 7). In most of the cases, prominent up-regulation was observed after 2 h of the stress. A set of genes including *MaHDZIV1, MaHDZIV8, MaHDZIV21, MAHDZIV* 9, *MaHDZIV3*, and *MaHDZIV20* genes maintained the up-regulated expression beyond the 2 h of the stress. Some of the genes, e.g., *MaHDZIV16, MaHDZIV17, MaHDZIV6*, and *MaHDZIV15* showed biphasic expression, i.e., up- and down-regulation at different time points. In addition, *MaHDZIV18* and *MaHDZIV12* were prominently down-regulated following drought stress.

**Figure 8:**
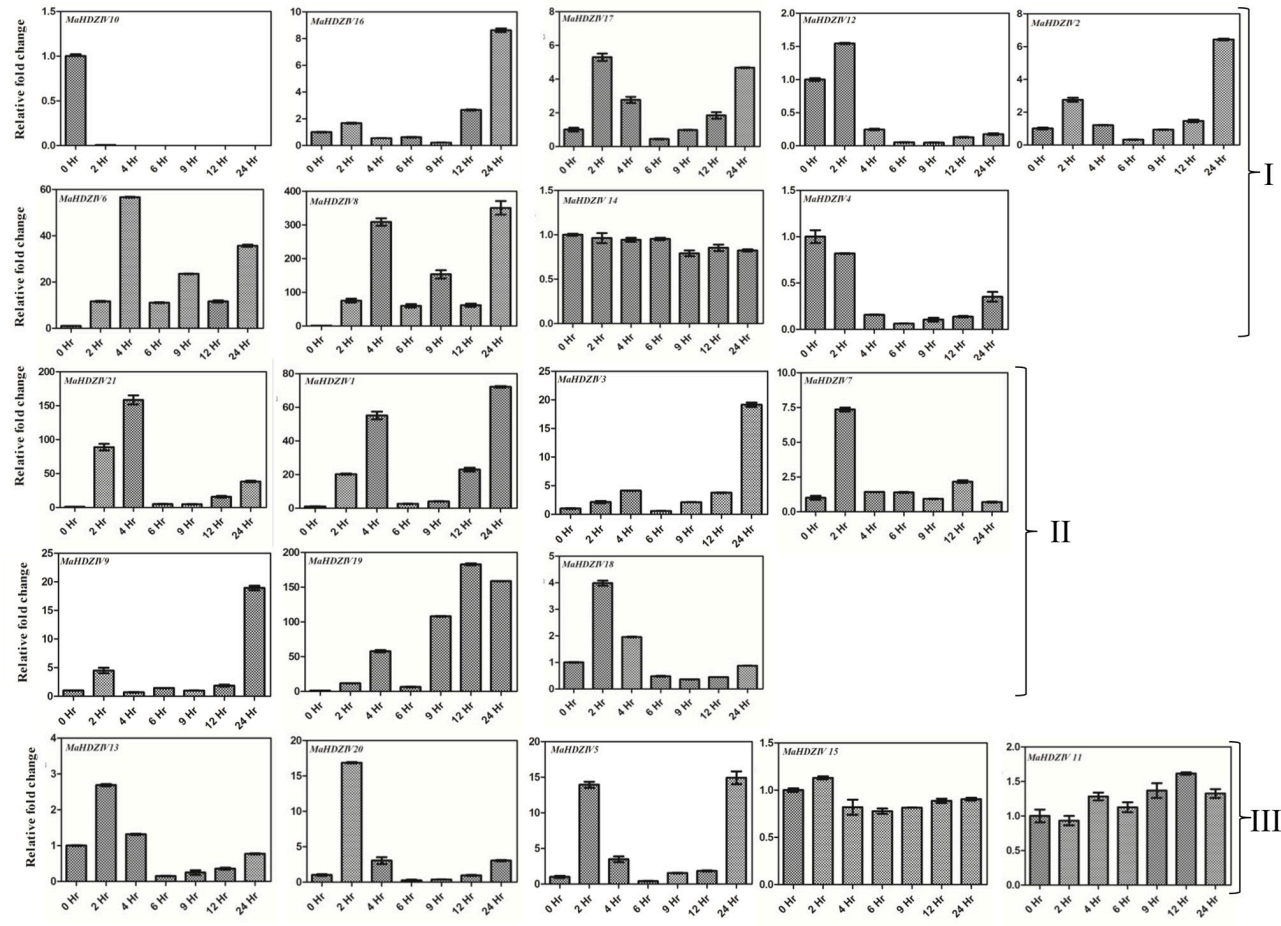
**Expression analysis of banana HDZIV genes following salinity stress treatment**. The salinity stress was imposed by sifting *in vitro* grown plantlet to a 250 mM solution of NaCl. The samples were collected at different time points of stress imposition. The graphs are arranged as per the grouping in phylogentic tree.

Salinity stress also modulated expression of several banana HDZIV genes (Figure 8). The transcript levels of a set of genes including *MaHDZIV16, MaHDZIV2, MaHDZIV17, MaHDZIV6, MaHDZIV8, MaHDZIV1, MaHDZIV3, MaHDZIV7, MaHDZIV11, MaHDZIV9, MaHDZIV19, MaHDZIV20, MaHDZIV5*, and *MaHDZIV21* were positively modulated at one or more time points of imposition of salinity stress. Furthermore, most of these genes displayed significant modulation in their expression just after 2 h of salinity stress imposition. As in case of water stress, several genes exhibited a mixed expression pattern. The *MaHDZIV10* gene exhibited down-regulation under salinity stress. Notably, among all these genes, the expression of *MaHDZIV1, MaHDZIV8, MaHDZIV2, MaHDZIV9, MaHDZIV3*, and *MaHDZIV20* genes was up-regulated in drought stress. Therefore, these genes seem to be under control of a common regulatory network involved in abiotic stress response.

## Discussion

The plant-specific HDZ sub family IV transcription factors have been primarily implicated in the developmental regulation of the epidermal structures such as stomata, trichomes, cuticle and root hairs (Ariel et al., 2007). As these structures play crucial roles in the gaseous exchange, water absorption and balance and in stress response, this gene family is considered as one of the important evolutionary mechanisms for the successful land life (Zalewski et al., 2013). Considering important regulatory role of these transcription factors, in the present work, we have carried out a comprehensive survey of this gene family in an important fruit crop, banana.

Our genome-wide analysis identified 21 HDZ subfamily IV genes in banana (Table 1). The numbers of HDZIV genes in banana is higher than those reported in case of other monocots, e.g., rice and maize (Javelle et al., 2011). Analysis also suggests that banana HDZIV genes encode proteins containing all conserved domains, characteristic of HDZ transcription factors (Figure 2). In total, analysis predicted 20 distinct conserved motifs among various banana HDZIV proteins. While motif composition within HD, LZ and START domains was almost conserved, variations were observed in SAD domain regions in different banana HDZIP proteins (Figure 2). The HD and LZ domains have been reported to be responsible for protein-DNA and protein-protein interaction, respectively (Elhiti and Stasolla, 2009). Precise regulatory roles of START and SAD domains are yet to be established (Ariel et al., 2007). However, based on the analysis of GL2 transcription factors of Arabidopsis, the START domain was shown to be required for transactivation of a set of genes (Schrick et al., 2014). Furthermore, there is evidence that like their animal counterparts, the START domain of plant HDZIV proteins could interact with lipid and steroid ligands (Schrick et al., 2014). The exact chemical nature of these ligands and mode of action toward modulating activities of HDZ proteins remain open questions for future research.

The HDZIV proteins were clustered in four distinct clades (Figure 1) in the phylogentic tree, which is in complete agreement with the phylogentic analysis in earlier report (Javelle et al., 2011). Within group/clades, HDZIV proteins shared similar motif composition as well as exon-intron architecture (Figure 3), further confirming their relatedness. In the monocot clusters, banana HDZIV proteins formed distinct groups. These results further support the fact that after evolving from a common monocot ancestor, banana and poales have followed distinct evolutionary history (D'Hont et al., 2012). Whole genome duplication (WGD) has significantly contributed in expansion and diversification of plant gene families (Flagel and Wendel, 2009). The banana lineage, after separation from the common ancestors of banana and poales might have experienced three whole genome duplication events, as opposed to the two in case of poales lineage. Comparatively more number of WGD events in banana lineage should explain the presence of more number of HDZ genes in its genome (D'Hont et al., 2012). Our gene duplication analysis further points toward the contribution of WGD in evolution of HDZ subfamily IV genes. Several closely related gene pairs were mapped on duplicated regions present at different linkage group suggesting that segmental duplication played a major role in evolution of banana HDZIV genes. One of the clades, clade IV, was devoid of any HDZIV gene from banana suggesting that this clade might be restricted to the poales lineage.

The phylogenetic analysis of the genes can be helpful in prediction of gene function in a given plant species. However, to reach such a conclusion, the tree must contain significant number of functionally characterized genes from different taxonomic groups. As compared to *Arabidopsis*, only limited number of HDZIV genes has been characterized in monocots. Also, the topology of the phylogentic tree indicated that some of the Arabidopsis, rice, and maize orthologs are missing in banana and that there are comparatively more numbers of paralogs in certain banana-specific groups (Figure 1). Taken together, absence of functionally characterized members of this gene family and a variable number in different clades makes it difficult to predict function of specific member. However, based on the homology and the expression profiling, analysis related to prediction of putative functions of some of the banana HDZIV genes has been attempted.

The drought tolerance in *AtHDG11* overexpressing lines was attributed to the improved root architecture and altered stomatal density (Yu et al., 2008). As of now, none of the monocot gene closely related to HDG11 has been functionally characterized. In the present work, certain banana genes phylogentically related to the *AtHDG11* (Group a of clade I) were identified to be up-regulated following drought stress suggesting that these could be functional orthologs of AtHDG11. The *ZmHDZIV4* gene of maize has been implicated in trichome and in regulation of some aspects of anther cell wall division and proliferation (Javelle et al., 2010). Provided that banana plant parts are devoid of trichomes, it will be interesting to elucidate functions of banana orthologs of *ZmHDZIV4*. In the clade II of the phylogentic tree, the dicot HDZIV genes, for example AtHDG1 and SlCD2 have been implicated in regulation of cuticle development (Wu et al., 2011). Most of the banana genes, closely related to these genes showed an up-regulation during progression from juvenile to mature leaves. In addition, their transcript levels were enhanced in the outer layer of the pseudostem. Therefore, we speculate that some of these could be involved in regulation of cuticle deposition. The closely related AtML1 and AtPDF2 transcription factors regulate epidermis development and floral organ identity in Arabidopsis (Kamata et al., 2013). The AtHDG2 and AtML1 both have been shown to be involved in differentiation of stomata in Arabidopsis (Peterson et al., 2013). There are multiple banana HDZIV genes in the clade containing AtHDG2, AtML1, and AtPDF2. It therefore remains to be established whether these banana HDZIV are functionally equivalent to their closest Arabidopsis homologs. Although several banana HDZIV genes with up-regulated expression in somatic embryos were identified, the transcript level of *MaHDZIV11* was significantly higher in this tissue as compared to other tissues (Figure 6). We hypothesized that this gene could be involved in regulation of some aspects of embryo development and differentiation.

The HDZIV gene family expresses primarily in epidermal cells and regulate differentiation aspects in this tissue domain (Chew et al., 2013). Apart from playing important role in the epidermis development, HPZIV members have been reported to play important role in several other developmental processes such as embryo development, root hair patterning and determination of floral identity (Wang et al., 2007; Kamata et al., 2013; Ogawa et al., 2015). Interestingly, tissue-specific expression analysis, carried out in this study, revealed significant expression of some of the banana HDZIV genes in pulp tissue. Moreover, expression of certain genes, e.g., *MaHDZIP14, MaHDZIP2, MaHDZIP8* was comparatively up-regulated in mature pulp suggesting that these might be involved in certain aspects of fruit ripening. AtHDG11 has been reported to up-regulate the expression of genes encoding cell wall loosening enzymes such as expansins, extensins, xyloglucan endotransglucosylase/hydrolases (XTHs), pectin-related enzymes, and cellulases in Arabidopsis roots (Xu et al., 2014). As fruit ripening is associated with a large scale transcriptional reprogramming of such genes (Kesari et al., 2007) it could be interesting to find out whether these banana HDZIV transcription factors target genes, encoding cell wall loosening enzymes in fruits.

Large numbers of banana HDZIV genes were reported to be up-regulated by drought and salinity stress. Various reports suggest that HDZIV transcription factors regulate cuticle deposition, root hair development and stomata differentiation (Takada and Iida, 2014). Since these structures are linked to the regulon of water stress response, the up-regulation of genes under abiotic stress might be a mechanism to modulate epidermal differentiation for minimizing water loss. Recently, HDG11 in Arabidopsis has been shown to regulate JA biosynthesis and potentiate JA–auxin cross-talk resulting in lateral root formation (Cai et al., 2015). We speculate that some of the banana homologs of HDG11 could have similar affect over phytohormone biosynthesis and signaling.

The transcriptional regulation is one of the most important mechanisms to regulate HDZIV expression (Chew et al., 2013). Several interesting *cis*-acting elements associated with plant growth, development, and stress response were identified in the proximal promoters of the banana HDZIP genes (Table 2). The light has been reported to have profound effect over diverse aspects of plant growth and development including differentiation (Fankhauser and Chory, 1997). Presence of several light responsive elements in promoter regions of all of the HDZIV genes suggest that light signaling might have modulatory effect over HDZIV gene expression. In addition, the presence of two conserved nucleotide motifs at 3′UTR in certain HDZIV genes points toward a mechanism for post-transcriptional gene regulation in HDZIP genes (Javelle et al., 2011).

The expression analysis in tissues as well as under different stresses suggests their possible role in development, fruit ripening, and stress response. Our expression analysis suggested that few genes, i.e., *MaHDZIV1, MaHDZIV8, MaHDZIV2, MaHDZIV9, MaHDZIV3*, and *MaHDZIV20* were commonly up-regulated by salinity and drought stresses. These genes can be used for the development of stress tolerant banana varieties using biotechnological tools. Future research involving development of banana lines with modified expression of these HDZIV genes should elucidate the function of individual gene family members. We believe that elucidation of functions of these genes should provide novel molecular tools to improve important agronomic traits in banana.

## Author contributions

AP and PT conceived the idea. AP, PM, AA, NK, SS, DL, ST carried out various experiments. AP, PT, PM, MA analyzed the data. AP, PM, and PT wrote the manuscript.

### Conflict of interest statement

The authors declare that the research was conducted in the absence of any commercial or financial relationships that could be construed as a potential conflict of interest.
