# Supplementary material for: Genome-Wide Identification and Expression Analysis of Homeodomain Leucine Zipper Subfamily IV (HDZ IV) Gene Family from Musa accuminata
Source: Front Plant Sci. 2016 Feb 1;7:20. doi: 10.3389/fpls.2016.00020 (PMC4740955; doi:10.3389/fpls.2016.00020)
Supplement: Supplementary file 1 [file Presentation1.PDF]

Supplementary information

Genome wide identification and expression analysis of Homeodomain leucine zipper subfamily IV (HDZ IV) gene family from *Musa accuminata*

Ashutosh Pandey[1,*], Prashant Misra[2,#], Anshu Alok[1], Navneet Kaur[1], Shivani Sharma[1], Deepika Lakhwani[2], Mehar Hasan Asif[2], Siddharth Tiwari[1], Prabodh Kumar Trivedi[2,*]

[1]National Agri-Food Biotechnology Institute (NABI), (Department of Biotechnology, Government of India), C-127, Industrial Area, Phase VIII, S.A.S. Nagar, Mohali 160071, India

[2]CSIR-National Botanical Research Institute, Council of Scientific and Industrial Research (CSIR-NBRI), Rana Pratap Marg, Lucknow-226001, INDIA

[#]Present address (PM): CSIR-Indian Institute of Integrative Medicine (IIIM), Canal Road, Jammu-180001, INDIA

*Authors for correspondence:

PKT: prabodht@nbri.res.in; prabodht@hotmail.com

AP: ashutosh_biotech@yahoo.co.in

Running title: HDZIV family in banana

Highlight:

We have identified a gene family encoding HDZ IV transcription factors in banana. Our analysis suggests that these genes could be involved in regulation of development and stress response.

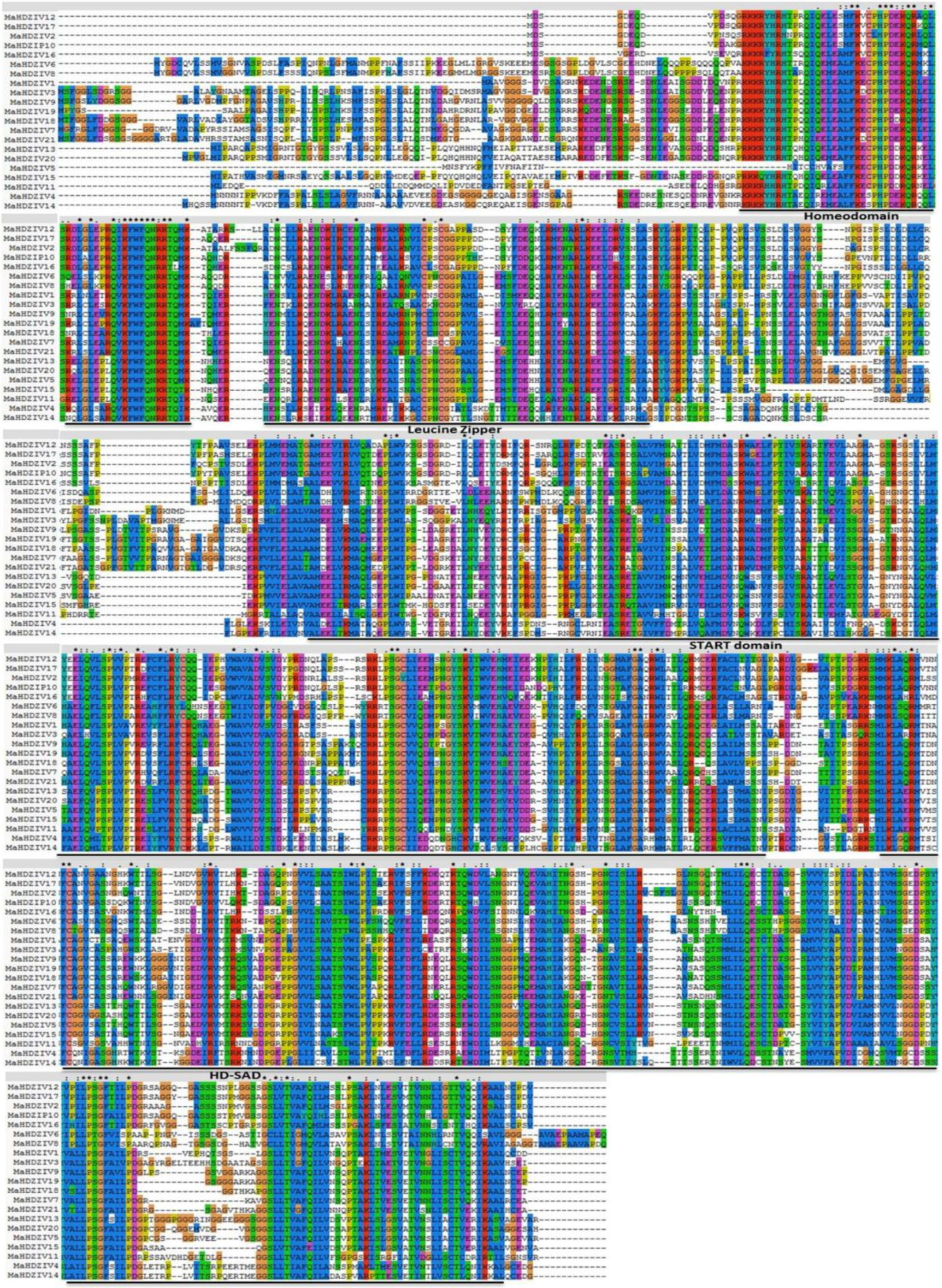

Supplementary Fig. S1. Multiple sequence alignment of banana HDZIV proteins. The alignment was done by using CLUSTAL X program. The conserved domains, namely homeodoamin, leucine zipper, START and SAD are marked. \

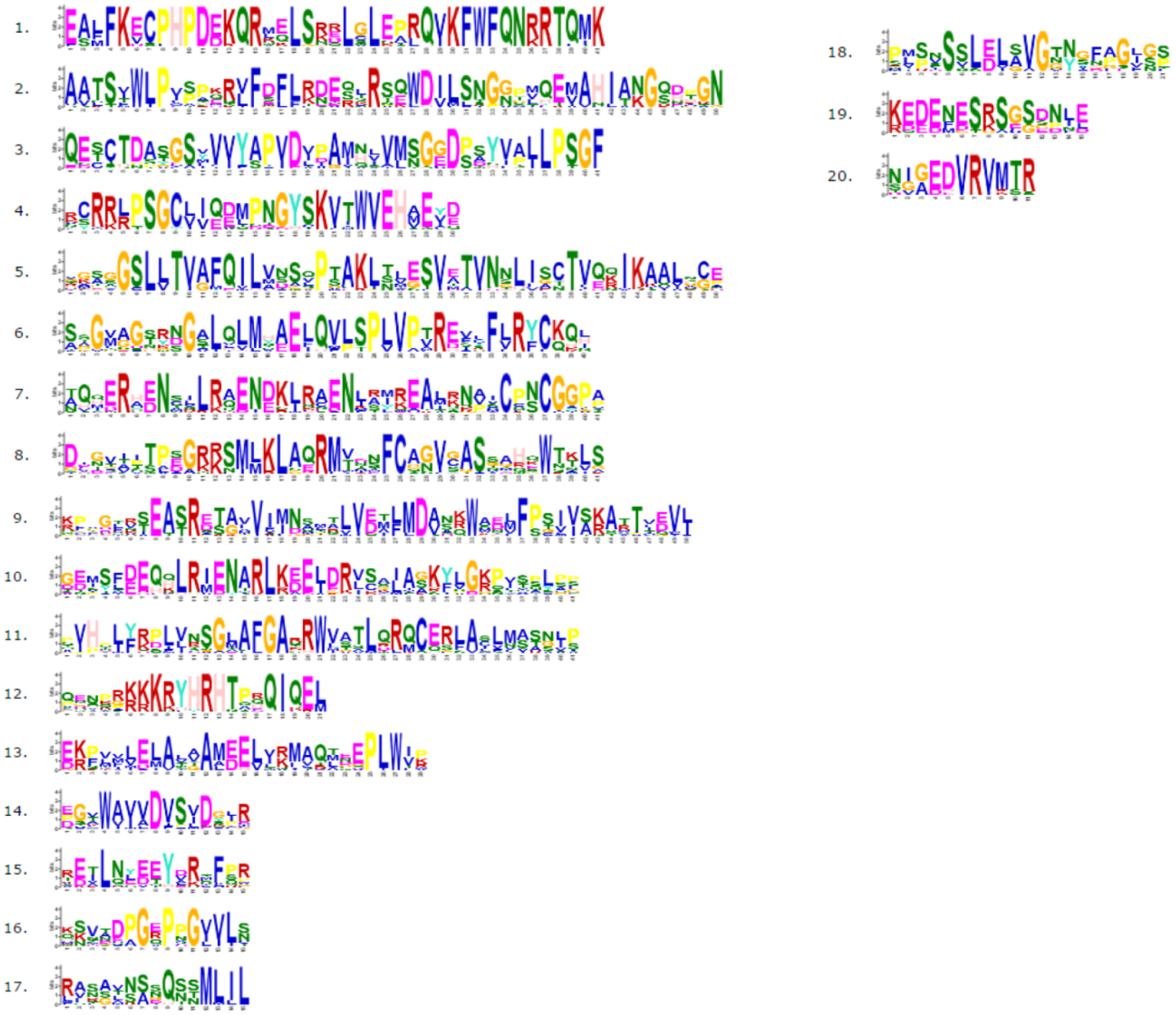

Supplementary Fig. S2. Sequence logos corresponding to the conserved motifs present over banana HDZIV proteins

Supplementary Table 1. List of primers used in qRT PCR

Primer name	Forward Sequence (5’ to 3’)	Reverse Sequence (5’ to 3’)
MaHDZIV1	CTCCGTGGACAGCATCAGAG	TGTTCCACCCAAGTCACCTT
MaHDZIV2	TCGGAGTCAGGGTTACAATTCA	TCCTTGAAGAAACCGAAGACCT
MaHDZIV3	CTAATGGATGCGAGTCGTTGG	CCGTCTCTTGTTCCACTCACG
MaHDZIV4	TGGACCAAGGTATCGACAAAGA	TGGACCAAGGTATCGACAAAGA
MaHDZIV5	AGAGGGTCTTCGATTTCCTACG	GAGACACAGTTCCCATGATCCT
MaHDZIV6	GTTATTCTCACTGCTGTCTCGAC	GTTACCGCTTGAGAGAACATCC
MaHDZIV7	TCCTTGTCTCCTCCTCCCTC	CAGAAGTTGTCGGTCATGCG
MaHDZIV8	TCTTGAACTTGGAAGAGCATGC	CGTTCATTATGACCATGGCACT
MaHDZIV9	GTTGACACTCTCATGGATGCTG	GTGCACCATTGTTAGTCCCAC
MaHDZIV10	GGTTGTGCTTCAGAAAACCTCA	GCTCGTCCTTAAACAAGCTGAA
MaHDZIV11	TGCGTAATACAACAACTGCCAA	AATGCTAGACAGGAGTTCACCA
MaHDZIV12	CGGTGAGGATCCATCTTACGTA	ATGGATTTGAGCTGGATGATGC
MaHDZIV13	TCTCTAATGGTGGTGTGGTTCA	CTGGTTGGAGTTTGTGCTGTT
MaHDZIV14	CGGAAGAAAGAGCATCCTGAAG	ACCTTTTGTTGACACCTTGGTC
MaHDZIV15	AAGTCTAATGGCAAGCAACGTT	AAAACTCGTCACCATCCTCTCA
MaHDZIV16	CTTCAACCAGCTCTTGTCCAAC	CACTGTGGCTATTGACTCGAAG
MaHDZIV17	GCAGCAACCTCTATATGGCTTC	GTACAGTGTTTCCGTTCGAGAG
MaHDZIV18	TGACGGTGGCCTTTCAGATC	CGCCTTTATCTTCTGAACGGTG
MaHDZIV19	CTCCTCCGGCTAAAATGACATG	GGTGAACTGTAGTCTCGTCGTA
MaHDZIV20	GCTCAGCTTCACATCAATGGAC	ATGTGGCCGCATTAAGAACAAT
MaHDZIV21	TTGGCTATGCTCATGTCCTCTT	CGCAGAAGTTGTCCGTCATG
MaActin	ATGACATGGAGAAGATCTGGCATCA	AGCCTGGATGGCAACATACATAGC

Supplementary File S1. Genomic regions and coding sequences corresponding to the predicted *HDZIV* genes from banana. The 5' upstream region, exons, introns and predicted 3'UTR are represented in blue, red, black and green colours, respectively.

GSMUA_Achr5P20050_001 (*MaHDZIV9*)

ATCACAAGTCGGCTTTTAAAATTGATGAATAATAATGAATATAAACTCAACTTAGAAACATAGGTTTCATGAATT
ATGGATCATTGCTAATATGTATCAGCTGACTCTTTCAGTCTCTAAGAACTTGAGTGTCAAATTCAACGTATAAGT
TCGCGTTTAATAAATGATGAATTAGAGTTAATAAATACCATTTCTTTTTTTTGCTTTATAATATTGAATGCAACT
TTAACATAAAAATTAGGTTTAATAAATTACATAATACATTTAATTTATATTTTTATTATATTAAATGGTATACAT
GTCTTGCTCACATATAGATAACAAGTTGTGTAACAATACACTAATATAAATATAAAAGTTAATATACAATAAATG
ATAAATCAAATTTAGTACATATCATCTAACTTTTTAAATTCTTAATAATATGATACTTTTTATTTTATATATTAA
TATTATAATTTATTTTTGTATCAAGTAAAAAAAATTAGATATAAGTACATAAATATTATATGTTAAAATTGTATA
TATGAATATTCTAATTATACTTTAATTTTCGCACAGAAATATGCATTTTTTTATTTTGATATCTCTGATTCAAAT
CGACAACTTAATATTTTGGTGGAAATTTTCTCCTTTTTGTTTTACAATGAGGAAAATTTCCTTCTTGAAGGCGAT
ACGGTGTGGACTTTAGCTTTAGCTTTTACGAGAAAGACGGACGGGACAGTCAACCAACCCTGTTCTTCTTTTTTT
TTTTGGCCAACCGACCCTGTTCTTTCTCTCTGTACTGAGCTCTCCCTTTCCCGATCTTTCTAAAAAGATCACCTT
TTTGTTTGATTTCTGCAGCCTCGAAGCGGCCGCGTATAAAGATCTTCCCCCCTGCCTTCGCCTCGCCCACTGTAC
TCCACCTCCCACGGTCTCTGAGCTTCTAAAGGTTCGATCTTTGTGCTTCGCTACTAAAAAGAGTTCAGAGCCATG
CAACAGAAGTAGAGAGAAGGGGGGAGGAAAGCTCGGGCTCTGTGGCTTCCCTTTCTCTTGCTTTTTATATGTTGG
AGGGGGTACGCTGGGGTTATGGGGTTCGAGAGGTTAGGCGAGGAACAGGGAGCGGGACAGAGAACGAGGCGCTAG
CGTTTCTTGCTTGGCTTACGTCTCTGTCTTTCTCCCTTGACTTTGGGACAGGGGGAGAGGGTGTTGAAGGCATTT
GTTCGTCCCACAGCTGATGCTTTTGCCTCCTCTCGCCTCTGCTTCAGATCTCTCCCTTCTGCTCTTTGCTTTCTT
GGTGTGTTTTCCCCGTTTTTCTCTTCCGTTTCTTTCATTTTTCGTTAGATTTCGGTTCGATCCCCTCGAGGTGCT
GCTGCATGTCCTTGCTGAATCGAGAAGAATCGTGTGGGAGGCGAGTGCACTCGGTAGATCTGCCACTGCCGTCAC
TGTTACTCGGAGGAAAAGAAGGCGTCTTCGAAGGAGAACGAAGCGACGTTGAGAAGAACAACTAAGGGATCTTTG
ATGAGTTTTGGGAGTTTGTACGACGGCGGCTCTGGCGGCGGCGCTCGTCTCGTAGGCGACATGCCGTTCGGCAAC
CCTGCCGGCGCCGTCTCCCACCCGCGCCTTCTTTCGTCCTCGCTCCACAAGTCCATGTTCAGCTCTCCTGGTCTC
TCCCTCGCCCTGGTAACCCACCATTTCTTCTTTCCTTCTCCCACACGTTCTTGGAGCGGTTTTGGTATACTTGTT
TGGGTTACTTTCCGCAGCAAACGAATTTGGACGCGCACGGAGTTCGGAACTTGGCCTCGGTGGTTGGCGGCGGTG
GAGGGCAGCTGGATTCTGCTCGTCGGAGCAAGGAAGATGAGAACGGGAGCAGATCGGGGAGCGATAACTTGGAAG
GCGGATCTGGAGACGATTTGGAGCAAGAAAACCCTCGGAAGAAGAAGAGATACCACCGCCACACCCCTCAGCAGA
TCCAGGAACTGGAAGCGTAAGGGATCCATTTGCGTCGAGCTCGTACCTTTTGATTCTACTCGCATCCCCCGAAGG
AAAAGTACATTTTGACCCCCTGTTTCTAGTCTTTTCAAGGAGTGCCCTCACCCCGACGAGAAGCAAAGGATGGAG
CTCAGCAACCGGCTTTGCTTGGAGGTTCGCCAGGTCAAGTTTTGGTTCCAGAACAGACGAACGCAGATGAAGGTG
TTTGTTCTTCCTGAGGCCATACCTCCCCTAAAGGTTCGATCTTTATCTCTCACGTGCTTGCAAATCGTGGAAACC
AGACCCAGATGGAACGGCACGAGAACATGATCCTGAGGCAAGAGAACGACAAGCTTCGAGCGGAGAACCTATCCA
TCAGAGAGGCCATGAGGAACCCCATGTGCTGCAACTGTGGTGGCCCGGCGGTACTCAGCGAGATCTCCCTCGAGG
AGCAGCACCTGAGAATGGATAACGCTCGCCTCAAAGACGACCTCGACCGCGTCCGCGCCCTCGCCGGGAAATTTC
TCGGAAAGCCCGTCTCCGCCTTGGCCGGCTCGCTCCCTCTCCCGTTGCCGAACTCGTCGCTGGAGCTCGCGGTCG
GAACGAATGGTTTTGCCTCCGTCGGCACGGTGGCCGCCGCGACGTTGCCTCCGCTGACTGATCTCCCTTCTGGGG
CATCAAGCCCTCTGGACACCGTCATAACTCCCTCGCGGGCCGTAGGCGGCGTGGACAAATCGCCGGACAGGTTCG
TGTTCTTGGAGCTTGCACTCGTAGCCATGGACGAGTTGGTGAAGATGGCCCAGTTGGAGGAGCCCCTGTGGATTC
CGAGCTTGGACGCCGGCAGAGAAACCCTGAACCACGTCGAATACGACCGGTGCTTCTCCCGGTGCATCGGCCCCA
GGCCCACCGGCTTCGTTTCCGAGGCCACGAGGGAGACCGGGGTGGTCATCATCAACAGCTCTTCCCTTGTTGACA
CTCTCATGGATGCTGTAAGCGTTTATCTATTTGCGTTACAGGCACTGTATTGGAATCCATCAAGGTTCTTATGAA
TTCACGTCTGATTCATGAGCAGGCTCGATGGGCAGACATGTTCCCGTCTGTGATTGCAAGAGCGAGCCCTGCGGA
CGTGATCTCCAGCGGCCTGGGTGGGACTAACAATGGTGCACTCCAGCTTGTGAGTCCAAGCATCACTCGTGAGCT
ATTGATTCATTCATCTATATCGAATCTGATGCCTGTTGCTTTCGGTGCCACCAGATGCATGCGGAGCTCCAAGTT
CTGTCTCCCTTGGTTCCTGTTCGAGAAGTCCGTTTCCTTAGGTTCTGCAAGCAGTTGACTGAGGGCGCTTGGGCC
ATAGTTGACGTCTCCATAGACGGAATTAGAGGCACCCCATCTGCCTCACCGGCTAAGACGCAATGCCGCAGGCTT
CCTTCCGGATGTGTGGTGCAAGATACGCCTACTGGCTATTCTAAGGTAATTGATCACTGTCCTTCCCCACTTCGT

TCCGTTTCGTGCATTTCCATTGGAGTTGCACGTCTCCCCACGCTGACTTCTCGTGCGTCTTTCTTTAGAGGTTTA
GATGCTTCTTCTTCTCCATCGAGAGCGTCACCCTAAGATTGCCTGCTGTCTTGTTTATGCAACCTATCTCGCCTC
TCTCTCCGGGCTCTCAAAGCCCTAAGTTGGGGCTCCGCCGCCGGGTTCGCCCATGTCTTTCGATCACAGTTTGTA
TTGTTTAGTCTGGACATGGAAGTCTCTTCTCTTCCTGTCCCCGTCTCCTGCAAATAAACATTAAAAATGTCGCGT
CCCTCGTGAGCTGTGCGTCGCTTTCTTCGAACATGGATTACGATGACTTAGTTTACATCTAGATTGGTTTAGTCG
CAACGAGTCTGCTGTTTCTAATCCCATTTCTATTCCAGGTCTTAGCTTTAATTAGTCATCTAAGCTAACACCCGT
GCAATAGCTCGAGGCCTATTCTTAAGAGGGTTCAGTAGTATATACTGCTTCTGTTCATGTGTTTTGGATCGATGC
AGTCACTGGTTCTCATAGTCAAAGCTTTTGGCGTCCATCAGAGCCCAAGTCGTGTGCAGTATCAAGCTTGCATAG
GGAAGAGTGAACTGTGTCTCCTAGTGACAGCGTGCACTGTAGCGTCGACCTAGCAGAGAGAGAGAGAGAGAAGGG
AGATTCAATGAACTAACGACGACGGTGGTGCGCAGGTCACATGGGTGGAGCATGCCGAGTACGACGAGGCGGCAG
TCCCGCCGCTGTACCGCCCGCTGCTGCTCTCCGGGTTGGCCCTCGGTGCCCGCCGCTGGGTGGCCTCGCTCCAAC
GCCAGTGCCAGTCCCTCGCCATCCTCATGTCCTCCTCCCTCCCTCCTGATGACAACACCGGTAAGCTTCTTGGCG
AAGAACGAACACGTATAAGTGCAATAGTTGTTTACAGCAGAAGTGGTCGTATTGCCATGACAGCGATAACGCCGA
GCGGACGGAGGAGCATGCTGAAGCTGGCGCAGCGGATGACCGACAACTTCTGCGCCGGCGTCTGCGCATCGTCGG
CCCGCGAGTGGAAAAAGCTTGGCGGCGGGATCAACATCGGGGAGGACGTGCGGGTGATGACGAGGCAGAGCGTGG
CCGACCCGGGGGAACCGCCGGGGGTGGTGCTCAGCGCCGCCACCTCGGTGTGGCTCCCAGTTTCACCGCAGCGCC
TGTTCGACTTCCTCCGCAACGAGCAGCTCCGCAGCCAGTGGGACATCCTCTCCAACGGTGGGCCCATGCAGGAGA
TGGCCCACATCGCTAAGGGCCAGAACACCGGCAACGCCGTCTCCCTCCTCCGAGCCAGCGTGAGTATCGTTTTAC
CTCACTTCCATAGCTCTTCCTTCTCCTCAGCGTACAAACGTACTAGTGACATTAGACTTGACATGAGAAGCATGA
CTGGCTCATAGCTGTCCTTTCTTCTTCACCCCTGACATCATAGTAACCTTCCCTCATGCTCAATCCATACTTTTA
TCATTATGTGGTTGCATTGCTGTTTCTTGAAGCAGTGTGGCGCCTCAGCATTTGAGTTGTATCTGCAGGCCATGA
ACGCGAATCAGAGCAGCATGCTGATACTGCAGGAGACGTGCACGGACACGTCGGGGTCGCTGGTGGTGTACGCGC
CGGTGGACATCCCGGCCATGCACCTCGTCATGAGCGGCGGTGACTCCGCCTACGTCGCTCTCCTTCCCTCCGGCT
TCGCTGTCCTCCCGGACGGCCTACCCAGCGGCAGCGTCGGTGGCGCGCGCAAGGCCGGTGGCTCTCTGCTGACCG
TGGCGTTCCAGATCCTAGTGAACAGCCAGCCAACAGCGAAGCTGACGGTGGAGTCGGTGGAGACCGTCAACAACC
TAATCTCCTGCACCGTCCAAAAGATAAAGGCGGCGCTCAACTGCGAGCCT**TGA**CGAAGCAGCAGTGTAGGGGAGA
GTGCCATGGCATGGGTTGAGTCAAGAACGAACCGCGCGAACAAAGATCCCTAGCACCGAGCGATGGTGGTGGTTC
GGGCATTGACTCTGTCTCTAAATGCCGTCGCTCCACCGCCTGCTGCGTTATTCAATTGATGTTGTCGTGTGGCTT
TTGTTTCGTGTGTGGAAGACTATGTTGTACGCTTGCTTTACGAGTATTGGGTTTCATCTAAGAACGAGATGGTTG
AGTTGTAGCAAGTTCAATAAATGTTGGTGATGGTAAGGAAGTCATACTCTTCTTCTTCCTCCTTGATTCATTTCC
ATGACTATGTGACATGCATCTCACACAGGCCATGCTACATATTTGATCTAAGAATTCTATGGTATACATGAAAAA
CACAATCTGTAGTCTACAATTTAGGATAAATAAACTGCTGTATTTAGATGTGTCTATCTACTTGATAAGAGTTTT
AAATTAAATTCAAACTTTTTGATGTATT

CDS

ATGAGTTTTGGGAGTTTGTACGACGGCGGCTCTGGCGGCGGCGCTCGTCTCGTAGGCGACATGCCGTTCGGCAAC
CCTGCCGGCGCCGTCTCCCACCCGCGCCTTCTTTCGTCCTCGCTCCACAAGTCCATGTTCAGCTCTCCTGGTCTC
TCCCTCGCCCTGCAAACGAATTTGGACGCGCACGGAGTTCGGAACTTGGCCTCGGTGGTTGGCGGCGGTGGAGGG
CAGCTGGATTCTGCTCGTCGGAGCAAGGAAGATGAGAACGGGAGCAGATCGGGGAGCGATAACTTGGAAGGCGGA
TCTGGAGACGATTTGGAGCAAGAAAACCCTCGGAAGAAGAAGAGATACCACCGCCACACCCCTCAGCAGATCCAG
GAACTGGAAGCTCTTTTCAAGGAGTGCCCTCACCCCGACGAGAAGCAAAGGATGGAGCTCAGCAACCGGCTTTGC
TTGGAGGTTCGCCAGGTCAAGTTTTGGTTCCAGAACAGACGAACGCAGATGAAGACCCAGATGGAACGGCACGAG
AACATGATCCTGAGGCAAGAGAACGACAAGCTTCGAGCGGAGAACCTATCCATCAGAGAGGCCATGAGGAACCCC
ATGTGCTGCAACTGTGGTGGCCCGGCGGTACTCAGCGAGATCTCCCTCGAGGAGCAGCACCTGAGAATGGATAAC
GCTCGCCTCAAAGACGACCTCGACCGCGTCCGCGCCCTCGCCGGGAAATTTCTCGGAAAGCCCGTCTCCGCCTTG
GCCGGCTCGCTCCCTCTCCCGTTGCCGAACTCGTCGCTGGAGCTCGCGGTCGGAACGAATGGTTTTGCCTCCGTC
GGCACGGTGGCCGCCGCGACGTTGCCTCCGCTGACTGATCTCCCTTCTGGGGCATCAAGCCCTCTGGACACCGTC
ATAACTCCCTCGCGGGCCGTAGGCGGCGTGGACAAATCGCCGGACAGGTTCGTGTTCTTGGAGCTTGCACTCGTA
GCCATGGACGAGTTGGTGAAGATGGCCCAGTTGGAGGAGCCCCTGTGGATTCCGAGCTTGGACGCCGGCAGAGAA
ACCCTGAACCACGTCGAATACGACCGGTGCTTCTCCCGGTGCATCGGCCCCAGGCCCACCGGCTTCGTTTCCGAG
GCCACGAGGGAGACCGGGGTGGTCATCATCAACAGCTCTTCCCTTGTTGACACTCTCATGGATGCTGCTCGATGG
GCAGACATGTTCCCGTCTGTGATTGCAAGAGCGAGCCCTGCGGACGTGATCTCCAGCGGCCTGGGTGGGACTAAC

AATGGTGCACTCCAGCTTATGCATGCGGAGCTCCAAGTTCTGTCTCCCTTGGTTCCTGTTCGAGAAGTCCGTTTC
CTTAGGTTCTGCAAGCAGTTGACTGAGGGCGCTTGGGCCATAGTTGACGTCTCCATAGACGGAATTAGAGGCACC
CCATCTGCCTCACCGGCTAAGACGCAATGCCGCAGGCTTCCTTCCGGATGTGTGGTGCAAGATACGCCTACTGGC
TATTCTAAGGTCACATGGGTGGAGCATGCCGAGTACGACGAGGCGGCAGTCCCGCCGCTGTACCGCCCGCTGCTG
CTCTCCGGGTTGGCCCTCGGTGCCCGCCGCTGGGTGGCCTCGCTCCAACGCCAGTGCCAGTCCCTCGCCATCCTC
ATGTCCTCCTCCCTCCCTCCTGATGACAACACCGCGATAACGCCGAGCGGACGGAGGAGCATGCTGAAGCTGGCG
CAGCGGATGACCGACAACTTCTGCGCCGGCGTCTGCGCATCGTCGGCCCGCGAGTGGAAAAAGCTTGGCGGCGGG
ATCAACATCGGGGAGGACGTGCGGGTGATGACGAGGCAGAGCGTGGCCGACCCGGGGGAACCGCCGGGGGTGGTG
CTCAGCGCCGCCACCTCGGTGTGGCTCCCAGTTTCACCGCAGCGCCTGTTCGACTTCCTCCGCAACGAGCAGCTC
CGCAGCCAGTGGGACATCCTCTCCAACGGTGGGCCCATGCAGGAGATGGCCCACATCGCTAAGGGCCAGAACACC
GGCAACGCCGTCTCCCTCCTCCGAGCCAGCGCCATGAACGCGAATCAGAGCAGCATGCTGATACTGCAGGAGACG
TGCACGGACACGTCGGGGTCGCTGGTGGTGTACGCGCCGGTGGACATCCCGGCCATGCACCTCGTCATGAGCGGC
GGTGACTCCGCCTACGTCGCTCTCCTTCCCTCCGGCTTCGCTGTCCTCCCGGACGGCCTACCCAGCGGCAGCGTC
GGTGGCGCGCGCAAGGCCGGTGGCTCTCTGCTGACCGTGGCGTTCCAGATCCTAGTGAACAGCCAGCCAACAGCG
AAGCTGACGGTGGAGTCGGTGGAGACCGTCAACAACCTAATCTCCTGCACCGTCCAAAAGATAAAGGCGGCGCTC
AACTGCGAGCCTTGA

GSMUA_Achr3P23090_001 (*MaHDZIV5*)

ATATATATATATATATCATCCATTTTCCCCCTCATTTTTTAAATGTATTTTTAACAATTTCTTGACTATATTATA
CTGAAAATTAAACTCATATACATATATGAATAGTTTTTATAAACCATTTTTCATTGTTTTTAATGCTTTTATAAC
AAGTCCTTGACATATCCCACTAATAGTTAACCTCTCTCTCTCTCTCTCTCTCTCTCAGACACACACATATTGA
TATATGATATCCAGGAACAAATATAAATAAATAAATCCTAGGTGTTCTTTTTCATGGAATAACACAAAAGACCTT
TTTGTTTCTTTTGTTGAATTGCTTATATTATTTTTTCTTAAAAAAATACTATATTATTGATCCTATGATATATGT
ATATACACCAATTCTTGACATATTACACTGATTTATGAATACATATATAGGTAGCTTTCTTCTTAGATCTATTAA
TGGATAACAGATCATATTACATGCTGCCATGTTGCTTTCAGTTTCTTCAAGGAATGCCCTCACCCTGATGACAAG
CAGAGGAAGGAGCTCAGCAGAGAGCTCGGCCTCGAACCTCTCCAAGTCAAGTTCTGGTTCCAGAACAAACGCACC
CAGATGAAGGTACACCTCCATGTCTCCAAGAACTGCCACCGTGATACCTTTGACCTTCTCATCGACAACCCCATC
ATGATCCACTAATCTCTGGTTTCCGAGTTGTTTCATGGGTGCAGAACCAGCACGAGCGGCAAGAGAACAACCAGC
TGCGAGCCGATAACGAGAGACTCCGTGCTGAGAACTTACGGTATAAGGAGGCCCTCAGCAACGCCTCGTGCCCCA
ACTGCGGCGGGCCTGCCTCCCTCGGCGAGATGTCCTTCGACGAACACCACCTCAGGATCGAGAACGCTCGGCTTA
GAGAAGAAGTAATCTTCCTTCATTTTCACCACAAGGCTTCGATTGGCGGCTGACAGGGCTAATTGCACCCTTCCT
TCCTTGACAGATCGATAGGATATCAGGGATCGCCGCCAAGTACGTCGGCAAGCCGATGGCGTCGTACCCACTGCT
CTCGCCTTCCGTCCCTTCCCGCCCGCCGTTGGATCTCGGCGTCGGAGGCTTCGGCGGCCAGCAGGGCGTCGGGGG
GGAGATGTTCGGGCCGGGAGAGCTGATGAGGAGCGTGTCGGGTGCGGCCGAGACGGACAAACCGATGGTCGTCGA
GCTTGCGGTGGCCGCCATGGAGGAGCTCATCCGGATGGCGCAGCTGAACGAGCCGCTGTGGATACCCGCTGCGCT
TGACAACGCCACCGAGGCCCTCAACGAGGAGGAGTACGTCAGGACGTTCCCGAGAGGGATCGGTCCACGGCCGTT
CGGGCTCAAGTCGGAGGCATCGCGGGAGACGGCGGTGGTGATCATGAACCAGATGAACGTGGTGGAGATCCTCAT
GGATGTGGTACGTAAAGTCGCAGTCTTAGCAGGTGTTTCAGAGAAGAAGGCTCGATCGGATCATACATGCGCCTA
ATTCTCGTCTTCCACAGAATCAGTGGTCGAATGTGTTCTCGGGCATCGTGTCGAGGGCCATGACGCTCGAAGTGC
TCTCAACTGGAGTGGCAGGCAACTACAATGGGGCTCTGCAAGTGGTATGGCTTCTTCCCTGCCCTGGAAAAACTA
CATCTCCATGAAGGGGGGATTGTTATTATTTACTTCATGGAATCCTCAGATGACAGCAGAGTTCCAAGTGCCATC
TCCACTCGTCCCAACTCGAGAGAGCTTGTTCGTGAGGTACTGCAAGCAGCACGCTGATGGAACCTGGGCAGTGGT
TGACGTTTCCCTCGACAGCTTGCGCCCCAGCCCAGTCCTGAGATGTCGAAGAAGGCCATCCGGTTGCCTGATCCA
AGAGTTGCCTAATGGTTACTCGAAGGTGAAGAATCTAGTCCAGTAAAAGTGGATTCCACCCCAGAATGCATCTGT
CTGTAACATGTTCAACTTGCTTCTTCTATCGTGCCGCAGGTTACTTGGGTGGAGCACGTCGAAGTGGATGACAGG
TCTGTTCACAGCATCTACAAGCCATTGGTCAACTCCGGCCTGGCGTTCGGTGCAAGGAGGTGGGTCAGCACCTTG
GACAGGCAATGCGAGCGGCTCGCGAGTGTGATGGCCAGCAACATACCCTCAGGAGACGTCGGTGGTAAGCCAGCC
ATCTGTTGACATGATCATCGACAAGCTTGAGGTAGATTGAACTCTCTGTTCTTTGCGCAGTGATAACTACACCGG
AGGGCAGGAAGAGCATGTTGAAGCTGGCTGAGAGGATGGTGATGAGCTTCTGCGGCGGTGTCAGTGCTTCGACCA
CACATCAATGGACTACTTTGTCTGGAAGTGGTGCGGAGGACGTGAGGGTTATGACGAGGAAGAGCGTGGATGATC
CCGGAAGGCCTCCTGGTATCGTCCTCAACGCCGCCACCTCCTTCTGGCTTCCGGTCCCGCCGAAGAGGGTCTTCG
ATTTCCTACGTGATGAGAGCTCGCGCAGTGAGGTACTCGAACTTCAGCGTGGTTCTCTTGGAATTGCTGCAGTTG
CAGTGAGTTCGTCACTCACATGGTGCTGTTGGTGCAATTGCAGTGGGATATCCTGTCGAATGGGGGTGGCGTTCA
AGAGATGGCTCACATCGCCAATGGCCAGGATCATGGGAACTGTGTCTCCTTGCTGCGCGTCAACGTAAGCATCAT
CTGCATCTTTTGCCGGCCTCTGATCACTGTGGTTGACTCGATGGTGGTTTGCAGAGCACGAACTCGAACCAAAGC
AACATGCTGATACTGCAGGAGAGCTGCACCGACGCGACGGGCTCGTACGTGATCTACGCGCCGGTGGACGTCATC
GCCATGAACGTGGTGCTCAACGGCGGCGACCCCGACTACGTCGCCCTCCTGCCGTCAGGGTTCGCCATTCTTCCC
GACGGGCCATGCGGCAGCGGAGGAAGGGTGGAGGAAGTAGGCTCCGGTGGCTCCCTGTTGACTGTGGCGTTCCAG

ATCCTGGTCGACTCAGTCCCCACTGCCAAGCTCTCCCTCGGATCGGTCGCGACGGTGAACAGCCTCATTGCTTGC
ACCGTCGAACGGATCAAGGCTTCAGTTGCAGGCGAAGTGGCTCGCTGAGAATAGCAAGCACGCGGCATGGATAGG
TAAGAAAGAACCCAAGCTTACTCATCTACCTCCTCCTCGACCTACTTGGTAATCTGCGACTCGCTACCATGAGCA
GGAGGAGAGGAAGTTGGGGGAGGGTGGAGATTAGGACATCATCTGTGGGAAGTCAAGAACGCACCTCACACTAAC
CTTGGCTCTCGGCCACCGCCGCCGCCATGCAAGGAGGGCTGGTTCGGGTATTGACTTCCCCCCATTTCATGACTA
CAACGACAGTTCTCTCGCTCTCTCTCTCTCTCTCTCTCTCCTATCGATCCTTTTGTGTTGCTGGCCTTGAAGTCT
TCGTTGAAGTGTTCGTTTTGAGCTTTGGTTTAGCTTTGGTGTTCCTTGCTTTCAGAATCTTGTTTGCTTTGGAAC
CTGCATTGGTGTGAAGGATAGTATTGAAGACATGCTTTGGAACCTGCTACATCTTAGTCCCTACTTGAAGCCATG
AGTGTTTTACTCTTTATGATGCA
CDS
ATGAATAGTTTTTATAAACCATTTTTCATTGTTTTTAATGCTTTTATAACAAATCATATTACATGCTGCCATGTT
GCTTTCAGTTTCTTCAAGGAATGCCCTCACCCTGATGACAAGCAGAGGAAGGAGCTCAGCAGAGAGCTCGGCCTC
GAACCTCTCCAAGTCAAGTTCTGGTTCCAGAACAAACGCACCCAGATGAAGAACCAGCACGAGCGGCAAGAGAAC
AACCAGCTGCGAGCCGATAACGAGAGACTCCGTGCTGAGAACTTACGGTATAAGGAGGCCCTCAGCAACGCCTCG
TGCCCCAACTGCGGCGGGCCTGCCTCCCTCGGCGAGATGTCCTTCGACGAACACCACCTCAGGATCGAGAACGCT
CGGCTTAGAGAAGAAATCGATAGGATATCAGGGATCGCCGCCAAGTACGTCGGCAAGCCGATGGCGTCGTACCCA
CTGCTCTCGCCTTCCGTCCCTTCCCGCCCGCCGTTGGATCTCGGCGTCGGAGGCTTCGGCGGCCAGCAGGGCGTC
GGGGGGGAGATGTTCGGGCCGGGAGAGCTGATGAGGAGCGTGTCGGGTGCGGCCGAGACGGACAAACCGATGGTC
GTCGAGCTTGCGGTGGCCGCCATGGAGGAGCTCATCCGGATGGCGCAGCTGAACGAGCCGCTGTGGATACCCGCT
GCGCTTGACAACGCCACCGAGGCCCTCAACGAGGAGGAGTACGTCAGGACGTTCCCGAGAGGGATCGGTCCACGG
CCGTTCGGGCTCAAGTCGGAGGCATCGCGGGAGACGGCGGTGGTGATCATGAACCAGATGAACGTGGTGGAGATC
CTCATGGATGTGAATCAGTGGTCGAATGTGTTCTCGGGCATCGTGTCGAGGGCCATGACGCTCGAAGTGCTCTCA
ACTGGAGTGGCAGGCAACTACAATGGGGCTCTGCAAGTGATGACAGCAGAGTTCCAAGTGCCATCTCCACTCGTC
CCAACTCGAGAGAGCTTGTTCGTGAGGTACTGCAAGCAGCACGCTGATGGAACCTGGGCAGTGGTTGACGTTTCC
CTCGACAGCTTGCGCCCCAGCCCAGTCCTGAGATGTCGAAGAAGGCCATCCGGTTGCCTGATCCAAGAGTTGCCT
AATGGTTACTCGAAGGTTACTTGGGTGGAGCACGTCGAAGTGGATGACAGGTCTGTTCACAGCATCTACAAGCCA
TTGGTCAACTCCGGCCTGGCGTTCGGTGCAAGGAGGTGGGTCAGCACCTTGGACAGGCAATGCGAGCGGCTCGCG
AGTGTGATGGCCAGCAACATACCCTCAGGAGACGTCGGTGTGATAACTACACCGGAGGGCAGGAAGAGCATGTTG
AAGCTGGCTGAGAGGATGGTGATGAGCTTCTGCGGCGGTGTCAGTGCTTCGACCACACATCAATGGACTACTTTG
TCTGGAAGTGGTGCGGAGGACGTGAGGGTTATGACGAGGAAGAGCGTGGATGATCCCGGAAGGCCTCCTGGTATC
GTCCTCAACGCCGCCACCTCCTTCTGGCTTCCGGTCCCGCCGAAGAGGGTCTTCGATTTCCTACGTGATGAGAGC
TCGCGCAGTGAGTGGGATATCCTGTCGAATGGGGGTGGCGTTCAAGAGATGGCTCACATCGCCAATGGCCAGGAT
CATGGGAACTGTGTCTCCTTGCTGCGCGTCAACAGCACGAACTCGAACCAAAGCAACATGCTGATACTGCAGGAG
AGCTGCACCGACGCGACGGGCTCGTACGTGATCTACGCGCCGGTGGACGTCATCGCCATGAACGTGGTGCTCAAC
GGCGGCGACCCCGACTACGTCGCCCTCCTGCCGTCAGGGTTCGCCATTCTTCCCGACGGGCCATGCGGCAGCGGA
GGAAGGGTGGAGGAAGTAGGCTCCGGTGGCTCCCTGTTGACTGTGGCGTTCCAGATCCTGGTCGACTCAGTCCCC
ACTGCCAAGCTCTCCCTCGGATCGGTCGCGACGGTGAACAGCCTCATTGCTTGCACCGTCGAACGGATCAAGGCT
TCAGTTGCAGGCGAAGTGGCTCGCTGA

GSMUA_Achr5P10510_001 (*MaHDZIV7*)

AGTAGTAGATATAATTACAGGGTATATACATCCGTAATAGTAAGTACCGTCACGTACTCACCAGACAGCAGGAGG
TCCCAAGCCACCACAGCCCTCCGATGCCGATCGTGAACTCCTAGCTCGCCTAAGAAGCTTCTTTACCCTCGTAAA
CCTCACGGACGTTGATGTCCCGTCGCGAAGCGGAGAGCAGAGAGAAGGGGACCAGGAGACCTTTGTGGCTTAGCG
GGAGGAAGGAAGCGGCGAAGCCATGGACTGACTGGCTTCCTTCTCCGGGGCGATGTCGTTGGTCCATGGCGCTCG
ATCGTAATTTTCCGTTCATCGAAACCCGGGAAATAATTATCACTCCGGTCAACATCTGGGGCCCGAGGAATGCTG
GTTCGGTGCATGGGGTGGGCCCACGCCTCGGATGTGGATGGAAGAAGTCGCTCCTCGAAGTCAGCCGTCCCCACC
GTCTCTTCCTCCCTTTGGATTTGATACGTATTTTGTTGTCTTGCCTATTTTATCGCTTTCGATCGAGTTCTCGGG
GCGCATGTTGATGGGGAGGAGAAGCAAACAAAGGAGATTGGGTGGGCGTACCAGCTGTTGGGCTCCCCGTTTTCA
CCTCTGCCTGCTGTGAAAAATCTCGCCTTTTGGTTTTGAGCAGCTGGAAAAAGGTGAAGACGGCAGAAGGTGGGC
GACGCCATCCCCTTCTCCTCCATGCTCCGATTATTTTATCGGAGGGTTGGAGCTTTTTGGATTATCTTTTGCGGG
GCGGATGAGGAGAGAGTCAACCGACCTCTTTCCACTGATTTCTCTCTCTCTCTCTCTCTCTCTCTCTCTCTCTCT
CTCTCTTCAGTCATTGTGGATTTCCTTTTGCTTTGATCTGTGAGGCCTCGTAGTGTCCACATGTGAGCGGGAGGT
CTGGTTTTATCTTCCACATCCATCTCCATCGAGACCAGCTCGCACCAGTCCCTAAGCCTCCTAAAGCTTTGATCT
TTGGGCGTTTTGGAGGAGGGAACTGCATGCCTCGGAGCCGAGAGCTGGCTACTGTTCTGGTCTTGTTTTCTTGTT
CTTCTCTCGATTTGGCTTTCGGTGAGGAGCTCCGAGGGTGCAGAGAAAAGAGAGGGAGAGGGAGAGGGAGAGGGA
GAGAGAACGAGGCGCTAGCTTTCCGCTTGGCTTCCTTCCCTTTTCTTTCTGCCCTTCAGCACTGGGATCGGGATC
TAAAGGCATTTGTTCATAGCCAAACTCGATCATCTATGTTTTTGCCTCCGCTGGTCTCTCCTTCAGATCTTTTCC
TCCGTCTTCTCCTTGAGTTTTCTTCCCTCTCTCCCCTTTTTCGTTGAACTGATCGTGTCGTGTTGTATGCCCTCG
GGAAACTGAGAAATTTGAGTGGGAAAAGGAGAGCAGTCATTAGATCTACTGCTGCCATCGCTAGTCCCTGCAGAA
AAAGGAGCCGCTTTGAAGCAGCAGCCGCGAGTCCGAAGAGCTTAGGTTAAAGAATATAAGAGACCCGATCTTTTA

ATGAGTTTTAGAGGATTGTTCGACGGCGGCTCCGGCGGCGGGGATCGTGTGGTGGCCGATGCGCCGTACCGCAGC
TCCATAGCCATGTCGGCCGGCTCCATCTCTCAGCCGTTCCTAACCTCGCCGTCTCTTCCCAATCCGGTGTTCAGT
TCCCCTGGCCTCTCCCTCGCCCTGCAGACGAACATGGAAGGCCAGGGCGACGCCGCCGTCGCAGGAAAAGGTGGA
AGAGGCGAGCCGGATTCCTTGCGGCGGAGCAAGGAAGATGAGAACGAGAGCAGATCAGGAGGGAGTGATAACCTG
GAAGTAATATCCGGTGACGATTTTGAGCAGGAGAACCCTCGCAAGAAGAAGCGATACCATCGCCACACCCCTCAG
CAAATCCAAGAACTCGAGGCGTAAGCAATCTATGCTTCTTGCTTGCTTCCATTGGTCGTTAGAGCTGATTCACTA
AACAAGAAGAGGGCAAAAGAAGCAGTTAGTTAATTACTACGTTGATTCCCTAACTATAGGCTCTTCAAGGAATGC
CCTCATCCCGATGAGAAGCAAAGGCTGGAACTCAGCAAGAGGTTATCCTTGGAGGCCCGCCAGGTCAAGTTCTGG
TTTCAGAACCGGCGAACTCAGATGAAGGCTAGTTATCACCTCATTCTTTTCTCCTAGCTAATTTGATCCCTCGAT
GTTGTTGTATCTTGCTTACGCGCTTGGAAGCTGACTGAACCAGACTCAAATAGAACGCCACGAAAACACGATCCT
TAGGCAGGAGAATGATAAGCTTCACGCCGAGAACTTGTCGATCAGGGAGGCGATGAGGAATCCCATCTGCAGCAG
CTGCTGTGGGCCGGCGGTGCTGGGGGCGTCTCGCTCGAAGAACAGCACCTGCGCATCGAGAACGCCCGTCTGAAG
GATGAGCTCGATCGAGTTTGCTCTCTCATCGGCAAGTTCTTGGGCAAGCCTATTTCAGTATTATCTGGTCCGGTC
CCTGTCTCGATCTCGAACTCCTCGTTGGAGCTGGCGGTCGGGACGAATGCTTTTGGCGGTCTGGGCTCGGTGGTT
ACGACAACATTGCCTCCGGTAGCTGATTTCGCTGCTGGGTTGTCAAGCCCGCTCGGTACTGTCGTTACTCCTGCT
AGGAACGCCGGCACAGGAGCCATCGGTGGTGGAGCGGATAAGTCGCAGGAGAGATTTGTTCTCTTGGAACTTGCA
CTCACTGCAATGGATGAGCTGCTGAAGATGGCTCAGATGGGGGAGCCTCTTTGGATCCCCGGCGTGGATGGAAGC
AAGGAGACACTGAACTATGACGAGTACGATCGCAGTGTCCCTTGCTGCATCGGCGCCAAGCCCATTGGATTTGTC
TCGGAGGCGACGAGGGCTACCGGCGCGGTGATCATCAATAGCTTGGCACTGGTGGAGACCCTCATGGATGCCGTA
AGGGTTTAGCTTCTATCATCCGATTCCTTGTTCTTGCAGTATTCTTTTGTGCTTACGGTGCTGCATTTCGTTTTT
CTTCGTCCGCGATTCATGAGCAGAATAGGTGGGCAGATATGTTCCCTTCGATCGTTGCAAGATCCACCACCACGG
GTGTGATTTCCGGTGGCATGGGTGGGAGTAAGAATGGATCACTCCAGCTCGTGAGTTCATGGCATTCGGATGTTC
GTTCGTACGTCCCACCTTTTTCCGCTCACACTCATTTCTCATTGCAAACGGATCTCCTGTGTGGACAGATGCAAG
CGGAGCTTCAAGTTCTGTCACCGCTGGTTCCGGTCCGACATGTTCAGTTCCTTCGGTTCTGCAAGCAGCTCGCTG
ACGCGGTTTGGGCGATGGTCGATGTCTCCATCGATGGAATCAGAGACAGCACTTCGGCGCAACAGACCAATTGCC
GCCGGTTGCCTTCGGGCTGCGTGGTGCAAGACTTGCCCAATGGTTATTCGAAGGTATGGAGTTCAATTTATTTTG
CTGCTGAAGTACTCTCTCTCTCTCTCTCTCTCTCTCTCTTTCTCTCTCTCTCTCTCTTTCTAGGCATAAATCT
TGAGCTTCCAAATCTTACTTGGTCATTTGCCTATTTATTTATGTTTCTACAAGTGATGGAATTGTATTAGTTTCC
AGTCTCTGCTTTCTTTATGCTCACTGCTTGTCGTGTTTCTTTAGAGTTTTAGATGCTTCTCTCCCAGTCTCGATA
GCATCACCCCTGAGTCGTTTACTATCCGCCCACAATTAAATCATGATTTGTTCAGCTAACCATGTGGCTAACACT
GCTCTCACATCATTATTCTACGAGAAGGGTTGCCTTCTGTTTCCTATCCCATTGTTTTTCCAAGTCCTCCTGGCA
TACTATGGAAATCTCTCTTAATGACCACACTCTTCCACCCCCTCGTCTTTTGTGCAAACTCCTCGTGAAGGTGTC
TTCATGGATTACTTCGAGATATCATGTACTTGAAACAATTGGTTGCTGTTCTACGATCTGTTCCCCCGGTAACAT
GAATCATGTCAACATCTTTGCATGAGCAATGAGCAAGCATACCACATGAACACTTCCATTATTGGCTCCATGTTA
CTACACTTCTGGCATTTGTCTAACCTTCTGAGTCGAGTGTTATTTGGTGGGCAGTGAACTATGTGCAAAGTTTTG
TCAGGTAAAACAGTAAACTAGTGTGATAACAAAGGTGCAGGCGGTGGCACTGTAGACAATGGTAGCGGACACAAG
GCATTAATGAGCTACCCGACGCTGTGGTGCGCAGGTCACATGGGTGGAGCACGCGGAATACGACGAGGCTACGGT
GCACCCGCTGTACCGCCCGCTGCTGCGCTCCGGCATGGCGCTCGGCGCCCACCGGTGGGTCGCGGCGCTCCAGCG
CCAGTGCCACTCCCTGGCCGTCCTTGTCTCCTCCTCCCTCCCCCCCGGCGACAACACTAGTAAGTCCCAACACAT
AACAAGCGAGGAGGTGATTTGAATTGGAGAGGGCTTACCCTCGGCTGTGCTCTCGCAGCGATCACACCGAGCGGG
AGGAGGAGCATGCTGAAGCTGGCACAGCGCATGACCGACAACTTCTGCGCGGGAGTGTGCGCGTCGTCGGCCCAC
CAGTGGAACAAGCTCCGCGGCGGCGTCGACATCGGCGAGGACGTGAGGGTGATGACGAGGCAGAGCGTGGCCAAC
CCCGGGGAGCCACCGGGCGTGGTGCTGAGCGCCGCCACCTCGGTGTGGCTCCCCGTCGCACCGCAGCGGCTCTTC
GACTTCCTCCGCAACGAGCGGCTCCGGAGCCAGTGGGACATCCTCTCCAACGGCGGGCCCATGCAGGAGATGGCC
CACATCGCCAAGGGACAGGACACCACCGGCAACGCCGTCACCCTCCTCCGTGCTAGCGTCAGTATTTACTCATAC
CTATATATTTTAAGTACATTTTCTGTAATAGAAATGGAGTCTCTTTTGCTGCCTGTCTTTATTACTTGCTCAGAT
GGTGAAAGGTCCAGGAAAAATATTAGTACTGAGCACTGGAAACCCTAACCTTCCGGTGGCGGAAGCACCGTATAC
AGGGAAATGGATGATAGATACGTGAAAAGACGCTTTCCAGATTGACATGACCGAGTGACGTTCTATTTCTGTTTT
GGCGTCTTCTGACGACCCTTTTTCCAACCCTTGTGCCGCACTCTATCGCTGGCTCAGTGGCCCAATGTCTTCTCC
ATGATTCTGCCATAATAATTTACCCTGTAGCTTCTCGTGAAGATTTACGTTTCGGTGATCTGTTTTGCACCAGGC
GGTGAGCGCGGACCAGAGCAGCATGCTGATACTGCAGGAGACGTGCACGGACGCGTCCGGGTCGCTGGTGGTGTA
CGCCCCGGTGGACGTCCCGGCCATGCACCTCGTCATGAGCGGCGGCGACTCCTCCTACGTCGCGCTCCTCCCTTC
CGGCTTCGCCATCCTCCCCGACGGTCGTAAGGCGGTAGGCTCGCTCCTCACGGTCGCGTTCCAGATACTGGTGAA
CAGCCAGCCGACGGCGAAGCTGACGGTGGAGTCGGTGGAGACCGTGAATAACCTCATCTCCTGCACCGTCCAGAA
GATCAAGGCCGCCCTCCGCTGCGAAGCTTGAGAAAGGAACCTGTGATGGCTGTCCTGTGGGCCGAGTCAAGGACG
AACCGCGTGGCGAAAAGCGTGGCTAAGTGGCAAGGAAGCATGGAAGGAAGCACCGGTAAAACCCTAACGCCTTCA
GGGGAAGTGGGTAAACCTTAACACACGGAGCCACGGTGGTGGTGGTTCGGGTGTTGACTCGGTCTAATCGACGCA
ATTGCTGCATGTAGTGTTGCAGTCACCCTCGTTTTGGTGGATTTTTCCTGTCTGACATGGAAACGAAGCAGGTTT
TAGCCTCGTTTGCAGCATTTTGAGCGTGTATTTTGTTGGGTATAGTCCACCAATTTGTCTGTGATCTTTATTTAT
ATTATACTATTGGGATACATAAATAAATCTAATGATTGAATATAGTTTGGTCTCTAATCTCACGTTTCAAAGATT

AGACACTTTATGTATCGAATATATATATATATATATTAATTTTATTATTTGTTATTTACATATATATCTTATTTGAC
GTGACG

CDS

ATGGGTTTTAGAGGATTGTTCGACGGCGGCTCCGGCGGCGGGGATCGTGTGGTGGCCGATGCGCCGTACCGCAGC
TCCATAGCCATGTCGGCCGGCTCCATCTCTCAGCCGTTCCTAACCTCGCCGTCTCTTCCCAATCCGGTGTTCAGT
TCCCCTGGCCTCTCCCTCGCCCTGCAGACGAACATGGAAGGCCAGGGCGACGCCGCCGTCGCAGGAAAAGGTGGA
AGAGGCGAGCCGGATTCCTTGCGGCGGAGCAAGGAAGATGAGAACGAGAGCAGATCAGGAGGGAGTGATAACCTG
GAAGTAATATCCGGTGACGATTTTGAGCAGGAGAACCCTCGCAAGAAGAAGCGATACCATCGCCACACCCCCTCAG
CAAATCCAAGAACTCGAGGCGCTCTTCAAGGAATGCCCTCATCCCGATGAGAAGCAAAGGCTGGAACTCAGCAAG
AGGTTATCCTTGGAGGCCCGCCAGGTCAAGTTCTGGTTTCAGAACCGGCGAACTCAGATGAAGACTCAAATAGAA
CGCCACGAAAACACGATCCTTAGGCAGGAGAATGATAAGCTTCACGCCGAGAACTTGTCGATCAGGGAGGCGATG
AGGAATCCCATCTGCAGCAGCTGCTGTGGGCCGGCGGTGCTGGGGGACGTCTCGCTCGAAGAACAGCACCTGCGC
ATCGAGAACGCCCGTCTGAAGGATGAGCTCGATCGAGTTTGCTCTCTCATCGGCAAGTTCTTGGGCAAGCCTATT
TCAGTATTATCTGGTCCGGTCCCTGTCTCGATCTCGAACTCCTCGTTGGAGCTGGCGGTCGGGACGAATGCTTTT
GGCGGTCTGGGCTCGGTGGTTACGACAACATTGCCTCCGGTAGCTGATTTCGCTGCTGGGTTGTCAAGCCCGCTC
GGTACTGTCGTTACTCCTGCTAGGAACGCCGGCACAGGAGCCATCGGTGGTGGAGCGGATAAGTCGCAGGAGAGA
TTTGTTCTCTTGGAACTTGCACTCACTGCAATGGATGAGCTGCTGAAGATGGCTCAGATGGGGGAGCCTCTTTGG
ATCCCCGGCGTGGATGGAAGCAAGGAGACACTGAACTATGACGAGTACGATCGCAGTGTCCCTTGCTGCATCGGC
GCCAAGCCCATTGGATTTGTCTCGGAGGCGACGAGGGCTACCGGCGCGGTGATCATCAATAGCTTGGCACTGGTG
GAGACCCTCATGGATGCCAATAGGTGGGCAGATATGTTCCCTTCGATCGTTGCAAGATCCACCACCACGGGTGTG
ATTTCCGGTGGCATGGGTGGGAGTAAGAATGGATCACTCCAGCTCATGCAAGCGGAGCTTCAAGTTCTGTCACCG
CTGGTTCCGGTCCGACATGTTCAGTTCCTTCGGTTCTGCAAGCAGCTCGCTGACGCGGTTTGGGCGATGGTCGAT
GTCTCCATCGATGGAATCAGAGACAGCACTTCGGCGCAACAGACCAATTGCCGCCGGTTGCCTTCGGGCTGCGTG
GTGCAAGACTTGCCCAATGGTTATTCGAAGGTCACATGGGTGGAGCACGCGGAATACGACGAGGCTACGGTGCAC
CCGCTGTACCGCCCGCTGCTGCGCTCCGGCATGGCGCTCGGCGCCCACCGGTGGGTCGCGGCGCTCCAGCGCCAG
TGCCACTCCCTGGCCGTCCTTGTCTCCTCCTCCCTCCCCCCCGGCGACAACACTACGATCACACCGAGCGGGAGG
AGGAGCATGCTGAAGCTGGCACAGCGCATGACCGACAACTTCTGCGCGGGAGTGTGCGCGTCGTCGGCCCACCAG
TGGAACAAGCTCCGCGGCGGCGTCGACATCGGCGAGGACGTGAGGGTGATGACGAGGCAGAGCGTGGCCAACCCC
GGGGAGCCACCGGGCGTGGTGCTGAGCGCCGCCACCTCGGTGTGGCTCCCCGTCGCACCGCAGCGGCTCTTCGAC
TTCCTCCGCAACGAGCGGCTCCGGAGCCAGTGGGACATCCTCTCCAACGGCGGGCCCATGCAGGAGATGGCCCAC
ATCGCCAAGGGACAGGACACCACCGGCAACGCCGTCACCCTCCTCCGTGCTAGCGCGGTGAGCGCGGACCAGAGC
AGCATGCTGATACTGCAGGAGACGTGCACGGACGCGTCCGGGTCGCTGGTGGTGTACGCCCCGGTGGACGTCCCG
GCCATGCACCTCGTCATGAGCGGCGGCGACTCCTCCTACGTCGCGCTCCTCCCTTCCGGCTTCGCCATCCTCCCC
GACGGTCGTAAGGCGGTAGGCTCGCTCCTCACGGTCGCGTTCCAGATACTGGTGAACAGCCAGCCGACGGCGAAG
CTGACGGTGGAGTCGGTGGAGACCGTGAATAACCTCATCTCCTGCACCGTCCAGAAGATCAAGGCCGCCCTCCGC
TGCGAAGCTTGA

GSMUA_Achr4P19010_001 (*MaHDZIV6*)

CATTAACGATAGATTCGTGGGAGTATCATCATATGAGTTGAATTGGAAATGTGTTGAGGAGAGATTCTGATATTT
AAGGCACTGTGTGAGTGCAGTTACGAGAAATATCGACTCTCGCTCAAAGTTTGATATGTATATATGTTAGGTTTG
ATTTGTAAGAGTAACAACATATAATGATCCCATTTCACTTTGCCTTCTCGCTAAATTACACATGGCACAACATTG
GTGCCACTTGGAAGGCCACTCATCACCGATCCCATCAACATTTGCTACTCGTCTCGGTGTACTCGAACCGTTTAC
CTTCCTTGCGATCGAAAGTTGTGGTGGCCCTCAACGAAATTATCCGCTGGGCGTTGGTGCCTCCATTCACAAGG
CAGGTAGGTCTAAAACTCTTTTCTTCACATCGACTGCGCTCGTAAGTCAAAGTCCTTTGACCGCCCCTGCGTTAT
TGCCTTTTTTTGGTTATATCGGAAACCCCCCCACCCCCCCCCCCGTTGGACTTCTTAACTTCCCGCTCCGCCAC
TAAATCGAACGTCATCAAGCGAATCAGAGCCGTCCAGTAGGTGGACCTCGGAAGCCACGGCGGGTCCGGCCGGGG
ACGTTTCGAACCGCGGAGAGAGAAAGGTGCAGTAGAAGACAGAGAGAAAGCGCGTAAGGGGGGGCGGGGAGGGAG
GAAGAGAGACAGAGAGGAGGGGGAGAGGGAAGGGGAGTTGACCTAGGGCAACAGACCGATCGCTGCTCGGGCTGC
CGAAAGACACGTGTCGGTTACGGTGGGCAAGACGGATTTGCTGGCGGGGGATCGGTTTCTGCCACCCGCAGTTAA
AGTCGCATTTACCATCCATNNNNNNNNNNNNNNNNNNNNNTCCCTCCTCCTCCTCCTCCTCCTCCCTTCCCTG
TGAAACCTCCCTCCCAGTAATGACCTCCCACCAGTAATAAATATTAGATAACTTTTTGGTCTAAGAGAGAGAATC
CTCCTTCTCCTCCCGATAGAGATAGAGAGCAGAGTTACTATGGTTTGTCCGTGAAGCAGCATCGAGATCATGTAG
AGAGAGGTGGAAGAAGAAAAACAGAAAGCCAAAGCGTAGGTCTACAAAACAGTGCAGGAGAAGAAGAAGAGGAGG

AGAAGTCGGAATAGAAGAGGACTTGTATCTTTTTTTTATCCTCCTGATCTTCCTCCCTTTCTCCCAAATATCACCG
ACAGTGGTGAGTGGTATTTATTTCTCACCATTTACTGCTCCTCCCTTTATCCCATCCTCTCTTTCAGATCTCCTC
TCTCTTCTTCTTTGCTCGGTATATCTACTCTCCTTTTCTTTTCATTCTTAAAGGATCTCTCCATTCTGTTGTGTT
CGGTTCTGTTTAGGAGAGGTTTGAATGATATGGCGAAGATGGAGGAAGTGTGGCTTTGAGAAAGGCAAAACAAAA
AACACTCGAGTGCTTCCTTGTTTCTAACCATTTCATCAGGGAAAAGAAGTAGTGGTGAACAAAGAGGGGCTAAAG
ATGTATGGAGATTGCCAAGTGCTGTCATCCATGGTGAGTGGTAATGTTGCGTCACCCGATTCACTCTTCGCCTCT
CCGATCCAAAACCCTAACCTCGGCTTCATGGCCAACATGCCCCCTTTCAACGCTTTCTCCTCTATCATTCCGGTA
AGCACCAGTCTTCCCCTACATATCGACTTTTTTTTTTTTTTTTCTCTGTGCATCTGCTTCTTTTTTTTTTTCTTG
TTCTTTTCCAAAAGAGATTATTGCTTAGATTTATCTGTTTTGGTGCAAGAAGGAGGAGGGGCTGATGTTGATTGG
GAGGGGAGTGAGCAAAGAAGAGGAGATGGAGAGTGGGTCTGGTAGCGGACCCTTGGACGGAGTGCTTAGCTGCGG
CGAGGAACATGATAACGAGTTGCAACAACAACCACCATCGCAGCAGCAGCAACAGCCGGTTGCCAAGAGGAAGCG
CTACCACCGCCACACCACCCGACAGATTCAAGAAATGGAAGCGTGAGATCAGATTCTCCCACATACCTCGTCATT
CATTCGATACATCATTCGTTTTGACTATTCTTTTTGATGGGGCATAGAAGACTGATGTGATCCGATGCTGATTTT
TGGAATAGGTTGTTTAAGGAATGTCCGCACCCGGACGAGAAGCAGAGGATGAAGCTAAGCCAAGAACTCAGCCTC
AAACCTCGGCAGGTCAAGTTCTGGTTCCAGAACCGCCGGACCCAGATGAAGGTCCGCCCATGCGTTCCCAATCCT
TGCACCATGTTTCCGTCAACCGCATCTAACTTTTTGATCATCGTTTGATGAGTACATATCCTCACGCGTGCATGC
ACATTCATGCACCCGCAAACCACGATCAAAAACTAGAAATCATTTCTTATTAGCGCACAATGTTTACTGATCCAT
ATGGTTTTGGGACTGGGGACTACTGAAGGCGCAACAAGACCGGGCGGACAACGTGGTACTGCGAGCCGAGAACGA
GAGCCTGAAGAACGAGAACTTCCGGCTGCAGGCTGCGATTCAGAACGTCGTCTGCCCCAACTGCGGCGGCCCGGC
GATCCTCGGCGAGATGTCTTTCGACGAGCAGCAACTGCGCATCGAAAACGCCCGACTCAAGGACGAGGTACTAAA
CCCTAGTTCCAGCAAATCAACATCATTAACCTCTTCCCGGAACAACATCACATGCATGAGACAACGCAAAAACTG
GAGTTCTAAGTCAAAAAAAGTTTTTCTTGTGCTACATGCTAGATTGTGTGTATCTCATCCTTTTTGAATCAGCTT
CTTTATGGAGGACACACAGTCAGTTCAGCTGCCTTTTTATGTACAGCTCGAACGTCTATCCTGCATCGCATCCCG
CTATAGTGGCCGGCAACAGCAGCCGTTAGGTCCCGCACCGCCTATACTGCTCCCCTCGCTGGATCTCGACATGGG
CATCTACTCGAGGCACTTCAAGGAGCCGCCAGTGGTGAGTTGCAATGACATAATCCCCGTTCCACAGATCTCCGA
CCAAGCCTCTCCCTTCTCTGGCATGCTCATCCTGGACCAAGAGAAACCGTTGGTTCTGGACCTAGCAATCACGGC
GGCGGATCACTTAGTCAGGATGTGCCGTACGAACGGGCCACTATGGATTAGAAGAGATGGTCGCACCACGGAGGT
CTTGGATTTGGAAGAGCATGCAAAAAACTTCTCCTGGCCGATGGACTTGAAACAGCAGCATGGAGAGATTAGGAC
CGAGGCTTCCAGGGACAGCGCCATGGTCATTATGAACAGCATTACGTTGGTCGATGCCTTCCTGGATGCCGTGAG
TTAATCGTTATAAGATTTGTATGTGTTCATTAGTCCATTTTGTGAGCTGCTTGTTGGTCATTTCTCTTTCTAATC
GTGCTGTCCAGGACAAATGGATGGGGCTGTTTCCTTCCGTCGTCTCCAAGGCTACAACAGTTCAGGTGTTGAGTC
CAGGAGTCGCTGGTCATGGCAATGGTTGCTTGCATCTGGTAATCTAGCATCTGCTGCACCGTTGTTTCCGCCCTG
TTCGATACAAGTGTTCTCATGTTGGAGCTATTGACAGTTGCACGCAGAGCTGCAATTTCTGTCTCCTCTGGTGCC
TGCGCGTGAGGCTCATTTCTTCCGGTACCTCCAACACAATTCAGAAGAAGGTACTTGGATAATTGTTGATTTCCC
TGTCGATGGCTGCGTCGATGGCCTTCAAACGTCCCTCCCCTGGTATAGAAGGAGGACCTCTGGTTGTGTCATCCA
AGATATGCCCAATGGGTATTCCAAGGTACAAAACGAATTACGTTTTCTTTCTGCTAAGTTGATGTTTTCAATTAA
AAGCTCAGTTCTAATCCCTTTCATTAATGATGTGAAATTACTGAAACTAGTTTCGATTAAGTTACAGGTTTCGTA
TTGATCGGTACACGATCAATTATAATTGTCTTTGTTCCATTATATGATAAGCACAAAATGTTAACTTCGACATGT
ATGGATGGGAATACAGAATTTAGTAGCAAAAAGGTTATCATCTTTCATTAGGGCCTGACTTATAGAAGATAAGAT
TGGCGCTTGTACTGATGCCCACCGCATGCCCGCCATGTTCATGGATCATGACTCGAAAGAACTGGTAACTGGAGG
CTCTGCATACGTTCCTACTGGAATGAAATATTGTATGAACTGATGTTATTAATCAAGCATCGGGATGCAAACGTT
CCAAGGCACTTTTTTTGTATTTATATAAAACAAATTATGCACTAGAATTTTAGAGAACATTACAGAAGCATATGA
CTTGGGGAATAGCAGCATTAGAACTGTGCATTTAATGACATTTGTTTTTGGATATCAAGTCATAATAAAATTTAG
GATGGATAGATTCATCTGTCTTGTTTGGGCAGGTGATGTGGGTAGAACATGCAGAGGTGGAGGATAAACCAGTGC
ATCAGATATTTGATCAATTTGTCAGCACCGGCGTGGCGTTTGGAGCAACACGCTGGGTTTCAACCCTGCAAAGGC
AATGTGAGCGGCTTGCAAGCCTTCTGGCCAGAAATATTGCCGACCTTGGAGGTACAGTGTCTAATAATGCTTGGC
ATAGATTCTGGCTTCTTTGTCTTTATATATATATATATATACTCTATAAGCTGTGATTCTCTCATCTAAATGGAC
TTGTGTATCCCTTCAGTGATCCCCACCCCAGAGGCAAGAAAGAATATGATGAAGCTGTCACAGAGAATGATGAGA
ACGTTCTGCGCCAGCGTACACGCCTCCGGAATGCAATCTTGGACAGCTCTTTCTGAATCATCCGATGACACAATC
AGGGTGACAACCAGGAAAAATACTGAACCTGGCCAGCCAAATGGTGTTATTCTCACTGCTGTCTCGACAACATGG
CTTCCCTTCTCTCATCAACAGGTGTTCGAGCTTTTGACGGACGAGCAAAGGAGGTCTCAGGTACCTGAACATAAG
ATTAGCAGCTTGTTCTTGCTTTCAGAGAACCCTTTTTTGTGTTTTTGTCAAAGCTGAAATGAAATGCCTGTTTCT
GCTGCAGTTGGATGTTCTCTCAAGCGGTAACTCCTTACATGAGGTGGCTCATATCGCCAATGGTTCGCACCCAAG
GAACTGCGTCTCGCTTCTGCGTGTTAATGTCAGTACGCCCTTCTATTGCCTGCGACTTGTTGCCTCAAACTTTCC
ATCTTTTGCAGGAGTTTTTAGATCATTCCTTCTGCTTAACCACAAGAAAAGAAAAAAGATTATTTGTTTGCACTT
TTGTTACACTACGAACTGCTTATACGTCTAATATTATTTGTTTGCCTCATTTTGTCGTTGGTTGCTGATGAATCT
GTTTCCGCATGCCAACAATGATATATCCACTCAGGCGGCAAGCAACTCCTCCCACAGTGTGGAGCTCCTGCTTCA
AGAAAGCAGCACTCACCCCTCCGGCGGCAGCATCGTCGTGTACGCTACCATCGACGTCGACGCCGTCCAGGTGGC
GATGAGCGGCGAGGACCCGTCTTATATTCCCCTCCTCCCAACTGGCTTCGTCATCTCGCCCGCTGCCCCACCAAA
CGGCGTCATCAGCAGCAGCGATGGTTCGGCCAGTACCATCGGATGCCTGCTCACAATCGGCATGCAGGTTCTGGC
CAGCGCAGTCCCATCTGCCAAGCTCAACCTCTCCAGCGTCACCGCCATCAACAACCACCTCCGCAACACCGTGCA

ACAGATCAGCGCCGTGCTCGGCGGTGGCGCCGTGGCCGAGCCGGCTGCTATGGCGCCGGAACAATAGCCCTAATA
AGTAAGTGGCTGGAGTCACCTGAACCCTAACTATTTACCCGATATAACCAAATTGCTCTTCGCTCCACTCTCAT
CACACCAGAATCATCATCATTCTGGCGCGACAAGGGACTACTTGACAAAGCCCAACCATTGACGCTTATGCCCAC
GATCTTTGAGGGCATCCCACATCAGTTCAGTGTACTTGGGGCTTTGTCAAGTTTTCGCTTGTTCAACCTGAGTGT
ATGGTGGGGGGGGGCCATTTTGGTCGTATCGGTGCTCAACAATGGGATCCAAGGTGATGTCCAGGGAAAGTGGAG
GTCGCACCATGTGTAATTAAGAACAATGCAGTAGGAGGTTTATTAGTTGAAATAGCAGCATTGTGTAGAGTGGAA
AGCGAACAAGGAAAGGGAATGTAGGTTGTTGGAGGGAAGTCAAGAGCGCACCAGATGTGACCCTCGCCCCAGCCG
GCGAAGTCTCTCACGAGGTAGAAGATGAAGAGGGTGTTGGTT

CDS

ATGTATGGAGATTGCCAAGTGCTGTCATCCATGGTGAGTGGTAATGTTGCGTCACCCGATTCACTCTTCGCCTCT
CCGATCCAAAACCCTAACCTCGGCTTCATGGCCAACATGCCCCCTTTCAACGCTTTCTCCTCTATCATTCCGAAG
GAGGAGGGGCTGATGTTGATTGGGAGGGGAGTGAGCAAAGAAGAGGAGATGGAGAGTGGGTCTGGTAGCGGACCC
TTGGACGGAGTGCTTAGCTGCGGCGAGGAACATGATAACGAGTTGCAACAACAACCACCATCGCAGCAGCAGCAA
CAGCCGGTTGCCAAGAGGAAGCGCTACCACCGCCACACCACCCGACAGATTCAAGAAATGGAAGCGTTGTTTAAG
GAATGTCCGCACCCGGACGAGAAGCAGAGGATGAAGCTAAGCCAAGAACTCAGCCTCAAACCTCGGCAGGTCAAG
TTCTGGTTCCAGAACCGCCGGACCCAGATGAAGGCGCAACAAGACCGGGCGGACAACGTGGTACTGCGAGCCGAG
AACGAGAGCCTGAAGAACGAGAACTTCCGGCTGCAGGCTGCGATTCAGAACGTCGTCTGCCCCAACTGCGGCGGC
CCGGCGATCCTCGGCGAGATGTCTTTCGACGAGCAGCAACTGCGCATCGAAAACGCCCGACTCAAGGACGAGCTC
GAACGTCTATCCTGCATCGCATCCCGCTATAGTGGCCGGCAACAGCAGCCGTTAGGTCCCGCACCGCCTATACTG
CTCCCCTCGCTGGATCTCGACATGGGCATCTACTCGAGGCACTTCAAGGAGCCGCCAGTGGTGAGTTGCAATGAC
ATAATCCCCGTTCCACAGATCTCCGACCAAGCCTCTCCCTTCTCTGGCATGCTCATCCTGGACCAAGAGAAACCG
TTGGTTCTGGACCTAGCAATCACGGCGGCGGATCACTTAGTCAGGATGTGCCGTACGAACGGGCCACTATGGATT
AGAAGAGATGGTCGCACCACGGAGGTCTTGGATTTGGAAGAGCATGCAAAAAACTTCTCCTGGCCGATGGACTTG
AAACAGCAGCATGGAGAGATTAGGACCGAGGCTTCCAGGGACAGCGCCATGGTCATTATGAACAGCATTACGTTG
GTCGATGCCTTCCTGGATGCCGACAAATGGATGGGGCTGTTTCCTTCCGTCGTCTCCAAGGCTACAACAGTTCAG
GTGTTGAGTCCAGGAGTCGCTGGTCATGGCAATGGTTGCTTGCATCTGTTGCACGCAGAGCTGCAATTTCTGTCT
CCTCTGGTGCCTGCGCGTGAGGCTCATTTCTTCCGGTACCTCCAACACAATTCAGAAGAAGGTACTTGGATAATT
GTTGATTTCCCTGTCGATGGCTGCGTCGATGGCCTTCAAACGTCCCTCCCCTGGTATAGAAGGAGGACCTCTGGT
TGTGTCATCCAAGATATGCCCAATGGGTATTCCAAGGTGATGTGGGTAGAACATGCAGAGGTGGAGGATAAACCA
GTGCATCAGATATTTGATCAATTTGTCAGCACCGGCGTGGCGTTTGGAGCAACACGCTGGGTTTCAACCCTGCAA
AGGCAATGTGAGCGGCTTGCAAGCCTTCTGGCCAGAAATATTGCCGACCTTGGAGTGATCCCCACCCCAGAGGCA
AGAAAGAATATGATGAAGCTGTCACAGAGAATGATGAGAACGTTCTGCGCCAGCGTACACGCCTCCGGAATGCAA
TCTTGGACAGCTCTTTCTGAATCATCCGATGACACAATCAGGGTGACAACCAGGAAAAATACTGAACCTGGCCAG
CCAAATGGTGTTATTCTCACTGCTGTCTCGACAACATGGCTTCCCTTCTCTCATCAACAGGTGTTCGAGCTTTTG
ACGGACGAGCAAAGGAGGTCTCAGTTGGATGTTCTCTCAAGCGGTAACTCCTTACATGAGGTGGCTCATATCGCC
AATGGTTCGCACCCAAGGAACTGCGTCTCGCTTCTGCGTGTTAATGCGGCAAGCAACTCCTCCCACAGTGTGGAG
CTCCTGCTTCAAGAAAGCAGCACTCACCCCTCCGGCGGCAGCATCGTCGTGTACGCTACCATCGACGTCGACGCC
GTCCAGGTGGCGATGAGCGGCGAGGACCCGTCTTATATTCCCCTCCTCCCAACTGGCTTCGTCATCTCGCCCGCT
GCCCCACCAAACGGCGTCATCAGCAGCAGCGATGGTTCGGCCAGTACCATCGGATGCCTGCTCACAATCGGCATG
CAGGTTCTGGCCAGCGCAGTCCCATCTGCCAAGCTCAACCTCTCCAGCGTCACCGCCATCAACAACCACCTCCGC
AACACCGTGCAACAGATCAGCGCCGTGCTCGGCGGTGGCGCCGTGGCCGAGCCGGCTGCTATGGCGCCGGAACAA
TAG

GSMUA_Achr3P12410_001 (MaHDZIV3)

AATATTTATCGAAAATACAGATAATAGTTATAATTATTATTATTGGGAGGGGATGTAGAAATACGAGAATGCAGC
CCACGCAAAGCAACGCGCGGTGGGTTCAGTTAATCTCACCGGATGCTCCGCCACACCGTGAGTCGCACCTGAACC
TGAACTCGAAGCTGGTCACCGCCTCAGTCAATTCCCCCCTCCCCCTACTTCCTAAAGTAGCGCGGCCTGGCCGTT
TCAACCGTAGGATCTCGATCGAACGGACGCCGCTCGCCTGATCCTGTGATGCACCGTCGTTGTTAAGACCGAGGC
CGGGGGCGATACCGTCATTTCGGCTCTCCGCGGTGAGCTTATAGACCCCATGCGTTGCATTCGTCCCCTCTAATC
CCCACCTCCTCGCTCTCCCCCCCTGTTCTTCTTCTCCTCGTTCGAAGTCGCCGTCGGTCACTTTCCGGGCTCCTG
GATTCGATCCGTTTCTTACCCTGTGAAAGAGTGAGGTAATTGCCATGGAAAGGAGTCTGCTTTTTCCCCCTCTTT
TACTGCTGTCTTGCAAGTCGCAGGGGTCGTTTTTGACTGCAGGGCGCTGCTGCCGTCTGCGACATAGTCAAAACC
TTTTTTATTGATCTGCCATGGTGCAGAACGTGGTCGCTCTGCCGCTCGCTCTCTCTCTCTCTCTCTCTCTCTCGT
TCTCGCTTCGGGTTAGCGAGAGTGTGTAAAGGGCCTTCTCTTCTGTTTTAATCTCGGTGTCGGCTTTGGACCTTT
CCTTTTGGTTTCATCTTTTACGAGGGATGTGAGAGAGAGGGAGAGAGGGAGTTGTGGGGGTCAAATGAACAGCGA
TCGGTTGTCGTTTTTGTTTATCTGTGAGGCCGAAGAGTCCACAATTCCGGTTTTCATGTTTGAGGTCTCCCTCTC
CGCTGCTGGCATCTCTGAGGCGCTTCCGGGTTTGCTCCCCTGCTTGTCTTTCTTCTGCCCCAAAGGGTGGTGCAG
TGGAGAGCCAAAGATCGGATTCTTCGCTCTTTTTCACGTTAAAAATTGGATCTTGTGGTTGGGATTGGGACTGCT

GCGAGTGAGTTAGGGTTCTGATCCAGGCTGTAGATAGGAGGAGCCGGGGAAGAGGGGGAGAGGAAGAGGTGACAC
AGCAGCACAAGTCTTGTCTTCTTTCTCCTTCTTTACCAGGAAAGAGGGAGAGGGAGTTGGAGGAGGCATTGGTTT
GCAGTAAAGCCTGCGGTTTTCGCCTTCTCTGATCTCTCTCCATTTCTGCCTCTTTCCTTAATTCTGTACAGTATA
TTCTCTGTATCCATCTTTTCCCTTTCTTTCACTGTACTCAATATTGATGCATCATTAAAACCGATTAAATTCCTC
AGAAAAATTCTGATTCTGGGCAAGCGAAAGCAAAAGATCCGATCCCCAGACAAGTGCAAGTCGGCGAGGAAAGAA
AATATTATGAGAGCACAAGCTAGCACGGAGGACCAAGTCTGGAGGGAGAAGAACCAAGAAAAGGAAGGATATTTG
ATGAGCTTTGGGGGGTTATCAGACGGCAGATCAGGAGGCGCTCTAGCTTACGGCAACGCGGCCATGACCGCCGGA
GAGTTATCTCCGCCGCAGCTCATCTCCCAGAGGCTCCCAAACTCTGCCTTCATTTCTCCCCGCCTCTCCCTCGGC
CTGGTAAACGAGACCGAACCTCGGACCATGTTTCCTTCCCTTCGATTCATTCATGACGCTTTCTCTTTTTTGGCT
TTTTGGGCCAGCAAACGAACGTGGATGGACAGATAGACATGAGCAGGATGGCAGGCGTCGGCGGTGGCAGTGACG
TGGGTTCGGCCAAGAGGAGCAAGGACGATGAGAATGAGAGCAGATCGGAGAGCGACAACTTGGAAGCCATATCCG
GTGACGACGTCGATCAGGAGAATCCTCGCAAGAAAAAGAGACACCACCGCCATACTCCGCAACAGATCCAGGAAC
TCGAAGCGTAAGACCGATCCTTGAATCCATTGGCAATCTGTGGCCTTTTCTTCTTCCTGATCGCCTGTTCCATGC
TGTAGTCTGTTCAAGGATTGCCCCCACCCGGATGAGAAGCAAAGGCTGGAACTGAGCAGGAGGCTATGCTTGGAG
AGCAGACAAGTCAAGTTCTGGTTCCAGAATCGACGCACCCAAATGAAGGTAGGAACGATACGAGACACACAAATC
CTCTCTTTGTTGCTTGGATTGTGGTTCCCTTTAGAACTTTTGCTCGTTGATGGATTACAGACGCAAATCGAGCGC
TTCGAGAACACGAAGCTCCGGCAGGAAAACGACAAGATGAGGGCGGCGAACATGGCGATTCGAGAAGCAACGCAG
AGCGCGGCTTGCAAGAACTGCGGAGGTCCGGTCATGCTCGGCAATGTCTCGGTGGAACGGCTGCAACTTAGACTG
GAGAACGCTCGGTTAAAGGAAGAGCTCGACCGGCTGTGCGCAATCGCCAAGAAGTTTCTGGGGAAGTCCGTCTCC
TCTTTGGCCGGCCACATCTCTCCGAACATGCCGAGCTCGGTTTTGGAGATTGGAGTCGGGAACAATATCTTCGCG
GGGTTCAACTCCGTCGTCGCTCCGACTATTTCCGCTTTGCCCGACTATTTGCCGGGGTTCAGCAGCAACCCACTG
GATGCTGTCGCTCCGACCATGGGCAAGAACATGGAGGCGCTGCTGGGGAGCGACAGGAGCATGCTCTTGGAGCTT
GCATTGGCTGCAATGGAAGAGCTAATGAGCATGGCGCAGATGGAGGAGCCACTTTGGCTTGCGAGCTCACAAGGT
GGTCCCAAGGCACTGAACTACGAGCAGTACCGCCGGACGTTTCGCCCCATCGCCGGGATATCGCCGGTTGGGTCT
GTGTCAGAGGCCTCGAGGGAGACGAGGATAGTGTCCATTGACAGCGTCGCCCTCGTCGAGACCCTAATGGATGCG
GTAAGTTATGATCTGTTAATCCTACTTCTCCGATGGTGGATGGATAGCTTCAGCTTCGTGACATGTCTTCTTGTC
CGTAACGCAGAGTCGTTGGGTTCATATGTTCCCAAGTATCGTGGCCAAGGCCACCACCACGGAGTTGATATCCAG
CGGCGTGAGTGGAACAAGAGACGGCGCCCTCCAACTCGTGAGCCAATCGTTGCACTGTGTAATGTGTTGCCTTCA
CAAATACCATGGGTTTCTTTCTGACACCGAGCTTTGCGAATGGTTCAGATGCAGGCGGAGCTTCACGTCCTCTCC
CCGCTGGTGGCAGTGCGCGAGGTGAGCTTTCTCAGGTTCTGCAAGCAACACGCGGAGGGCGTTTGGGCCGTGGTC
GATGTCGCCGTTGACGGCATCAGAGCAGATCTCTCTTCTGCTAACTGCAGGAGGCTGCCGTCTGGTTGCCTGGTG
CAAGACATGCCGGATGGCTACTCCAAGGTCCTCCTCTCCCTGTCTTTAGCCGAGCAAAATCTTTCTTATTCGATC
ACAAGAAAAATCTCATTTAGATCTTCCTCCTACTACCTCGGGACTTGAGTCTTGATCAACATTTTTGTCTGTGTG
TCGTTATCCCTTTTATCCAATTTCTTTAATGCCATCCCCACTGCACAACATCTTCTCCTTCTTAGTTGCCCTTTA
CGTACCTGTGGAGGTATGAATGCTTCGTGTTCATGTCCTGCACAAATAATAAATGAGAAATATGAAAGAAGTAGA
TGTATTAAGATGAAGTACTAACGATTAGACCAAATGGCGGTATCATTGTTTGATTGAATTGGCAGGTCACCTGGG
TGGAGCACGCGGAGTACGACGAAGGCCAAGTGCATCATCTGTATCGCCCGCTGCTCCGCTCCGGCCAGGCGTTCG
GCGCCGGACGCTGGGTTGCCACCCTACAGCGCCAGTGCGAGTGCCTTGCCACCCTCGTGTCCTCCACCGCCGTGG
CCCGCGACGAGACTGGTAAGGAGCCAGCCACTTGATCTCTGAAGCATCGTGAACAGCGTCGTTTGACTGATTCTT
ACGATATTGTGATCTCGACAGCATCGATAACGGCGAGCGGGCAGAGGAGCATGTTGAAGCTGGCGCGTCGGATGA
CCAACGCCTTCTGCGCCGGGGTCTGCGCGTCGCCCGCGCATGGGTGGAGCAAGCTGGCGTCGGAGACCATCGGGG
AGGACGTGCGGGTGATGTCGAGGATGAGCGTGAACGTACCGGGTGAGCCGGCGGGGTTGGTGCTCAGCGTGGCCA
CATCGGTGTGGATCCCCGCGCCGCCCAAGCGGCTGTTCGACTTCCTATGCGAGACCAGATTCCGCAGCAAGTGGG
ACATCCTCTCCAACGGCGGGCCGATGTGCGAGATGGCGCACATCGCCAAAGGCCAGGAAGCAGCCAACCGCGTCT
CCCTCATATGGGCCACTGTAAGCGAACCCTGCCTGTCTCTCTCTCTCTCCCTCCCTCCCCGCCCCCTCTTTAACC
CTCATCGTCCACGGTGTGCTGCACGAACATAACTTGTGATCCCGGTGACGCGCAGGCCACGAACGCCAGCCAAAC
CAGCATGATGCTACTGCAGGAGACCTACACCGACGCTTCCGGCGCAATGCTGGTGTACGCGCCGGTGGACATCCC
AGCCATGCACCTAGTGATGAACGGCGGCGACTCCGCCCACGTCGCCCTCCTCCCTTCCGGTTTCGCCATCGTCCC
CGATGGCGCCGGCTACCGAGGAGAGCTCACCGAAGAGCACCACAGCGACGGCGCCGCCACCGCCGGCTCCGGTTC
GCTGCTGACCATCGGGTTCCAGATTCTGGTGAACGGCCAGCCGACGGAAAAGCTTACGGCGGAGACGGTGGACAC
CGTCAACGGCCTCATCTCATGCACGGTTCAAAAGATCAAGGCTGCAGTCCACAGCGAGATCTGAGTGAAGCCGGC
CGTATACAACTCGGAAAGGGAAAGGTCAAATAAATAGAGACCGAGTCAAGAACGAACCGCGGGTGTGACCGAGTC
ATCACGTAGTTTTAGGTTCGTCTTCTTCAACACAGAGCGATGGTGGTTGTGTGGTTCGGGCATTGACTCCCTCTC

CAAACCAAACCAAACCAAAGCAAACCCAGAATTTACTTCTGTGCCTTTACTGTTCTTTTTCCTCCATGTCATACA
AAAGGAAACCTCTCAAGAATCTATAGCTTCCGATACAAATAGGCCAAACCGCGCGTTGTCTTGTTGTTTATATAT
ATATCTATATTCTGTTGTCCTTGAGTGGTTTGTGTTGCCTATGGGTTAGCTAATAACATCTATAAGACGATCAGA
TCCATCCTCCGTTCTTCTTCTTCTTCCTCATGAGTAATCCAAAGCTCGATGAGAAGTACACTTTGGCTTGTTGTT
CTTGTAGTGCTGGGGTTAATAGCTCGGCTGTAGAAGTTG

CDS

ATGAGCTTTGGGGGGTTATCAGACGGCAGATCAGGAGGCGCTCTAGCTTACGGCAACGCGGCCATGACCGCCGGA
GAGTTATCTCCGCCGCAGCTCATCTCCCAGAGGCTCCCAAACTCTGCCTTCATTTCTCCCCGCCTCTCCCTCGGC
CTGCAAACGAACGTGGATGGACAGATAGACATGAGCAGGATGGCAGGCGTCGGCGGTGGCAGTGACGTGGGTTCG
GCCAAGAGGAGCAAGGACGATGAGAATGAGAGCAGATCGGAGAGCGACAACTTGGAAGCCATATCCGGTGACGAC
GTCGATCAGGAGAATCCTCGCAAGAAAAAGAGACACCACCGCCATACTCCGCAACAGATCCAGGAACTCGAAGCT
CTGTTCAAGGATTGCCCCCACCCGGATGAGAAGCAAAGGCTGGAACTGAGCAGGAGGCTATGCTTGGAGAGCAGA
CAAGTCAAGTTCTGGTTCCAGAATCGACGCACCCAAATGAAGACGCAAATCGAGCGCTTCGAGAACACGAAGCTC
CGGCAGGAAAACGACAAGATGAGGGCGGCGAACATGGCGATTCGAGAAGCAACGCAGAGCGCGGCTTGCAAGAAC
TGCGGAGGTCCGGTCATGCTCGGCAATGTCTCGGTGGAACGGCTGCAACTTAGACTGGAGAACGCTCGGTTAAAG
GAAGAGCTCGACCGGCTGTGCGCAATCGCCAAGAAGTTTCTGGGGAAGTCCGTCTCCTCTTTGGCCGGCCACATC
TCTCCGAACATGCCGAGCTCGGTTTTGGAGATTGGAGTCGGGAACAATATCTTCGCGGGGTTCAACTCCGTCGTC
GCTCCGACTATTTCCGCTTTGCCCGACTATTTGCCGGGGTTCAGCAGCAACCCACTGGATGCTGTCGCTCCGACC
ATGGGCAAGAACATGGAGGCGCTGCTGGGGAGCGACAGGAGCATGCTCTTGGAGCTTGCATTGGCTGCAATGGAA
GAGCTAATGAGCATGGCGCAGATGGAGGAGCCACTTTGGCTTGCGAGCTCACAAGGTGGTCCCAAGGCACTGAAC
TACGAGCAGTACCGCCGGACGTTTCGCCCCATCGCCGGGATATCGCCGGTTGGGTCTGTGTCAGAGGCCTCGAGG
GAGACGAGGATAGTGTCCATTGACAGCGTCGCCCTCGTCGAGACCCTAATGGATGCGAGTCGTTGGGTTCATATG
TTCCCAAGTATCGTGGCCAAGGCCACCACCACGGAGTTGATATCCAGCGGCGTGAGTGGAACAAGAGACGGCGCC
CTCCAACTCATGCAGGCGGAGCTTCACGTCCTCTCCCCGCTGGTGGCAGTGCGCGAGGTGAGCTTTCTCAGGTTC
TGCAAGCAACACGCGGAGGGCGTTTGGGCCGTGGTCGATGTCGCCGTTGACGGCATCAGAGCAGATCTCTCTTCT
GCTAACTGCAGGAGGCTGCCGTCTGGTTGCCTGGTGCAAGACATGCCGGATGGCTACTCCAAGGTCACCTGGGTG
GAGCACGCGGAGTACGACGAAGGCCAAGTGCATCATCTGTATCGCCCGCTGCTCCGCTCCGGCCAGGCGTTCGGC
GCCGGACGCTGGGTTGCCACCCTACAGCGCCAGTGCGAGTGCCTTGCCACCCTCGTGTCCTCCACCGCCGTGGCC
CGCGACGAGACTGCATCGATAACGGCGAGCGGGCAGAGGAGCATGTTGAAGCTGGCGCGTCGGATGACCAACGCC
TTCTGCGCCGGGGTCTGCGCGTCGCCCGCGCATGGGTGGAGCAAGCTGGCGTCGGAGACCATCGGGGAGGACGTG
CGGGTGATGTCGAGGATGAGCGTGAACGTACCGGGTGAGCCGGCGGGGTTGGTGCTCAGCGTGGCCACATCGGTG
TGGATCCCCGCGCCGCCCAAGCGGCTGTTCGACTTCCTATGCGAGACCAGATTCCGCAGCAAGTGGGACATCCTC
TCCAACGGCGGGCCGATGTGCGAGATGGCGCACATCGCCAAAGGCCAGGAAGCAGCCAACCGCGTCTCCCTCATA
TGGGCCACTGCCACGAACGCCAGCCAAACCAGCATGATGCTACTGCAGGAGACCTACACCGACGCTTCCGGCGCA
ATGCTGGTGTACGCGCCGGTGGACATCCCAGCCATGCACCTAGTGATGAACGGCGGCGACTCCGCCCACGTCGCC
CTCCTCCCTTCCGGTTTCGCCATCGTCCCCGATGGCGCCGGCTACCGAGGAGAGCTCACCGAAGAGCACCACAGC
GACGGCGCCGCCACCGCCGGCTCCGGTTCGCTGCTGACCATCGGGTTCCAGATTCTGGTGAACGGCCAGCCGACG
GAAAAGCTTACGGCGGAGACGGTGGACACCGTCAACGGCCTCATCTCATGCACGGTTCAAAAGATCAAGGCTGCA
GTCCACAGCGAGATCTGA

GSMUA_Achr8P27340_001 (*MaHDZIV14*)

GGTGGCAAACTCATTCGAGGCATTGTTATCAAGGGGATAAAACCAAAGAAGCATATAGAGAGAGATAGATTTCAT
GGATGCGATGCGTTTTCATTAAGGGTTTGCAACTAAAGTAGGTAACATTTAGTGATTCACCTAATGCATGTCACT
GGCTGGTCAGCACTACAAGTTATAACACCAAGATAAAAGTAACCTAAATGACTCATATGACTGCAAATGCCTTGT
TTTTATCGACAGGATCCATCCAAGTGATTCCAACTACTATAACATGTAAAAGAAATAGAATCATTAAGCTCAAAT
GTTCAATGATGCTCTCTGTACATCTTCTCTGTTTTCTAGTTTCACAGGGGAGTACAGCAGCAAACATGGCTTCAA
CACTAACCCTGACATGGAAAAGGACTAGACTAGTAAAACCATTTGCTGTTAATCATTGTTTCAAAGCAATCCTGA
TGTGATTAACAGAAGCCCTGAAAGCATGACTGCTCATCATTCTTTCAGTGTACAGCTGACTCCAGTGGACACAGC
ATAACTGCTCCAGTGGAAGACAATTTCATAATGTTTATTAGTAAGTGACTCGATAGTAGCTTTCTCAAGATAACA
AGCTGGCTGAAGGGACTGATTAATAGGTGATGCATTTGCACTGGCTGAAGGGACACAGCATAAATGCTGATGAGG
GGTGCTGCATTTGCATCAACTCTACACTTAATTACCTTCACCGAACAGGAAGACATCCCTGTCTCAAGTAGGCTT
TCTGTTCTTGTGCAGGTGGATCAGACATTATCTGAAATACTGCTGTGAATCAGATGAATTTCTATTCAAATATCC
AAACAACCAGTCAAGCTCATGACTCCCTTAATCCTTAGAACAATTCTCTTAACAGTCCATCCTACTGACGAATCT
AGGAAAACTTGTCTCCAGGAATTCATGCTTCCTTACGGCCGCTTTACATTGCCACCACGTAAAGCTCGGCCACCA
GACATTCTAGTCAGCATGCCAGCTTTGACTCAATAGATTTTGATTTGTAATATCAAAAATTCATCCAACTGGTTA
ATACGAAAAATTATTCACAAAAATGGAAGAGCAAAGATGGTTGTCAAAAGCCCTCAGCTTTGGTAAGAATGTTCA

AGAACTTGGACTCTCCAAATGGGCATTAAGAAGAATTGATAAAAAAGAATAACACAAGCCAATGAAATCTTAGAG
AAATGGGAGTTTTCTCATCAAAGAGAAAACTATCATACATAACTGTCATTCAAATGCTCATTCCTATGACACAAC
TATCATACATAACTCTCCAAATGCTCATTCCTATGACACAACTATCATACATAACTGTCATTCAATCCTAACAGT
CCACCTCAATTACACCTTTTTATGTCAGACCTTCCTTTGTTTTGTACCATTTATTCCTCATTCTATGGTTCAACA
ACCTCCCTCTTCAACTTTCCCTATTAACACCATTCACTATCTCACATTCTCCTCCAACTTCATTCCAGACTTTGA
ATGCATCAAAGCAGCATGAACAACAACAATACTCCAGTGAAAGACTTCTTTGCATCCCCAGCTCTCTCCCTCTCT
CTGGTAAGTATTATATCCTTTGCTTTTAGGGATAGAAATTTATGACAAACATGCATTTAGATTTTTTCCTATTAA
TTTGTGTCGTCGTTTCCCGAAATTACATTAGGCAGGTGTGTTCAGGAATAATGCAGCAGCAGTTGTGGATGTGGA
AGAAGGGGATGAGGCGAGTAAAGGAGGCTGTCAAAGGGAGCAGGCAGAGATCAGTGGAGAGAACTCCGGGCCAGC
AGGTCGGTCAGACGAAGACAGGGAGTCCAATGAATCACAGGAAGAGAATAGAGAGGTTGGAAACAACAGGAAGAG
AAAGAAGTACCATAGGCACACAGCTGAACAGATCAGAGAAATGGAAGCGTGAGTTGGTTTGGTCATATTGTTTTT
ATTTCAGACATGCTACCAGCACTAATGCATTACTTAGATGCCCGAGTCATATTGAGATACCGCTTTTTATATGTT
TAAAATGACATATTATGAATTTTAAAGATGTCATTCTGAATCATGTAAGATTGGCCATTTATTTTCCAGAAAGCA
GTACCCAGACCAGTCAAACATCAGAAAAATATACCAGATAATCCAGATAGCAGGAACTATGCACAGTAAATAGAA
TGAACATCTAGGATAAACATGCTGGTCAAATTTGGATCAGGAATATAGACCAGAAAACAGAGCTAATAATGACTG
TCATAAGTGAAGTCAAACAATCAATTCTGACATTGTAAATGGTCAAAGCAGGCTGTTCAAGGAATCACCCCATCC
AGATGAGAAGCAGAGGCAGCAATTGAGCAACCAGCTAGGACTTTCTGCCAGGCAAGTCAAGTTCTGGTTCCAAAA
TCGACGAACCCAAATCAAGGTAGCCATTTGAAAAACTAGCCTGTCATGAAATTTATATGCTAATTTGATCAGTCA
TCGCAAGGAATCAGTCCAGAGTACAAATACTTTGAAGGATAGTAAATTTACGTTACGTACCAGTTACATTTATTA
TATCAACTTATGATCATTGGACCATCATTTCACTGCTCTGATGCTTAATGGGTGTAAGCTCCCCTCAGGCAGTGC
AGGAGCGGCATGAGAACTCGCTACGTAAGTCTGAGATAGAGAAACTGCAGGAGGAAAACCGGACCATGAGGGAGA
AAATCAAAAAAGGATGCTGTCCTAACTGTGGTTATACAACCTTGAGCAATGGTACCACCATAACCACAGAGGAGC
AACAACATCATATAGAAAATACCAGGCTAAAAGCAGAGGTACGTCATGTGCCAATGGCTCATCATCAGAGTACCT
AATTTGTTCTCATGCTAAAGCTTACATTTGACTTCAGCTATAGAATTTTCCACATTTTGACCATCTATACGTTTT
AACTTTTGCTGTTTCTACCAAAATAACTTTGATCTTCAACAGATAAAAAAATTACGTCGAATGCTAGGAAGCATC
CCAGATGGAAACACTTCACCAAGTTCATCATGCTCAGCAGGAGCTGATCAAAACAAGAGCTCATTAGATAGCTGC
AGTAGGTTCTTGGGTCCTGAGAAATTTAGAATTTTAGAGATTGTCAATGTTGCCCTGGAAGAGCTGACCAAGATG
GCTACTGCCCAGGAGCCTTTGTGGGTTCGGAGTGTTGAGACTGGAAGGGAGATACTGAACTATGATGAGTATGTT
AAGGAATTCTCACCTGATATGTCAAGGAATGGATGTGTCAGAAACATTGAGGCTTCTAGAGAGACTGGTATTGTC
TTCTTTGACATGCCAAGGCTTGTACAAGCATTCATGGATGTGGTAAGGTTCATACATATACATAGAAACAAGTAC
ATTTATGACAATCCAATTTTGTCATTAAGAACTATCATATTGAAATTCCTGAAATTTTCTTGTCTCCAAATTGTA
CTGACAGATTGCTGAACATCAAACATAAATTGATAATTATATACTTTGACCGTTCACTAGTCACTCTTTTAGCAC
TTTCTTGTTGCCAACTACCTACTTATTGCACATGAAAATTGTCATACACTCAAACAAAAGCTAATGAAAGGATTA
TAACAAAATTATACTCTTAAATTTGCATTTGCATGGGTTAATATACTTAAAAGTGGCATCTATATCCTCTAACTC
TGACAAAATTAACAATAAATCTATATAGCGTCATATGATACATATATGTATCATTCTAATTTCTGTTTGTTCCTT
TATTGTTGTAGAACCAATGGAAGGAATTTTTCCCTTGCCTGATCTCCAAGGCTGTCATTGTTGATATCATTTCCA
AGGGTCTCGGTGATAGCAAAGATGGCACCATACAACTGGTAAGCACCCTTAACCATCATAATTGAATCAGCAATA
TAAGTCTCCTCATCTCGACAATATTTTTATCATAAATTCGACTTGCCAAGACTAGCTTTGTTACGAGGACCATCA
TTACCCAACAGATGTACTGGAGTTCTTTCTGACTAGAGTTGCTGTGATGGTACAATCTACAGAAAACAGGCAATA
ATTCTCATAACAACTCATTAAATTTCTCAGATGTTTGCAGAAATTCAGATGCTTACTCCTTTGGTGCCGACACGA
GAAATCTACTTTGTGAGATATTGCAAAAAACTATGCCCCACTAGATGGGCAATTTTAGATATATCTATTGACAAA
CTTGAAGAAAACATAGATGCATCACTCATGAAGTGCAGGAAGCGGCCCTCAGGTTGCATAATAGAGGACCAAGAC
ACTGGTCATTGCAAGGTAACTCAGTTATCTTATAGTTGTTTCCCTCTTCATTGTGGCAGTTTGACAACCTTCCTC
TCAAAGAATGCCATACTCATTAACAGTAGACACTTTTAAGATAAAATTGCAAGGGCATTTGGTTATACCAATGGG
ATTATCACTTTAAAAAGGTACACAACTACATCATTCTAAGTCATTATATTTCATTCTTTGTGTCTAGGTGATTTG
GGTAGAACATATGGAATGCCAAAAGACTGTAGTTCCAACACTATATCACCCAATTGTTACCAGTGGTCTGGCTTT
TGGGGCTCGACACTGGATGGCAACATTACGTCTGCAATGTGAAAGATCAGTTTTCTTCATGGCAACTAATGTTCC
TACAAGAGACTGCAATGGTTGTAGCAATTCTATCACCCATTCTATGAACATGAACCTATTGCTTACCTCACATAG
GATTTTCTTAGGGGTCTCGACACTGGCCGGAAGAAAGAGCATCCTGAAGCTTGGACAAAGAATGACTTCGTGCTT
TTGCCAGAACATTGGTGCATCAGGACACCACAAATGGACCAAGGTGTCAACAAAAGGTGGTGATGAGATTCGATT
CACGTCAAGGAAGAATATCAATGACCCCGGGGAGCCTCTGGGACTGATCATTTGTTCGGTTTTGTCGACATGGTT
GCCTGTTCCTGCAATGTCTCTGTTTAATTTCTTGAGGGATGACTCAAGAAGAACTGAAGTAAGACATTTTTTATT
ACCCCTTTTTGTCATATTTTCCTATAGACTGAATGAAACTTCTTTTTGAGTTTTCAGTGGGACATCATGCTTACA

CCAAGCCCTACTCAAACAATGGTGAATTTAGTAAAGGGCCAAGATCGTGGAAACTCTGTTACTATCTATGTAAGTTTTAGTTTTATCATTTTGTTGATATGATTCACAATCACTGATTGAAAAAAAAAACAAAACAAAACACTTCAATCGCCCAGTTTCAGATAAAATATTAAATGCCCAAATATAAAAATATTAATACAATATTTTAAACATATTAACCCATGTCATTACCTGTACAGAGCCTACAGACAACAACTTCATCAGAGAGGACCAACATTTGGGTTCTCCAAGATTGTAGCACCAACTCTTACGAGTCAATGGTGGTTTTTGCTCCTGTGGAGATTGATGGCACACAATCTGTGATGAACGGGTGTGACTCGAGTAGCTTGGCCATATTGCCATCAGGATTTTCTATACTTCCAGACGGACTAGAGACCAGGCCTCTTGTGATCACATCTAGGCCACAGGAGAGGACAATGGAAGGAGGATCTTTGCTTACGGTAGCATTCCAAATTCTTGCGGATGCGTCACCTGTGGCAAGGCCAACAACAGAATCGGTGGAGACCATAAACACACTTGTTTCGTGCACATTGCAAAATATAAAAAAAGCCTTGCAGTGTGAAGATGGGTAATTTGTGTGGGATAAGCAAATATGTGAAGATTGATGTAAATAGGTTTCAACCAATAGTGGGAGGAACCACATGTGCAACTCTTTGTTGTTAACAGAGGAAGAGCATAGGAATCGGTTTGAATTAACAACACGAATAACTAATCTGCACTGGAAGAATTTAGATCAGATGATCCAGCTTCAAAATTGGATCTGGTGCAGAAGGTATAAAGAAACGTAACATGTAGCAATGCTTGCCAATGTCAGTGAGTCATCTTTCTAGGTTCTGGAGGTGGATGGAAGTGTTGCATGATATCGAATAATAAATTCCTTGGTTTTCGGACTCATAATTGATGAGATGCACTTAGACCGCAAGACTTTGAAGCTTCATTGATAATAAGGATGGTACTAGAGCTGGTTTTCCTCCCTCCTCTCAATCCTCTGAATGAGAAAAGGAGGTGGCACAGGAAAACAACAGTTCCAAGAGAAGGAACTAGGCGGGTAGTTCTCTCAGATAAAAGATGG

CDS

ATGCATCAAAGCAGCATGAACAACAACAATACTCCAGTGAAAGACTTCTTTGCATCCCCAGCTCTCTCCCTCTCTCTGGCAGGTGTGTTCAGGAATAATGCAGCAGCAGTTGTGGATGTGGAAGAAGGGGATGAGGCGAGTAAAGGAGGCTGTCAAAGGGAGCAGGCAGAGATCAGTGGAGAGAACTCCGGGCCAGCAGGTCGGTCAGACGAAGACAGGGAGTCCAATGAATCACAGGAAGAGAATAGAGAGGTTGGAAACAACAGGAAGAGAAAGAAGTACCATAGGCACACAGCTGAACAGATCAGAGAAATGGAAGCGCTGTTCAAGGAATCACCCCATCCAGATGAGAAGCAGAGGCAGCAATTGAGCAACCAGCTAGGACTTTCTGCCAGGCAAGTCAAGTTCTGGTTCCAAAATCGACGAACCCAAATCAAGGCAGTGCAGGAGCGGCATGAGAACTCGCTACGTAAGTCTGAGATAGAGAAACTGCAGGAGGAAAACCGGACCATGAGGGAGAAAATCAAAAAAGGATGCTGTCCTAACTGTGGTTATACAACCTTGAGCAATGGTACCACCATAACCACAGAGGAGCAACAACATCATATAGAAAATACCAGGCTAAAAGCAGAGATAAAAAAATTACGTCGAATGCTAGGAAGCATCCCAGATGGAAACACTTCACCAAGTTCATCATGCTCAGCAGGAGCTGATCAAAACAAGAGCTCATTAGATAGCTGCAGTAGGTTCTTGGGTCCTGAGAAATTTAGAATTTTAGAGATTGTCAATGTTGCCCTGGAAGAGCTGACCAAGATGGCTACTGCCCAGGAGCCTTTGTGGGTTCGGAGTGTTGAGACTGGAAGGGAGATACTGAACTATGATGAGTATGTTAAGGAATTCTCACCTGATATGTCAAGGAATGGATGTGTCAGAAACATTGAGGCTTCTAGAGAGACTGGTATTGTCTTCTTTGACATGCCAAGGCTTGTACAAGCATTCATGGATGTGAACCAATGGAAGGAATTTTTCCCTTGCCTGATCTCCAAGGCTGTCATTGTTGATATCATTTCCAAGGGTCTCGGTGATAGCAAAGATGGCACCATACAACTGATGTTTGCAGAAATTCAGATGCTTACTCCTTTGGTGCCGACACGAGAAATCTACTTTGTGAGATATTGCAAAAAACTATGCCCCACTAGATGGGCAATTTTAGATATATCTATTGACAAACTTGAAGAAAACATAGATGCATCACTCATGAAGTGCAGGAAGCGGCCCTCAGGTTGCATAATAGAGGACCAAGACACTGGTCATTGCAAGGTAACTCAGTTATCTTATAGTTGTTTCCCTCTTCATTGTGGCATTCCAACACTATATCACCCAATTGTTACCAGTGGTCTGGCTTTTGGGGCTCGACACTGGATGGCAACATTACGTCTGCAATGTGAAAGATCAGTTTTCTTCATGGCAACTAATGTTCCTACAAGAGACTGCAATGGGGTCTCGACACTGGCCGGAAGAAAGAGCATCCTGAAGCTTGGACAAAGAATGACTTCGTGCTTTTGCCAGAACATTGGTGCATCAGGACACCACAAATGGACCAAGGTGTCAACAAAAGGTGGTGATGAGATTCGATTCACGTCAAGGAAGAATATCAATGACCCCGGGGAGCCTCTGGGACTGATCATTTGTTCGGTTTTGTCGACATGGTTGCCTGTTCCTGCAATGTCTCTGTTTAATTTCTTGAGGGATGACTCAAGAAGAACTGAATGGGACATCATGCTTACACCAAGCCCTACTCAAACAATGGTGAATTTAGTAAAGGGCCAAGATCGTGGAAACTCTGTTACTATCTATAGCCTACAGACAACAACTTCATCAGAGAGGACCAACATTTGGGTTCTCCAAGATTGTAGCACCAACTCTTACGAGTCAATGGTGGTTTTTGCTCCTGTGGAGATTGATGGCACACAATCTGTGATGAACGGGTGTGACTCGAGTAGCTTGGCCATATTGCCATCAGGATTTTCTATACTTCCAGACGGACTAGAGACCAGGCCTCTTGTGATCACATCTAGGCCACAGGAGAGGACAATGGAAGGAGGATCTTTGCTTACGGTAGCATTCCAAATTCTTGCGGATGCGTCACCTGTGGCAAGGCCAACAACAGAATCGGTGGAGACCATAAACACACTTGTTTCGTGCACATTGCAAAATATAAAAAAAGCCTTGCAGTGTGAAGATGGGTAA

GSMUA_Achr1P18760_001 (*MaHDZIV1*)

CTCGGTTTTAGTGCGTACAGGCGCTTCTCTTCTGTTTTAATCCTGGTGTCGGTTTTGGACTTCCTTTTCTTTTGATCTTTTACGATGATGAGATAGATAGATAGATAGAAAGAGAGAGAGAGAGAGAGAGAGAGAGAGTTGTGGGGGTCAAATGAATGAATTGCTGTAGTTGGTATTGTGTTTGTGAGGTGCCGTAGAGTCCACATTTCCGGTTTTTACATTGGGGTCGTCGTCTCCGCTGCTGACATTTCTGAAGCCCTTACCGGGTTTGCTTCCGTGGTATTGTGTCCTTCTTCTGTCTCTAAGGACGATGCAGGGGGAGAGCCAAAGATCCAAATTTTCGCCCTTTTTTACGTTAAAAATGGGATTTTGAGGTTGGGATTGGAATTATTGGAGTGAGTCAGGGTTGTTAGGGTGATCGTAGGAGCAGCGGGGAAGAAGGGGAAGAGAAAGAAAGGCTTAGTTTCCTTCTCTTTCTTTCGACCCTTTATCGGGAAAGAGGGAGGCGGCATTCTTTTACCGTAA

AGCCAGTGCCGTTTTGCCTTCTCTGATCTCTCCATTTCCGCTGCTTTCCTTTATTTCGTACAGTATATTCATTAT
ATCTGTCTTTTCTCCTTCTTTTACCATACTCGATATTGCTGCATCATTGAAATTGATTGAAATACCCAGAAAATT
TTGATTCTGGGAGGAGAAGGCGAAAGATCTGATCTCAGAGTGAAGATTATGAGAGCACAGCCAAGCAAGGAGGAT
CGACTCTGGAGGGACACAAGCAGCGAGAAAAAGGAAGGATCTTTGATGAGCTTTGGGGGCTTATCAGACGGCAGA
TCGGGCGCTCTACCCTACGGCAAAGCTGCCATGACTACCGGAGAGTTATCTCCGCCGGAATTCATCTCCCAGAGG
CTCCCTAAACCCTCTTTCGTGTCTCCTGGCCTCTCCCTTGGCCTGGTAAAGAAAACCGATCCCTGTATCATGTTG
CTTCTCGTCGGCTTTCGTTCCTGACTTCCCTTTCTTTGCGTCGTCGGACCAACCGTGGATGGACAGAGCGATACG
AGCCGGATGGCGGCCGTCGGCGGTGGCAGTGACGTGGATTCGGCCAAAAGGAACAAGGAGGACGAGAACGATAGC
CGATCGGAGAGCGACAACTTGGAAGCGATATCCGGAGACGACACCGATCAGGTGAATCCTCGCAAGAAGAAGCGA
TACCATCGACATACCCCGCTACAGATCCAGGAACTCGAAGCGTAAGTCCGATCCCTGAATCCTTCCGTGATCTTC
TGCTGCTTTGTCTTTCTCCTTGTCGCCTCAATTTGGTTGCACTTTACGTGATGTAGCTTGTTCAAGGAGTGTCCT
CACCCGGACGAGAAGCAAAGGCTGGAGCTCAGCAAGAGGCTTAACTTGGAGACCCGACAGGTCAAGTTCTGGTTC
CAGAACAGACGCACCCAAATGAAGGTAGCAACCCTCTGATGCCACGGAATTTTCGTTTGGATCGTCGGATTCTGC
TCTCGTTTTGAGTTCGCTGTTTTGATGAGTTGTAGACGCAAATCGAGCGTCACGAGAATTCGATCCTCCGGCAAG
AGAACGATAAGCTGAGGGCGGAGAACATGGCGATCCGGGAAGCGGCGAGGAACCCGGTTTGCAACAGCTGCGGCG
GCCCGGCGATGCTGGCCGACATCTCCATGGAAGAACAGCAACTTAGAATCGAGAACGCCCGTCTCAAAGAAGAGC
TCGACCGGGTTTGTGCTCTCGCCGGCAAGTTCCTTGGCAAGTCCATCTCTTCCTTGTCGGAACCCATCTCTCCGT
CCATGCGGAGCTCGGTCTTGGAGCTCGGAGTCGGGAACAACGGCTTCCTTGGGTTCGGCTCGGTGGCTCCTACCA
TCTCCGCGGTGCCCGATTTCCTACCGGGGATCGACAACCCACTGGGTAAGAACATGGACGCACTGCTGGGGAGCG
AGAGGAGCATGCATTTGGAGCTAGCACTGGTTGCAATGGAGGAGCTCGTGAACATGGCGCAGATGGAGGAGCCGC
TTTGGGTTCCGAGCTCGGATGGCGGCACTGAAACACTGAACCATGAGCAGTACCTCCGCACCTTTCGCCGGATCT
CGGGGACGGGGATGCCGCCTGTGGGGTACGCTTCCGAGGCCTCAAGGCAGAAAGGGGTGGTGATCATCAACAGCC
TCGCCCTTGTCGAGACCCTCATGGACGCCGTAAGCATGACCTTCTTCTCATCCAAGTAAATGGATGCTTCGTGCC
ACTGCAACCTGAAACCTTTGATCTTTCTCGCAGCGTCGCTGGGCTGACATGTTCCCATGTATCATCGCAAAAGCA
ACGACGATGGAAGTAATATCCGGTGGCGTGGGTGGAACCAGAGACGGCGCTCTTCAGCTTGTGAGCCGAAAAGCC
TAACCCTTACTAGTTTGATGGATTCTGATACAAAGAGCCATTGGTTTCTGATCTCAAGGTTTTGCACATGGTTCA
GATGCACGCGGAGTTGCAGGTTCTCTCCCCGTTGGTTGCAGTCCGGGAAGTGTACTTCCTCAGGTTCTGCAAGCA
GCATGCTGAAGGCGTTTGGGCTGTGGTTGACGTCTCCGTGGACAGCATCAGAGCAAGCTTCTCTTCTGCGAGCTG
CAGGAGGCTGCCTTCTGGCTGTGTGGTGCAAGACATGCCCAGTGGTTACTCCAAGGTAACCCTCTCTCTCTCTCT
CTCTCTCTCTCGCTCGCTTATAATTTAGTGTGTATTCCTTCAGTATAGTATTAGTTTGGATGCTGCATGTCCCTC
TGGACTTGTTCGTGAGCGAGATTTTTGGCACTGTTCTTGCTATTTCGCTTCTCCCAGTCCATATCATATCCATCT
TAGATGCCAAGTTACGTACTTTTCGAAGTTTGAATGCGTGAAGTCGAGGTCCTGCACGAATAATAAATGAGAATT
ATGAAAGCAATAGATATCTTAAGATGGCATTCCCCCACCCCCCGACGAATTAACATGATGGACTGAGGAAGGTAC
ATGATGTGCATGATTAAGCAGGGCGACATGGAGGCGGAGCTCTCATTGTTTTGGAACGGCTGTGGTGTGTAGGTG
ACTTGGGTGGAACACGCGGAGTACGAGGAGGGCCAAGTGCATCAGCTGTATCGCCCGCTGCTCCGCTCCGGTCTG
GCGTTCGGTGCCGGACGCTGGGTAGCCACCCTCCAGCGCCAGTGCGAGGGCCTCGCCATCCTCATGTCCTCCGCC
ATCACCGCCCGCGACGAGACTGGTAATGAATCGGCCGCGCGCGCTCCATCTCTGATACGTAATTGGATTCATTCA
TTCATCCTCACGGACGGTTTGTTGGATTCGAACTGGATAGAGACGATCACGGCGAGCGGGAGGAGGAGCATGCTG
AAGCTGGCGCAGCGGATGACCAACGCCTTCTGCGCCGGGGTGTGCACGTCGTCCGCGCAGGAGTGGAGCAAGCTG
GCGACGGAGAACGTGGGGGACGAGGTGCGGGTGATGACGAGGATGAGCGTGAACGAGCCGGGGGAGCCGGCGGGC
GTGGTGCTGAGCGCGGCGACGTCGGTGTGGATCCCCGAGTCCCCCAAACGGCTCTTCGACTTCCTTCGCGAAGCC
AGCTTCCGCAGCAAGTGGGACATCCTCTCCAACGGCGCGCCGATGTACGAGATGGCGCACATCGCCAAAGGCCAG
GACGCAGGCAACGCCGTCTCCCTCCTCCGCGCCAGCGTACGATACCCCCTTCCCTCTTCCTCCCCACCCTTAACC
CTCCTCAACCTTCACGCCATGCTGATACTGCTGACGCGCAGGCCGCGAGCTCCAACCAAACCAGCATGCTGATAC
TGCAGGAGACCTGCACCGACGCGTCCGGCGCCATGGTGGTGTACGCGCCGGTGGACATCCCAGCCATGCACCTCG
TCATGAGCGGCGGCGACTCCGCCTACGTCGCCCTTCTCCCCTCCGGCTTCGCCATCCTCCCCGACCGTTCCGTCG
AGCCGCACCAGACCAGCGGCAGCCTCGGCTCGCTGCTGACCGTGGGGTTCCAGATCCTGGTGAACAGCCAGCCGA
CGGCAAAGCTGACGATGGAGTCGGTGGAGACCGTCAACGGCCTCATCTCCTGCACCGTCCAGAAGATCAAGGCTG
CACTCCAGTGCGACGACTGAATCCCCCCCCACCCCCGTGTACAACCCGTAAAGAGACGGAGAAATAAACAGAGGC
CGAGTCAAGAACGAACCGCGCGTGCGTGTGGCACATCCTTTGCTATGTGATAGAAGATGAAGTAATCACACCCAG
CAGGTTTGTCTTTTAACACCGAGCAACGGTGGTGGTTCGGGCATTGACTCCGTCTCCAAATCTGTGTCCAGTGAT
CGACAGCCGCCTCCGCATACATTGTGGTGACATACGTACTATGTTATTCAGTAGTGCTTTGGTATATATGTTGTC
GTCGTCATCGAGCGACTCACGTAGATTGCCTGCCCATCAGCAGCTCAGTATTGCTTGTTTTTGTGGCCTTACATT

TCCATGCTCGATCCCTGCGCTATCTTGCTCTCTGCTGCGTGTTGCTCCGCTGGAGACGATGGCCCATCTAGCTTG
GCCATGCCACGCACACAGAGAGAGAGAGAGAGAGAGAGAGGTGGATGCTGGCAGCCAGCGGAGCGCATTA

CDS

ATGGCGGCCGTCGGCGGTGGCAGTGACGTGGATTCGGCCAAAAGGAACAAGGAGGACGAGAACGATAGCCGATCG
GAGAGCGACAACTTGGAAGCGATATCCGGAGACGACACCGATCAGGTGAATCCTCGCAAGAAGAAGCGATACCAT
CGACATACCCCGCTACAGATCCAGGAACTCGAAGCCTTGTTCAAGGAGTGTCCTCACCCGGACGAGAAGCAAAGG
CTGGAGCTCAGCAAGAGGCTTAACTTGGAGACCCGACAGGTCAAGTTCTGGTTCCAGAACAGACGCACCCAAATG
AAGACGCAAATCGAGCGTCACGAGAATTCGATCCTCCGGCAAGAGAACGATAAGCTGAGGGCGGAGAACATGGCG
ATCCGGGAAGCGGCGAGGAACCCGGTTTGCAACAGCTGCGGCGGCCCGGCGATGCTGGCCGACATCTCCATGGAA
GAACAGCAACTTAGAATCGAGAACGCCCGTCTCAAAGAAGAGCTCGACCGGGTTTGTGCTCTCGCCGGCAAGTTC
CTTGGCAAGTCCATCTCTTCCTTGTCGGAACCCATCTCTCCGTCCATGCGGAGCTCGGTCTTGGAGCTCGGAGTC
GGGAACAACGGCTTCCTTGGGTTCGGCTCGGTGGCTCCTACCATCTCCGCGGTGCCCGATTTCCTACCGGGGATC
GACAACCCACTGGGTAAGAACATGGACGCACTGCTGGGGAGCGAGAGGAGCATGCATTTGGAGCTAGCACTGGTT
GCAATGGAGGAGCTCGTGAACATGGCGCAGATGGAGGAGCCGCTTTGGGTTCCGAGCTCGGATGGCGGCACTGAA
ACACTGAACCATGAGCAGTACCTCCGCACCTTTCGCCGGATCTCGGGGACGGGGATGCCGCCTGTGGGGTACGCT
TCCGAGGCCTCAAGGCAGAAAGGGGTGGTGATCATCAACAGCCTCGCCCTTGTCGAGACCCTCATGGACGCCCGT
CGCTGGGCTGACATGTTCCCATGTATCATCGCAAAAGCAACGACGATGGAAGTAATATCCGGTGGCGTGGGTGGA
ACCAGAGACGGCGCTCTTCAGCTTATGCACGCGGAGTTGCAGGTTCTCTCCCCGTTGGTTGCAGTCCGGGAAGTG
TACTTCCTCAGGTTCTGCAAGCAGCATGCTGAAGGCGTTTGGGCTGTGGTTGACGTCTCCGTGGACAGCATCAGA
GCAAGCTTCTCTTCTGCGAGCTGCAGGAGGCTGCCTTCTGGCTGTGTGGTGCAAGACATGCCCAGTGGTTACTCC
AAGGTGACTTGGGTGGAACACGCGGAGTACGAGGAGGGCCAAGTGCATCAGCTGTATCGCCCGCTGCTCCGCTCC
GGTCTGGCGTTCGGTGCCGGACGCTGGGTAGCCACCCTCCAGCGCCAGTGCGAGGGCCTCGCCATCCTCATGTCC
TCCGCCATCACCGCCCGCGACGAGACTGAGACGATCACGGCGAGCGGGAGGAGGAGCATGCTGAAGCTGGCGCAG
CGGATGACCAACGCCTTCTGCGCCGGGGTGTGCACGTCGTCCGCGCAGGAGTGGAGCAAGCTGGCGACGGAGAAC
GTGGGGGACGAGGTGCGGGTGATGACGAGGATGAGCGTGAACGAGCCGGGGGAGCCGGCGGGCGTGGTGCTGAGC
GCGGCGACGTCGGTGTGGATCCCCGAGTCCCCCAAACGGCTCTTCGACTTCCTTCGCGAAGCCAGCTTCCGCAGC
AAGTGGGACATCCTCTCCAACGGCGCGCCGATGTACGAGATGGCGCACATCGCCAAAGGCCAGGACGCAGGCAAC
GCCGTCTCCCTCCTCCGCGCCAGCGCCGCGAGCTCCAACCAAACCAGCATGCTGATACTGCAGGAGACCTGCACC
GACGCGTCCGGCGCCATGGTGGTGTACGCGCCGGTGGACATCCCAGCCATGCACCTCGTCATGAGCGGCGGCGAC
TCCGCCTACGTCGCCCTTCTCCCCTCCGGCTTCGCCATCCTCCCCGACCGTTCCGTCGAGCCGCACCAGACCAGC
GGCAGCCTCGGCTCGCTGCTGACCGTGGGGTTCCAGATCCTGGTGAACAGCCAGCCGACGGCAAAGCTGACGATG
GAGTCGGTGGAGACCGTCAACGGCCTCATCTCCTGCACCGTCCAGAAGATCAAGGCTGCACTCCAGTGCGACGAC
TGA

GSMUA_Achr3P15400_001 (*MaHDZIV4*)

ACCTCGTGTTATCCCATTTTGATTCAGATTCAAATACAACATCCTACCAGTTGTTGCAGGAAAAGAAAGAGACAC
CTGAAGGCAAAACATTGCAGATGTTATCTCCACAAAAAAAAAAGAAAAAAAAAAACATTATTGTGAGGAATTGTG
ATCACCAGCAAAATATCCTGAGAAAGCAGGAGCATGAGAACATTATGTAAATAATCCAATTAAAAGATGCACATG
CACGCATATTCTTAATTTCTTTAATAAAAGTTCCATGGGTTTCTAAATTCTGACTAGCAAAAAATAAGGGTTTCT
TGGTGGCTGAGTCATTAGAGCCATCGTTATTAAGAAACAAAGCCACAGAGGCACAAAAAAAAGGAGATATGGAGA
TCATGGATGCATTATTTTGGTCCAAGTAATTCCAAATTCTTTGATATATAAAAGAAATATATCATTAAACTCAGA
TGTTCACTATTCCAACACTAACAATGATGAGAAAGACATGGAAAAGGAACGAGTCCTAGTAAAAAGCTTTTGCTA
TTAAATTTTCTTCTCTTTCAAAACAATGCACAAGTGATTTGGTTTAAGCACGAGTGCTGCCATGGAAGTGGCCGC
GGAGACTGATTACTGGTTGTTGATGAGGGCGATGCATTTGAATCAACCCTGCAATTGACCACCTGGACCGGACAG
AAACATGTCCCTGTTTCAAGAAAAAGCTTCCTGTTCTTATGCAGGTGGAACAGACATGCGCAGAAACACCATTGT
GAATCAGATGAATTCATATTCTCACCCCTAGACAACCACCATTCAAGCTCATGACACCCTTAATCCTTCAAAACG
ATGCCTGCATCCCACCATCTTCCTCAAAATTCCAGATGTACTAGTCTCCAGGAATTCATGTTTCTCCAAGGCCTT
CTTTACGTCTAAAGTTCGGCCACCAAACATTCTTATCTCCATGCTCGCTTTGACTCGATTCGAGACCGATTTGGA
ACATCAAGGATTCACCCATCTAATTATTTAACAGGAAAGTTTACTCATCAAGAAAAAAGAGCACACGGAGAAGTA
TCAAATAGATAATAACAATTGTTTGCTTTGAAGACGCAGCATGGACGGATAGAATCATAGGGAAATGGGAGTCTT
CACTTCACAGGTAAGGGACAATAGTTTCTGCAAATTTACATTCCAGATTGGTAAAAGTGAAGGATGGATCATATG
GTAGCATGGCTACCGAAGGCCTGCATGGATGACTCTCTTTAAAGGCACAGTTGCCAGCCATGTAAAGATAGTGTT
GTTTCCTATGACACACATTGCATACATAACTGTCATTCAATCCTAACAGTCCTCCATTACATCTTTCCATTGCAG
AGTCTCTCCCTTTGTTTTGTATCATTTATTCCTCATTCCATATTTCAGCTACCTCCCTCCTCAACTTTCCCTCTT

AACACCATTCCATATCTCCATTCCCCTCCAACTTAATCTGGGGGCTTTGCATGCATGAGAAAACACCTAAGCAGC
ATGAACAACAACAACATTCCTCCTGTCAAAGACTTCTTTGCATCCCCAGCGCTCTCCCTCTCTCTAGTAACTACT
TATTTCTTTGCATCCTTACCGCTCTCCCTCTGTTCGATCATGAATTTAACATTTTTCCTTCCTGAAAATATGATA
GGCAGGTGTATTCAGGAACAATGCAGCGGCGGCAGCAGCCGAGGTGGAGGAAGGGGACGAGGGGAGTGGTGGAGG
GGGTCAAGGGGAGCAGGCAGGGATCAGCGGCGAGAACTCTGGGGCAGCGGGTCGATCCGAGGAAGACAGAGAGTC
CAATGAATCACAGGATGACAACAGAGAGGTCGGAAACAAGAGGAAGAGGAAGAAGTATCATAGGCACACAGCTGA
ACAGATCAGAGAAATGGAGGCGTGAGTTGGTTTGCTCGACTTGATCCTCCCTCCTGTCTTTGTGACATACTAGGA
GTTTCTACATACTCATGCATGTCTCGGTTTCTGCCAGGTCATAATGGTATATTGCTTTTTGCCATGTCTAAAGCT
TCATATTGTGAATTTTAAAGATATTATTCTAAGTAGTGTAAAACCGGTCATTTGATTTCAGAAAAAGAATTGCCC
AGACCACGACCCACTCATGTTCCTTCAACTCTAAATCAGAATAAAGCACATAACCATAGAGAGTGTAGTCGATCA
TCAAAAGGATACCAGGTAGCAGGAGCTTTGTGCAGTCCAAGAAAAACCATTATCTAGGAACTGATCTGTTCCTTT
CTGTGATAAAATCATACTGGGTAAACAGGAACATGTACCGAATAAAAAACTAATGATGACTACCATTAAGTGTAG
TTAACCAATCAAATCTAATATAAGGAATGGGCAACACAGGTTGTTCAAGGAGTCACCCCATCCAGACGAGAAGCA
GAGGCAGCAGTTGAGCAAGCAGCTAGGCCTTTCTGCCAGGCAGGTCAAGTTCTGGTTTCAAAATCGACGAACCCA
GATCAAGGTACCCATTCAAAGAACTAGCCTGTCTCGAAAATTATATGCCAAATGGATCAGTTGCTTAAAGAAATG
AGATCAGAGCACAAATAATTTTAAGAAGACTAGTTTTGCAATCACATACCAGTTTCATTTGCTCTCGCATCTTTT
GAACATTGAACTATCATGAAACTGTAAGTTCCCCACAGGCGGTGCAGGAGCGGCATGAGAACTCTCTACTGAAAT
CTGAGATAGAGAAACTGCAGGAGGAAAACCGTGCCATGAGAGAGACGATAAAGAAAGCGTGCTGTCCCAATTGTG
GTATTGCAACCTTAAGCAAGGACACCACCATGACCACAGAGGAACAACAACTTCGTATCGAAAATGCCAGGCTAA
AAGCAGAGGTATGGCTTATGACCCAATGGCTCATTATCTGACTAGCTAATCTGTACTCTTGCTAGAAATAACATT
TTGACTTTTGCTACAGATTTCCCCGTATTTTTATACCTATTGAACATTTCAACTATTGCTATTTCTACTGCAATA
ACTTTGATCTTCAATCCAACTCCTAGATAGAAAAACTACGTCGAATGCAAGGAAGCATCCCGGATGGAAACACTT
CACCAAGTTCATCATGCTCTGCAGGCGCCGATCAAAACAAGAGCTCATTAGATTGCTATAGTGGATTCTTGGGTC
TTGAGAAATCTAAAATTTTAGAGATCGTCAACGTTGCCCTGGATGAGCTGATCAAGATGGCTACTGCCCAGGGAC
CCTTGTGGGTTCGAAGTGTTGAGACCGGAAGGGAGATACTTAACTATGATGAGTATGTTAAAGAATTCTCACCCG
ATAATTCAAGGAATGGATGTCTCAGGAATATTGAGGCTTCCAGAGAGACTGGTGTTGTCTTCTTTGACATGACAA
GGCTCGTACAAGCCTTCATGGATGTGGTAAAGTTCATATGTCTACATGCATATCAGTTTCTATCCTATTGAAATT
CCTGGAACTTTCTTGTTTGCATATATTTACAGATAGCTGGATATCAAACATAACTAAGTCATTATAAACTTTGAA
TTTTTATTGTCCATTCTTATAGCACTTTCTCGCTGCCAGTCACCCATGAAGATATCAAACATCAAACAGAAGCTA
ATTAGCAAATTAGAACAGAATTGCACTCCTAAATTGGTATTTGCATGACATCGGCATATTTAGCCTTTTACTTCT
GACCAAATTAACAATAAATCTTAATGATGACTATTTAGATACCGGTACATAACCTTCTAATCTCTGTTTGTTCCT
TTCTTGTTGTAGAACCAATGGAAGGACTTATTTCCTTGTATGATCTCCAAGGCTGCCATAGTTGATGTCATTTTC
AATGGACAGGCTGACAGTAAAGATGGTACCTTACAACTGGTAAGCTCCCTTAACCAGCATGACTGAATCAGTAAG
GAAGGCCTCTTCACTTCGACATTGTTTTCATCATGAACTCGACCAATCAAGTTTATCTTATCACAGGGGTCATCA
GTACCCCATAGATGTCCTATTCAACTAAGTTCTTGCTGACTAGAGTATCTGTGACTATTCAATGTACATAATACA
GGTAGTAGTTCTCATAGAAACTCATTAAATTTTCTCAGATGTTTGCAGAAATTCAGATGCTTACACCTTTGGTGC
CAACAAGAGAAATCTACTTTGTGAGGTACTGCAAGAAACTAAGCCCTAGTAGGTGGGCAATTTTAGACATATCCA
TCGACAAACTTGAAGAAAACATAGATGCATCACTCATGAAGTGTAGGAAGCGCCCATCAGGCTGTATAATAGAGG
ACCAAGACAATGGTCACTGCAAGGTATTTCAAAATCTTATAGTTGTTTCCCTCTTATACGCAGCAGTTCTGCAAC
CTTCCTCTCAAGATTGAGATGCTCATTATCTTAAGATGAAATTGTAAGATCATTTGGTTATACACAACTGCATCA
TCTGCAAGTCATCATATTTCATTCTCTGAATCTAGGTGATTTGGGTAGAACACATGGAATGCCAAAAAAGTGTAA
TTCCAACACTATTTCGCTCGATTGTTACCAATGGCCTGGCTTTTGGGGCTAGACACTGGATGGCAACACTACGAC
TGCAATGTGAAAGATCAGTTTCTTCATGGCCACTAATGTTCCCACGAGAGACTGTAATGGTACGTAGCAATTCT
TTTACCTGTTAGGAAAATGAACTTATTACTTACCTCGCATGAGATTCTCAGGAGTCTCCACACTGGCTGGAAGAA
AGAGCATCCTGAAGCTTGGACAAAGAATGACTTCAATCTTTTGCCAGAACATCGGTGCATCAGGACACCGCACAT
GGACCAAGGTATCGACAAAGAGTGGAGATGAAATTCGATTCACATCAAGGAAGAATATGAATGATCCTGGTGAGC
CTCCAGGACTGATCATCTGTGCAGTTCTGTCGACGTGGCTGCCTGTTCCTACAATGACTCTATTTGATTTCTTGA
GAGATGAATCAAGAAGAGCTGAAGTAAGATATTCTTCATCACTCCTTTTTGTCATATTTGCTCTGCTAGATAGTA
AACAAACCTCCTTTTTGTTGTTCAGTGGGACATCATGCTTACACCAGGCCCTACTCAAACAACGGTGAATTTAGC
AAAGGGCCAAGATCGTGGAAACTCTGTTACGATGCATGTAAGTATAAGTATTCAACAATCTGTTGATATGATTCA
TAATTAGCAGTCGAAAAACCAAAGAACTTTAATCCCTAAGTTTCCAGTAGAATATTAAATTAACAAAAATAGAAA
TACAAAATAAAATTCAAAACATACTAGGCCAGTGATCAAATTGACATACAACATTTCTTTTTAAAGATGTAAAAG
TTGAGATGTTTAATATTTGATGTGCATACACAGACAACAACTTCATCAGAGAGGACTAACATTTGGGTTCTTCAA

GATAGCAGCACTAACGCTTATGAGTCGATGGTGGTTTTTGCTCCTGTGGACATTGATGGCATGCAATCAGTGATG
ACTGGGTGCGATTCTAGTAGCTTGGCTATATTGCCATCAGGATTTTCTATACTTCCTGATGGGCTTGAGACCAGG
CCTCTCGTGATCACATCTAGGCCAGAGGAGAGGACGATGGAAGGAGGATCTTTGCTTACAATAGCATTCCAAATT
CTTGCCAATTCCTCACCCATGGCGAAGCTAACAATGGAATCAGTGGAAACTGTGAACACACTTGTTTCATGCACA
CTGCAGAATATCAAGAAAGCACTGGGCTGTGAAGATGGGTAACTTTTGTGGCCTAAGCAAATTTGTGAAGATGAT
GGAAACAGATTCAAATCACTAATCGGAGGAATCAGACATGCACAAACACGGGAAGAGTAAGTAAAATATAGAAAT
CAATATAGATTGGTAAGAATTAACAACATAGTCAGAAAGCTTGCACTGGAAGAATTCAGATCAGATGATCTAGGT
TGAAAAGCAGGCCTGTGCAGAAGGTATAAAAGAATGTAACATGTAGCAAGTTCTTCCAATGCCAGTGAGTCTTCA
CAAAGCATTTCTTTTGGCCTTTTGGAGGGGACCGTGTCACATGATATTGATATAAGCTAATGAGTTCTACAATTA
TTGAACTCATAACCTAATGAGATGTATTTAACCGCAAGAGTTTGAAGCTTCGCTGATGGTAAGGATGGCACTAAA
GTTGGCTTTTCTCCCTCCCATCAATCAATCCTCTGAAAGGGAGGTGGAAGAGACCAAGAACAGGCAACTAAAAGT
AGTTCCTTTACAAAGGT

CDS
ATGAACAACAACAACATTCCTCCTGTCAAAGACTTCTTTGCATCCCCAGCGCTCTCCCTCTCTCTAGCAGGTGTA
TTCAGGAACAATGCAGCGGCGGCAGCAGCCGAGGTGGAGGAAGGGGACGAGGGGAGTGGTGGAGGGGGTCAAGGG
GAGCAGGCAGGGATCAGCGGCGAGAACTCTGGGGCAGCGGGTCGATCCGAGGAAGACAGAGAGTCCAATGAATCA
CAGGATGACAACAGAGAGGTCGGAAACAAGAGGAAGAGGAAGAAGTATCATAGGCACACAGCTGAACAGATCAGA
GAAATGGAGGCGTTGTTCAAGGAGTCACCCCATCCAGACGAGAAGCAGAGGCAGCAGTTGAGCAAGCAGCTAGGC
CTTTCTGCCAGGCAGGTCAAGTTCTGGTTTCAAAATCGACGAACCCAGATCAAGGCGGTGCAGGAGCGGCATGAG
AACTCTCTACTGAAATCTGAGATAGAGAAACTGCAGGAGGAAAACCGTGCCATGAGAGAGACGATAAAGAAAGCG
TGCTGTCCCAATTGTGGTATTGCAACCTTAAGCAAGGACACCACCATGACCACAGAGGAACAACAACTTCGTATC
GAAAATGCCAGGCTAAAAGCAGAGATAGAAAAACTACGTCGAATGCAAGGAAGCATCCCGGATGGAAACACTTCA
CCAAGTTCATCATGCTCTGCAGGCGCCGATCAAAACAAGAGCTCATTAGATTGCTATAGTGGATTCTTGGGTCTT
GAGAAATCTAAAATTTTAGAGATCGTCAACGTTGCCCTGGATGAGCTGATCAAGATGGCTACTGCCCAGGGACCC
TTGTGGGTTCGAAGTGTTGAGACCGGAAGGGAGATACTTAACTATGATGAGTATGTTAAAGAATTCTCACCCGAT
AATTCAAGGAATGGATGTCTCAGGAATATTGAGGCTTCCAGAGAGACTGGTGTTGTCTTCTTTGACATGACAAGG
CTCGTACAAGCCTTCATGGATGTGAACCAATGGAAGGACTTATTTCCTTGTATGATCTCCAAGGCTGCCATAGTT
GATGTCATTTTCAATGGACAGGCTGACAGTAAAGATGGTACCTTACAACTGATGTTTGCAGAAATTCAGATGCTT
ACACCTTTGGTGCCAACAAGAGAAATCTACTTTGTGAGGTACTGCAAGAAACTAAGCCCTAGTAGGTGGGCAATT
TTAGACATATCCATCGACAAACTTGAAGAAAACATAGATGCATCACTCATGAAGTGTAGGAAGCGCCCATCAGGC
TGTATAATAGAGGACCAAGACAATGGTCACTGCAAGGTGATTTGGGTAGAACACATGGAATGCCAAAAAAGTGTA
ATTCCAACACTATTTCGCTCGATTGTTACCAATGGCCTGGCTTTTGGGGCTAGACACTGGATGGCAACACTACGA
CTGCAATGTGAAAGATCAGTTTTCTTCATGGCCACTAATGTTCCCACGAGAGACTGTAATGGAGTCTCCACACTG
GCTGGAAGAAAGAGCATCCTGAAGCTTGGACAAAGAATGACTTCAATCTTTTGCCAGAACATCGGTGCATCAGGA
CACCGCACATGGACCAAGGTATCGACAAAGAGTGGAGATGAAATTCGATTCACATCAAGGAAGAATATGAATGAT
CCTGGTGAGCCTCCAGGACTGATCATCTGTGCAGTTCTGTCGACGTGGCTGCCTGTTCCTACAATGACTCTATTT
GATTTCTTGAGAGATGAATCAAGAAGAGCTGAATGGGACATCATGCTTACACCAGGCCCTACTCAAACAACGGTG
AATTTAGCAAAGGGCCAAGATCGTGGAAACTCTGTTACGATGCATACAACAACTTCATCAGAGAGGACTAACATT
TGGGTTCTTCAAGATAGCAGCACTAACGCTTATGAGTCGATGGTGGTTTTTGCTCCTGTGGACATTGATGGCATG
CAATCAGTGATGACTGGGTGCGATTCTAGTAGCTTGGCTATATTGCCATCAGGATTTTCTATACTTCCTGATGGG
CTTGAGACCAGGCCTCTCGTGATCACATCTAGGCCAGAGGAGAGGACGATGGAAGGAGGATCTTTGCTTACAATA
GCATTCCAAATTCTTGCCAATTCCTCACCCATGGCGAAGCTAACAATGGAATCAGTGGAAACTGTGAACACACTT
GTTTCATGCACACTGCAGAATATCAAGAAAGCACTGGGCTGTGAAGATGGGTAA

GSMUA_Achr11P25820_001 (*MaHDZIV20*)

CCATCTCTCTCTCTCTCTCTCTCTCTCTCTCTCTCTCTCTCTTCTGTCTAAGAAGCAAAGAAGAACTTGTCATTTAC
ATGTACATGCGTATGCGAACGCGGCATGAACGCATTAAAGCCTTCGAGTTCGCTTCTCCTCTATCTTCTCTCTCC
TTCACACTATCGCAACCCAAGCAGTGGCGATCAGAGCCATTAAATGCGAGGCGTTGGAGGGGAAGAGAGGAGGGC
TCCTCACAGGAGAAATTGAAGACAGGGAATAGGGCACAAGCTCATTTAATAGGGGGAGTTGGGGAGGTGTTTCTC
AGTGGGAAAATGAGAAAACCCAGTCAACCCCACCCCACCACTCCGCCATCATGAAGGTTGGAGATCTTAGGGTTT
GTGCCATGGTTCAGCATCGCTCTGCCCTGACAGGAAGAGCCTTGGCTCACTCCTAGGGCAACCCAACCGATGATG
AAGAAACTCTGTTGGCTCTCCATTTCGCCAATGGTCAATTCTATGAACATCTCTAGTTAATGGTGAGAACAAGTG
AACGAAAAGTTTATCCTCTGAATGAGAACAAACAATAATCTCCAAAATGAAACTGTTATTTCCAAGTCATTCGGT
GTGCAGATAGAACTCTTTGCATGACATGCAAGTAATCTTTTGGGGGCACAGATTTGCAAAGCAAAGAGAGAAAAA
GTATGATGAATGCATACGTAGGTCCAAATATTCTTCTGTTTCATTACCAGAGTATCGTTATTCTTAGGACTGCAC
ACATCCAACAAGAAAACTACTAACAGCAAATGATACAGTATGTTACATGTCTTAAACTTAGTAATCTCTATAGGT

TGGTATATCCACCAATCGTCTCTTTCACCTTATACTCAACCAGTGGAAAAAAATTTAAAGCACACATTTGCTCTC
TCTCTCTCTCTCGCTTTCTTTCTCTCTCTACCCTAATAACACCATCCTCTCAATAAATGCAATTGCACCATCT
CAATGCGCTCTGCAATAACTCATCCTCATCACTTCCTCTCCACCTGTTCCCTTTTCTCCTTCTTCCCTATCCCTT
GCCCTCCTCTTGTCCAGATCTCCTTCCCGTCCCTGATCTAATGGCTTCTCTCCCGGTGCTTTCTTCCCTGAAGCC
ACCTCTTCCTCTTCCTCGCATGCACTAAGTCCTGCTCTCTTCTGTTTGCTCTCTTCCTGACGTAGAAAACAAAGG
AGGAGGAGAGAGAGAGAGAGAAGAGAGAGCGTCCATGTCCTATGACTAGATCTCAGTGATATTGGCTACTCACAT
TCCATCTATCCCTTGGTTGTCACGTTTCTTGATGTTAAAAAGCCATTTCTCTCTGCTCTCTCTCCTATTTTTTTT
CTGAAACTTGTGACTTTGGAGACCCAAGTTGAGTCCAATCCGCAGTCTGCTAGTAAGACACCTTTTTATCATCTT
CATCGAGATTGAGAGACATATATACACAAAGGGAGAGAGAAAGAGAAGTGGAGGGGGGGAAGAATTAGGGTTTGA
ATGCCTGTGGGGCTCATGATACCAGCAAGGCAACCACCATCCATGATCGGGAGGAACACCGGCACCGGTTATGGC
TCGTCGTCGGTGTTGTCGCTCAGCCAGGTCAGCAGATCAGTACTCTTTCTTCTTGCACATGTTGTTGTGGTAAGA
GCTTTGTCGAGGTCTGTAAAGATAAGGTGAGTAGTGAAAGGATTACAGTTGCACAAGTTTGAGTCCTTTTGTTCG
TTTTCCACTTAATCTTTGTCTTCCAGTTCTTTAAATCTTTCTATCCTTCGTCTCTTCAACAGGAGATAGGAAAAA
GAGACTCTCTCTCTCTACAGAATACATCAACCAGTTTTGTGTTTGTTCATCCCCTCTCACCAATCTTCTTATATG
CACTTGTCCACACTTGATCTACAGAAACACCTTCGGACATCAAACTTTGCTTAACGATTGATCGACGGCACTCCT
TTATCCCAACCTCCTTTCTATTTCACCTTTGCACCTTTTCCAAATTCATCACACTGGTCACAACACTCAGATTGA
TTGATTGAATTCAATTTCTTGTCGAAATACGAGGACTGAAGGAGCCTTTGTGGCTGCTTTTGTAGCCAAACTTGT
TAGAAGGGCAGCAAATCCCACTCCAGCACCAACATCATAACCAGTTTGTGGAGATCGCACAGGCCACCACCGCAG
AGAGCGAGATGGCAAGGGCTCGAGAGGACGACTTCGAGAGCAAGTCATGCAGCGAGAACATCGAGGGCGCTTCCG
GCGATGATCAGGACCAAAACCAGCGCCCTAGGAAGAAACGCTACCATCGCCACACACAGCATCAAATCCAAGAAA
TGGAGGCGTAATTCTCTCATCACTCTCCTTGCACAAGATACCAACACAAAGGACAGTTACTGAGAAGATTAAAGC
AACAAGACCTTGTCTTTTGATTGTTTTCTTTCTCAATCACTTCCTTATGCCAATTCTTTTATGTTAATTGTTGTT
TCTCTGTCCTGTTCTTGACATATTGCACTCACATTTCTTGCAGTTTCTTCAAGGAATGCCCTCACCCGGATGACA
AGCAGAGAAATGAGCTGAGCCGACAGCTTGGGCTGGAACCTCTCCAAGTCAAGTTTTGGTTCCAGAACAAGCGCA
CCCAAATGAAGGTGTTTTCCTACGACTTCAATAACCATTAAGGACATTTACTTAGCTCACACTGCATGTCGGCCA
TTGACCACGTATTTTCTTGGCCCATTATCCTATTCCCACACGTTATTGCCATCGTCTTAGTCTCTTATCACTTGC
TGCTTAACACTGCTCTTAAACTTCTCCTCCCGTTGTCATAGAATCAACACGAGCGGCAGGAGAACTCTCAGCTAC
GAGCCGATAACGAGAAGCTTCGTGCCGACAACTTAAGGTACAAGGAGGCCCTCAGCAATGCTTCATGCCCAAACT
GTGGCGGGCCTGCTGCTCTCGGAGAAATGTCCTTCGACGAACACAACCTCAGGATCGAAAATGTTCGGCTGAGAG
AAGAAGTAACTGCTCTTCACCCCCCCATTATGAGATTTATGATGCTTTGCGGTAGGTATGTGAATTGCAACTGCT
CTTCCCATGCTTTGACAGATCGATAGGATATCAGGGATAGCAGCTAAATACGTGGGTAAGCCAGTGGCGTCGTAC
CCGCTCCTTTCTCCCGCCATCCCTTCACGTTCACCATTGGACCTCGGCGTCGGAGGTTTGGGGGTGCAACAGGGG
ATTGGGAGTGAGATGTTCGGAGCCGGGGAGTTACTGAGGAGCGTGTCAGGGCTGCCGGAGATCGAGAAGCCCGTG
GTCATCGAGCTCGCCGTGGCCGCCATGGAGGAGCTCATCAGGATGGCGCAGCTCAGTGAGCCACTCTGGATTCCG
GGTCTCGATGGCGCAGCCGAGACTCTCAACGAAGACGAGTACGTCAGAACGTTCCCCAGAGGGATTGGCCGAAA
CTTCTGGGGCTGAAGTCCGAGGCGTCGCGCGAGACTGCGGTGGTGATCATGAACCAGATGCATGTTGTCGAGATA
CTCATGGACGTGGTACGGGAAGTCGTGGTTTAACTAAGTAGGATGGATTACGAAGGGTTAAAGGTTTGCTCTTCT
GATGCTGGGATTTCATGTCTCTGTTCTCATAACTCTTTTGATGGTTTACAGAATCAATGGGCAAATGTGTTCTCG
GGCATTGTGTCGAGAGCATTAACACTCGAAGTATTATCGACTGGAGTGGCTGGCAATTACAATGGAGCTCTGCAA
GTGGTATATTTCTTTTGCCCAATGTTCTACCTTCACTTATTTTCTCGATCTATTTCCAAATTTCTCTTGAATTTA
AAGCGGTTTTAGTTCATACGTGTAAGATTGCATCTTCTTTGTGGCGAGACATAGTCTTATACGAAATCGTTTACC
TGGTAAACTGCATCTGCCATATTCCACTGAGCTCATGAAGAGCTGTGATCTGTTCTTCTTGATCGACGTTATACA
CTTCCGAAGGACTCACTCTGTGCTGAAGCATAATATCCTCCATCTTTGAATCCTCAGATGTCAGCAGAATTCCAA
GTGCCATCTCCGCTTGTTCCAACTCGGGAGAGCTTGTTCGTCAGGTACTGCAAGCAGCACGCGGATGGAACTTGG
GCGGTGGTTGATGTTTCCTTGGACAGCTTGCGCCCCAGCCCAGTCCTGCGATGCCGAAGAAGGCCATCCGGCTGC
CTGATTCAAGAAATGCCCAATGGCTACTCAAAGGTGAAGAACAATCAGGAAGATTAGAAATCCAAAGCTAACATG
TTGATCGACTGTTCTTAGGAGTTGTTTTCCCGTTCCACAGGTTACTTGGGTGGAACATGTCGAAGTGGACGATAG
GTCTGTGCATAATATCTACAGGCCCTTGGTGAACTCAGGTCTGGCATTTGGTGCAAAGAGGTGGGTCAGTACCTT
GGATAGGCAATGTGAGCGCCTAGCGAGTGTGATGGCTAGCAACATACCCTCTGGAGACATCGGCGGTAAAACTTT
CCGCTGCTCCCATTTCATATGATCATTGATATTGTCATTATGTGCGAGTTTCTAATCTTAGCCAATTCGATGTTC
TCTTCTTCTCCACGGAATATACGTACATACAGTGATCACTACTCCGGAAGGCAGGAAAAGCATGTTGAAGCTAGC
TGAGAGAATGGTGATAAGCTTCTGTGGCGGTGTCGGTGGCTCAGCTTCACATCAATGGACTACACTGTCCGGTAG
CGGTGCAGAGGATGTGAGGGTTATGACGAGAAAGAGCGTAGACGATCCTGGAAGGCCTCCTGGTATTGTTCTTAA

TGCGGCCACATCCTTCTGGCTTCCCGTCCCACCGAAGAGGGTGTTCGACTTCCTACGTGATGAAAGCTCTCGCAG
CGAGGTATGCCTCGTATGCTTCTGAGATCCGAACTATGCGAGCACACATCACTTCTTGGTTTTGTTCATGCATTT
ACAGTGGGATATCCTCTCAAATGGTGGTGGTGTTCAAGAAATGGCTCACATCGCTAATGGCCGAGACCATGGAAA
CTGTGTTTCCCTGCTACGCGTCAATGTGAGTGTATTAGTATTTGCAGTCGTATGTTGCTACACCATACAGACTGG
GTGATCGTTCTCTAAATTGCATGACGGGCGATCGCAGAGCACGAACTCAAGCCAGAGCAACATGCTGATACTGCA
AGAGAGCTGCACAGACTCAACGGGCTCCTACGTGATCTACGCCCCGGTGGATGTCATCGCCATGAACGTGGTGCT
TAACGGTGGCGATCCTGACTACGTCGCGCTCCTGCCGTCAGGTTTCGCCATCCTCCCGGACGGGCCATGTGGAGG
ACAAGGCGGCGAAATGGTGGACGGCGTCGGATCAGGCGGCTCCCTCTTGACTGTAGCATTTCAGATCCTGGTCGA
CTCGGCTCCGACGGCCAAGCTGTCTCTTGGATCAGTCGCAACAGTCAACAGCCTCATCGCATGCACTGTTGAACG
GATCAAGGCTTCAGCTGGAGGCGAAAGTGCCCACTGAGCCTGAAGAATACACAAGATATGCGTGAGTGACCTCTT
CCATCCGCATAATCCATGGTTTGTTGTCTTATTACATTCGTTGGTGCATGATGATGCTTTGATGTGCAGAAGAAA
GGGTGGAGTTTGGAGCATCAATTGTGGGAAGTCAAGAACGCACCTCAGACTTACCCTGCAGCGTAGTGTTGGTTT
GGCGGCCTGGGAGCAATAACCCGTAGGCACTCTCCACCACCACCATGCGAGGAAAGGTTGGTTCGGGCATTGACT
TCCCCTCGTTTCAGGACTACCACCTCTAGGATTTTGCTGGAATTTTACCCCTTTCTCTCTCTCTCTCTCTCTTTC
TCTCTCTCTCCATGGAACAAGCATATACTCTCTCATTTCCCACCATTTTATCTTGTGTTTTGTGTTGTTCTTGTG
GTGCTGGTTGTAAGATTCTGTGCTACGCCACCCTTTTAAGATGAAGAGCTTAAGTTGATTTGCTTTCATTTAGTT
CCTTCGTTTCAC

CDS

ATGCCTGTGGGGCTCATGATACCAGCAAGGCAACCACCATCCATGATCGGGAGGAACACCGGCACCGGTTATGGC
TCGTCGTCGGTGTTGTCGCTCAGCCAGCCAAACTTGTTAGAAGGGCAGCAAATCCCACTCCAGCACCAACATCAT
AACCAGTTTGTGGAGATCGCACAGGCCACCACCGCAGAGAGCGAGATGGCAAGGGCTCGAGAGGACGACTTCGAG
AGCAAGTCATGCAGCGAGAACATCGAGGGCGCTTCCGGCGATGATCAGGACCAAAACCAGCGCCCTAGGAAGAAA
CGCTACCATCGCCACACACAGCATCAAATCCAAGAAATGGAGGCTTTCTTCAAGGAATGCCCTCACCCGGATGAC
AAGCAGAGAAATGAGCTGAGCCGACAGCTTGGGCTGGAACCTCTCCAAGTCAAGTTTTGGTTCCAGAACAAGCGC
ACCCAAATGAAGAATCAACACGAGCGGCAGGAGAACTCTCAGCTACGAGCCGATAACGAGAAGCTTCGTGCCGAC
AACTTAAGGTACAAGGAGGCCCTCAGCAATGCTTCATGCCCAAACTGTGGCGGGCCTGCTGCTCTCGGAGAAATG
TCCTTCGACGAACACAACCTCAGGATCGAAAATGTTCGGCTGAGAGAAGAAATCGATAGGATATCAGGGATAGCA
GCTAAATACGTGGGTAAGCCAGTGGCGTCGTACCCGCTCCTTTCTCCCGCCATCCCTTCACGTTCACCATTGGAC
CTCGGCGTCGGAGGTTTGGGGGTGCAACAGGGGATTGGGAGTGAGATGTTCGGAGCCGGGGAGTTACTGAGGAGC
GTGTCAGGGCTGCCGGAGATCGAGAAGCCCGTGGTCATCGAGCTCGCCGTGGCCGCCATGGAGGAGCTCATCAGG
ATGGCGCAGCTCAGTGAGCCACTCTGGATTCCGGGTCTCGATGGCGCAGCCGAGACTCTCAACGAAGACGAGTAC
GTCAGAACGTTCCCCAGAGGGATTGGGCCGAAACTTCTGGGGCTGAAGTCCGAGGCGTCGCGCGAGACTGCGGTG
GTGATCATGAACCAGATGCATGTTGTCGAGATACTCATGGACGTGAATCAATGGGCAAATGTGTTCTCGGGCATT
GTGTCGAGAGCATTAACACTCGAAGTATTATCGACTGGAGTGGCTGGCAATTACAATGGAGCTCTGCAAGTGATG
TCAGCAGAATTCCAAGTGCCATCTCCGCTTGTTCCAACTCGGGAGAGCTTGTTCGTCAGGTACTGCAAGCAGCAC
GCGGATGGAACTTGGGCGGTGGTTGATGTTTCCTTGGACAGCTTGCGCCCCAGCCCAGTCCTGCGATGCCGAAGA
AGGCCATCCGGCTGCCTGATTCAAGAAATGCCCAATGGCTACTCAAAGGTTACTTGGGTGGAACATGTCGAAGTG
GACGATAGGTCTGTGCATAATATCTACAGGCCCTTGGTGAACTCAGGTCTGGCATTTGGTGCAAAGAGGTGGGTC
AGTACCTTGGATAGGCAATGTGAGCGCCTAGCGAGTGTGATGGCTAGCAACATACCCTCTGGAGACATCGGCGTG
ATCACTACTCCGGAAGGCAGGAAAAGCATGTTGAAGCTAGCTGAGAGAATGGTGATAAGCTTCTGTGGCGGTGTC
GGTGGCTCAGCTTCACATCAATGGACTACACTGTCCGGTAGCGGTGCAGAGGATGTGAGGGTTATGACGAGAAAG
AGCGTAGACGATCCTGGAAGGCCTCCTGGTATTGTTCTTAATGCGGCCACATCCTTCTGGCTTCCCGTCCCACCG
AAGAGGGTGTTCGACTTCCTACGTGATGAAAGCTCTCGCAGCGAGTGGGATATCCTCTCAAATGGTGGTGGTGTT
CAAGAAATGGCTCACATCGCTAATGGCCGAGACCATGGAAACTGTGTTTCCCTGCTACGCGTCAATAGCACGAAC
TCAAGCCAGAGCAACATGCTGATACTGCAAGAGAGCTGCACAGACTCAACGGGCTCCTACGTGATCTACGCCCCG
GTGGATGTCATCGCCATGAACGTGGTGCTTAACGGTGGCGATCCTGACTACGTCGCGCTCCTGCCGTCAGGTTTC
GCCATCCTCCCGGACGGGCCATGTGGAGGACAAGGCGGCGAAATGGTGGACGGCGTCGGATCAGGCGGCTCCCTC
TTGACTGTAGCATTTCAGATCCTGGTCGACTCGGCTCCGACGGCCAAGCTGTCTCTTGGATCAGTCGCAACAGT
CAACAGCCTCATCGCATGCACTGTTGAACGGATCAAGGCTTCAGCTGGAGGCGAAAGTGCCCACTGA

GSMUA_Achr2P15140_001 (MaHDZIV2)

CATTTAATTTATATTCTCACAGCCACTTGTCACAGGGCCCAAGATCTCGGTTGACTCCAGGTTTCTTTTGGTAAT
AATGATGATCTTCTCGCATTATTTGGTTGCTTGTAATTGCAATTGCAATTGAGATACAGGATGTGACAATTTGCA
GTGAGAGAGGAAAGAGGATATAGCATTTAAAGGTTTTTAATGGCATATACATATGCTACAAAGTGTTGGACCTCC
GGAAGCAATTATTAGCTGAGAAATAACTACATGTATTGAAGATCCCAAGCTGCTGGTGTCGAAGGGGAAGAAGAC

AGAGGGAGGGAGGGAGGGAGAGGGAAAGTTTTCAAAAGGAGGGGTCATGGCAACCTGAAGCTACTCACCATAAAT
TTGTGCACCAATGGTTTCAGCAGGAGGAAGAGAAGGTACCAGCACCTTGGCCATGACACCTCTGCTCAGCTGACA
TGGAACCTTGATTGCTAGCATTCAGTGCACGGGCGGAACACACCATCAGTGACGAAGCTTGAGTTCTTAGGCTAT
ATCTAGTGGAAAGCTAGAGGGGTGCAGATTACCATGCTGTCCTGCTCTCGGCGACTGTTTGCTGTGTTGACATGT
TATCAGCATCTTCTACTTGAGGGGAGGATAACAAAACACATGAGAGAGAAAAAGGAGGGTGAGAGCTGGCAGATT
AGTATCCTTAAGCTCTCGCTCTGTCTCTCTCCTTTGCTGTCCCATCTTTAATTCTGGAAGCTGTACTATACCAGA
AGACAACCAAACCATATCAGAGTGAAGTCATTGCCCCCAACTAAAACATACAGCACAAAAGGAAGAGGCCAAAGC
CATGCAGAACCCAACGACAGAAGAAAGAAAACCTTTTCATGTATATTTCTGCTAAGCTTTATGTCTCTGGTTTCA
GAAGATCACTTGGAAATGTACACAAGGAATTCAATGTTTAATCAAAATATAAATGCAAATATACTTGATCAGATG
TTTATATTGTCATCTGATTCGCTGATGATAATTGTTTGTAGATTCTGTTTGGAGAGGATACAATATACCTCAAAA
TCTAACATATTTAAAGGCTCGGCCTTTTAATTCGTGTCAGTTACTGTAAGCTTTAAATACGGAGAGAGAGAGAGA
AAGAGAGAGAGAGACGTGAATTCCGGTGCGTCTTAATAAGGGGAAGGGTGGATCGAATCAGCCCGGATCGCAGC
CGGCATCTGCGTCGCCACACCGCCGGTTACCTCATCTGGCTCCCGGCCGAACTCGGCACTCGGATAAGCCCCATA
GAGAGAGGAAGACGTGAGCCAGAGGAGGAGCGGTGTGAGGCGGAGAAGAAAGGCGCCACCTTTGAGGACGAAGCA
TGGAGAGGACGGCGTTGCCATGACTTGGAAATCGACAGCACGAACTGGCTCGCTTCTTTTGTAGACAGCTGTGGG
TTATCGTAGTGCTTGTTTTGGTGGTTGTAGTGTAAGCAGGAGTGGGGAGGGCGGGCAGCGTCGGAGTTGCGTAGG
ATGGATTCCGGCGACGAACAGGACGTTCCGAACTCACAGAGTAGGAAGAAGCGGTACCACCGGCACAACCCGCGG
CAGATTCAGGAACTCGAATCGTACGCACTGTTCTCGTCTATCTCCTTCTCACTCTATGTGTGTCCTGAGCGGTCA
TTGCGTAGTTTTGTACTATGGTTTGGGGATCTTCGTGGCGGCGCTCTCTGACGAGGTTTCTGTGAGCTTGTTGCT
TAGGTTGTTCAAGGTTTGTCTGCACCCGGACGAGAAGCAGAGGCTGCAGCTGAGCCGGGATCTGGGGCTGGAGCC
GCGGCAGATCAAGTTCTGGTTTCAGAACCGAAGGACGCAGATGAAGGCAACAGCTGCTCTCTTTTCCTCTTTTCG
TTAGCGTAATCGACGCGGAAAAAAAATTGGATTTTGGTGCGAGCAAAACCGAAAAGGCATCGCTGATTGATTCTG
GTGCTTGGCAGGGGCAACAAGAGAGGGCGGACAACTGCTTGCTCCGTGCCGAGAACGACAAGATCCGGTGCGAGA
ACATTGCCATGAGGGAGGCACTCAAGAACGTCATTTGCCCCTCCTGTGGCGGTCCTCCGCCCGATGACGACTCCT
ATTTTGACGAGCAGAAACTGCGGATGGACAATACAAGGCTGAAAGAGGAGGTAATACCGAGCTCGCTTTCGTTGT
GGTGTTTCACTGGTCATGATCAGTTCTCACTGGTGCAAGTCTCCCTTTTTAGTCACCTCGACCCTGCTATTTGTC
TTATGCAGTTGTTAATAGCTGTTAGGGGAATAATGTTTCCAAAGGGTACCAATTGAATCTTTGTCCGATTCTAGA
TTAAAGCAAGATCCATTTCCAGTTATGCAGAAACCAAAACATAAACATGTAATCGTAAGATCTATTTCTATGCTA
GGTCGTCATCTACTTGGTCATCTTTTCTCATTTTAGTGTTTGCAAGAGAAGAAGTAGAAGAAAAAGTTCGTACTA
GCCGTTGTGTTTTTATCACGACAGATAAAATCTTCAAACGATAACATTTGTTTAGTCAATTATATGCATATATTT
AACTATTTTGATTTGGAGGAAGCCGGTCATAATGGATCAACTGTTCAAAGATTAAAATTGTTAGAGCTCCTTTTA
GCAAAATTCATTGGCTGGCGTGGCATGAAAATGTGGATCAGAAGTAGCTATTGCTGTTTGGAAACTTTTGTTTCT
GAGGGCCATCGATCGATAACTCTATTTATTAGTTTTCCATTAGTTATCAGCATGTTCTGATCTGAATTCGGTGAT
AGAGACAACGAAGCCTTCTAGCTTGAAGTTAGCTATTTGTTCACTACTTCTTAGGCTTTATTGCAGAAAACTGAT
CATCGCTGCTTACATTTAACTAAGGTCAATGATGTTCATTCCCTTAACTTCATGTAATTTCCTTGCAGCTTGACC
GTGTTTCAAATCTTGCATCAAAGTACCTTGGAAGGCCTCTCACCCAGCTTCCCCCAGTTCAGCCAGTATCCTTAT
CATCATTAGACTTATCAGCTGGGGGTTATGGTGATGCAGGGATAAGCCCTTCCCTTGATCTTGATCTCCTGTGCC
GGAGTTCCTCTTCAGCTTTTCCATTTCAATGTCCCTCAACAGTTTCTGATCTTGAGAAACCTCTTATGGTGGAGA
TGGCTACCGGTGCAATGGAGGAGGTAATCAGGCTAGTGCAGACTGATGAGCCTCTTTGGGTGAAGTCAAGTGATG
GGAGGGACATACTTCAACTTGAAACCTACGACAGCATGTTCCAAAGGCTAGGTAGGCAGCTCAAGTTCCCAGGTA
CTCGAATTGAGGCATCAAGGGATTCAGCTCTTGTCATCATGGGTGCTATGACATTGATCGATATGTTCATGGATG
CGGTAAGTTTTGACCTTCCAGTAAAAAGAAATTACTCCTCATTCTATTTTGGACTGAGTTGGATTTTGTTGCAGA
GCAAGTGGGCAGAGTTGTTTCCCACAATTGTTTCTAAGGCAAGGACCATTGAAGTTCTTGCTGCTGGAATGGCTG
GAAGTAGAAGTGGGTCTTTGTTATTGGTAATGGCAGTTCTTAAGCCCTTTCAGTTGAACATAAATTTCCAAACTT
GCTATCATGCTGATATGTTCAACGGCATGCAGATGTATGAAGAGATACAGGTTCTTTCACCAGTTGTTCCTATGC
GTGAGTTCTGCTTTCTGCGGTACTGCCAGCAAATTGAGCCAGGCGTGTGGGTAGTAGCTGATGTTTCGGTGGACT
ATCCTAGAGACAATCGGCTTGCTCTTTCTTCGCGATCAAGGAGGCTTCCTTCTGGATACTTGATTGAGGAAATGC
CCAATGGCTATACTAAGGTGAGAGATCTGGTTTTTGTGCTTGCACAATGTTCTTAGTTTGCTGATTTCTCTCTCT
TAACCAAATTTGTTGGTGATTTGATGTAGTTAACTTGGGTTGAGCACATGGAAATCGAGGACAAGAATCCGATCC
ATATACTTTTCAGGGATTTAATAAACAGTGGAATGCTGTTTGGGGCACAGCGCTGGCTTGCCGCCCTACAGAGAA
TGTGCGAGAGGTTTGCTTGTTTGAATGTCGCTGGACTTCCAGCTAGAGACATCGGAGGTAACACGTGGTTTGCTC
AGTAGTTGCTATAGAGTACAAGAATCTGCAAATTGCTATTTGACTGTGATATCAACTATGGACTTCAGTGGCTCC
GTCGCCTGATGGCAAGAGGAGCATGATGAAGCTTGCACAGAGAATGCTGAGCAGTTTCTGTGCCAATGTTGGTGC
ATCAAATGGGCACCAATGGAACACCATCTCTGGGTTAAATGATGTCGGAGTCAGGGTTACAATTCACAAAACCAC
AGATGCTGGCCGGCCTGATGGCATCGTCCTCAATGCAGCAACCTCAATGTGGCTGCCGATATCATCTGAGAAGGT
CTTCGGTTTCTTCAAGGATGAACGAACACGATCTCAGGTTTCTCACATCTTCTTGAGATATCTATTCAAAGGACA
CTAGTCTTCTATCGTGTTCTCTTATTTTAATGGACACTTACTTTCTGTTTGTTGTTTTGTTCTTTCTTTCAGTGG
GATGTTCTCTCAAACGGCAACACTCTACAAGAGGTGGCTCACATCACAAATGGTTCACATCCGGGGAACTGTATT
TCTCTTCTTCGTGTATGCTCTTTTTCTCTTGTCCATTTATTCTTCTTACATTTAGACTTATGGCATATGATTTTT
ACAGAAGGCTCTTCTTTTTATAGAACCAATAACCAGAACACTTCATTTTTATCATCAATTCACATGTAATACAGT
GTAGTCCTGACTAATTTTGCTTGCAAATGAGTGCAGCTATTGGCATTATGAAGGTTGTATATGTTCAGAATAAAG

TATCCCTTGTCAGGTATTAATTTTCTGGTTTTGGTGGTGAATGAATAGGGACTCAATTCGGGCCAGAACACTATG
TTGATACTCCAAGAATGCTGCACCGATGCATATGGCTCTGTCATTGTTTATTCTCCTGTCGACCTACCCGCCATC
AACATTGTCATGAGCGGCGAGGACCCGTCCTACATTCCCATTTTGCCTTCAGGTTTCACCATTTTGCCAGACGGG
CGAGCTGCTGCGGGGGCTTCATCCAGCTCAAACCCAATGGTAGGCTCATCTGGCTCGTTGCTGACCGTCGCGTTT
CAAATTCTCATGAGCGGCTTGCCATCTGCAAAACTCAACTTGGAGTCGGTGATGACGGTCAACAACTTGATTGGC
ACCACGGTTCAGCAAATAAAGTCTGCCTTGAACTGTCCTGACATC**TGA**TGCAATGAGGAGAATTAAAAGCCATTT
TCTTCTATTGCCACCACCTTTTCCTTGCTGCTTCAGATCTGATGGGTTCTCGAGGCATTCAAGATGGGATTAATG
TGGTGTTCTTTTGTTGGTTGGTTACCCTTGATTGCTTTCATGGTGGGCTTGAGTTGAAACTTCAAGATGACTCTG
CTGCTGCTGCTGATGCCGGTCTATGGAGGATCCCATTTTTTGGCAGAAACCAGTGAGTTTTTACATTTACGAGCT
CTCAGTAAAGTCGAGAACAAGCCATATCTGCCTCTCCTTTTTGCTTGCTTCGATCAAATTCTTTCGTTTCTTAAT
GCATCGAGTGGTTATGGTTTGGCTATTGACTTCTATCAACAACACACTATTTGACGAGCATTTGTTTTGAAGACG
GTCTTGTTGGATTGTGGTTCGATGCATCTGTGATGACTTTGGCAACAAGTAGTGAGGAGCAGTGAGTTGGGTTAA
ACTCTTGAATGACAGAGTGCTC

CDS

ATGGATTCCGGCGACGAACAGGACGTTCCGAACTCACAGAGTAGGAAGAAGCGGTACCACCGGCACAACCCGCGG
CAGATTCAGGAACTCGAATCGTTGTTCAAGGTTTGTCTGCACCCGGACGAGAAGCAGAGGCTGCAGCTGAGCCGG
GATCTGGGGCTGGAGCCGCGGCAGATCAAGTTCTGGTTTCAGAACCGAAGGACGCAGATGAAGGCAACAGCTGCT
CTCTTTTCCTCTTTTCAGAGGGCGGACAACTGCTTGCTCCGTGCCGAGAACGACAAGATCCGGTGCGAGAACATT
GCCATGAGGGAGGCACTCAAGAACGTCATTTGCCCCTCCTGTGGCGGTCCTCCGCCCGATGACGACTCCTATTTT
GACGAGCAGAAACTGCGGATGGACAATACAAGGCTGAAAGAGGAGCTTGACCGTGTTTCAAATCTTGCATCAAAG
TACCTTGGAAGGCCTCTCACCCAGCTTCCCCCAGTTCAGCCAGTATCCTTATCATCATTAGACTTATCAGCTGGG
GGTTATGGTGATGCAGGGATAAGCCCTTCCCTTGATCTTGATCTCCTGTGCCGGAGTTCCTCTTCAGCTTTTCCA
TTTCAATGTCCCTCAACAGTTTCTGATCTTGAGAAACCTCTTATGGTGGAGATGGCTACCGGTGCAATGGAGGAG
GTAATCAGGCTAGTGCAGACTGATGAGCCTCTTTGGGTGAAGTCAAGTGATGGGAGGGACATACTTCAACTTGAA
ACCTACGACAGCATGTTCCAAAGGCTAGGTAGGCAGCTCAAGTTCCCAGGTACTCGAATTGAGGCATCAAGGGAT
TCAGCTCTTGTCATCATGGGTGCTATGACATTGATCGATATGTTCATGGATGCGAGCAAGTGGGCAGAGTTGTTT
CCCACAATTGTTTCTAAGGCAAGGACCATTGAAGTTCTTGCTGCTGGAATGGCTGGAAGTAGAAGTGGGTCTTTG
TTATTGATGTATGAAGAGATACAGGTTCTTTCACCAGTTGTTCCTATGCGTGAGTTCTGCTTTCTGCGGTACTGC
CAGCAAATTGAGCCAGGCGTGTGGGTAGTAGCTGATGTTTCGGTGGACTATCCTAGAGACAATCGGCTTGCTCTT
TCTTCGCGATCAAGGAGGCTTCCTTCTGGATACTTGATTGAGGAAATGCCCAATGGCTATACTAAGTTAACTTGG
GTTGAGCACATGGAAATCGAGGACAAGAATCCGATCCATATACTTTTCAGGGATTTAATAAACAGTGGAATGCTG
TTTGGGGCACAGCGCTGGCTTGCCGCCCTACAGAGAATGTGCGAGAGGTTTGCTTGTTTGAATGTCGCTGGACTT
CCAGCTAGAGACATCGGAGTGGCTCCGTCGCCTGATGGCAAGAGGAGCATGATGAAGCTTGCACAGAGAATGCTG
AGCAGTTTCTGTGCCAATGTTGGTGCATCAAATGGGCACCAATGGAACACCATCTCTGGGTTAAATGATGTCGGA
GTCAGGGTTACAATTCACAAAACCACAGATGCTGGCCGGCCTGATGGCATCGTCCTCAATGCAGCAACCTCAATG
TGGCTGCCGATATCATCTGAGAAGGTCTTCGGTTTCTTCAAGGATGAACGAACACGATCTCAGTGGGATGTTCTC
TCAAACGGCAACACTCTACAAGAGGTGGCTCACATCACAAATGGTTCACATCCGGGGAACTGTATTTCTCTTCTT
CGTGTATGCTCTTTTTCTCTTGGACTCAATTCGGGCCAGAACACTATGTTGATACTCCAAGAATGCTGCACCGAT
GCATATGGCTCTGTCATTGTTTATTCTCCTGTCGACCTACCCGCCATCAACATTGTCATGAGCGGCGAGGACCCG
TCCTACATTCCCATTTTGCCTTCAGGTTTCACCATTTTGCCAGACGGGCGAGCTGCTGCGGGGGCTTCATCCAGC
TCAAACCCAATGGTAGGCTCATCTGGCTCGTTGCTGACCGTCGCGTTTCAAATTCTCATGAGCGGCTTGCCATCT
GCAAAACTCAACTTGGAGTCGGTGATGACGGTCAACAACTTGATTGGCACCACGGTTCAGCAAATAAAGTCTGCC
TTGAACTGTCCTGACATCTGA

GSMUA_Achr10P17790_001 (*MaHDZIV16*)

TACGTCTTACAGTTTGGAAAACAGTTATCCTACGTATCATTGAGAGTTCAAAGGGATCAAATATGAAATTATATT
ATAGATTATATAATAATAATAAAATATACGATCAACTAAACATTATCAAATTTTATATATATATATATATATATA
TATGTATGTATGTATGTATTAGAAGAAATGGTTCATCCCTCTATTTCTAGAATGGAAAGATCCATAGATCAGTAT
CCCAGTATATATAGATAGAAATGTTCCAATGAGTATTTTGACACTTCATATGAAAGCCAATGATATCCTTGATTG
TCATGTGGCTACCTTGAGCTACAATAGAAATGCTATTATGGACAAAAACTTTGGCCCTTCTTATGATGATACTTG
AGTTTAAAGCCTGACAAGCTGCTCAATCATGAAAGGGATACACCCCCTTAGCAGGCATCAGTGTCACTGTAGTAG
AGAGTAGCTTAGTCCAGGTATGTCCAGGAGCTGAATCCTTTCATACTGTATGCTTTGGGCTGGGCATCAAGAAGC
ATTGATGGTGGGGAAGGACCTCCCACCCCTGCATGGCTGTATGATTTTGGTTAGAAGGAGAGGAGGAATCACATG
ATGGAAACACTGGCCTTTTCATTCAAGGTGCTAGCTAAGTCATCATAAATTGGAATCACCAACCATAACAGCTGA
ATCCGAATGGCGAGGTGCAATGCAACCATAAATGTGCTTTGCTTACCCTACACATGTTCCTGTTTGCAAGGATGG
CTGGTGAGTTATGAGCACGCTGATGCTGCAGATGCCAAGTCCTTCCATGGTGTCTTCTTCGGATCACATCAATTT
ATTATCCTACCTACGTTAAGCCCAGTAGTACAATATCACTGTTTACACCGTGTTCTTTTTTACCTCCCAATTCTA
TTATATATGTGTATGTATCAGCTCCAAAGTACTTGAGCTTTGGAGAGGTAACAGCAGAGAGAGAGAGAGAGAGAG

AGAGGGAGAGAGAGAGTTGCGTCCCGCTTCTCGTCTTCCTTCAAAGCTTTGCCGCTTCAGAGGAGGCGAAAGATG
AGATAGAGTACGGGAAGAAGGCAATCCAAAACCATTTAACTCGCGTCCTTCGTCAGTATTTTCCCTCCGCAAAGG
AACGAACGGTATAATTCGTAAAGTTGTGAGCTTTTAATACACACAGAGAGAGAGAGAGAGAGAGAGAGAGAGAGA
GAAAGAGAGAGAGAGACGGGAATGATCGCTGTGCGTTAATGAGGAGGTCAAGCGGACTGAACCTTTTGAGATTTC
TTCCGCGCGCGTCGACGGTTTCCTTATTCGGCTTCAGAGCCGAACCCAGATGAAATCGGAATAGAGAATGGCGAG
CGAGTAAGCACGAACTGGCTCGGCCGGGTGCCACCTGTCAGCCTCGCTGGGATGGGTCCCAGTGCCATGGAGGCG
CTGTAGCGAACGCTCCTGTCATTATAAATAGCGGCAAGGAGGAGAGGTGTTGTAGGAGAGGTTGGAATAGAGGGG
ATGGATTCGGTCGGTGAGCGAGATGTTTCTGAGGTCCAGAGAAGGAAGAAGCGTTTCTACCGGCACACATCCCAG
CAGATTCAGGAGCTCGAAGCGTACGCTCTCGATCTCTTTTTGCATTGTTTCTCCTCTGATCTGGCTGCTTTTTC
GTGGCCTGCATCGGTTGAATGCTTTTGGGTTGGTCAGGATGTTTAAGGTGTGTCCACACCCGGACGAGAAGCAAC
GGATGAAGCTGAGCCGAGATCTGGGGCTGGAGCCCCGGCAGATCAAGTTCTGGTTCCAGAACAGGAGGACCCTGA
TGAAGGCAACCTTTCCATTCCCCTTCTTCTCCATGATTTTACAGTGGGGTGATCTTTCTCGTTGATCAAATCTTG
ATCTTTCTTTGCAGACACAGCACGAGAGGTCGGACAACTGCTCACTGCGGGCTGAGAACGACAAGATACGGTGCG
AGAACATCACCATGGAAGAGGCTCTCAAGAGAGCCGTCTGCTTGTCCTGCGGCGCCGGCCCGCCCCCCGACAACC
CCTTCTTCGACGAGCAGAAGCTGCGGATGGAGAATACACGCCTGAAGGAAGAGGTACTTGAGCTGCTGCCTTACG
ATGGGATCTTGTCATCGCATGTTCCTGTGTAGACTTTGGCTTTCGAAAGTGGCAGTCGAGTAATCCTTCGTCTGA
TAGTAAATTTTATATTAAGATAAGCTCTACTTCGATGTTAAATTCATATTTATTTATTTTAATGAACTCAATTTT
GTTAATTAATGCTAGGATGAAAGAACTATGAGGTAATGAAGTATTAGCATAAATCACCTTTCTTTGCTCTTGGGA
TTAATGATGTCGAATTATTAACTACTTTGATTATGGTTTTTCACTGTCATTTAATGTCTCCGACATTTAACATCA
GAATCCTTCCACCGCTTACTCGCATAAAACTTGTTTTGCTTCATGTAATTAAAGGGAGAGGTTATGCTAATGTGA
TTAATTTGGTCTCCCTGTGCTGGAAACTATCGCAGCATTTCATCTAACTACAAACCAACGCGTGATCTGTACTCG
AGCCACTGTTGGTTTTGATTAGAACTCTGACATGTTCATTAATTTCTCTTTATTCCCAAAAGAAACATTTTTTTG
GGTTAAACCGGGATCTAATATTCTTCGCATTGCTTGGCAAATTCCAAACTATTCAGGTTTTTCTTTTCTTTTTTT
TCCTTTTTTTTCATGTGAATTATTTTATTTGTTTATCCTATCCTGGAATTGTTAGATACACACACACACACACAC
GTAAAGGGAATGAAAATTTTGATGCTAATGTCCTGCTGCAGGTAGATCGCCTCTCAGAAATTGCATCGAGGTACT
TAGGCAGGCCTATCACTCAACTTCCTTCGAGTCAGCGACTATCGGTCTCACAATCTGAAGTGTCCGTAGGAACTT
ACTATAATCCAGGGATTAGCTCTTCCCTTGATCTCGGCCTCTTGTGCCAGAGTTCCTCTTCTGTTTTATCGAACC
CTTCTCCCACAACTATTTCTGATCTCGAGAAACCTATTATGATGGACATGGCCTCTGCTGCCTTGGAGGAAGTGG
TCAAGTTGATTCAGACCAATGAACCCCTCTGGCTGAAGTCAGCAAGTATGGGGACAGAGGTCCTTCAATCTGAAA
CCTACGAGAGGATCTTTCAGAGACCGAGTCAGCAGCAGTTCAAGTTCTCAGATACGCGAACCGAGGCATCGAGGG
GCTCAGCTTTAGTGATCATGGACGCAGCCACACTGGTTGATATGTTCATGGATTCTGTAAGCATTAGCCTTGCTT
TAAATGACTACTTTTTTTCTTCTTCTTCTTCTTCTTCTTCCTTTATCATGTTTCGATTGCGTCGGATTCGGTTT
AATCGCAGAGCAAGTGGATGGAGTTATTTCCGACCATAGTTTCCAATTCGAGGACCATCGATGTTCTTGCTTCTG
GAACGTCAGGAACGAGGAGCGGATCTCTACTATTGGTAATGATATGTTCTTCTGTACCTTCGATCGATACAAAGC
TACTGATTTTTCTCGTCTATATTGATCTAACCCAAAGGTACGTAGATGTATGAGGAACTGCAGGTTCTGTCGCCG
GTTGTTCCTACACGGCATTTCTGCTTCCTACGATATTGTCAGCAAATCGATCCTTGTTTATGGGTGATAGCTGAT
GTTTCGGTCAACTATCCTATGGACAGTCGTCATCTCCCTTCTCCCCTGTCGTGCAAGCTTCCATCTGGCTGCTTG
ATTGAGGCTATGCCCAATGGATACTCTAAGGTGATCGGTATCCAATTCTGTAGATGATACTCTCGACATTGCTGT
ATCTTATTTAGTTAACAGGATGTTCTTGGTATCTTCGATGTAGGTAAGTTGGGTCGAGCACGTACAGTTCCAAGA
GAAGAACACAATCCATCGACTCTTCCGAGATCTAGTAAACAGTGGAACTTCTTTTGGCGCAAATCGATGGCTCAC
CACTCTACAGAGAATGTCTCAGAGGTTTGCGTGCCTGATGTCAGCTGGACTTTCTACCAGGGACATTGCAGGAGG
TAATGGCTAACCGCTTGAAATGCATGGAAGCACTTTGACCGACTGGTTTATACGCTATTTGACTGTGAATTCAAC
TGTGGGTCTCAGCGGTTCCCTCGGTCGAAGCTAAGAAGAGCATGATGAAGCTTGCCCACAGATTGGTGATGGACT
TCTGTGCCAGTTTTAGTGCTTCTGTGGGGAATAAATGGACCATGCTGTCCGGGATAAATGATGATCTCCGAGTCA
CTCTTCACAGAACAGATTCCTCCCTGCCTCATGGTGTTGTCCTCAGTGCAGCTACATCAATATGGCTACCATTGC
CCAGGGACAGAGTCTTTAGTTTCTTGAAGGATGAACAAAACCGACCTCAGGTCGCTCTGCAACTTCATGAGACAT
CGATCGTAGCTTGTCTCTCTTGTAATCAATCGCTTATGACATCTAACTTGCTGTTTATATGATTGCTTTCAGTGG
GACGTGTTCTCGATCGGAAACAATCTGCAAAAGGTTGCTCACATTACAAACGGTTCAGATCAAGGAAACGCAATC
TCTATCCTTCGTGTAAGTTCTTAATCGTCTCATTTCCTTATAGATATCAGGTAGTGTGTCATCACATTACGGAAA
TCATTCATTACTGTTCTTCTTCTTCAATTTGCATCAAGCGTGAATTACTTCATTTCAGTTATGATCACTTTGATA
GTTTTCTTTGTTTGATGGTGAACAACAGGGATTAAACTACACTCACAACATGTTGATACTCCAAGAGAGCTGCAC
TGATGCATCTGGCTCGGTCGTGGTGTATTCTCCTATTGATCTACCGGCCATGAACACTATCATGAGTGGTGAGGA
TCCATCCTACATACACATATTGCCGTCGGGCTTCACCATACTACCTGATGGCAGATTCGGTGTGGGAGGAGGAGC
TTCAACCAGCTCTTGTCCAACGGGAAGGCCTTCGGGGTCGCTGGTTACTGTTGCATTCCAAATGCTGATGAGCAG
CTCTCCAGGTGCTAAACTGAGCTTCGAGTCAATAGCCACAGTGAACAACTCGATAAGCAATACGATTCATCAAAT
AAAGGCTGCCTTGAGCTGCCCCAGCGTCTAATAATCTGAAGACCATTAAGATCTCCCTCCTCTCTCTAAAATTTC
CAGTGGCTGCTTCAAGTTTGATGAGATGGGGAGGGCATCCTGTTGTGGCATTCGGAAGAAGATTAAAGCGGTCT
TGTTTTGTTGGTTGGTCGTCAATACTTCCGTCAGCATGGAAGCTGCTGCTGCTGCTGTTAGTCTCGGGACAACTC
CGGTCTTCGTGTCAAATAAACCAATCAAGTGCTTACATTTAGAAGCTAAGCACTTGAAGGAGTCAAGAACGCACC
ATACCTTCTCCCTCTTCCTCTGTTGTCAAAGCTGTAGGCTTTTTCTTTTCCTGTGTTCCATCTTTTCCATTCTTC
GTGCAGCAAGAGGGTGTAGGTTCGGGTATTGACTTCTGTCCCTGAACTTTCATGTATCAATAAGCAAATCTTCTC

CAAACTGACTTGCTCTGAAATTGAGAAGCATTTCCACATCTCAAATCTTTGGCACAATGAGCAGGAAACAGCAGA
TGCAGA

CDS
ATGGATTCGGTCGGTGAGCGAGATGTTTCTGAGGTCCAGAGAAGGAAGAAGCGTTTCTACCGGCACACATCCCAG
CAGATTCAGGAGCTCGAAGCGATGTTTAAGGTGTGTCCACACCCGGACGAGAAGCAACGGATGAAGCTGAGCCGA
GATCTGGGGCTGGAGCCCCGGCAGATCAAGTTCTGGTTCCAGAACAGGAGGACCCTGATGAAGACACAGCACGAG
AGGTCGGACAACTGCTCACTGCGGGCTGAGAACGACAAGATACGGTGCGAGAACATCACCATGGAAGAGGCTCTC
AAGAGAGCCGTCTGCTTGTCCTGCGGCGCCGGCCCGCCCCCCGACAACCCCTTCTTCGACGAGCAGAAGCTGCGG
ATGGAGAATACACGCCTGAAGGAAGAGGTAGATCGCCTCTCAGAAATTGCATCGAGGTACTTAGGCAGGCCTATC
ACTCAACTTCCTTCGAGTCAGCGACTATCGGTCTCACAATCTGAAGTGTCCGTAGGAACTTACTATAATCCAGGG
ATTAGCTCTTCCCTTGATCTCGGCCTCTTGTGCCAGAGTTCCTCTTCTGTTTTATCGAACCCTTCTCCCACAACT
ATTTCTGATCTCGAGAAACCTATTATGATGGACATGGCCTCTGCTGCCTTGGAGGAAGTGGTCAAGTTGATTCAG
ACCAATGAACCCCTCTGGCTGAAGTCAGCAAGTATGGGGACAGAGGTCCTTCAATCTGAAACCTACGAGAGGATC
TTTCAGAGACCGAGTCAGCAGCAGTTCAAGTTCTCAGATACGCGAACCGAGGCATCGAGGGGCTCAGCTTTAGTG
ATCATGGACGCAGCCACACTGGTTGATATGTTCATGGATTCTAGCAAGTGGATGGAGTTATTTCCGACCATAGTT
TCCAATTCGAGGACCATCGATGTTCTTGCTTCTGGAACGTCAGGAACGAGGAGCGGATCTCTACTATTGATGTAT
GAGGAACTGCAGGTTCTGTCGCCGGTTGTTCCTACACGGCATTTCTGCTTCCTACGATATTGTCAGCAAATCGAT
CCTTGTTTATGGGTGATAGCTGATGTTTCGGTCAACTATCCTATGGACAGTCGTCATCTCCCTTCTCCCCTGTCG
TGCAAGCTTCCATCTGGCTGCTTGATTGAGGCTATGCCCAATGGATACTCTAAGGTAAGTTGGGTCGAGCACGTA
CAGTTCCAAGAGAAGAACACAATCCATCGACTCTTCCGAGATCTAGTAAACAGTGGAACTTCTTTTGGCGCAAAT
CGATGGCTCACCACTCTACAGAGAATGTCTCAGAGGTTTGCGTGCCTGATGTCAGCTGGACTTTCTACCAGGGAC
ATTGCAGGAGCGGTTCCCTCGGTCGAAGCTAAGAAGAGCATGATGAAGCTTGCCCACAGATTGGTGATGGACTTC
TGTGCCAGTTTTAGTGCTTCTGTGGGGAATAAATGGACCATGCTGTCCGGGATAAATGATGATCTCCGAGTCACT
CTTCACAGAACAGATTCCTCCCTGCCTCATGGTGTTGTCCTCAGTGCAGCTACATCAATATGGCTACCATTGCCC
AGGGACAGAGTCTTTAGTTTCTTGAAGGATGAACAAAACCGACCTCAGTGGGACGTGTTCTCGATCGGAAACAAT
CTGCAAAAGGTTGCTCACATTACAAACGGTTCAGATCAAGGAAACGCAATCTCTATCCTTCGTGGATTAAACTAC
ACTCACAACATGTTGATACTCCAAGAGAGCTGCACTGATGCATCTGGCTCGGTCGTGGTGTATTCTCCTATTGAT
CTACCGGCCATGAACACTATCATGAGTGGTGAGGATCCATCCTACATACACATATTGCCGTCGGGCTTCACCATA
CTACCTGATGGCAGATTCGGTGTGGGAGGAGGAGCTTCAACCAGCTCTTGTCCAACGGGAAGGCCTTCGGGGTCG
CTGGTTACTGTTGCATTCCAAATGCTGATGAGCAGCTCTCCAGGTGCTAAACTGAGCTTCGAGTCAATAGCCACA
GTGAACAACTCGATAAGCAATACGATTCATCAAATAAAGGCTGCCTTGAGCTGCCCCAGCGTCTAA

GSMUA_Achr11P03290_001 (*MaHDZIV18*)
TGATAAAAATATAATAGAATTTCAAATAAGAATCCTTATTGATTTAAAAACGAAAATTATTAAATATAAATATTT
TTGACACCTCTAAAAAAAAAAAAAAAACACATACTGTAATTATCTTATCAAAGAACCGATTTAGATCCTTTGAGA
AATACCAAAAAGATATCTTCTGAAAAGAAAATATGTCCATAGAATTCGTTCCATTACAAACAAATAATAACAAAT
TTTAAGTATTTATACGGTATTTTCTGTGGCGGTTGATTCGGACAGTGACGATGAAATAGAAGATAAGGGATTTTG
AGGCGACGGTTGGATACGCTGCGATCGCGCATGCGACGGGAATCCTCCACCGTTGATCCGTGCACCGACCGTGGA
TCTCCGCCCTAATCCCCCCGGCCAATCATTTACCACCGTCGGAACGCTCTTCTTCCCCAACCAAGTCTTCACATC
GACAATCTCCGCATCACACTCGTCTGATTGAGATCAGACGGTGGAAATTATTTGGACGCGTTCCTGACTCCACGC
TGTTCGTTCTTTTTTTTCTGCTGCTGCTCGCTTCTTCGTTCTGCTCGATCGCTTTCTTGTTCGCTTTGAGCAGAT
AAGAGGGGGGGTGGGGGTCCCTCCCGCTATTGGCTGCCATTTCCATGAGCCCTGCGAGTGTTTGGATGCTATTTT
ACTGCAGGGGGATAAAAGGGAGGAGAGAAGGGAAGAGGAGGACGGAAGGGGTGGGGAGGGGGGGGGGAGGGTGGT
GGGGGGAAGAGAGGTGTGCGGAAAAAGGTTGCCCCTCGCACCGAGGTGCCAGTTTTGATGTGAAGGTGTGGACTT
TTTGTTCTTATCTTTTACGAGAACGACGGAGGCGACGGTCAACCGATCTCCTGATCTCGCGTTTTCCTTCTTCTC
TGAGATGCTCTCCTTTTTCTTTCATTTCTTAGGCCTCGGAGTTTCCGCATATAAAGATGCGTCTCCCCTCTGTGG
TATCCTCGCCTCGCCCACTTCACACCGTCGTCCACCGTTTCTGAGCTTGTAAAGCTTCGATCTTCCGGGAAAGCC
ATGCAGTAGTGAGAAGCGGAGATTTGTTTTCTTGTTCTGTGCCTTTCTTTTCCCTGTGTTCTTTATTCTGTTGGG
GTCGGTTTGATGGTGTAGGAAAGGGGAAGGGAGAGGGGGGGAGAACGAGGTGCTAGCGATCTTTGCTTGGCTTCC
TTCTCTTTCTTTCTGCCCTTTGATTAGGTGACGGAGAGGGAGTTGGAGGCATACATAGTTCATACCACGGTCTAT
GCTTCTGCCTCCACTGGCCTGTCTTCCATCTTTCTTTACTTAAATCATTTTTTTGTTTCTCCTTTTTTTCTTTTT
CTTTGTTGTGGAGATACTGTTAAACGCCTCGCATAGATCGAGTAAAGTTGTGCACTCATTAGATCTGGTGCTGCC
GTCACTGCTCCTCGGAGGAAGGGAAGGCGCCGTTGAACGGAAAGAAGCGACGTTGAAAAGACGGGGAGATCTTTG
ATGACTTTTGGGGGTTTGTTCGACGACGGTTCCGGAGGCGGCGTTGCCCGTCTGGTCGCCGACATAGCGTACGGC
GGCACCGCCGACTCCGTGTCGCACCCACGGCGCCTTGTTTCGCCTTCGCTCCACGAGTCCATGTTCGCCTCCCCT
GGCCTCTCCCTCGCCCTGGTAACCCATCGTTTCCCCTCCCGTTCTTTCTTTTCCGCTTTCACCTTTTCTGAGCGA
CTTTGGTTCCTTCGCGCAGCAAACCAACTTGGGGGCGCACGGGGAACGGAACTTGGCTCGTGTCGGAGGAGTTGG
AGGAGAGCTGGATTCGGTTCGTCGGAGCAAGGAAGATGAGAACGAGAGTAGAGCGGGCAGTGATAACTTTGAAGG
TGGATCTGGAGACGATCTGGAACAGGAGCATCCTCGCAAGAGGAAGAGATACCACCGCCACACCCCTCAGCAGAT
CCAAGAACTGGAAGCGTAAGGGATCTGTTCGCATCCTAATATCACTCGCTCATTTCCCAAACCAAAGCTGCTTAC

TTTGGTTCCCTATGTCCAGTCTCTTCAAGGAATGCCCTCATCCGGATGATAAGCAAAGGATGGAGCTCAGCAATC
GGCTTTGCTTGGAGGCCCGCCAGGTCAAGTTCTGGTTTCAGAACCGACGAACCCAGATGAAGGTTTTTGTACTCC
CTCAGTTCATCTCTTCCCTAAAAATTCGATTTTTGTTGGTCCTTTCGTCTCTTTTTGACATGCTCGCGAATCGTG
GAAACCAGACCCACCTGGAACGGCACGAGAACACAATCCTTAGGCAGGAGAACGATAAGCTTAGGGCGGAGAACC
TATCCATCCGGGATGCCATGAGGAACCCCATATGCTGCAACTGCGGGAGCCCCGCAGTGCTCGGCGAGATCTCCC
TCGAGGAGCAGCACCTGAGAATCGAGAATGCTCGCCTCAAGGATGAGCTCGACCGTGTTTGTGCCCTCGCTGGAA
AGTTCCTCGGCAAGCCTGCGTCCCCCTTGGCCAGTCCGCTCCCTCTTCCGATGCTGCCAAACTCGTCCTTGGAGC
TCGCGGTCGGAACAAATGGTTTTGCCGGCCTTGGTTCGGTGGCTACGTCGACTTTGCCTCCGTTTACTGATTTCA
CTCCCGCGGCGTCAAGCCCCGTCGGCACCTTCGTAACTCCAGCGCAGGTGGTTGGCGCCGGAGCCACCGGCGCGG
TGGACAAGGCGCAGGAGAGGTTTGTATTTTTGGAGCTTGCACTCGCTGCCATGGATGAACTGGTGAAGATGGCCC
AGATGGAGGAGCCACTCTGGATTCCGGGCTTGGAAGCCGGTAGCGACGCGTTAAACTACGACGAGTATTACCGGT
GCTTTTCCGGGTGCATCGGGGCAAGGCCTACCGGCTTCGTGTCGGAGGCCACGAGGGAGACGGCCGTGATCGTCA
TCAATAGCCCGGCTCTCGTCGAGACCCTCATGGATGCGGTAATCTCTATTCTACTCACGGTCACTGGATACGAAC
AGCGAGCCCATTCCTTATCGATTCATATCTAATTCATATTCGATTCACGACCAGGCTCGATGGGCAGATATGTTC
CCTTCTGTGATCGCTAGAACGACGACCACCGACGTGATATCCAGCGGCATGGGCGGGACTAGAAATGGTGCTCTC
CAGCTGGTGAGTTCATATGGCCTGCATGCGACCATTTCCGATCATCTGGTTTCAGCCATTTACGTATATCCGTAA
CCATGCTTGTTCTCTTGTTGCCCATCAGATGCAGGCGGAGCTTCAAGTTCTCTCTCCCCTGGTTCCTGTTCGAGA
TGTCAGTTTCCTTAGGTTCTGCAAGCATCTAAGCGAGGGCGCTTGGGCCGTGGTCGACGTCTCCGTCGATGGCGT
CAGAGACAACCGACCTGCTCCACCTGCTACCGTGAAATGCCGCAGGCTTCCTTCTGGGTGCGTGGTGCAGGACAT
GTCCAATGGCTATTCTAAGGTAATAGAAACCCCATCGTCGATAGAGATTTAGAACCTCGTAATTTGGCCTGAATG
ATACCTTATGTTTCCATATATTAGTTTATAGTGGTCGAGGCCTGATCTCGTGCTCCCAAATCGGGTTTCCATGAG
TCACACACGTCCATCCATGCTTATTTCTCGTGTTTCCTTCTCGAGAAGTTTTAGATGTTTCTTCTACCCCTCTCG
AGAGCAACACACCCTCGATCACTCTGTGTCCGCATTCAATTCGTAGGTGCCTTGTTTCTGCTTACCTATCTCGCA
TCTCCCAGCGACCTCGACTACTGTTTAGCTTTGTTTAATCCAAGCATGGAAGTCTCCTCTCTTGCTGCCCTCGGA
ATACGATCACCTAATTTTTACGTCTTGATTGGTGCCGGTGATTCTATTCCCATCTTCTATTCCCCCGTCTTGGAC
ACCTCTCTTTATAATTTACGTTATCCCTGCCTCTCAACACCGTCCTTGCTTGATCTCAGTAATCAACACATGGGT
ATTGTAGCCCATGGAATTCCATTATAGGGTTTAGTGATGAACGCATCCGTAAGCCCTAGTTTGCAGTCTCAACTA
TCCATGTTGGGAAGAGAGACCATAAGCTGTGCCTACCAGTGGTAAGGTGCACTGGCACCCATGGGGCATGGTGAT
ATACAGGTCCAGCTGGTGAGACTGGGGGCGATATGGCAGAGAGGCAATTCGACAAGAGGTATTAATGAGCTAACG
ACGAGGGTGGTGCGCAGGTCACGTGGGTCGAGCATTCTGAGTATGACGAGGCCACAGTGCACCCGCTGTACCGCC
CGCTGCTGCGCTCCGGCTCGGCCCTCGGCGCCCGCCGCTGGGTCGCGTCCCTCCAGCGCCAGTCCCTCGCCGTCC
TCGTACCCCCATCCCTCTCTCCCGGTGGTGATTCCAGTAAGTCTTTATGTGAATTGGTGAATGCCATAGCGGCTG
TTGCCGTTCGTAGCATTAAGTGCTCGGATTGCTGACGGGATGCCATGGCAGCGATAACGCCGAGCGGGCGGCGGA
GCATGCTGAAGCTGGCGCAGCGGATGACCGACAACTTCTGCGCCGGAGTATGCGCGTCATCGGCTCGCGAGTGGA
GCAAGCTGGGCGGCGCGATCAACATCGGGGAGGACGTGCGGGTGATGACGAGGCAAAGCGTGGCCGACCCCGGGG
TGCCGCCGGGGGTGGTGCTCAGTGCCGCCACCTCGGTCTGGCTCCCGGCCTCCCCGCAGCGCCTGTTCGACTTCC
TCCGCAACGAGCAGCTCCGGAGCCAGTGGGACATCCTCTCCAACGGCGGGCCCATGCAGGAGATTGCGCACATCG
CCAAGGGCCAGAACACCGGCAACGCCGTCTCCCTCCTCCGTGCCAGCGTCAGTATCCTCTATCCCGTCTCAACAC
ACCACCATGGCCCTTTAGCATACAACTCCTATTTACTGGTACTCATTACTAGGCTAGACAGTAACTTCACATGCA
TTCCTCATCAAGATCATCAAGGTCAACCGTGCTCACTGTTGAGAATCTTAAAGTAAATTTTCTGGGTGGTCGTCT
CTGGTGGCACCCACCTGTCTCGAAACAACCGTCATCCTCTCTGCCCAAGCATGGCTGTTTTCTTTCCTCTGCATA
CACATGCAGCGGTGGCAGTAGACTTCGCATGTGACCAGGCGCGATGGCCATCGATACGACTGTACCGTAGCTGTG
TTTTCTTAGATCTGACGTCTCGTTGACGTTTGACCTCTGTAGATGCCCTCGGTCACACTCAACTCGTATTTTGTA
CATACGTACTCGTCCTGATCGATGCATTTGTTGTGATTTGAAGCAATGTGATCTTCTTAAGCATGGTAGTTTATC
TGCAGGCGGCGAACGCAACCCAGAGCAGCATGCTGATACTGCAGGAGACCTGCACGGACGCGTCGGGGTCGCTGG
TGGTGTACGCCCCGGTGGATATACCAGCCATGCACCTCGTCATGAGCGGCGGTGACTCTGCCTACGTCTCGCTCC
TCCCCTCCGGCTTCGCCATCCTCCCGGACGGCGGACGCACAAGGCTCCGGGATCGCTGCTGACGGTGGCCTTTC
AGATCCTGGTCAACAGTCAGCCGACGGCGAAGCTGACGGTGGAGTCGGTGGAGACCGTCAACAACCTCATCTCGT
GCACCGTTCAGAAGATAAAGGCGGCACTCCACTGCGAGACTTGAGAGGACGGAGATCGAAGTGCGACGAGGAGGG
GGGGGAGAGTGTCATGGCTGTGGATTGAGTCAAGAACGAACCGCGCGAATAAAGAACCTTTGCTAAGTGATAAGG
AATAATGGCCGACTGCAAACCTTGTACGCAGTCCATCATCCTCATCACCGAGCAACGGTGGTGGTTCGGGTATTG
ACTCTCTCTCTCTAAATGCCGTCGCTCGATCCCAGCCAGAGTTAATGTCGTGTCAGGTTGCAGCGTTCTCTTCTT
TTCCTGTTTGCATTTGATTCACCTGTAGAGGACAACGTAATCTTCTTCCCTGTATGCAAGTAGACAAATGCGTAT
GTATTGTTGCGAAGTCTTTTTTGTTGACTTTGTTATTTACTTCTTTTAAGTTTGATTTTAGCATATATATATTAT
GAGCTTTTATAAGATTTTTGTTTTTGATTTGATCAAAACACCCTTACTGGAATCGTAAAAAGATGATAATTATTT
TTTGACTAGGTTTGATTTG

CDS

ATGACTTTTGGGGGTTTGTTCGACGACGGTTCCGGAGGCGGCGTTGCCCGTCTGGTCGCCGACATAGCGTACGGC
GGCACCGCCGACTCCGTGTCGCACCCACGGCGCCTTGTTTCGCCTTCGCTCCACGAGTCCATGTTCGCCTCCCCT

GGCCTCTCCCTCGCCCTGCAAACCAACTTGGGGGCGCACGGGGAACGGAACTTGGCTCGTGTCGGAGGAGTTGGA
GGAGAGCTGGATTCGGTTCGTCGGAGCAAGGAAGATGAGAACGAGAGTAGAGCGGGCAGTGATAACTTTGAAGGT
GGATCTGGAGACGATCTGGAACAGGAGCATCCTCGCAAGAGGAAGAGATACCACCGCCACACCCCTCAGCAGATC
CAAGAACTGGAAGCTCTCTTCAAGGAATGCCCTCATCCGGATGATAAGCAAAGGATGGAGCTCAGCAATCGGCTT
TGCTTGGAGGCCCGCCAGGTCAAGTTCTGGTTTCAGAACCGACGAACCCAGATGAAGACCCACCTGGAACGGCAC
GAGAACACAATCCTTAGGCAGGAGAACGATAAGCTTAGGGCGGAGAACCTATCCATCCGGGATGCCATGAGGAAC
CCCATATGCTGCAACTGCGGGAGCCCCGCAGTGCTCGGCGAGATCTCCCTCGAGGAGCAGCACCTGAGAATCGAG
AATGCTCGCCTCAAGGATGAGCTCGACCGTGTTTGTGCCCTCGCTGGAAAGTTCCTCGGCAAGCCTGCGTCCCCC
TTGGCCAGTCCGCTCCCTCTTCCGATGCTGCCAAACTCGTCCTTGGAGCTCGCGGTCGGAACAAATGGTTTTGCC
GGCCTTGGTTCGGTGGCTACGTCGACTTTGCCTCCGTTTACTGATTTCACTCCCGCGGCGTCAAGCCCCGTCGGC
ACCTTCGTAACTCCAGCGCAGGTGGTTGGCGCCGGAGCCACCGGCGCGGTGGACAAGGCGCAGGAGAGGTTTGTA
TTTTTGGAGCTTGCACTCGCTGCCATGGATGAACTGGTGAAGATGGCCCAGATGGAGGAGCCACTCTGGATTCCG
GGCTTGGAAGCCGGTAGCGACGCGTTAAACTACGACGAGTATTACCGGTGCTTTTCCGGGTGCATCGGGGCAAGG
CCTACCGGCTTCGTGTCGGAGGCCACGAGGGAGACGGCCGTGATCGTCATCAATAGCCCGGCTCTCGTCGAGACC
CTCATGGATGCGGCTCGATGGGCAGATATGTTCCCTTCTGTGATCGCTAGAACGACGACCACCGACGTGATATCC
AGCGGCATGGGCGGGACTAGAAATGGTGCTCTCCAGCTGATGCAGGCGGAGCTTCAAGTTCTCTCTCCCCTGGTT
CCTGTTCGAGATGTCAGTTTCCTTAGGTTCTGCAAGCATCTAAGCGAGGGCGCTTGGGCCGTGGTCGACGTCTCC
GTCGATGGCGTCAGAGACAACCGACCTGCTCCACCTGCTACCGTGAAATGCCGCAGGCTTCCTTCTGGGTGCGTG
GTGCAGGACATGTCCAATGGCTATTCTAAGGTCACGTGGGTCGAGCATTCTGAGTATGACGAGGCCACAGTGCAC
CCGCTGTACCGCCCGCTGCTGCGCTCCGGCTCGGCCCTCGGCGCCCGCCGCTGGGTCGCGTCCCTCCAGCGCCAG
TCCCTCGCCGTCCTCGTACCCCCATCCCTCTCTCCCGGTGGTGATTCCACGATAACGCCGAGCGGGCGGCGGAGC
ATGCTGAAGCTGGCGCAGCGGATGACCGACAACTTCTGCGCCGGAGTATGCGCGTCATCGGCTCGCGAGTGGAGC
AAGCTGGGCGGCGCGATCAACATCGGGGAGGACGTGCGGGTGATGACGAGGCAAAGCGTGGCCGACCCCGGGGTG
CCGCCGGGGGTGGTGCTCAGTGCCGCCACCTCGGTCTGGCTCCCGGCCTCCCCGCAGCGCCTGTTCGACTTCCTC
CGCAACGAGCAGCTCCGGAGCCAGTGGGACATCCTCTCCAACGGCGGGCCCATGCAGGAGATTGCGCACATCGCC
AAGGGCCAGAACACCGGCAACGCCGTCTCCCTCCTCCGTGCCAGCGCGGCGAACGCAACCCAGAGCAGCATGCTG
ATACTGCAGGAGACCTGCACGGACGCGTCGGGGTCGCTGGTGGTGTACGCCCCGGTGGATATACCAGCCATGCAC
CTCGTCATGAGCGGCGGTGACTCTGCCTACGTCTCGCTCCTCCCCTCCGGCTTCGCCATCCTCCCGGACGGCGGG
ACGCACAAGGCTCCGGGATCGCTGCTGACGGTGGCCTTTCAGATCCTGGTCAACAGTCAGCCGACGGCGAAGCTG
ACGGTGGAGTCGGTGGAGACCGTCAACAACCTCATCTCGTGCACCGTTCAGAAGATAAAGGCGGCACTCCACTGC
GAGACTTGA

GSMUA_AchrUn_randomP10540_001 (*MaHDZIV21*)

GATAAGAACTCTGAATTATAAAAACATAAACTAAAATGTGAGTCATTTCTAAGAAAAATGGCGTAAGATTTCGAA
CAAAAACTAATCGTCAGATGAAAAAAAAAAAGGATGAACATGACATGTTATGGCTGTCCTAACTATCGTACGCAA
AATTTTTAGATTTAAGAAAATTTTGTAAAATCTATGGATGGAGTTTTAACTCCCACCAAACCCACCCTGTTCAGC
CAAGTCCTTCGTCTCACCCGCCATGTTGACTTTTAAGCCCTCTCTTCTTCCAAAAACCTGTGTCCTACACCTGTG
ACAGCAGCGGTCCAGAGCCTCTGCAGCCCTGTGATGCGGATCAGAGGGCTGACATCTCTTCTGCGCCGGGTCTCC
CATGTCTTCTGCTGTATTCTGAGCAGGAGGAGATGGAAGCAGGGAAGGCGTGGACTTCCTTCTCCGAGACGAGCC
CGTTGGTCACTTCTCTGCCTTTTCCCGGGGCGTGCTCACACCGGTCCTCCTCCGAGGTGAGCCGTGGTACCGTTT
CGTCTCTAATTCGATACCTGTTTTGTTTTCTTCTATCTTTTATCGCTTTCCATCGAGTTCTCACGTTGCCTGTTG
ACGGAGCGGAGGAAGACGAGAAGGAAACAAACCAGATGGCGGCAGGACCTGTACCAGTTTCATCTCTCTGCTGTG
AAAACTTGTTTTTTTTTTTCTTTTAACTCGAGCAGACGATGGAAAGGGTCGTAGGGCTCGAGAAAGAGACGGTGC
AAATCTGCTTCATTCCACAAACCCAAGGTCCCGTTTTTTATCCCAAGGTTGGGGCTTTTTGATTCATTTTTTTAT
GGGACAGATGACGGGAGGGTCAACAGGCCTATTTCTACTGACGTCTCTTTCTCTCTCTTCCTCGGCCTAGAGATT
TCCTTTTCCTTTGTTTTGCGAGGCTTCATAGTGTCCACGCGTAGGAAGTCTCCTTTTATCTTCCACACCCGTTTA
CACCAGCTCGCACCAGCCTCTCGGCCTTCTAAAGCTTCGATCTTTGAGGTCTCGGAGCAGGAAACTGCCTCGTTC
GCGTCCTAGGTGGTAGGGAAGGGAGAGGAAGACAGAACGCGGTTCTTGAGTTCCTCTTGCCTTCCTTCTCTTTCT
TTCTGCCCTTCGGTGACAGCGAGAGGGTGTTGAGGCATCTGCTCATACCTTAAACGGATCGTCTATGCTTTTGCA
TCCTCTGGTGTATCCATCAGATCTCCCTTTTTTTATTTTTTTTTGTGTTGTATTTGTTCTCCCTGAGTCCTCTT
CCCCTCTATTCAGTTCTGTCGATTTGATTCTGCTCCTTTGTTTATTGCATGGAGATCTTGTTCTAAGACCTTGGG
AAACTCGGAAATCTGGGTTGAAAAGCGAGAACACGCATTAGATCTACTACTAGCATCTGCTAGCACTTGGTGAGG
AAGAGTGTTTTTGAAGCAACGAAGCGAGTTCGAAGAGGTGTGATTGAGAAAGAGAGGAAATAAAATTGATCTTCA
ATGAGTTTTGGAGGTTTGTTCGACAGCGGCTCCGGCAGCGGTGGCGGTGGCGCTCGGACGGGGTTGGCCGATGCG
GTGCACCGCAGCTCCACCGCCATGTCCGCCAGCAACATCTCACAGCCGCTCCTCGCCTCGCCTTCTCTCCCCATG
TTCAACTCCCCTGGCCTCTCCCTCACCCTGGTAAGTTTACCACTCACTATTTCTCTTCCTTTTTCTTCTTCCGT
TTTCTTGCGTTCATGTAGAGGTTTCAACGAGTATACCTTTTGGTTTTCCGGCCAGCAATCGGATATGGAAGGCAC
GGGTGCCGCCGGTAAAGGTGGAGCGTGGGATCTGGATTCGGGGCGTCAGAACAAGGAAGACGAGAACGAGAGCCG
GTCAGGAGGGAGCGACAACCTGGGAGGACTCTCCGGAGACAATTTAGAACAGGAGAACCCGCGCAAGAAGAAGCG
ATACCATCGCCACACCCCGCACCAAATCGAAGAACTCGAAGCGTAATGAGTCCAACCATCTCACTCCATTCCTTT

1415 GATTGCAAGAACATTAAACGACCGAAAAAAATAGACAGCTCTCAATTTAATCCCCCATGTACAGATTCTTCAAGG
1416 ATTGCCCTCACCCTGATGAGAAGCAAAGGTTGGAACTCAGCAAGAGGTTATCCTTGGAGGCTCGCCAGGTCAAGT
1417 TCTGGTTCCAGAACCGCCGAACCCAGATGAAGGTATCATTTCATTCTTTTCTAAAAATATTCATCTACTTCTGAT
1418 TATTATATCTTCCTTACATGCTTGGGCGTCGATTGAATCAGACCCAAATGGAACGCCACGAAAACATGATCCTAA
1419 GGCAGGAGAACGACAAGCTTCGCGCTGAGAACTTGTCAATTAGGGAGGCGACGAGGAACCCTCTCTGCAGCAACT
1420 GCGGTGGGCTGGCGATGCTCGGCGAAGGGTCGCTCGAAGAACAACACTTGAGAATTGAGAATGCCCGTCTCAAGG
1421 ATGAGCTTGATCGTGTTTGCTCCCTCATTGGGAAGTTCCTCGGCAAGCCTATATCTGCGTTGTCTAGTCCACTTC
1422 CTCTCCCGATGTCAGATTCAACATTGGATCTTGCAGTCGGGAACAATGTTTTCGGTGGCCTGGGTTTGGTGACCC
1423 CGGCAACGTTGCCTCCGGTCACTGATTTCACCGCCGGAGCAACAAGCGGCCCATTCGGCACCGTTACAACTCCTG
1424 CAAGGAACGTTGGCACGGGAACTCTAGACGGTGTGGATAGATCGCAGGAGAGATTCGTGTTCTTGGAACTTGCAC
1425 TCACAGCAATGGATGAGCTGGTGAAGATGGCTCAGATGGAGGATCCTCTTTGGGTTCCAACTTTGGATGGAAGGA
1426 AGGAGACACTGAACTACGAGGAGTATCTTCGAAGTTTCCCTCGCTGCATCGGTGCGAAGCCCGTTGAGTTGGTGT
1427 CGGAGGCCACCAGGGCTACCGGCGCCGTGATCATCAATAGCTTGGCGTTGGTTGAGACTCTCATGGACGCCGTGA
1428 GCTTTCGTCTGTTGCATTTGCTTTTATCAGCTGATCACTTGCATCTTCCAGCATCATGCTAATAAAATCTAATTT
1429 CTTTTTATCATATTCTTACATCAGAATCTTTTTACTGCGTTTGCATCGATCTGCATTGAAATGTTTCTTTGCTTG
1430 CTGTTTCACCTCCTATACAGATAATTTTCTTCGAAATCGTCACTTGCATGTGTCCTCCGAGCAACTTTTTGGAGT
1431 GAACTAATACGTCGTTTGCTATATTTGTAACCCATTCATGCGCAGACTCGGTGGGTAGATATGTTTCCTTCTGTG
1432 ATTGCAAGAGCGACCACCATTGATGTGATCTCCAGTGGCATGGGTGGTAGCAGAAATGGTGTGCTCCAACATGTG
1433 AGTCCATGGCATTCAGATGTGTATTTATGCTAGTAGCTGTTTGCTACACAATTATTCTCCTAATTGCACCACAAT
1434 TTCCTGGGTCTGCAGATGCATGCAGAACTTCAAGTTCTCTCGCCGCTGGTGCCTGTCCGAGATGTCCGTTTCCTT
1435 AGGTTCTGCAAGCAACTTACAGAGGGAGCTTGGGCGGTAGTAGATGTCTCCATCGATGGAATTAGAGACGACCTT
1436 TCTGCTCCACCTCCTAATATGAGTTGCCGCCGGTTGCCGTCTGGTTGTCTGGTGCAGGACATGCCTAATGGTTAT
1437 TCTAAGGTATTAAAATCCTTCCTCATCTCCACTCTCTCCCTTCCCGCTCTACCTATTAGCAGTCTATCAGCAGTG
1438 ACCACGCATGGGTTACCTTTGGATCAGTGTTAATAACTTTGACTTAGACATGTATAATATGTCTCTCTGCACACA
1439 ATACGTTCCTCGGCGTGCTCAAGTGTTACTGTTGTGATTCCCTGTCTCTTTTTCTTCGAGTCAAGGCCTAAGCTC
1440 GTGCTTCCAAGTCTAATCGAGGTCTCGTGCCAGTATATCTTCTTCTACCAAAAATATTTCAGTCAGCGAAAGACG
1441 GATTTGTGTTAGTCTTCGATCCTTTTTTTTATTATTAGTATATTCACACGTCTTTCCATGTTCATCATTTCTCG
1442 TGTTTGTTTCTTCAGACTTTTAGATGCTTCTCCCACACTCTCGAGAGCACCACTTCTGGGTTGTTCACCGTCCGC
1443 TTATGAATAATGGATGATGTGCCGCCAACCATTTGGCTAAGGCTGCTCTCACCGTCATTATCTGTGAAGGCTTGG
1444 CTATTGTTCACCGTTCCATTTGTTTTCCAAGTCCTCGTACGGAAATTACCCTTTCTATTCCTTTCCGTGTTCTTT
1445 ACCTTGCCTGTTGTGCAACCCCTTGTACACCGAGTCTCTCTTGGTAACTTTAGATATCATATGCTCTTGACGACG
1446 CTATTTGATCTCACTCTCCTGACAGCGTGCATCATGTCAACAGAGACCTCAATTTAATTTAATAATCAGCATCAC
1447 CTTGGCATTTGGCCAATTGTATAACCTTCTTAGTTTGGTTCAACATTTTCCTATCGTTGTTTCTTCGAGTCAGTT
1448 TCTCATATCGATGCTCATGTGATCCTCGGAAGCTTTTCTCCGTTTGTCTAACCTTCTGACTCGAGTGTAATCAAT
1449 TTAGCAGTGTACTGTGTGCCCAGTGTACTCAGGTAAACAGCAAACTGCGTTTCACGGTGGTAAGGTATACAGCGT
1450 TCTAGGGACATGATACGTTAAGTCAAGGTCCAGCTGTGGCACAAAAGTCAACGTTAAGGGACACCCGGCATTAAT
1451 GAGCTAACTGACGCGGTGATGCACAGGTCACATGGGTGGAGCACGCGGAGTACGACGAGGCCGCGGTGCACCCGC
1452 TGTTCCGGCCGCTGGTGCGCTCCGGCATGGCGCTCGGCGCACATCGTTGGGTCGTCTCCCTCCAACGCCGGTGCC
1453 AGTCCTTGGCTATGCTCATGTCCTCTTCGCTCTCCCATGATGATACCACCAGTAAGTCCCAGCAAAAATGTGCAA
1454 TTGCGATTGGCATGGATTGATGATATGGCGGCTGATTGTTGAACTCTGGTCGCAGCAATAACGCCGAGTGGGAGG
1455 AGGAGCATGCTGAAGCTGGCGCAGCGCATGACGGACAACTTCTGCGCCGGGGTGTGCGCGTCGTCAGCCCACGAG
1456 TGGAACAACCTCAGTGGCGGGATCAACATCGGCGAGGACGTGAGGGTCAAGACCAGCCAGAACGTGGCCGAGCCC
1457 GGGGAGCCACCGGGGGTGGTGCTGAGCGCTGCCACCTCGGTGTGGCTCCCTATCGCCCCACAGCGCCTTTTCGAC
1458 TTCCTCCGCAACCAGCAGCTACGGAGCCAGTGGGATATCCTCTCCAACGGCGGTCCCATGGAGGAGATGGCCCAC
1459 ATCGCCAACGGAAAGGAGACCGGCAACACCGTCTCCCTTCTCCGTGCCAGCGTCAGTATATTTATCGTTTCACGC
1460 ACATCTATTTCCTTTTCGCCTTTCTTTCTCTGGTTGGACATTTTACTTGCGTGATCGGTGAAAGGTTTGGAAAAA
1461 TAATCAGAGTCTGGTACTGGCTACTGTAGCGTCAGACTAGTGGGGCCATGTCGGTGGTCGGGAACTTGCTGGATG
1462 ACAGATCCATGAAAAGATGGATTGACGTGACTACTGACGATCCATTGCTGTCTTTGGCCTTGTGTTCAAGCTTTC
1463 TTCTCACTCTATACGTGGTTTATTGGTTTACGTAATTCACCTTATATTTTATAGATTTTCTGAAGTTAAATGGAC
1464 TTGTTGTATGTTTTGGTCAGTATGTTGCAAATCTGGTTCACTTCCTAAATGATGTGTTGGGTTGTTACAACAGGC
1465 GGTGAGTGCAGACCACAACAGCATGCTGATACTGCAAGAAACGTGCACGGACGCGTCCGGGTCGGTTGTGGTGTA
1466 CGCCCCGGTGGACGTGCCGGCCATGCACCTCGTTATGAGCGGCGGCGACTCCACCTACGTCACCCTTCTCCCGTC
1467 TGGTTTCGCCATCCTCCCCGACGGCCGCGGAAGCGGCGCAGGCGTCACCCACAAGGCGGGAGGGTCGCTACTCAC
1468 CGTCGGGTTTCAGATTCTGGTGAACAACCAGCCGACGGCGAAGCTGACGGTGGAGTCGGTGGAGACCGTCAGCAA
1469 CCTTATCTCATGCACCGTGGAAAAGATCAAGGCAGCCATCCACTGCGAAGCTTGAAGAGGAGCAGACTTGTTGAG
1470 GAGCATGGCGAGGATGTCAGGTAATGCATGGATTGAGTCAAGAACGAACCACAACGTAAAAAACCTTTGCCAAGT
1471 AGTTAGGAAGTATGGAAACACTGCTAAAACCCTATCGGCACTAAGAGCAGTCCATAATCCTTAACACGGAGCAAT
1472 GGTGGTGGTTCGGGCATTGACTCGTTCCAATCGACGCCCTTGCTGAATCTCCAGCGCAGTGAGATGCTGCATGAT
1473 TAGTATTGTAGTGGTTTGGCCTTTGTAGTTTTTGCCATCTGAAACATAGTCAACTAGTGATGAAAGGAGCAAGTA
1474 GTGCTGTTTGCTGCATATGAGCGTAGTTTTTTATCGGACATATAAGTTGCTTCGTATAACTGCATATGAGCTCCA

TTAGCTCTTCCTTTACTTCCTCTGTGTTTACCAGCCATACCATGTCGATAAAAGAAGCATGTGAAGGTAAAAAGG
AAACTGTACTCGTGACTAGTGGGGTTCCCA

CDS

ATGAGTTTTGGAGGTTTGTTCGACAGCGGCTCCGGCAGCGGTGGCGGTGGCGCTCGGACGGGGTTGGCCGATGCG
GTGCACCGCAGCTCCACCGCCATGTCCGCCAGCAACATCTCACAGCCGCTCCTCGCCTCGCCTTCTCTCCCCATG
TTCAACTCCCCTGGCCTCTCCCTCACCCTGCAATCGGATATGGAAGGCACGGGTGCCGCCGGTAAAGGTGGAGCG
TGGGATCTGGATTCGGGGCGTCAGAACAAGGAAGACGAGAACGAGAGCCGGTCAGGAGGGAGCGACAACCTGGGA
GGACTCTCCGGAGACAATTTAGAACAGGAGAACCCGCGCAAGAAGAAGCGATACCATCGCCACACCCCGCACCAA
ATCGAAGAACTCGAAGCATTCTTCAAGGATTGCCCTCACCCTGATGAGAAGCAAAGGTTGGAACTCAGCAAGAGG
TTATCCTTGGAGGCTCGCCAGGTCAAGTTCTGGTTCCAGAACCGCCGAACCCAGATGAAGACCCAAATGGAACGC
CACGAAAACATGATCCTAAGGCAGGAGAACGACAAGCTTCGCGCTGAGAACTTGTCAATTAGGGAGGCGACGAGG
AACCCTCTCTGCAGCAACTGCGGTGGGCTGGCGATGCTCGGCGAAGGGTCGCTCGAAGAACAACACTTGAGAATT
GAGAATGCCCGTCTCAAGGATGAGCTTGATCGTGTTTGCTCCCTCATTGGGAAGTTCCTCGGCAAGCCTATATCT
GCGTTGTCTAGTCCACTTCCTCTCCCGATGTCAGATTCAACATTGGATCTTGCAGTCGGGAACAATGTTTTCGGT
GGCCTGGGTTTGGTGACCCCGGCAACGTTGCCTCCGGTCACTGATTTCACCGCCGGAGCAACAAGCGGCCCATTC
GGCACCGTTACAACTCCTGCAAGGAACGTTGGCACGGGAACTCTAGACGGTGTGGATAGATCGCAGGAGAGATTC
GTGTTCTTGGAACTTGCACTCACAGCAATGGATGAGCTGGTGAAGATGGCTCAGATGGAGGATCCTCTTTGGGTT
CCAACTTTGGATGGAAGGAAGGAGACACTGAACTACGAGGAGTATCTTCGAAGTTTCCCTCGCTGCATCGGTGCG
AAGCCCGTTGAGTTGGTGTCGGAGGCCACCAGGGCTACCGGCGCCGTGATCATCAATAGCTTGGCGTTGGTTGAG
ACTCTCATGGACGCCACTCGGTGGGTAGATATGTTTCCTTCTGTGATTGCAAGAGCGACCACCATTGATGTGATC
TCCAGTGGCATGGGTGGTAGCAGAAATGGTGTGCTCCAACATATGCATGCAGAACTTCAAGTTCTCTCGCCGCTG
GTGCCTGTCCGAGATGTCCGTTTCCTTAGGTTCTGCAAGCAACTTACAGAGGGAGCTTGGGCGGTAGTAGATGTC
TCCATCGATGGAATTAGAGACGACCTTTCTGCTCCACCTCCTAATATGAGTTGCCGCCGGTTGCCGTCTGGTTGT
CTGGTGCAGGACATGCCTAATGGTTATTCTAAGGTCACATGGGTGGAGCACGCGGAGTACGACGAGGCCGCGGTG
CACCCGCTGTTCCGGCCGCTGGTGCGCTCCGGCATGGCGCTCGGCGCACATCGTTGGGTCGTCTCCCTCCAACGC
CGGTGCCAGTCCTTGGCTATGCTCATGTCCTCTTCGCTCTCCCATGATGATACCACCACAATAACGCCGAGTGGG
AGGAGGAGCATGCTGAAGCTGGCGCAGCGCATGACGGACAACTTCTGCGCCGGGGTGTGCGCGTCGTCAGCCCAC
GAGTGGAACAACCTCAGTGGCGGGATCAACATCGGCGAGGACGTGAGGGTCAAGACCAGCCAGAACGTGGCCGAG
CCCGGGGAGCCACCGGGGGTGGTGCTGAGCGCTGCCACCTCGGTGTGGCTCCCTATCGCCCCACAGCGCCTTTTC
GACTTCCTCCGCAACCAGCAGCTACGGAGCCAGTGGGATATCCTCTCCAACGGCGGTCCCATGGAGGAGATGGCC
CACATCGCCAACGGAAAGGAGACCGGCAACACCGTCTCCCTTCTCCGTGCCAGCGCGGTGAGTGCAGACCACAAC
AGCATGCTGATACTGCAAGAAACGTGCACGGACGCGTCCGGGTCGGTTGTGGTGTACGCCCCGGTGGACGTGCCG
GCCATGCACCTCGTTATGAGCGGCGGCGACTCCACCTACGTCACCCTTCTCCCGTCTGGTTTCGCCATCCTCCCC
GACGGCCGCGGAAGCGGCGCAGGCGTCACCCACAAGGCGGGAGGGTCGCTACTCACCGTCGGGTTTCAGATTCTG
GTGAACAACCAGCCGACGGCGAAGCTGACGGTGGAGTCGGTGGAGACCGTCAGCAACCTTATCTCATGCACCGTG
GAAAAGATCAAGGCAGCCATCCACTGCGAAGCTTGA

GSMUA_Achr8P07310_001 (*MaHDZIV13*)

AAAAGGATAAAGGTGACGCTATCGTATGACTGTTTCCCACGTAATTCCCGACACAAACTCCAATCCATGCAAACG
ACACGCGCGCAACTCGCACCACAACACCTCTCCTGGCCTCACGTTCACGAGTCACGGGCCCCACCAACCGTGGGT
CCCAGATCCATCTGTGATCGGGCTTGAAGTTGGGTCACGGTTCGAGTCTCTTTTCCCTCTCTCTCTCTCCGGTTT
CCCTGTGGATACTCTCATCTACGATCTATGTGAGACCGTATACATGCATGCATGCACGCTGCCTGTGTATGCGAG
CGAGGCATGAACGCAGTTAATGGCGACGGGTTTGCTCCCACCGTCGCACACCACCGCAGCAGCACTCAGAGGCAT
TAAATGTGGGAAGTTGGAGAGGAGGAGTCTTCTCATTAATGGCGGAAGCAGCCGGGTCTCTCTGCAAGACAACAG
CAACCCACCTCACCTCTCCTCTACCCTCACCAGGGTTGGGGTCTTTTAGGGTTTGGTCCAGCGCTGAGGAGCATT
AGGGCAGCCAACCGATGAAGAAACTCTTGCTTAAGAAAGAAGCCAACCTTGGAATCTGTCTGACACACAGTCTGC
TCTCCAAGTCTTTTACTATCTACAATCTGTTAATTGGTATCTTTCTTTGATAAGAACAGCTTCAGCCGCAGTATG
ACATAAGTAGGTGGAAATATGTGTGGTTCTATATCTTCATCCTTCCACCAAGAAGTCTATTGACACTAATAACAG
GAAATGATACAGTAAATTAAGCTCCTTGAAGGCAGTAATCTCTAGGAATTGTATGGTCAATCGTCCCTTTCACCT
TATACCCATCTTGTGGAAGAATTCAAAGCACATAGGTGCTCTCTCACTCTCTCTCTATCTCTCTCTCTCGCTCTC
TCTCTCTACCCTAATAACACCACCCTCTCAATAAATGCAATTGCACCCTCTCTAGAGGCTCTGCAATAACTCATC
CTCTCCACCTCCTCTTTCCTCCTTCCTCCCTTTCCCTTGCTCTTCTTCTCTTGTCCAGATCCCCTTCTGTCCCTT
CCATGGTTTCTCTCCTGGTGCTTTCTTCCCTGAAACCACACATCATCCTTTTTCTTGCATGAAATAAACCCTCAT
CCCTTTCGCTTGCTCTCTGCCTGTATCAGAGATCAAAGTGAAGAGGAGAGAGAAAAGAGTGTGTGTCTATGTGCG

TGTGTGTTCATGCCCTAGAACTAGATCTTAGTCATATTAGCTGCTAAGGTTCCTTCTCTAGTTTGCTCTCGAGTT
ATCTCATGATCAAGGCCATCAATCTCTCTTTTCTCTCTACTCTTTCTAAGATAACTTGCAGGAGCTTCGGAAGCC
AAGGTTAGTCCGTGGCAGTCTTGTCGCGAAGAGAAGCCCGAAGACACACAGAAGAATAGATCCAGAGACATCCGT
ATGCAGAGAGACAGATCAGAAGTGGAAGGGGGAAAGAATTAGGGTTTCACCCAGAGCACTATGCCTGTGGGGATC
ATGATACCAGCAAGACAAGCACCATCCATGATCGGGAGGAACACCGGCACCGGCTATGGCTCCTCGTCAGTGTTG
TCGCTCGGCCAGGTCAAAAGCTTTCTTGTTTCTCCCATCATCCCTTTGTCGTAAGCGTTTGTGCAAGTATTTTGT
ATGATAAATGATCTCCATGACCACAGTTCTGCTAGGTTTTCAAAGGCCAAGTGACAAGTTCTTATTCTTCTGCTC
TATATTTCAGCTTCCTTTGACCTCTCCTCGTTCTCTTCTAGAGGAGAAGGAGAAAGATATCTCACGCCGCTTCCT
TCTCTCCCTGCATACTACATCTTATTATGTATATATTTATATATATAGATAGATAGATCTAGACTTGTGTTTCAC
CTTGCACCCTCTCAAGCTCTTCATGCTGATCTTGAGAGAGGTAGAATCTAATCTAATTTTCTCTTTTGATCTTTT
TTTCCTCTCAGTTATTTTAGCCCTAATTTTCTGTTTAAAGCTATCGATTACAGTAGAAGAGGACTAAAAAGGACT
CTTTTTGTGCTTCTTGTAGCCAAACTTATTAGAGGGGCAGCAAATCCCACTTCAGTACCAGCATCACAACCAGTT
CATGGAGATCGCACCGACTACCACAGCGGAGAGCGAGATGCCAAGGGCTCGCGAGGAGGACTTCGAAAGCAAGTC
CGGAAGCGAGAACATCGAGGTTGCTTCCGGTGATGACCAGGACCAAAGCCATCGCCCGCGCAAGAAGCGCTACCA
CCGCCACACACAGCATCAGATTCAAGAGCTGGAAGCGTACTATGATCTCTCTCTCTCTCTCTCTCTGTCTCTC
TCTTCTCTCCTTCATCGAACAGCTTAAGGATTTCGCCTTCGTTCCCCATTCTTTCTGCGATTCGCCGAGCTATTT
TACTCTGCATGTTGTACAAGTTTTGATCTTAACCTGTGTACCGTTCAGCTTCTTCAAGGAATGCCCTCACCCGGA
TGACAAGCAGAGAAAGGAGCTCAGCAGAGAGCTTGGGTTGGAGCCTCTCCAAGTCAAGTTCTGGTTTCAGAACAA
GCGCACGCAGATGAAGGCACGTCTCCATATATCTTCAAGAACTAGTAGGGGCAGTTACTTATCTCTCTGATCTCA
ATTCTTTTGGTGTGGCAGAATCACCACGAGCGGAACGAGAACAGTCAGCTACGAACCGAGAATGATAAGCTCCGC
GCCGAGAACCTGAGGTACAAGGAGGCGCTCACCAACGCGTCGTGCCCCAACTGTGGCGGACCTGCTGCTCTAGGA
GAGATGTCCTTCGACGAGCACCACCTCAGGATCGAGAATGCCCGGCTGAGAGAAGAAGTTGCTCTTCTTCATCTC
TATCACAAGCTTTATGAGGTTGGTGATGGAGCTAATTGCACTCTTCTTACCATGCTTTCACAGATCGATCGGATA
TCAGGGATAGCTGCCAAGTACGTGGGCAAGCCAGTGGTGTCGTACCCGCTGCCCTCTCCTGCCATCTCTTCACGT
TCGCCTTTGGATGTTGGTGTGGGCGGCGAGATGTTCGGAGTCGGGGTCTCGGGCCAGACTGACATAGAAAAGCCA
GTGGTCGTCGAGCTCGCGGTGGCCGCCATGGAGGAGCTCATCAGGATGGCCCAGCTCGGCGAGCCACTGTGGATT
CCCGGTCCGGATAACGCAACCGAGACCCTCAACGAAGAGGAGTACGTGAGGGCATTACCGAGAGGCATCGGGCCA
AAGCCATTTGGCCTGAACTCTGAGGCATCTCGTGAGACTGCAGTGGTGATCATGAACCAGATGAACGTTGTGGAG
ATGCTCATGGATGTGGTATGAATCAATGCGCAGGCAAATTAAATGCTTTGTTCTTTCGATGCTTCGGAATATTCT
GTATGTGCCTAATTCTTTGCTTATTCTCCAGAATCAATGGTCAAGTGTGTTCTCCAGCATCGTGTCGAGGGCCAT
GACGCTCCAAGTGCTATCAACTGGAGTGGCAGGCAATTACAATGGAGCTCTGCAAGTGGTATGACTCTCTCCCTC
TAACATTTCCTTTTGCTATATATCAACTGGATTACTCTTCTGAGCAAATGATTGCTCGATCTATGAATCCTCAGC
TGTCAGCAGAATTCCAAGTGCCATCTCCACTTGTTCCAACTCGAGAGAGCTTGTTCGTGAGGTACTGCAAGCAAC
ACCCTGACGGAACCTGGGCGGTGGTCGATGTTTCCTTGGACAGCTTACGCCCCAGCCCCGTGTTGAGATGCCGAC
GACGGCCATCCGGCTGCCTCATCCAAGAACTGCCCAATGGCTACTCCAAGGTGAAGAACCAACCACACCAACAAG
ATCTACAGCGCACATTGGCCTTTTGAATCCCCAACTCTCCTAATGGAAACCTCTCTCTCTCTCTCTCCCTCTCTC
ATGGCGCAGGTTACTTGGGTGGAGCATGTTGAAGTGGATGATAGGTCTGTTCATAATCTATACAAGCCCTTGGTC
AACTCAGGCCTGGCATTTGGCGCAAAGAGGTGGGTGGGGACTTTGAACAGGCAATGCGAGCGCCTAGCTAGCGTG
ATGGCCAGTAACATACCCTCAGGAGACATCGGTGGTAACTATCTGCTCTATTTCGTATGATCGTTGATGCTATGG
ATGCCATCATATCCTCTGTGTCTTTGGTATATGTACAGTGATAACCACTCCAGAAGGCAGGAAGAGCATGTTGAA
GCTGGCCGAGAGGATGGTCATCAGCTTCTGCGGCGGCGTGAGTGCTTCAACTACGCATCAATGGACGACTTTGTC
TGGAAGTGGTGCGGAGGACGTCAGGGTGATGACCAGGAAGAGCGTGGACGATCCCGGTAGGCCTCCTGGTATCGT
CCTCAACGCTGCCACGTCCTTCTGGCTTCCCGTCCCACCGAAGAGGGTGTTCGATTTCCTCCGTGATGAGAGCTC
TCGTAGTGAGGTACTCGAGACTCATGGTCTCGGCTTCTCTTCTCCCATATGTCATGTTAGTTGATGTGCTTCTTG
TTGGTGCATTTGCAGTGGGACATCCTCTCTAATGGTGGTGTGGTTCAAGAGATGGCTCACATCGCCAATGGCCAA
GATCACGGAAATTGCGTCTCTCTGCTGCGCGTCAACGTAAGAAACGTCGGTGACGGTGCCACTTTACGATTTATC
TCTGCTCTCTTACACTCTGTGACGTACTTGATGGTGGTTGCAGAGCACAAACTCCAACCAGAGCAACATGCTGAT
ACTGCAAGAGAGCTGCACGGACGCCACGGGCTCGTACGTGATCTACGCCCCCGTGGACGTGATCGCCATGAACGT
GGTGCTCAACGGCGGCGACCCCGACTACGTGGCGCTCCTGCCGTCCGGGTTCTCCATCCTCCCCGACGGGCCGAC
CGGAGGAGGACCAGGCGGCGGGAGGATCAATGGGGGGGAGGAGGGGGGTGGATCCGGCGGTTCCCTCTTGACGGT
CGCGTTCCAGATCCTGGTCGACTCCGTGCCTACGGCCAAGCTCTCGCTCGGCTCGGTGGCCACGGTCAACAGCCT
CATTGCTTGCACCGTCGAACGAATCAAGGCTTCAGTTGCAGGCGAAGTTGCCCGCTGAGATCGGGGTAGTGTGAC
TCTGCTCGTCCCCATGCTTTTCTTCTTCTTCCGCGCTGCTGTTGTTGTATCTCATCTGCGTAGTGGTGGGGTTT

GGAGGCGTCCGTCATCTGTGGAAGTCAAGAACGCACCTCGTGCTTCCCTTGCCGGGTGGTGGCGGTGGTGGTGGT
GGTGTTCAGTTGACTCTGGAAACAAGTCAACAGCAGGCACTCTCCGCCGCCGCCGCGCAAGGAGGGGTTGGTTCG
GGCATTGACTTCCTCTCGTTTCAGCGTGTTGCAGGAACCACCGGCTCTCTTCCTCTCTCTCGTGCTCGCTAAGTT
GTGTTTCCTCTCTTCCCTGGAACAGACACATACTCCCATCGCCTCTCACCATTCAATTCCAATCCTCCCCATGTT
GTGTTGTTTGGTGTTGACTATATGTTGAGCTTATTTTAGTCTATGTTCCTTTTGCTTTCATCCTATTTTCTCATG
ATAATAACAAAAATTATAATAATAATTACAATG

CDS
ATGATACCAGCAAGACAAGCACCATCCATGATCGGGAGGAACACCGGCACCGGCTATGGCTCCTCGTCAGTGTTG
TCGCTCGGCCAGCCAAACTTATTAGAGGGGCAGCAAATCCCACTTCAGTACCAGCATCACAACCAGTTCATGGAG
ATCGCACCGACTACCACAGCGGAGAGCGAGATGCCAAGGGCTCGCGAGGAGGACTTCGAAAGCAAGTCCGGAAGC
GAGAACATCGAGGTTGCTTCCGGTGATGACCAGGACCAAAGCCATCGCCCGCGCAAGAAGCGCTACCACCGCCAC
ACACAGCATCAGATTCAAGAGCTGGAAGCCTTCTTCAAGGAATGCCCTCACCCGGATGACAAGCAGAGAAAGGAG
CTCAGCAGAGAGCTTGGGTTGGAGCCTCTCCAAGTCAAGTTCTGGTTTCAGAACAAGCGCACGCAGATGAAGAAT
CACCACGAGCGGAACGAGAACAGTCAGCTACGAACCGAGAATGATAAGCTCCGCGCCGAGAACCTGAGGTACAAG
GAGGCGCTCACCAACGCGTCGTGCCCCAACTGTGGCGGACCTGCTGCTCTAGGAGAGATGTCCTTCGACGAGCAC
CACCTCAGGATCGAGAATGCCCGGCTGAGAGAAGAAATCGATCGGATATCAGGGATAGCTGCCAAGTACGTGGGC
AAGCCAGTGGTGTCGTACCCGCTGCCCTCTCCTGCCATCTCTTCACGTTCGCCTTTGGATGTTGGTGTGGGCGGC
GAGATGTTCGGAGTCGGGGTCTCGGGCCAGACTGACATAGAAAAGCCAGTGGTCGTCGAGCTCGCGGTGGCCGCC
ATGGAGGAGCTCATCAGGATGGCCCAGCTCGGCGAGCCACTGTGGATTCCCGGTCCGGATAACGCAACCGAGACC
CTCAACGAAGAGGAGTACGTGAGGGCATTACCGAGAGGCATCGGGCCAAAGCCATTTGGCCTGAACTCTGAGGCA
TCTCGTGAGACTGCAGTGGTGATCATGAACCAGATGAACGTTGTGGAGATGCTCATGGATGTGAATCAATGGTCA
AGTGTGTTCTCCAGCATCGTGTCGAGGGCCATGACGCTCCAAGTGCTATCAACTGGAGTGGCAGGCAATTACAAT
GGAGCTCTGCAAGTGCTGTCAGCAGAATTCCAAGTGCCATCTCCACTTGTTCCAACTCGAGAGAGCTTGTTCGTG
AGGTACTGCAAGCAACACCCTGACGGAACCTGGGCGGTGGTCGATGTTTCCTTGGACAGCTTACGCCCCAGCCCC
GTGTTGAGATGCCGACGACGGCCATCCGGCTGCCTCATCCAAGAACTGCCCAATGGCTACTCCAAGGTTACTTGG
GTGGAGCATGTTGAAGTGGATGATAGGTCTGTTCATAATCTATACAAGCCCTTGGTCAACTCAGGCCTGGCATTT
GGCGCAAAGAGGTGGGTGGGGACTTTGAACAGGCAATGCGAGCGCCTAGCTAGCGTGATGGCCAGTAACATACCC
TCAGGAGACATCGGTGTGATAACCACTCCAGAAGGCAGGAAGAGCATGTTGAAGCTGGCCGAGAGGATGGTCATC
AGCTTCTGCGGCGGCGTGAGTGCTTCAACTACGCATCAATGGACGACTTTGTCTGGAAGTGGTGCGGAGGACGTC
AGGGTGATGACCAGGAAGAGCGTGGACGATCCCGGTAGGCCTCCTGGTATCGTCCTCAACGCTGCCACGTCCTTC
TGGCTTCCCGTCCCACCGAAGAGGGTGTTCGATTTCCTCCGTGATGAGAGCTCTCGTAGTGAGTGGGACATCCTC
TCTAATGGTGGTGTGGTTCAAGAGATGGCTCACATCGCCAATGGCCAAGATCACGGAAATTGCGTCTCTCTGCTG
CGCGTCAACAGCACAAACTCCAACCAGAGCAACATGCTGATACTGCAAGAGAGCTGCACGGACGCCACGGGCTCG
TACGTGATCTACGCCCCCGTGGACGTGATCGCCATGAACGTGGTGCTCAACGGCGGCGACCCCGACTACGTGGCG
CTCCTGCCGTCCGGGTTCTCCATCCTCCCCGACGGGCCGACCGGAGGAGGACCAGGCGGCGGGAGGATCAATGGG
GGGGAGGAGGGGGGTGGATCCGGCGGTTCCCTCTTGACGGTCGCGTTCCAGATCCTGGTCGACTCCGTGCCTACG
GCCAAGCTCTCGCTCGGCTCGGTGGCCACGGTCAACAGCCTCATTGCTTGCACCGTCGAACGAATCAAGGCTTCA
GTTGCAGGCGAAGTTGCCCGCTGA

GSMUA_Achr6P05990_001 (*MaHDZIV10*)

GATTTTAAATTTGAAAGTTAAAAAATACGATACTTATTATACTAGGTTGTCCCTTATTTAGAAGGATCAGAGAAG
AGGTTTGATCGAAATAATTAAGCAGATAAGGATGGTGTTAGAAAAATATAGTATGTACTTTCTTACATGATATGA
TATATTTTTAAAGATAATTTTTACCCAATAATTTTTTTTAAAAAAATTCTTAATCGATTAAGGTAAGATTACTTA
ACAGCTTTGAACTACCCACCCAATTGCTGATAAAATAAAAAGAAATAGCTGAGACATATTGTAAGGCCATGGCAA
GATCATTATATGGAGGCCAAGTTTTCATGCAGAGTCTCCACAATCTGATCTCAATCAAGGATGATGCTGGCCACC
ATAAATTTGTGCACCAACAATTTCTGCTGGGAGCCATTAAACCATTCCAACTCTGCTGAGCTGACACATAATCTT
GATTGCTAGCACTGACTACAGTAGTAGAAGTCACTCCCTCGTCACCGAATGGATCACCCACCTCTTCTCACTTCT
CACTTCTCCCCTCCCCCATGATGAACTTGGAGTTCCCATGTCACTGACTTGATTCCCAGATAGAGTGTGCTCATT
CATTATTTATTATCTCTCTTCTGGTTGCATACACTTACCTGTAATTTTCTTGCTTGCAGTGTTGGCGTGTGATCA
GAGTTTTCCGGTGTCGGAGAGTAACCAGATACATGAGAGAGAAGAATGAGAGCTGGCATATTAATAGTCTCTCCC
TCTGCTGTCCCATCTTTAATTCTCAAAGCTGTATCATTCCATAAGACAACCAAATTAATATTAGCTAAACATATC
AGCACAAAAGGAGAAGGTCACACCATTAAAAAAAAAAGGGGAATAATTTACATGTATATTTTTGCCAAACTACAT
CTCTATAGTTCTTCATATGAGGGAGGTATGAAAAGGTAGAAAAATAAAATTTAATTGGTTGTTATCTACACATAA
AATATGTCTTGCATATATAAATATGTGGGTTGAGGAGGAAGTAATACACCGTCAAAATCCGACATTTTCTAAGCC
CCGGCATATTAGATCGCGTCGGTTACTGTTGGCCTCGAAGACACCGTGAGAGGAAGGAGAGGCAGAGCCGAAAGC
TTGTGTGTGAGTCTTCATTAAGGGGGGAGGGAGGGGTCGAATCCGCCCGGATTGCTGCCGGTGTCTTTGGTGCGC

1641 GCACCGCCAGTTTCCTCATTTGCCTCTCGCCCGATCCCTTCACCCGGACAAGCACCATAAAGTGTGTGTTTGTGT
1642 GAGAGAGAGAGCAGCCGAAGCAGAGAGGAAAGGCGCCACCTTTAAGGACGAAGCATCGGGAGAACAGAGAGAGAG
1643 AGAGAGAGAGAGAGAGAGAGAGAGAGAGAGAAGAGGGCGTGAACAGGGGAACAGAGCGAAGCAGAGAAGAAAGACGC
1644 CGACTTGCATGAACTGGCTGCTTCTTTAGCGGATGGTAGTAGTTTAGAGCAAGTAGAAGTTGGAAGTTGGGCTCG
1645 ATGGATTCCGGCGACGAACAGGACGTCCCCGATGCCAAGGGGAGGAAGAAGCGGTACCACCGGCACACCCCGAGG
1646 CAGATTCAAGAGCTTGAATCGTATGTTCTCTCTCTCTCACTCTCTCTCTCTCTCTCTCGTATATCTGTACTGG
1647 TCGTTGTGTTGTTTTCTTCCCTGCTTTGAGGCTCATCATGGCGGCTTGTATCTGACGGGTTTGTCTGAGCTTGTT
1648 GTTTAGGATGTTCAAGGTTTGTCCGCACCCGGACGAGAAGCAGAGGATGCAGCTGAGCCGGGATCTGGCGCTGGA
1649 GCCGCGACAGATCAAGTTCTGGTTCCAGAATCGGAGGACGCAGATGAAGGCAAGCACTGCTCCTCCCTTGCTCTC
1650 TTTCCTCTTTTCTCTAGTGTTGTTGGTGGAAGGATTGGTCTTGTTTTGCGCGTGGGTCCGAAGAGGAGCCACCTC
1651 TGCCTCGTCTGATCGACCCTGCTGCTCTTCCCAGGCGCAACACGATAGGGCGGACAACTGCGTGCTGCGTGCCGA
1652 GAACGACAAGATCCGGGCCGAGAACATCGCCATGATGGAGGCTCTCAAGAGCGTCATCTGCCCCTCCTGTGGCGG
1653 CCCTCCCACCCATGAGGACTCCTACTTCGACGATCAGAAGCTGCGGATGGAGAACGCAAGGTTGAAGGAAGAGGT
1654 ACGCTCAATCTATCTTTCCTTTTAGTTACCTCCACCCTGCTATTTGGCTTGTATGCACTTGAGATATCTCTTCAG
1655 AAGAACAATGGTTCCACTGTTTGATACTGGAACAAAGGTAGATCCATCTCCGGCCAGGAGAAAGCAAAGCCATCA
1656 GCCTACTTGGTCATCTGTTCTCATCTTAGTGCTTGGTTGATATTGATAAGAAAACTCAAACCAGTGTGCTTTTGT
1657 CATGATGGATGAAGCCTCAATTGACACCATCTGCTTGGTTGTTTAGTTGGTCATCCCATTTCATATGCTTTTTTT
1658 TTTTTGGCTACTTCAGCATGGAGGAAACAGAACATTGTTTTGTATTGATCGATTGAAGTTTGCACTTGATTTAAA
1659 AAGGCAGTCTGCATCAATCGGATTCGATGATAGATTACATAAACATGCCTGCACATGATTGTTTAATACAAGACT
1660 ATGATGCTTCTTCCGAATTCTTCATGTTAATGCCCGCCTTGCAGCTTGATCATGTCTCGAGTATTGCATCAAAAT
1661 ACCTTGGAAGGCCTGTCACGCAGCTTCCCCCAGTGCAGCCAGTCTCTGTTTCATCATTGGACTTATCAGTTGGGG
1662 TTTACAGTGGTCCAGAGGTGAACCCTACCCTCGATCTTGATCTCCTGCGCCGGAATTGTTCTTCTGCTTTCCCGT
1663 ACCCATACACCCCAGCCGTTTCCGAGCTTGAGAAACCTCTCATGGTGGAGATGGCCACTGGTGCAGCGGAGGAAG
1664 TTCTCAGGCTTGTGCAGACCGATCAACCCCTGTGGGTGAAGTCCGGAAGTGATGGCAGGGACAGACTTCAGCTTG
1665 AAATCTACGACAGGATGTTCCAGAGGTCAGGCCAGCATCTCAGGTTCCCACACACTCGAACTGAGACGTCGAGGG
1666 ACTCGGCTCCTGTCGCCATGAATGCTATGACATTGATCGACATGTTCATGGATGCGAGCAAGTGGGCGGAGCTTT
1667 TTCCCAGCATTGTTGCCAAGGCGAGGACCATCGAGGTCCTCGCAGCCGGGATGGCCAGCAGCAGAAGCGGCTCTT
1668 TGGTGTTGGTAACGACAGCTCTCGATCCCTTTTTTGGTTTAATCCACTCACAGTACCCCTTCGATGATTCTAATA
1669 CACGCAAATGCATGTGCATGCAGATGTACGAGGAGCTGCAAGTTCTCTCACCTCTTGTTCCTACGCGCGAGTTCT
1670 GCTTTCTGCGGTATTGCCAGCAGATCGAATCGGGTATGTGGGTGGTGGCCGATGTCTCGGTGGACTATCCAAGAG
1671 ACAACCAGCTCGGCCTTTCTCCCCGATCAAGGAGGCTTCCTTCAGGCTGCTTGATCGAGGAAACGCCCGATGGCT
1672 ATTCCAAGGTGATTGATCTATGGATTTTGTGCTTCTGCTCCTTTATCTACCTTTTGACAGAATCACATCGATGAT
1673 TTTGATGTAGATTACTTGGGTCGAACACATGGAAATCGATGCAAATGATCAACCCCACGTCCTGTTCAAGGATCT
1674 AATCAACAGCGGGACGGCATTTGGAGCACAGCGCTGGATCACCACCCTCCGTAGAATGTGCGAGAGGTTTGCCTG
1675 CTCAAACGTCGCTGGTCTCCCGGGCAGAGACCTCGGAGGTAAGGCTTGGTTTTGCTTCAAGGTTTTACACCCAGT
1676 AGTTGAGTTGCTGATCGTTCGACGACTATGATTCGTACTGCGGAATGCAGTGGTTTCTTCCCCCGATGGTAAGAG
1677 GAGCATGATGAAGCTTGCTCAGAGGATGGTGAACAACTTCTGTGCCAACGTTGGTGCATCGAGCGATCAGAAATG
1678 GACGAACGTGTCAGGGTCGAACGACGTCGGCGTCAGGGTTGTGCTTCAGAAAACCTCAGATGCTGGCCAGCCCAG
1679 CGGCGTCGTCCTCTGTGCCGCAACCTCAATATGGCTGCCCGTGTCAGCTGAGCGGGTCTTCAGCTTGTTTAAGGA
1680 CGAGCGAACTCGCACTCAGGTGCCCATGCATGCAGCTTCTTGAGATGTCTACTTCGATCTCGATTGCTCTCTTTC
1681 TTACTTTGGGTATGTTATTGTTGTCATTAATCAGTGGCACATTCTATCAAATGGCAACACGCTACAAGAGGTGGC
1682 TCACATCACGAATGGCTCTCATCCGGGGAATTGCATCTCTCTTCTTCGTGTATGCTTTCTTTCTCTCGTGCATTC
1683 ATCTTCCTCGCCTTGGAACTCTCGTAAATAGGGTTTACTTGTGAATGAACAGGGGCTCAGTTCTTCCCAGAACAC
1684 CATGTTGTTACTCCAGGAGTGCTGTACCGACGCGTCAGGGTCGGTGGTGGTCTACTCCCCCATCGACCTACCGGC
1685 CATCAACATCGTCATGAGCGGCGAGGATCCATCCTACGTTCCGCTGTTGCCGTCGGGCTTCGCCATACTACCGGA
1686 CGGGCGATCTGCCGGGGGACAAGGAGCATCGTCCAGCTCGACTCCGATGGTCGGCTCATCTGGCTCGCTGGTGAC
1687 TGTCGCGTACCAAATACACTTGAGCAGCTTGCCGTCCGCCAAACTCAACATGGAGTCAGTAATGACCGTGAATCA
1688 CTTGATCGGCACCACAGTTCAGCAAATAAAGGTTGCATTGAACTGTGCGGATGCCTGATGCTCTCCCGAGCTACG
1689 TCTCCCCTCTTCTCTCTCCCATCAGCTCTTTCTTTACTGCTACAGATCTGATGGAGTCTCTTGAGGCCTTCGATG
1690 TGGCATTCAAGACGGGATTAAAGTGGGTCTTCTTGTGTTGGTTAGTTGGCCTTGAATGCTTCCCTCCATTGAGGG
1691 GGCTTCAATTCCAACTTTAAGATGCTGCTGCTGCTGCTGCTAGGCGCAGGACAATCTGATTCTCTTACAGGAACC
1692 CGTCAGTCTTTACATTTACAAGAACTTGATGAAGGAAGTCAAGAACGCACCATTGCCTGCTTCTCCTCTTCTGCT
1693 GCTGTCATAAATCCTACTTGTCCTTCAGGTTTCTTTCTTTAGGCCTGCTTCCTTCTCTCCTTTCCCTTGCTTCAA

TCCCATTCTTTCCTTTCTTCATGCAGCAAGTGGTGATGGTTCGGGCATTGACTTCTGTCCATAAACATTTACTGA
TGAGCATTTACTTTGAAGATGGTATCGATGGTG

CDS

ATGGATTCCGGCGACGAACAGGACGTCCCCGATGCCAAGGGGAGGAAGAAGCGGTACCACCGGCACACCCCGAGG
CAGATTCAAGAGCTTGAATCGATGTTCAAGGTTTGTCCGCACCCGGACGAGAAGCAGAGGATGCAGCTGAGCCGG
GATCTGGCGCTGGAGCCGCGACAGATCAAGTTCTGGTTCCAGAATCGGAGGACGCAGATGAAGGCGCAACACGAT
AGGGCGGACAACTGCGTGCTGCGTGCCGAGAACGACAAGATCCGGGCCGAGAACATCGCCATGATGGAGGCTCTC
AAGAGCGTCATCTGCCCCTCCTGTGGCGGCCCTCCCACCCATGAGGACTCCTACTTCGACGATCAGAAGCTGCGG
ATGGAGAACGCAAGGTTGAAGGAAGAGCTTGATCATGTCTCGAGTATTGCATCAAAATACCTTGGAAGGCCTGTC
ACGCAGCTTCCCCCAGTGCAGCCAGTCTCTGTTTCATCATTGGACTTATCAGTTGGGGTTTACAGTGGTCCAGAG
GTGAACCCTACCCTCGATCTTGATCTCCTGCGCCGGAATTGTTCTTCTGCTTTCCCGTACCCATACACCCCAGCC
GTTTCCGAGCTTGAGAAACCTCTCATGGTGGAGATGGCCACTGGTGCAGCGGAGGAAGTTCTCAGGCTTGTGCAG
ACCGATCAACCCCTGTGGGTGAAGTCCGGAAGTGATGGCAGGGACAGACTTCAGCTTGAAATCTACGACAGGATG
TTCCAGAGGTCAGGCCAGCATCTCAGGTTCCCACACACTCGAACTGAGACGTCGAGGGACTCGGCTCCTGTCGCC
ATGAATGCTATGACATTGATCGACATGTTCATGGATGCGAGCAAGTGGGCGGAGCTTTTTCCCAGCATTGTTGCC
AAGGCGAGGACCATCGAGGTCCTCGCAGCCGGGATGGCCAGCAGCAGAAGCGGCTCTTTGGTGTTGATGTACGAG
GAGCTGCAAGTTCTCTCACCTCTTGTTCCTACGCGCGAGTTCTGCTTTCTGCGGTATTGCCAGCAGATCGAATCG
GGTATGTGGGTGGTGGCCGATGTCTCGGTGGACTATCCAAGAGACAACCAGCTCGGCCTTTCTCCCCGATCAAGG
AGGCTTCCTTCAGGCTGCTTGATCGAGGAAACGCCCGATGGCTATTCCAAGATTACTTGGGTCGAACACATGGAA
ATCGATGCAAATGATCAACCCCACGTCCTGTTCAAGGATCTAATCAACAGCGGGACGGCATTTGGAGCACAGCGC
TGGATCACCACCCTCCGTAGAATGTGCGAGAGGTTTGCCTGCTCAAACGTCGCTGGTCTCCCGGGCAGAGACCTC
GGAGTGGTTTCTTCCCCCGATGGTAAGAGGAGCATGATGAAGCTTGCTCAGAGGATGGTGAACAACTTCTGTGCC
AACGTTGGTGCATCGAGCGATCAGAAATGGACGAACGTGTCAGGGTCGAACGACGTCGGCGTCAGGGTTGTGCTT
CAGAAAACCTCAGATGCTGGCCAGCCCAGCGGCGTCGTCCTCTGTGCCGCAACCTCAATATGGCTGCCCGTGTCA
GCTGAGCGGGTCTTCAGCTTGTTTAAGGACGAGCGAACTCGCACTCAGTGGCACATTCTATCAAATGGCAACACG
CTACAAGAGGTGGCTCACATCACGAATGGCTCTCATCCGGGGAATTGCATCTCTCTTCTTCGTGGGCTCAGTTCT
TCCCAGAACACCATGTTGTTACTCCAGGAGTGCTGTACCGACGCGTCAGGGTCGGTGGTGGTCTACTCCCCCATC
GACCTACCGGCCATCAACATCGTCATGAGCGGCGAGGATCCATCCTACGTTCCGCTGTTGCCGTCGGGCTTCGCC
ATACTACCGGACGGGCGATCTGCCGGGGGACAAGGAGCATCGTCCAGCTCGACTCCGATGGTCGGCTCATCTGGC
TCGCTGGTGACTGTCGCGTACCAAATACACTTGAGCAGCTTGCCGTCCGCCAAACTCAACATGGAGTCAGTAATG
ACCGTGAATCACTTGATCGGCACCACAGTTCAGCAAATAAAGGTTGCATTGAACTGTGCGGATGCCTGA

GSMUA_Achr10P04900_001 (*MaHDZIV15*)

TTTAGCTTTATAAGAATATATATATAAATACACTAAAATTTACAGGAGTAAATACAAAAAAAAATGATATACGAA
TATCGATATATAAATTTTTGCCAAAAAATGAAAAATAAAAAGATAATACATTGCATCGTAAGTTATATATGCTTT
TTCTTGAGGAATACATTTTTATATGACTTTTACAACCAAAAAAAGGTGGTAGGCGTATGTATGTATATACGTCAT
ATAGATTCGAGTTTTCTCTTTGCTCTCTTCTCCATAAATGCACTCTCAGAGGCCTTAAATGTTGGGAGTTGGAGG
GGGCTCCATAATATATATATACATATGTAAACGAAAATGGTAAACAGGAATGGAGATAATTAAGTCTCCTTAGCT
GGGAGACTTGACGATTTACCCTTCTTGATCACCTCCATCCGGCTTTGGAATCTTGCAGGTTCTCACTGCCACCAC
TTTGCAGTAATGAAAAGCCTAGCTTACCTCGAGGAGACACTCGAGAAGCTCTAGCTTTGCTCTTAGTTGTTAGCA
TTAATTGCAGAGCTCGTAAGTGTTGTGTGGAAATAGGATCATTTGTCATTATTTACCATGTCATTCAAATAAGTT
CTCCATTATGAAGTCGTACAAGATTCAAAGCTAGCACATTTAATTCGTAATAAAACACCAGTTCTGCTCAGGAAG
CATTTAATGCTTCATCTTTTCTCTTTTGAGTTTATCTTTTTCCTGTTAGATGATTAACAAAGGATATATATATAT
ATATATATATATATATATATATATATATATATATATATATATATATATATATATATATATATATATATANNNNNNNN
NNNNNNNNNNNNATATATATATATATATATATATATATATATATATATATATATATATATATATATATATATATATA
TATATATATATATATATATATATATATACACACATACATCAAAAGCTTTCACCTTCTCTCTTGTAGAATCATTTATA
GAATCCAGTCTCTGTGGTGCAGCCGAAGCAATTACACAACCTTCTCAATAAATGCAATTACACCCTATCAATACA
GTCTGCAATAACTCCTATGGAGTCCACCTCTCTCCTCTTCTCTCATCTCCTCTCCATCCCCTTCTCTTTTCTGTC
ATCCGTCTCTAAGAGCTTCTCTTGTGCTGCTTTCTTCTCAGAACCCACCATATTGTCTTACATGCATGCATTAAA
CCCACTATTACTTTAATTTCTCCCACAATTCTCGCCTTCTAAGAGGGAGAGAGAGAGAGAGAGAGAAGGAAGCGT
GAGAAGCAGTGCTGCTCGTCCTAGGACTACGTCTTGGTTGTCAACATTATTTGGCATTAAAAGCTATGCATCTCC
CTGTTCACCTCTGCTGTTTCTCGATATGTAATCCAGTTACAAGGTCTTCTCTAAGAACTTTTAGGGTTCTCCCAG
CCTAGAGATAAATAAAGAGAAAGAGAGGTGAGCCTAGGGTTTGGTGCAGAGGGAGGGGTTATCCCTGCAGGGATT
ATGATTCCTGCAACGCATGTGGCATCGATGATCGGGATGAACAGAAGTGCCGAGTATCAATCCTCGGCGGCTCTA

TCTCTCGGTCAGGTGAGCGAGTCTAAGCATTCTTGTTCTTTCTATTGCCTTTTAGATCATATATGTGATCTCCAT
GGTAGTACTAGGTTTTGCAGGACTGACTGAGAGAAAGAATGAGATCAGTTTCTTTTGTGCATGTTCTCTAGGTAG
AGTTTGGTAGAGTTAAAGATTTAAGGGTTTTTGGAGCCATTCGATGTCGAATCCTCCTCTCTATATGAGAAACAA
AGAGATGAAATGTCCGTTTGGTGGTCTTTCATCTCCCTCTACTTGTTGTCTTGTTCCCTTGTTGAGGAAGATGCT
ATGACACTTTTTTTTTTCCTGCGTATTTTGGAGTTTTTGAAGGGTTCTCTTCATCCTTTTTCAGTACCCAAAGTT
CTCTTGTGTTTTCTTGAGAAACAAATGGGAGGCTAAAACTGTAGGAGTGACATGTTGTCATCCCTTTTTCTTCTT
TCCTGTCAACTTCCCATCTTCTTCTTCTTCTTCTTCTTCTTCTTCTACGTCAAGTGCCCGTTTCTAACTTTTGTA
GCCAAATTTGATGGATGAACAGGAACCCCCATTTCAATACCAACATCAGCATCAGCTTGTGGAGATTCCGCAGAC
AGCGGTAGCTGAGATTGAGATGCCAACTGTTCGGGATGACGAGTTCGAGACCAAATCTTTTGGCGACAACATTGA
GAATGCCTCCGAAGACGATCGGGATGGGAACCAGCGGCCTCGCAAGAAGCAATACCACCGCCACACTCAGCACCA
AATACAAGAATTGGAGGCGTAATAACTCGTGCCTGTTCTTCCATTCCTTTATTGCTTTACATAAACTCTCCCAGT
GATCTAAGCGGGCATGCAAGTCTCTCTCTCTTTCTCAAGATAAAAGGAATAGGAGAAGAGAACATTTAATTCTAT
TTTCCACAATGTTTTTATTTCTCTTTTAATCTCATTAAATATTACCTCTTACAACTTTTGGAAATGCTTTCTGTT
TGGGGAAGAAAAAGAACATGCCTTTGTTTCCTCTCAGTTTCTTCAAGGAGTGTCCTCACCCGGATGACAAGCAGA
GAAAGGAGCTGAGTCGCGAGCTTGGGCTGGAGCCTCTCCAAGTCAAGTTCTGGTTCCAAAACAAGCGCACCCAAA
CCAAGGTTTAACCCTAAACCTCCTTGAATCGAATTCATGCAAATGACCAGAACACTTTTTGCCACCGCTGTGTTA
GTCTGGTTATCAGTAATGCTCAGTAGTCGATCTCTCTCTCTCTCTTTCCTATTTGGTTCGTGACGTAGAATCATC
AAGAGAGGCACGAGAACTCGCGGCTGCGGGCTGAGAACGAGAAGCTCCGGGCCGAGAACTTGAGGTACAAGGAGG
CACTAAGCAATGCGTCATGTCCCAATTGTGGTGGCCCTTCGTCTCTAGGAGAGATGTCCTTCGATGAGCACCAAC
TCAGGATCGATAATTCTCGTCTGCGCGAGGAGGTTTGTATCTGTTCTTGATCTTTACATGACCTTCGAGTGCTCG
GGTACTTCATCGAACGTCTAAAAATGCCGTTGAAATGCCTGCTAGATTGGTAGATTATCTGCCATAGCCGCCAAG
TACGTCGGCAAGCCCGTAGTTCCTCACCAGTTATTCTCTCCGATGGCTGAGAGCGACATGCTTGGGGCAGGAGAC
TTACTCGGGAGCATGTTCGGCCACAGGGAGATCGAGAAACCCGTCGTTATCGAGCTCGCAGTGGTTGCCATGGAG
GAGTTAACAAGGATGGCGCGACTCAGTGAGCCTCTATGGACGATGAAACATGGTGACTCATTCGAGATACTGAGC
GAAGATGAGTACGTGAGGAACTTCCCTAGAGGAATTGGGCCAAAACCCTTGGGAATGAAGTCGGAGGCGACGCGT
CAGACCGCGGCGGTCATCATGAATCGTGTCAACCTGGTTGAGATGCTTATGGATGTGGTAAGAGAGTTACAGTTT
TAGGAAAGCTTGTGATGAAATTGATTCAAGTTGCTTTACTTATTTGTCCATTTCACCTTGACGATGAACAGAATC
AGTGGTCGAATGTGTTCTCCGGCATTGTATCGAAAGCTATCACGCTCGAGGTGCTATCCACTGGAGTAGCAGGAA
ATTACGATGGAGCTCTACAGCTGGTATATATAGTAGCTTCATATACTGTATCTTCTTACACTTTGCAGTTGTTCC
AAGCGAACAAGGATGAAACTTCTGATCTTGAATTCTCAGATGACAGCGGAATTCCAAGTACCATCTCCTCTTGTT
CCAACCCGGGAGATCTTGTTCGTCCGGTACTGCAAACACCAAGCTGACGGAAGTTGGGCGGTGGTTGATGTTTCC
TTGGACACCTTACGCCCTCCTCTGGTTGCAAGGTGTCGAAGAAGGCCATCTGGCTGCCTGATTCAAGAGATGCCT
AATGGATACTCGAAGGTAAAAGAACCTGTAAACCACAAAACAATGAGATGCTGCTTCTGATTCTTATCACATGTA
TGATTCATTCCCCGTCGTGCAGGTTACTTGGGTTGAGCATGCTGAGGTTGACGATGGATCTGTTCATGACATATA
TAAGCCCTTGGTTAACTCGGGCCTGGCATTTGGTGCAACGAGGTGGATTGGTTCCTTGGATCGCCAATGCGAGCG
CCTCGCAAGTCTAATGGCAAGCAACGTTCCCTCTGGGGATATCACTGGGTAATGAGCTTTTCTTGTTTCTATTCAC
CTTCATGCTACAGCTAATATAGCGGAATGCAAATAAGTGGAGATGTTTCTGTGGTGTCGCAGTCATAACTACAGC
GGAAGGGAGGAAATGCATGTTGAGGTTGGCTGAGAGGATGGTGACGAGTTTTTGTGGTGGTGTGAGTGCCTCCAC
CGCTCATCAATGGACTACGGTATCTGGTAATGGTGCAGAGGACGTGAGGGTGATGACCAGAAAGAGCGTTGGCGA
TCCTGGCAGGCCTCCCGGCATCGTCCTCAACGCTGCTAAATCCTTCTGGCTTCCTGTCCCGCCGAAGAGGGTCTT
CGATTTCCTCCGCGACGAACGATCTCGCAACGAGGTAAACACAATCTTATCACTTCACAATCTAAGTAGATTTCT
TACCTCTCAGTTTTGGTGCATCGACAGTGGGATATCCTTTCAAATGGTGGTGGCGTTCAAGAAATGGCTCACATC
GCCAATGGCCGAGACCACGGCAACTGCGTCTCTCTTCTCCGAGTCAATGTAAGCTTATAAACCGCTTGTGGAGTA
CATTGATAGTCCCAGTTTCTTGCACTGGACTAACTGATGCTGATCTATTAGAGTGTGAAGTCAAGCCAGAGCAAC
ATGCTGATACTGCAAGAGAGCTGCACGGATCCGGTCGTCTCCTACGTGATCTACGCGCCGGTGGACGTGGTCGCC
ATGAACGTGGTGCTTAATGGTGGCGATCCCGACTACGTTGCTCTCTTGCCCTCCGGCTTTGCCATCTTACCAGAT
GGCGCTTCTGCAGCGCAAGGTAGCCTCGTCACTGTTGCATTCGAGATACTGGTCGACTCGGTCCCGACCGCGAAG
ATCTCGCTCGGCTCGGTCGCCACCGTTAACAGCCTCATTGCGTGCACCGTTGAACGAATCAAGGCTGCATTGGTT
GGCGAAAACGTTGCATGAGTCTGAATCATTTGGGTAAGAGAGAATGGTTTTGCCTGCTTCCAATTCATAATT
GGTGGCTCCACATCTGTGGGTGGTCTTGTTGAACAGAGTTTCCATGTAGCTACAGAGGCCTTAGGGTTTATATTA
TGCTATCTCAAGAAAAGAATCATGTCATGACTTGGTACTGAGTAGCTTAGAATTATTGGTAATTTTGTCTTGAGC
CCTTGAGATGATGAAGGGTTTGATTTTGTTTTCCTTGATCTGTTTAGTTATTTTTTCTTATGAGATGCTCCTAAA
TGCAGTTTTAGTTGTAGAGAGAGGTTGTGATGGTCTCAAAATTTCAGTAAGAAAATAAATCTGTCCTGTGGACGT

GTTCTTGAAAAAGTCAAAGTTGTTCATATGCTCAAAGCATACTGTGGCGGCCCACCGCGGCCGTGGGTTTCCCCC
ACGTGGGCCGAACCATCGCCTCCATGGGAAGGCCGCAATCACTCGTCTTCGCGCTCTCTGAATGGGTTTTC

CDS
ATGATTCCTGCAACGCATGTGGCATCGATGATCGGGATGAACAGAAGTGCCGAGTATCAATCCTCGGCGGCTCTA
TCTCTCGGTCAGCCAAATTTGATGGATGAACAGGAACCCCCATTTCAATACCAACATCAGCATCAGCTTGTGGAG
ATTCCGCAGACAGCGGTAGCTGAGATTGAGATGCCAACTGTTCGGGATGACGAGTTCGAGACCAAATCTTTTGGC
GACAACATTGAGAATGCCTCCGAAGACGATCGGGATGGGAACCAGCGGCCTCGCAAGAAGCAATACCACCGCCAC
ACTCAGCACCAAATACAAGAATTGGAGGCTTTCTTCAAGGAGTGTCCTCACCCGGATGACAAGCAGAGAAAGGAG
CTGAGTCGCGAGCTTGGGCTGGAGCCTCTCCAAGTCAAGTTCTGGTTCCAAAACAAGCGCACCCAAACCAAGAAT
CATCAAGAGAGGCACGAGAACTCGCGGCTGCGGGCTGAGAACGAGAAGCTCCGGGCCGAGAACTTGAGGTACAAG
GAGGCACTAAGCAATGCGTCATGTCCCAATTGTGGTGGCCCTTCGTCTCTAGGAGAGATGTCCTTCGATGAGCAC
CAACTCAGGATCGATAATTCTCGTCTGCGCGAGGAGATTGGTAGATTATCTGCCATAGCCGCCAAGTACGTCGGC
AAGCCCGTAGTTCCTCACCAGTTATTCTCTCCGATGGCTGAGAGCGACATGCTTGGGGCAGGAGACTTACTCGGG
AGCATGTTCGGCCACAGGGAGATCGAGAAACCCGTCGTTATCGAGCTCGCAGTGGTTGCCATGGAGGAGTTAACA
AGGATGGCGCGACTCAGTGAGCCTCTATGGACGATGAAACATGGTGACTCATTCGAGATACTGAGCGAAGATGAG
TACGTGAGGAACTTCCCTAGAGGAATTGGGCCAAAACCCTTGGGAATGAAGTCGGAGGCGACGCGTCAGACCGCG
GCGGTCATCATGAATCGTGTCAACCTGGTTGAGATGCTTATGGATGTGAATCAGTGGTCGAATGTGTTCTCCGGC
ATTGTATCGAAAGCTATCACGCTCGAGGTGCTATCCACTGGAGTAGCAGGAAATTACGATGGAGCTCTACAGCTG
ATGACAGCGGAATTCCAAGTACCATCTCCTCTTGTTCCAACCCGGGAGATCTTGTTCGTCCGGTACTGCAAACAC
CAAGCTGACGGAAGTTGGGCGGTGGTTGATGTTTCCTTGGACACCTTACGCCCTCCTCTGGTTGCAAGGTGTCGA
AGAAGGCCATCTGGCTGCCTGATTCAAGAGATGCCTAATGGATACTCGAAGGTTACTTGGGTTGAGCATGCTGAG
GTTGACGATGGATCTGTTCATGACATATATAAGCCCTTGGTTAACTCGGGCCTGGCATTTGGTGCAACGAGGTGG
ATTGGTTCCTTGGATCGCCAATGCGAGCGCCTCGCAAGTCTAATGGCAAGCAACGTTCCCTCTGGGGATATCACT
GTCATAACTACAGCGGAAGGGAGGAAATGCATGTTGAGGTTGGCTGAGAGGATGGTGACGAGTTTTTGTGGTGGT
GTGAGTGCCTCCACCGCTCATCAATGGACTACGGTATCTGGTAATGGTGCAGAGGACGTGAGGGTGATGACCAGA
AAGAGCGTTGGCGATCCTGGCAGGCCTCCCGGCATCGTCCTCAACGCTGCTAAATCCTTCTGGCTTCCTGTCCCG
CCGAAGAGGGTCTTCGATTTCCTCCGCGACGAACGATCTCGCAACGAGTGGGATATCCTTTCAAATGGTGGTGGC
GTTCAAGAAATGGCTCACATCGCCAATGGCCGAGACCACGGCAACTGCGTCTCTCTTCTCCGAGTCAATAGTGTG
AAGTCAAGCCAGAGCAACATGCTGATACTGCAAGAGAGCTGCACGGATCCGGTCGTCTCCTACGTGATCTACGCG
CCGGTGGACGTGGTCGCCATGAACGTGGTGCTTAATGGTGGCGATCCCGACTACGTTGCTCTCTTGCCCTCCGGC
TTTGCCATCTTACCAGATGGCGCTTCTGCAGCGCAAGGTAGCCTCGTCACTGTTGCATTCGAGATACTGGTCGAC
TCGGTCCCGACCGCGAAGATCTCGCTCGGCTCGGTCGCCACCGTTAACAGCCTCATTGCGTGCACCGTTGAACGA
ATCAAGGCTGCATTGGTTGGCGAAAACGTTGCATGA

GSMUA_Achr5P14100_001 (*MaHDZIV8*)

TATATTGATATATCTATAATTATAAAATTAAAATATTCAGATCTATTTACCCTACCTAACATCATCAATTTAATC
AATAAAAAATAGAATTTTAATGTTATAATCAACGATGATGAATTACAATACTAGCGAGCAATGAAAGGATTGTCAA
TGAAGATGTCAAACGATACCGGTCTATATCAGCATCGATGTCATCCGTCATCATCAATTAGAATATTAATTTTTT
TTTATATATTTTTATTAGTAAAATCAATTATGTTAAGATAAATGGATCAAATATTTTAGTTTCGTAATTATATAG
ATGTCAATATAATTTTTAAAATATAAGGATAAAAATACTAGAGGTAGGTAACTGATAACACGTAAATACTACGTC
CAATATATCAGCTATACGGCTGTGTAATGCCAATGTCCTTTACGGGCATTATAATCGAGGTTGGCGTCCAGAAGC
GTGTGAAGCGTCGCTTCGCCTTCCATCTACGTCACACATGGCGCCACATGGAGGACACCTCATTCTCAAAACCCC
ATCAACCTTCCTTGCGACTGTAGCTGGCGCCGGGACCCACCAATCTCAAGGGCAGCACTCTTGTCGAGTCAAAGC
TGGCGCGACGTTCCTCTTCGAATCCAACGCCAGATCGAGCGAATCGGAGCCGCTTCATTCGGTGGACCCCACCGG
CGTGGTGGGTCTCACATAAGACATTTGGGAGACGGCGGCAAAAAGCGAAAGCGCGTGATGGGGGCGAGAGAGAGA
GGGAGGGAGGGAGAAGGGGGAGGGTTGACTCGCGGGGCAGGGGAGAGAACGCTGCTCCGGAGGCGACGAAAGACA
CGTGTCCGTTACGCCAGGCGGAGCAGATTCCCTGGCGGGGAATCGGTTTCTGCTGCCCGCAGTTAAAGTCGCATT
TACCACCACGCGCTCTCTCTCTCTCCCTCTGAAACCTCCCTCCCACTAATTACCCCCACCGGTAATAAATAATTA
CTACCAATAAAGGTTTTAGTGAAAGAGAGTGAGTCTTCTTCTTCTGATGGAGAGAGAAAGAAGAGTTAATACCGT
TTGTCTGTGAAGCAGCACAGAGAGAGAAAGAGAGCGGGGAAAGAAGAAGAAGAAGAAGAAGAAGAAAAAGCTTAA
GTGTAGGTCCACGAAGAGGGAGAAGACAGCAGGTCTTCTGTCTTGTTTTCTCCTCTCTCTCTCTCTCTCTCTCCC
AGAAACACCGACAGTGGTTAGTGGCATTCATTTCTCGCCATTACTGCACTTTCCTCTATCCCATTCCTCCTACCT
TTCTTTCTTTCTTTCTTTCTTTCTTCTCAGATCTCCCCTTCCTTCTTCATTCTTTTGTTTTCCTCGTGTCGTTGT
ACTCTCATTCTTCGACGATCTCTCCATCGACCGTGTTTGGTTCTGTTTACTTGAAGATGGACGAAGTGTGGTTCT
GAGAAAGGCAAAACAGTCGAGTGTCTTCTTGTTTCGAGGGCAAAAGAAGTGGAGGTCAAGAAAGAGAGGCTAAAG

ATGTATGGAGACTGTCAAGTGCTGTCATCCATGGTGGGAGGCAACGTTGTGTCACCCGATTCGCTCTTCTCCTCT
TCGATCCAAAATCCTAGCCTCAGCTTCATGGCTAACATGCCACCATTCCATGCCTTCTCCTCCATCATTCCGGTA
AGCACTGATCTCTTCCTATACCTTACCCTACGCGTTCTCAAAACATTGTCGCTAAGATTTGGTTGTGGTGGCGTA
AGAAGGAGGAAGGGATGATGTTGATGGGGAGGGGTGGGAGCAAGGAAGAGGAGATGGAGAGCCGCTCTGGTAGCG
GACCCCTGGACGGAGTGCTCAGCTGCGGCGAGGACCACGACAACGAGTTGCAGCAACCGCCGCCGCCGTCGCAAC
TGCAGCAGACGGCTGCGAAGAAGAAGCGTTACCACCGCCACACCGCCCGGCAGATTCAAGAGATGGAAGCGTGAG
ACTCTCACCACATCCATCTCTGTTAATGATATGATATGGTATGATGTGATATGATTTGATGGCCATTAATAGGAT
GTTTAAGGAGTGCCCTCACCCGGACGAGAAGCAGAGGATGAAGCTAAGCCATGAACTCGGCCTTAAGCCTCGCCA
GGTCAAGTTCTGGTTCCAGAACCGCCGGACTCAGATGAAGGTGCGTCTGCAACCCTTCATTCATGCATCAGTACC
AACAATGATACAAGAATTAGATGCTATTCGTTGTGGAAGAAAGCTTACTGATATGCATGGGATTGTAACTGCGGC
ACGCTGCAGGCTCAACAAGACCGGGCGGACAACGTGGTGCTGCGGGCGGAGAACGAGAGCCTGAAGAACGACAAC
TTCCGGCTGCAGGCAGCCATTCGCAACGTCGTCTGCCCCAGCTGCGGCGGCCCGGCCATCCTCGGCGAGATGTCC
TTCGACGAGCAGCAACTGCGCATCGAGAACGCCCGGCTCAAGGACGAGGTACTGAACCCTAACCCTAGCTCCTCC
CAGAACGACGTTACCTCCACATGCACTTGCTCTTGCTATCATTTCCTCTGTTCACCACTTCAATGTGCAGCTTGA
GCGTCTATCCTGCATCGCGTCGCGTTACAGTGGCCGGCAGTTGCAGCCATTAGGGCCGGCACCGCCTCTACTGCT
CCCCTCTCTTGACCTTGACATGGGCATCTACTCCAGACACTTCCACGAGCCGCCGGTGGTGAGCTGCACCGACCT
GATCCCCATCCCGCAGATCTCCGATGAGCCCTCGCCGTTCCCTGGCATGCTCATCATGGACCAAGACAGGCCATT
GGTTCTCGACCTGGCAATGACGGCAGCAGATCACCTGGTCAGGATGTGCAACACGAACGAGCCGCTGTGGATCCG
AAGAGGTGGTAGCACCGTGGAGGTCTTGAACTTGGAAGAGCATGCGAGGATGTGTCCATGGCCGATGGATCTGAA
ACAGCAACAGGGGCGGTTTAGGACGGAGACTTCCAGGGACAGTGCCATGGTCATAATGAACGGCATCACAATGGT
GGATGCCTTCCTGGATGCAGTGAGTCAATCTTTTCCTAGATCCATGAATCGTCTCTGTAGCATCATGAGCTCTTA
CCCTCTCGGTTACTTTGTCCAGAACAAATGGATGGAGCTGTTTCCTTCCCTTGTCGCAAAGTCCAGAACGGTGCA
GGTTTTGAGTCCCGGAGTTCCCGGCCACGGCAATGGCTGCTTGCATCTGGTAATTTGTCGGATCACCTCCATGAA
TACATGTTGTTGTTGGCATCTGCTTTGCTCATGTTCGGGCTATTAACAGATGCATGCAGAGCTGCAATTTCTATC
TCCGTTGGTGCCCGCCCGTGAAGCTCACTTCTTCCGGTACTGCCAGCAGAACTCAGAAGAAGGCACTTGGATTAT
AGTCGACTTCCCCGTTGATGGATTCCGAGATGGCATTCAAAGCCCCTTCCCCTGGTATAGAAGGAGGACTTCTGG
TTGCGTCATCCAAGATATGCCCAATGGGTACTCCAAGGTACGGAAACTTGATTTGCTGTGTTTATGTATCACCGG
GACAAGAAAAAGCGCTTCTATGTTCTGGGAATGTTGCTCACCTGCCATGATCACTTCTCTAATAGTAATAGAGGA
CATGGTTAAGAATTTTATGGAAGAACACAGGAGAAAGATAACAAGAACAAATCAGAGTTACCTGCTTTATACCTC
ATATTTCTTTGGGCAGGTGATCTGGGTAGAACATGCAGAGGTGGAGGACAAACCAGTGCATCAGATATTCCAGCA
ATTTGTCAGCGCCGGTGAGGCATTCGGAGCTACACGCTGGGTTTCAGTCCTACAAAGGCAATGTGAGCGGCTCGC
GAGCCTTATGGCGAGAAATATTTCTGACAATGGAGGTATCTCTGCCGATTCATCCCCACAGAAAAGTCCTACACT
GAACTGTGTTGCGGTTCTCTCATCCGTGTACTCCGGTGTTTCAGTTATCTCATCTCCGGAGGCGAGAAAGAACAT
GATGAGGCTGTCACAGAGAATGATAACAACATTCTGCACCGGCGTGTATGCCTCTGGAATGCAGTCATGGACGGC
GCTCTCGGATTCTTCCGATGACACGGTCCGGGTGACAACTAAGAAAAATACTGCACCCGGCCAGCCAAATGGGGT
TATTCTCACGGCAGTGTCCACCACATGGCTTCCTTCCTCCCATCACCAGGTGTTTGAGCTTTTGACGGACGAGCA
AAGGAGGTCTCAGGTACTTCGTGGTTTGTTGTTTACTGCAATCTGCATCTTTGTTTGTGTCTTCAGGTAGCTACC
TTCTCGTGTCTCGTTCAGCTGCAGTTGGATGTGCTCTCCAGCGGGAACTCGTTGCATGAGGTGGCTCATATAGCC
AATGGCTCGCACCCGAGGAACTGCATATCACTGTTGCGTGTCAATGTCAGTATGCCCTCCCACGCACTTCATCCT
ATTTTGCTTTCGATTGCCGATTGAGATATTTTCTACGTGCATGTGTAATCCACTCGCTCAGGCAGCAAGCAACTC
CTCCCACAGCGTGGATCTCCTGCTCCAAGAGAGCAGCACCCACCCCTCCGGTGGCAGCATCGTGGTGTACGCCGC
AATCGACGTCGACGCCGTGCAGGTGGCGATGAGCAGCGAGGACCCGTCCTACATTCCCCTCCTCCCGACCGGGTT
CGTCATCTCGCCCGCCGCCCGCCAACCAAACGCTGGGACCGGTAGCGGCAGCGATGGTCACGCCACCGTCGGATG
CCTGCTCACGGTCGGCATGCAGGTGCTCGCCACCGCCGTCCCCTCCGCCAAGCTTAACCTCTCCACCGTCACCGC
CATCAACAACCACCTCTGCAACACCGTGCAACAGGTTAGAGCCGTCATCGCCGGTGCCGGTGGCACGGCCATGGC
CGAGCCGGCTGCCGTGGCGCCGGATCAATAACACGAGGAAGTGACTGGAGTTACCTGAACCCTAAGTACTGAACC
ATTAAACCTCGTGAACATGTCTCTTCAGTGACGATGACCCCAGTGGTCGTGTTGGCGAAGTAGGAAGGCGGGGTT
CAAGGTAAACTCCAAGGATGGTAGTGGAGTCTGTAGAGTGTCTAGTTCAAGTGGCAGCGTCGTACTGTAACATGT
CAGTGGAAGAAGAGAGGCCAAGGAGACTAGTTAGGTTGAAGGAGGGAAGTCAAGAGCGCACCAGATGTGACCCTT
GCTCGCTGGCGAAGCCCATCTCACGAGGGGAGGAGGGCGTTGGTTCGGGAATTGACTTCTTGCTCTGTGGTGGTC
AGCTTCCTGTTGGTTTGTGTTTGTGTTTGTGTTTCTGTTGAGTTGCCTCGGATGGGGGTTTGGTTGGTAGAACTG
GATTGAATGATGCATGCTGTGCTGGAACCATTATGGTTATTTATGTTGTGTATTCATTGCTCTGTTTGCCTATCT
TCCTTTTTCTT

CDS

ATGTATGGAGACTGTCAAGTGCTGTCATCCATGGTGGGAGGCAACGTTGTGTCACCCGATTCGCTCTTCTCCTCT
TCGATCCAAAATCCTAGCCTCAGCTTCATGGCTAACATGCCACCATTCCATGCCTTCTCCTCCATCATTCCGAAG
GAGGAAGGGATGATGTTGATGGGGAGGGGTGGGAGCAAGGAAGAGGAGATGGAGAGCCGCTCTGGTAGCGGACCC
CTGGACGGAGTGCTCAGCTGCGGCGAGGACCACGACAACGAGTTGCAGCAACCGCCGCCGCCGTCGCAACTGCAG
CAGACGGCTGCGAAGAAGAAGCGTTACCACCGCCACACCGCCCGGCAGATTCAAGAGATGGAAGCGATGTTTAAG
GAGTGCCCTCACCCGGACGAGAAGCAGAGGATGAAGCTAAGCCATGAACTCGGCCTTAAGCCTCGCCAGGTCAAG
TTCTGGTTCCAGAACCGCCGGACTCAGATGAAGGCTCAACAAGACCGGGCGGACAACGTGGTGCTGCGGGCGGAG
AACGAGAGCCTGAAGAACGACAACTTCCGGCTGCAGGCAGCCATTCGCAACGTCGTCTGCCCCAGCTGCGGCGGC
CCGGCCATCCTCGGCGAGATGTCCTTCGACGAGCAGCAACTGCGCATCGAGAACGCCCGGCTCAAGGACGAGCTT
GAGCGTCTATCCTGCATCGCGTCGCGTTACAGTGGCCGGCAGTTGCAGCCATTAGGGCCGGCACCGCCTCTACTG
CTCCCCTCTCTTGACCTTGACATGGGCATCTACTCCAGACACTTCCACGAGCCGCCGGTGGTGAGCTGCACCGAC
CTGATCCCCATCCCGCAGATCTCCGATGAGCCCTCGCCGTTCCCTGGCATGCTCATCATGGACCAAGACAGGCCA
TTGGTTCTCGACCTGGCAATGACGGCAGCAGATCACCTGGTCAGGATGTGCAACACGAACGAGCCGCTGTGGATC
CGAAGAGGTGGTAGCACCGTGGAGGTCTTGAACTTGGAAGAGCATGCGAGGATGTGTCCATGGCCGATGGATCTG
AAACAGCAACAGGGGCGGTTTAGGACGGAGACTTCCAGGGACAGTGCCATGGTCATAATGAACGGCATCACAATG
GTGGATGCCTTCCTGGATGCAAACAAATGGATGGAGCTGTTTCCTTCCCTTGTCGCAAAGTCCAGAACGGTGCAG
GTTTTGAGTCCCGGAGTTCCCGGCCACGGCAATGGCTGCTTGCATCTGATGCATGCAGAGCTGCAATTTCTATCT
CCGTTGGTGCCCGCCCGTGAAGCTCACTTCTTCCGGTACTGCCAGCAGAACTCAGAAGAAGGCACTTGGATTATA
GTCGACTTCCCCGTTGATGGATTCCGAGATGGCATTCAAAGCCCCTTCCCCTGGTATAGAAGGAGGACTTCTGGT
TGCGTCATCCAAGATATGCCCAATGGGTACTCCAAGGTGATCTGGGTAGAACATGCAGAGGTGGAGGACAAACCA
GTGCATCAGATATTCCAGCAATTTGTCAGCGCCGGTGAGGCATTCGGAGCTACACGCTGGGTTTCAGTCCTACAA
AGGCAATGTGAGCGGCTCGCGAGCCTTATGGCGAGAAATATTTCTGACAATGGAGTTATCTCATCTCCGGAGGCG
AGAAAGAACATGATGAGGCTGTCACAGAGAATGATAACAACATTCTGCACCGGCGTGTATGCCTCTGGAATGCAG
TCATGGACGGCGCTCTCGGATTCTTCCGATGACACGGTCCGGGTGACAACTAAGAAAAATACTGCACCCGGCCAG
CCAAATGGGGTTATTCTCACGGCAGTGTCCACCACATGGCTTCCTTCCTCCCATCACCAGGTGTTTGAGCTTTTG
ACGGACGAGCAAAGGAGGTCTCAGTTGGATGTGCTCTCCAGCGGGAACTCGTTGCATGAGGTGGCTCATATAGCC
AATGGCTCGCACCCGAGGAACTGCATATCACTGTTGCGTGTCAATGCAGCAAGCAACTCCTCCCACAGCGTGGAT
CTCCTGCTCCAAGAGAGCAGCACCCACCCCTCCGGTGGCAGCATCGTGGTGTACGCCGCAATCGACGTCGACGCC
GTGCAGGTGGCGATGAGCAGCGAGGACCCGTCCTACATTCCCCTCCTCCCGACCGGGTTCGTCATCTCGCCCGCC
GCCCGCCAACCAAACGCTGGGACCGGTAGCGGCAGCGATGGTCACGCCACCGTCGGATGCCTGCTCACGGTCGGC
ATGCAGGTGCTCGCCACCGCCGTCCCCTCCGCCAAGCTTAACCTCTCCACCGTCACCGCCATCAACAACCACCTC
TGCAACACCGTGCAACAGGTTAGAGCCGTCATCGCCGGTGCCGGTGGCACGGCCATGGCCGAGCCGGCTGCCGTG
GCGCCGGATCAATAA

GSMUA_Achr11P21700_001 (*MaHDZIV19*)

TTACTATTGATTGCATCATGCAAATTATATGTCTTTTGATAACTTATTTAATAAATAAATAATAATGTTTTCAGC
ATATCTTTGTATTCATTGGACTTGCAACAAGGCTTTGATGATATATTCTCCTAAGGTACCGAGTTTAATTTTATA
TTACATATATATATTTTTTATGGGATTTTGTGATCTTGGCCGGTCCATTTCATTTATGGCTTGACCAGTGTCGAC
AAGCTTCTCTTCTTCCACTCGAACAGCAGCAGCAGCAGCAGCAGCAACAGCAGCAGCGAGTAGGCTATCAATAAA
TGCAATTGCACCCGCTATACCTTCCGCAATAACTCCTCCGCCCACCGCTCCCTTTTTCCTCCCCTTCCTCCTTCC
TCCTTCCTCCTCGCGGCAGTCTCCCTCCTCTTAAGATTCCAGTTTCCGTTTCCAAACAATACTGCACGAGTCAAG
AAATGAGAGATAGAGAGATTTAGGAGCGCTCTCCCCTCTCCTCAGAAACACTTGGAAGCCGCCACCGATGCATTC
TTAAACGGATGGCAGTTTTGTCTCGCGACTTCTCCCTGCAGCATCCAAGCTAGGTTTCGATCGGAACAAATATGC
TGAGAAAGAATAATGATCCCGGCAACCAGATGGAATCATCACCGGTTTCTAGGAGAGGGTATGAGGGTGCCTTCC
CAGCTTTGGCACTTGGTCAGGTCTGTAAGCTACACTAGATACTCAAAGATAAGCCGTTATATTTTTCTCTATCTT
TCTTTGGACTTTAGTGCAACGATGCCATGAGGGTACATGTTCTGAGAGAAGGTGGCATCGAGTGAGAACAAAGTC
GTGGGTGCATGCACCCGCTTCGCAAAGCTACCTGCCCCTCTGATTCGCCCTCTCCATCGCATTGCAGCTTGTTAT
CTTTGAGGGTTGATCTTTGCAGCCTATATAGCAGCCAGCAATGGCGGCCGTAGCGAGTGGCTACTGGTTGGTTAT
GAGGGGTTTGAGAGAGTTTCGCTAGCTAGAGGCTGTGGAGTTTAGGGAAATGGAACAAGGTTGAGCTCTTACATT
TTCTTTTCGCTCTTTCTTCGCAGATGTTTATCTTATGCTCGATTAAGATTACATTCCCTGTCTCTCTGTCTCTCT
CTCCATCCATACCATTCTCTGTCCATGTTCTGATGTGAGAGACAACGACTCATTCCTCTTCATCCGTGTGCTACG
GTTTCTCAGCCATATTGCTAATGTTCATGTAGCAAGGCAGTTCTTAGTCTCTTTGACATTTATATTGAGAAAACG
ATATCGGTGAGACCTGCCCTAAAACTTAATGTTAGTCGCAGGATCTTAATTTGTTCGTAATGCTCTTCTGACTAA
TTTCTCCTTCTTATCCACTGGTAACCTTGGGATCCATTTGCCTCTTAGTTTCTCTCTAAATCAAGAAAAATTACA
TATCGACATGCATGCATGCATGCATGCATACTAGAATTTTAAGCAAAGATTAAGGTTTGTTGTTTGTGGCAGTTC
ATGCCGTATGGCAGCTCCGCCGCTTTGCCCGCCGGCGCCATCTCCCACCCGCCCCTCCTCTCGCCCTCACTCCAT
AAGTCCATATTCAGCTCCCCAGGCCTCTCCCTCGCCCTGGTAACCCACCGTTTCTTGTCTCCTTCTTTATCCCGT
ATTGTTCGCTTTCTTGGAGCGGTTTTGGTGTCTTTCCGCAGCAAACGAACTTGGACGCGCACAGGATCGAAACT

TGGTCCCCGCCGTCGGAGGAGACGGAGAAGACCTGGATTCGGCTCGTCGGAGAAAGCAAGATGAGAACGATAGCA
GATCAGGGAGCGATAATCTGGAAGGCGGATCTGAAGACGATTTGGAGCTAGAGAATCCCCGGAAGAAGAAGAAAT
ACCACCGCCACACCCCTCAGCAGATCCAGGAGCTGGAAGCGTAAGAGATCCATTTGCATCGACCTCGTATCGTTT
GATTCTGCCCTCGTTTCCCAAAGGAAAACAACTCGTTTGAATCTTCTGTTTCTAGCCTTTTCAAGGAATGCCCCC
ACCCCGACGAGAAGCAAAGGATGGAGCTCAGCAAGAGGCTCTGTTTGGAGCCTCGCCAGGTCAAGTTCTGGTTCC
AGAACCGGCGAACACAGATGAAGGCATTTGCTCTTGCCTGAGTTCATCTCTGTTATAAAAATCTCGTTTTTTTTG
GGTTGTGTTTCCCCTCTTTCCTGACGTGCTTGCGAATCATGGAAACCAGACCCAGATGGAGCGGTACGAGAACAG
CATCCTCAAGCAAGAGAACGATAAGCTTCGAGCGGAGAACCTGTCCATCAGAGAGGCCATGAGGAACCCCATGTG
CTGCAACTGCGGTGGCCCCGTGGTGCTCGGCGAGATCTCTCTCGAGGAGCAGCACCTGAGAATCGAGTATGCTCG
CCTGAAGGATGAGCTCGACCGGGTCTGCGCCCTTGCCGGGAAGTTCCTCGGCAAGCCCGTGTCTGCCTTGGCTGG
CCCACTCGCTCTCCCGACCCCGAACTCTTCGCTGGAGCTCGCGGTCGGTACGAATGGCTTTGCCGGCCTCGGTTC
GGTGGCGGCCGCTACCTTGCCTCCGCTGGCTGATTTCACCTCTGGAACCTCAAGCCCTCTAGGCACCGTAATAAC
CCCCGGGCGGGCTGTCGGCGCTGGAGCCATCGGTGGGGTGGACACGTCTCAGGAGAAGTTCGTGTTCTTGGAGCT
TGCACTCGCTGCCATGGATGAACTGGTGAAGATGGCCGAGATGGAGGAGCCTCTGTGGATCCCGAGCTTGGACGC
GGGGAGGGAAACACTGAACTACGTCGAGTACGACCGGTGCTTCCCTCGGTGCGTCGGGGCCAAGCCCAATGGTTT
CGCGTCGGAGGCCACCAGGGAGACCGGCTTGGTCATCATCAACAGCTCAGCCCTCGTCGAGACTCTCATGGACGC
TGTAAGCTAGCTTTAATCTATGCATCGTTTTACCAGTGCATACGCACGTGAATCCAAGCTCTTATATGATCCATG
GGCAGGCTCGATGGGCAGATATGTTCCCTTCTGTGATCGCAAAAGCGACCGCTGCAGACGTGATCTCCAGCGGCA
TGGCCGCGACTAGAAACGGTGCACTCCAGCTTGTGAGTTCATATGTCCTGCAGCTGCTTAAGCAATCTTGCACCA
ACCACTCCTTCGTTCATGGGTATCGAGTCTGATGCCGACTTCTTTTGGTGTGTCACCAGATGCATGCGGAGCTCC
AAGTTCTCTCTCCCTTGGTTCCCGTTCGGGATGTCAGTTTCCTTAGGTTCTGCAAGCAGTTGTCCGAGGGTGCTT
GGGCCATAGTCGACGTCTCCATCGACGGAATTAGAGGCAACCCATCTGCTCCTCCGGCTAAAATGACATGCCGCA
GGCTTCCTTCTGGGTGCGTAGTGCAAGATATGCCTAATGGGTATTCCAAGGTAATAAAATCCCTCTACTTCCCCA
CTCCTCCCTCTTTCTTCCCTCTCTGGAACACATTACTCACGTTTCAGTAGCATCTTGCACTAACCATAAGCAGTT
CTATATGTGTTGGGCGTGATGTTTTAATAGAGATTTAGAAGTCAAGCCTAAGCTCGTGCTCCCAAATCTATTTGG
GTCGCGCGTCCACGCTACCATCAATATAATTCGGTCATCTCTTCTTCACCCTTGTTTCTGGTGCTTCTTTCTTTA
TATGTTTAGATGCTTCTTCTACCACCTTGAGAGCCTCGCCCGTGGATTGCTCTCTGTCCGCATTCAATTCGTAGG
TGTCTCGTTTGTGCTACCTATCTCATTATTTTGTGGGAAGTCTCTCCTCTGGCTGTTCCCAACTCCCGCGGATTG
GTGCTAAAAATGGCTACTCCCTCGCGAACCGCGACTCCTTTCCTTCGGACATGGGTTTCTTGTATTCCCGGTCTT
GACTTCCTCCCCTAAAGATTCATTAATACGCCCTCTTTTAATAGGGTCTAATGGTAGAAAATTCCGTCCATCTAT
TGGGGATATCATGCATTGGTTATGGTGGTCAAAGCTTCTGGCGTTTTTCTTTTAAGTCTCGAGTTTCCATGGTGG
TTGACAGATTAAGCTGTCTCTTAGTGATAACGTGCACTGGCGACCATGAGATGTGGTGATCTCCAGGTCCAGCAG
GTGCACTGTAGCGTCAACCTTGCAGACTGAGGAATTAATGAACTAACGACGAGGGTGGTGCGCAGGTCACATGGG
TCGAGCACGCCGAGTACGACGAGACTACAGTTCACCCGTTGTACCGCCCGCTGCTGCGCTCCGGTTTGGCCCTCG
GCGCACGCCGCTGGGTCGCGACACTCCAGCGCCAGTGCCAGTCCCTCGCCATCCTAATGTCCTCCTCCCTCTCCC
ACGATGACAACACCGGTGAGTGTCATGCGAATAATGAATTTGTTGTCTTCAGCGGTAGTTTCATAGAATTCAGTA
TTTGACTTGCCCCACGGGATTGCCGCGACAGCGGTGACGCCGAGCGGGCGGAGGAGCATGCTGAAGCTGGCGCAG
CGGATGACCGACAACTTCTGCGCCGGGGTCTGCGCGTCGTCGGCTCGCGAGTGGAACAAGCTGGGCGGAGGGGTC
AACATCGGGGAGGACGTGCGGGTGATGACGAGGCAGAGCGTGGCCGACCCAGGGGAGCCGCCGGGCGTGGTTCTC
AGCGCCGCTACCTCGGTGTGGCTCCCGCTCTCGCCACAGCGCCTGTTCGACTTCCTCCGCAACGAGCAACTCCGG
AGCCAGTGGGACATCCTCTCCAACGGCGGGCCCATGCAGGAGATGGCCCACATCGCCAAGGGCCAGAACACCGGC
AACGCCGTTTCCCTTCTCCGTGCCAGCGTCAGTATTTTCCCACTTCGATACGCACTGTGGTGGCCTGTTCCTTTT
TCTTCTCCGAAAAGATACAGTACTACCATTAGATTTGACATATGAAGGACAGACACAATGCCGTCGTTTCTTCTA
CAGTTCTGACATGATGCTTAGCTATCTTAGCTTCAGAAGCCTTCTCTCACACTCGATTCATCTTATACATATATA
ATTTGGCTGCGATCACTACTGATCCAGGTTATATGACTGCCATAACATGGGTTCCATGCAGGCCGTGAGTGCAAG
CCAGAGCAGCATGCTGATACTGCAGGAGACGTGCACGGACGCGTCGGGATCATTGGTGGTGTACGCGCCGGTGGA
CACCCCGGCCATGCACCTCGTCATGAGCGGCGGCGACTCCGCCTACGTCGCCCTCCTCCCCTCCGGCTTCGCCAT
CCTCCCGGACGGCAGCGGCGGCGGTGCGCGCAAGGCCGGGGGCTCGCTGCTGACAGTGGCGTTCCAGATCCTGGT
GAACAGCCAGCCAACGGCGAAACTGACGGTGGAGTCGGTGGAGACCGTCAACAACCTCATCTCGTGCACCGTCCA
AAAGATCAAGGCGGCGCTCAACTGCGAGCCGTAACAGAGACGGGATCCAGCGGTGGTGGGGAGAGTGCCGTGGCC
ATGGGTTGAGTCAAGAACGAACCGCGCGAGCGAAGAACCTTTGCTATGTGGTAAGGAAGTATGGCCAACTGCAAG
CCCTGCGCGCAGTCCATAATCCTTAACACCGAGCAATGGTGGTGGTTCGGGCATTGACTCTCTCTCTCTCTCTCT
CTACCTCTCTAAACGCTGTTGCTCGATCCACAGCCGCGTCCCGTTGGTGTTGTAGTGTTCCCTTCTCCCAGCGAG
TTTGGATTTCCTTGTGGAAGACGACGCTGTAATCTTTGCTATTTCATTGTATGTGAACGTATTCAGACATTGATC
ATTTTACTTGCTTGTTTGCAGTAACATATGGGAATGTATTAACCTATCATATTTATCATCATAGATCGAAGTTGG
ATTGCTACTTAAAGTCATAAGCTAATCACTATGATTTAACCATGTACGCTTCTCACGGGAACCCAACTCATGGCG
GGGGTAATA

CDS

ATGCCGTATGGCAGCTCCGCCGCTTTGCCCGCCGGCGCCATCTCCCACCCGCCCCTCCTCTCGCCCTCACTCCAT
AAGTCCATATTCAGCTCCCCAGGCCTCTCCCTCGCCCTGCAAACGAACTTGGACGCGCACAGGGATCGAAACTTG
GTCCCCGCCGTCGGAGGAGACGGAGAAGACCTGGATTCGGCTCGTCGGAGAAAGCAAGATGAGAACGATAGCAGA
TCAGGGAGCGATAATCTGGAAGGCGGATCTGAAGACGATTTGGAGCTAGAGAATCCCCGGAAGAAGAAGAAATAC
CACCGCCACACCCCTCAGCAGATCCAGGAGCTGGAAGCCCTTTTCAAGGAATGCCCCCACCCCGACGAGAAGCAA
AGGATGGAGCTCAGCAAGAGGCTCTGTTTGGAGCCTCGCCAGGTCAAGTTCTGGTTCCAGAACCGGCGAACACAG
ATGAAGGCATTTACCCAGATGGAGCGGTACGAGAACAGCATCCTCAAGCAAGAGAACGATAAGCTTCGAGCGGAG
AACCTGTCCATCAGAGAGGCCATGAGGAACCCCATGTGCTGCAACTGCGGTGGCCCCGTGGTGCTCGGCGAGATC
TCTCTCGAGGAGCAGCACCTGAGAATCGAGTATGCTCGCCTGAAGGATGAGCTCGACCGGGTCTGCGCCCTTGCC
GGGAAGTTCCTCGGCAAGCCCGTGTCTGCCTTGGCTGGCCCACTCGCTCTCCCGACCCCGAACTCTTCGCTGGAG
CTCGCGGTCGGTACGAATGGCTTTGCCGGCCTCGGTTCGGTGGCGGCCGCTACCTTGCCTCCGCTGGCTGATTTC
ACCTCTGGAACCTCAAGCCCTCTAGGCACCGTAATAACCCCCGGGCGGGCTGTCGGCGCTGGAGCCATCGGTGGG
GTGGACACGTCTCAGGAGAAGTTCGTGTTCTTGGAGCTTGCACTCGCTGCCATGGATGAACTGGTGAAGATGGCC
GAGATGGAGGAGCCTCTGTGGATCCCGAGCTTGGACGCGGGGAGGGAAACACTGAACTACGTCGAGTACGACCGG
TGCTTCCCTCGGTGCGTCGGGGCCAAGCCCAATGGTTTCGCGTCGGAGGCCACCAGGGAGACCGGCTTGGTCATC
ATCAACAGCTCAGCCCTCGTCGAGACTCTCATGGACGCTGCTCGATGGGCAGATATGTTCCCTTCTGTGATCGCA
AAAGCGACCGCTGCAGACGTGATCTCCAGCGGCATGGCCGCGACTAGAAACGGTGCACTCCAGCTTATGCATGCG
GAGCTCCAAGTTCTCTCTCCCTTGGTTCCCGTTCGGGATGTCAGTTTCCTTAGGTTCTGCAAGCAGTTGTCCGAG
GGTGCTTGGGCCATAGTCGACGTCTCCATCGACGGAATTAGAGGCAACCCATCTGCTCCTCCGGCTAAAATGACA
TGCCGCAGGCTTCCTTCTGGGTGCGTAGTGCAAGATATGCCTAATGGGTATTCCAAGGTCACATGGGTCGAGCAC
GCCGAGTACGACGAGACTACAGTTCACCCGTTGTACCGCCCGCTGCTGCGCTCCGGTTTGGCCCTCGGCGCACGC
CGCTGGGTCGCGACACTCCAGCGCCAGTGCCAGTCCCTCGCCATCCTAATGTCCTCCTCCCTCTCCCACGATGAC
AACACCGCGGTGACGCCGAGCGGGCGGAGGAGCATGCTGAAGCTGGCGCAGCGGATGACCGACAACTTCTGCGCC
GGGGTCTGCGCGTCGTCGGCTCGCGAGTGGAACAAGCTGGGCGGAGGGGTCAACATCGGGGAGGACGTGCGGGTG
ATGACGAGGCAGAGCGTGGCCGACCCAGGGGAGCCGCCGGGCGTGGTTCTCAGCGCCGCTACCTCGGTGTGGCTC
CCGCTCTCGCCACAGCGCCTGTTCGACTTCCTCCGCAACGAGCAACTCCGGAGCCAGTGGGACATCCTCTCCAAC
GGCGGGCCCATGCAGGAGATGGCCCACATCGCCAAGGGCCAGAACACCGGCAACGCCGTTTCCCTTCTCCGTGCC
AGCGCCGTGAGTGCAAGCCAGAGCAGCATGCTGATACTGCAGGAGACGTGCACGGACGCGTCGGGATCATTGGTG
GTGTACGCGCCGGTGGACACCCCGGCCATGCACCTCGTCATGAGCGGCGGCGACTCCGCCTACGTCGCCCTCCTC
CCCTCCGGCTTCGCCATCCTCCCGGACGGCAGCGGCGGCGGTGCGCGCAAGGCCGGGGGCTCGCTGCTGACAGTG
GCGTTCCAGATCCTGGTGAACAGCCAGCCAACGGCGAAACTGACGGTGGAGTCGGTGGAGACCGTCAACAACCTC
ATCTCGTGCACCGTCCAAAAGATCAAGGCGGCGCTCAACTGCGAGCCGTAA

GSMUA_Achr10P25900_001 (*MaHDZIV17*)

TGGTTCCTTTGTCTTGTGCATGGTAGTCCCATGCGCACTCCTCTCACTACGAGTACCCATCATGTGGTTCCTAAA
CCTGGACGGCTCTCAAAGTTCTTGGAGGCTTCTGTGACTAAACTACCATGCCCAACATGGGGAATCGATCTGGTT
AGTGTCAGAGGATGGTGTTCAAGAAGAAAAAGGAACAAGATATGTTCTCTCTTGTTTGTATTCTTTGGAGCCCAC
AAATCAGATGACACAGCTCAAGATCTTGCGTGGTTTGACTCCCATGGATTTCCGGGTTTGGGGGAGACAGTAGGC
AAAGGGGGGCAAGCTTTAGCTCTGTGCTGCTGACATAGCATTTCACCCTCCTCCCTTCCTCTACATCATCCTTGG
CATGCAGCAAGCGGAAACAGCTGCAGGTATTAAAGATCTCTAACTCCTGGTATTTAAGGAAGGGAGGCTAAGTTT
TCAAACAGGGTGTCTTTACGGCCTGATTTGGTCTCAGGATGCTGGTCATCATATGGTTGTGCAGCAGTAATTTCA
GCTGCAGAGGGAGAAAGAAATTGTACCACCACATAACCATCATAACTCTGCTCAGCTGACAAGTAATCTTGATTG
CTAGCATTCACTGCATGAGTAGAAGCAGAGCTTCCCACTTCCCACATGAGAGAGATCAGACTGTGTTCCTACGCT
CTCCTTATCAGCAATTATATGTGCGGTGTTGGTGTGTAATCAGCATTTTCCAAGTCGGAGAATTGTAACAGCAGA
CGTGAGAGAGAAGGGAAGGGGTGAGAGCTGGCAGATTAGGAAGCTCACCGTTCCTCATGCTTCTTTCCTCTTGTC
TGTGTCTCTGCTCTGCTGTCCTGCCTTTAATTCTTGAAGATGTACTGTTCCACTAGAATAACCAGACCAAAATCA
TAATCAAAATCGAACCTTTTGTTGTATGTTTCTGCCAAAACTTGTTTTTTTTTGCTTTAGAAAGTGCATTGGAAA
ATAGGAAGAAGAAACAATACCAGATGATGATAAATCGGAACCAGAAAACTGGCATTTGGAGAAGCATATAACAAA
AGAATCAGATGTATATTCTTGCAATCTGCTGATAATAAATATATGGAGATGATATAGTATGCCTTCAAGATCCAA
CATCTTTAAAGCCCCGTCCTTCTAAATCGCGTCAATTACTGTGAGCTTTAAATACACACACATACGCAGAGAGAG
TTTCTCGGTGGTAGAATCTCTCGGTGCGTCTTAATAAGGGGAGGGGGCGAATTCGCCCAGATTGCTGGCGATATC
TGCGATGCGCGCACCGCCGGTTTCCTCAATTGGCTCTCGCTTAAGCGCCGCAAGGAGAGGAGCCCCGTAGAGGGA
AAAAGGTGCCACCTTTGAGGACCAAAGGTAGGGGGATAGCGTCGCCAAGTTTCGGAAATAGATGGTGAGGACTGG
CTGGCTTCTGTAGCAGATTGTTGTGGGTGCTGGTCGTGATAGGAGCGGTGAGGAGGAGTTTCGGAGGTGGCGAGG
ATGGACTCCGGCGACGAACAGGACGTTCCGGACTCACAAGGGAGGAAGAAGCGGTACCACCGGCATACCCCGCGG
CAGATTCAAGAACTGGAGTCGTATGCTCTCGTCTCTTTCTTCTTCTCCTATCTCTCGCGTTGTCTTTACTGTGTC
TGACGACGTCATGGTGTTGTTCTCTTGCGTCGTGGGGTTGTTCCGTGGCGGCGCGACGATGGGCCTTGTCTGAGT
TTGTTGTTTAGGATGTTCAAGGTCTGCCCGCACCCGGACGAGAAGCAGAGGAAGCAGCTGAGCCGCGATCTGGGG
CTGGAGCCGCGGCAGATCAAGTTCTGGTTCCAGAACCGCCGGACGCAAATGAAGGCGACCGCTCTGCTCCCCGTC
GTCCTCTTCCCTCCCTCGTCACGCCTCGCGTGCGAGCAAAAGGAGCGACCTTTGCCTCGTTTGATCGACCCTCCC

GCTGTCTTTGCAGGCGCAACAAGAGCGGGCGGACAACTGCTTGCTCCGCGCTGAGAACGACAAGATCCGGTGCGA
GAACATCGCGATGAGGGAGGCCCTCAGGAACGTCATCTGCCCCTCCTGCGGCGGTCCTCCCCCCGATGACGACCC
CTACTTCGACGAGCAGAATCTGCGGATGGAGAATGCCAAGTTGAAGGAGGAGGTAACCTCCACCTCCCTTCCTTT
TCGATCCTACACGTGCATTCGTCGTATAGTTGCGATAGCTTCAACGAAACAATGGAAAATCCATCTTTTTTCATT
GATAGTTTGGTCAGTCATCCCATCTTTCAATGAATTGGATCAAAAAGAGCTGTCCTCGTTCTATTTCTTTGGCTT
TTGAAGGATGTCAGCCGATGAGTTCATTAATCCATTTTGATGGTGGAGGATCAAACAAACATACTTCAACACACT
CATTTAATGTGATACTGTTGTCTTTGATCCATGCCAATGAATCCTTGCAGCTCGATCGCGTTTCGAGTCTTGCAT
CGAAGTACCTGGGAAGGCCCATCACGC**AG**CTTCCTCCAGTTCAGCCAATGTCGATCTCATCGCTAGACTTATCGG
TTGGAGGATACAGTCATCCGGGGATAAGCCCTTCCCTCGATCTCGATCTCCTGTGCCAGAGCTCGTCTTCGGCAT
TTCCGTATCCGTTTCCAGCATCAATGTCGGAGCTTGACAAGCCTCTCATGGTCGAGATGGCCACCGGTGCAATGG
AGGAAGTCATCAGAGTGGTGCAGACCGATGAACCCCTGTGGGTGAAGTCGGGCAGCGATGGAAGGGACATACTCC
AACTCGAAACCTACGACAGGATGTTCCAAAGGTCAGCTCGGCAGCTCAGGTTCTCAGATACTCGAGTCGAGGCAT
CGAGGGATTCAGCTCTTGTGGTCATGAATGCTGTGACATTGGTCGACATGTTCATGGACGCAGTAAGTTCCAATC
TCGTTGAAAGGAAACGAGTTCTCCTTTGATCTTCTACCTCTGAATTGGATTTCGTTGCAGAGCAAGTGGGGAGAG
CTGTTCCCGACGATCGTGTCCAAGGCAAGGACTGTCGAAGTCCTGGCGGCTGGAATGGCTGGAAGTAGAAGTGGA
TCTTTGGTGTTGGTAACGACAGTTCTTCAGCTCTTCTGCTTGAATCCAATAAACTTCCTCTGCGATACTGATACA
TCCAAAGGGCGTGCAGATGTACGAGGAGCTACAGGTTCTTTCGCCGGTCGTTCCTACTCGCGATTTCTGCTTCCT
GCGGTATTGCCAGCAAATAGAGCCCAGCGTGTGGGCGGTGGCTGATGTTTCGGTGGACTTTCCCGGAGATAACCA
GCTCGCACCTTCTTCCCGATCAAGGAGGCTTCCTTCTGGCTGCTTGATCGAGGAAATGCCCAATGGCTATTCTAA
GGTGATCCAAATCATGGTGCATGATCTACTGATTTCTTTCTCGCCCTTACCGAATTGTCGATGTAGGTAACCTGG
GTCGAACACATGGAGATCGAAGAAAAGAATCCGATTCACGCGCTCTTCAGGGATCTTATAGACGGTGGAATGGCA
TTCGGAGCACAGCGCTGGCTCGCGACTCTCCAGAGAATGTGTGAGAGGTTTGCTTGTTTGAACGTCGCCGGAATT
CCAGCTAGAGACATCGGAGGTAAGGAGCGTTCAGAATCCGCACAGCAGATAGCTAAGATTGCTATTTGATTCTGA
TTCCGACTGTGGAAATCAGTGGCTCCCTCTCCCGATGGCAAGAGGAGCATGATGAAGCTTGCTCAGAGGATGGTG
ACCAACTTCTGTGCCAACGTCGGCGCATCGAATGGTCACAAATGGACTACACTCTCGGGGGTAAACGATGTCGGA
GTCAGAGTTACGCTTCACAAGAACACAGATGCCGGCCAGCCTAATGGAGTGGTTCTCAGCGCAGCAACCTCTATA
TGGCTTCCTATATCAGCTGAGAGGATCTTTAGTTTCTTCAAGGATGAACGAACTCGAACTCAGGTTTCTGCAGAC
ATCCTTCTTTGCTGTTCTATCTTTCTCGATCGAAGGACACTTTTTAACTCTCTGCGTCTTCTTCTTGTTCTCAGT
GGGATGTTCTCTCGAACGGAAACACTGTACAAGAGGTGGCTCATATCACGAATGGTTCACATCCGGGAAACTGCA
TTTCTCTTCTTCGTGTATGCATTCTTTCTCGTCTTTATTCAAGTGTAGATGAATTTAATTTGGGTGCCAACCTCT
GTCAGAAGTTCTTGATGGTCTATTTACTGTGGTGGATGAACAGGGACTCAATTCTGGGCAGAACACGATGCTGAT
ACTCCAAGAGTGCTGCACTGATGCTTCGGGCTCGGTTGTGGTCTATTCGCCGGTCGACCTGCCGGCCATCAACAT
CGTCATGAGCGGAGAGGATCCGTCCTACGTACCGATTCTGCCGTCAGGCTTCACCATATTACCGGATGGGCGATC
GGCTGGGGGCTACGGCGCGTCGTCCAGCTCGAATCCGATGGGTGGCTCCGCCGGCTCGCTGGTGACCGTGGCGTT
TCAGATACTAATGAGTAGCTTGCCGTCGGCGAAGCTCAACTTGGAGTCGGTAATGACGGTCAATAACTTGATCGG
CACCACAGTTCAGCAAATAAAGGCTGCCTTGAGCTGTCCCGACGTCTGACGACGCTCCGAAGAGAATTAAAAGTC
TGTGCTTCTCTTCCGACCACCACCACCTTCCTTGAACTGCCTGGGGTCTGATGGACTCTCGAGGCTTTTGATGTG
GCATTCAAGATGGGGATTGAAGTGGGTCTTCTTTTGTTGGTTGGCTCGCCATGAATGCTCCCCTGGTGGTGGTGG
TGGTGGTGGTGCTGCTGCTGCTGGTGGTGGTGGTGGAGGTGGTGGTGGTGCGAGTCTCAGGACAGCCCAATTTTT
TTTGCTTCACAGGGATCAGTCAGGCTCTACATTTATAAGTACTTAATGAAGGAAGTCAAGAACGCACCATGTCTG
CCTCTCCTCTGCTGCTGTCATTAACTTCTTCTTGTAGCTTTGCTTTGCTTTGCTTCTCTTCTCCTCACTTGGATC
CAATTCTTTCCTCTCTTCATGTAGCAAAGTGTGGTCATGGTTCGGGCATTGACTTCTGTCCTTGAACATCCATCA
ATGAGCATTTACTTTGAAGTAGTC

CDS

ATGGACTCCGGCGACGAACAGGACGTTCCGGACTCACAAGGGAGGAAGAAGCGGTACCACCGGCATACCCCGCGG
CAGATTCAAGAACTGGAGTCGATGTTCAAGGTCTGCCCGCACCCGGACGAGAAGCAGAGGAAGCAGCTGAGCCGC
GATCTGGGGCTGGAGCCGCGGCAGATCAAGTTCTGGTTCCAGAACCGCCGGACGCAAATGAAGGCGCAACAAGAG
CGGGCGGACAACTGCTTGCTCCGCGCTGAGAACGACAAGATCCGGTGCGAGAACATCGCGATGAGGGAGGCCCTC
AGGAACGTCATCTGCCCCTCCTGCGGCGGTCCTCCCCCCGATGACGACCCCTACTTCGACGAGCAGAATCTGCGG
ATGGAGAATGCCAAGTTGAAGGAGGAGCTCGATCGCGTTTCGAGTCTTGCATCGAAGTACCTGGGAAGGCCCATC
ACGCAGCTTCCTCCAGTTCAGCCAATGTCGATCTCATCGCTAGACTTATCGGTTGGAGGATACAGTCATCCGGGG
ATAAGCCCTTCCCTCGATCTCGATCTCCTGTGCCAGAGCTCGTCTTCGGCATTTCCGTATCCGTTTCCAGCATCA
ATGTCGGAGCTTGACAAGCCTCTCATGGTCGAGATGGCCACCGGTGCAATGGAGGAAGTCATCAGAGTGGTGCAG
ACCGATGAACCCCTGTGGGTGAAGTCGGGCAGCGATGGAAGGGACATACTCCAACTCGAAACCTACGACAGGATG
TTCCAAAGGTCAGCTCGGCAGCTCAGGTTCTCAGATACTCGAGTCGAGGCATCGAGGGATTCAGCTCTTGTGGTC
ATGAATGCTGTGACATTGGTCGACATGTTCATGGACGCAAGCAAGTGGGGAGAGCTGTTCCCGACGATCGTGTCC
AAGGCAAGGACTGTCGAAGTCCTGGCGGCTGGAATGGCTGGAAGTAGAAGTGGATCTTTGGTGTTGATGTACGAG
GAGCTACAGGTTCTTTCGCCGGTCGTTCCTACTCGCGATTTCTGCTTCCTGCGGTATTGCCAGCAAATAGAGCCC
AGCGTGTGGGCGGTGGCTGATGTTTCGGTGGACTTTCCCGGAGATAACCAGCTCGCACCTTCTTCCCGATCAAGG

AGGCTTCCTTCTGGCTGCTTGATCGAGGAAATGCCCAATGGCTATTCTAAGGTAACCTGGGTCGAACACATGGAG
ATCGAAGAAAAGAATCCGATTCACGCGCTCTTCAGGGATCTTATAGACGGTGGAATGGCATTCGGAGCACAGCGC
TGGCTCGCGACTCTCCAGAGAATGTGTGAGAGGTTTGCTTGTTTGAACGTCGCCGGAATTCCAGCTAGAGACATC
GGAGGTAAGGAGCTGGCTCCCTCTCCCGATGGCAAGAGGAGCATGATGAAGCTTGCTCAGAGGATGGTGACCAAC
TTCTGTGCCAACGTCGGCGCATCGAATGGTCACAAATGGACTACACTCTCGGGGGTAAACGATGTCGGAGTCAGA
GTTACGCTTCACAAGAACACAGATGCCGGCCAGCCTAATGGAGTGGTTCTCAGCGCAGCAACCTCTATATGGCTT
CCTATATCAGCTGAGAGGATCTTTAGTTTCTTCAAGGATGAACGAACTCGAACTCAGTGGGATGTTCTCTCGAAC
GGAAACACTGTACAAGAGGTGGCTCATATCACGAATGGTTCACATCCGGGAAACTGCATTTCTCTTCTTCGTGGA
CTCAATTCTGGGCAGAACACGATGCTGATACTCCAAGAGTGCTGCACTGATGCTTCGGGCTCGGTTGTGGTCTAT
TCGCCGGTCGACCTGCCGGCCATCAACATCGTCATGAGCGGAGAGGATCCGTCCTACGTACCGATTCTGCCGTCA
GGCTTCACCATATTACCGGATGGGCGATCGGCTGGGGGCTACGGCGCGTCGTCCAGCTCGAATCCGATGGGTGGC
TCCGCCGGCTCGCTGGTGACCGTGGCGTTTCAGATACTAATGAGTAGCTTGCCGTCGGCGAAGCTCAACTTGGAG
TCGGTAATGACGGTCAATAACTTGATCGGCACCACAGTTCAGCAAATAAAGGCTGCCTTGAGCTGTCCCGACGTC
TGA

GSMUA_Achr7P00210_001 (*MaHDZIV3*)

TTACTATTGATTGCATCATGCAAATTATATGTCTTTTGATAACTTATTTAATAAATAAATAATAATGTTTTCAGC
ATATCTTTGTATTCATTGGACTTGCAACAAGGCTTTGATGATATATTCTCCTAAGGTACCGAGTTTAATTTTATA
TTACATATATATATTTTTTATGGGATTTTGTGATCTTGGCCGGTCCATTTCATTTATGGCTTGACCAGTGTCGAC
AAGCTTCTCTTCTTCCACTCGAACAGCAGCAGCAGCAGCAGCAGCAACAGCAGCAGCGAGTAGGCTATCAATAAA
TGCAATTGCACCCGCTATACCTTCCGCAATAACTCCTCCGCCCACCGCTCCCTTTTTCCTCCCCTTCCTCCTTCC
TCCTTCCTCCTCGCGGCAGTCTCCCTCCTCTTAAGATTCCAGTTTCCGTTTCCAAACAATACTGCACGAGTCAAG
AAATGAGAGATAGAGAGATTTAGGAGCGCTCTCCCCTCTCCTCAGAAACACTTGGAAGCCGCCACCGATGCATTC
TTAAACGGATGGCAGTTTTGTCTCGCGACTTCTCCCTGCAGCATCCAAGCTAGGTTTCGATCGGAACAAATATGC
TGAGAAAGAATAATGATCCCGGCAACCAGATGGAATCATCACCGGTTTCTAGGAGAGGGTATGAGGGTGCCTTCC
CAGCTTTGGCACTTGGTCAGGTCTGTAAGCTACACTAGATACTCAAAGATAAGCCGTTATATTTTTCTCTATCTT
TCTTTGGACTTTAGTGCAACGATGCCATGAGGGTACATGTTCTGAGAGAAGGTGGCATCGAGTGAGAACAAAGTC
GTGGGTGCATGCACCCGCTTCGCAAAGCTACCTGCCCCTCTGATTCGCCCTCTCCATCGCATTGCAGCTTGTTAT
CTTTGAGGGTTGATCTTTGCAGCCTATATAGCAGCCAGCAATGGCGGCCGTAGCGAGTGGCTACTGGTTGGTTAT
GAGGGGTTTGAGAGAGTTTCGCTAGCTAGAGGCTGTGGAGTTTAGGGAAATGGAACAAGGTTGAGCTCTTACATT
TTCTTTTCGCTCTTTCTTCGCAGATGTTTATCTTATGCTCGATTAAGATTACATTCCCTGTCTCTCTGTCTCTCT
CTCCATCCATACCATTCTCTGTCCATGTTCTGATGTGAGAGACAACGACTCATTCCTCTTCATCCGTGTGCTACG
GTTTCTCAGCCATATTGCTAATGTTCATGTAGCAAGGCAGTTCTTAGTCTCTTTGACATTTATATTGAGAAAACG
ATATCGGTGAGACCTGCCCTAAAACTTAATGTTAGTCGCAGGATCTTAATTTGTTCGTAATGCTCTTCTGACTAA
TTTCTCCTTCTTATCCACTGGTAACCTTGGGATCCATTTGCCTCTTAGTTTCTCTCTAAATCAAGAAAAATTACA
TATCGACATGCATGCATGCATGCATGCATACTAGAATTTTAAGCAAAGATTAAGGTTTGTTGTTTGTGGCAGT
TCATGTTAGAGGATCAAGAGCAAGATGATCTCCTGGATGATCAGATGATGGATCAGCTGATTCCCGAC
GTCGATGAAGACTTCGCTAACACGCCAGGAAGTGAGCCCACGGAGGGAGCTTCCGAGGACGAGCTGCA
AGATCATGGAAGCCGCGGTAAGAGGAAGCGGCACCACAGGCATACTCCGGATCAAATTCAACGGCTGG
AGGCGTAAGAACGATCTGCTTCCTCCCCCGACCTTTTTTGTTTTATTTCTGTTCCTTCGGAGAGCTTT
AACATGGTTCGCCACACGGACGCTCTGCTATTCATTAGGTTCTTCAAGGAGTGCCCGCACCCTGATGA
CAGGCAGAGGAATGAGCTCGGCCGAGAGCTGGGATTGGAGCCTCTCCAAGTGAAGTTTTGGTTCCAGA
ACAAGAGAACTCAAATAAAGGTCTGCCTCGCCTTGCTCTTACCGCGCGTGCCCACCTACTAAAGACTT
TTGATGTGAAAGCCATACATAAGATATCATCGTACATCGCTTCCTCTGTTCATTCATACACACTCTGT
CACATCATGCGTGCAGAACCAGAGTGAGCGGGAGGAAAACCAAAGGCTCAAGGATGAGAACGACCGGC
TGCTATCCGAGAACTTAAGGCTCAAAGAGGCTCTGACCGGTGCCTGCTGTCCCAACTGCGGTGGCCCT
CCTCATCTCGGTGAGATGTCGATCGACGAGCAAGAGCTGCAGGCCGAGAATGCTCGTTTACAGGACGA
GGTACGCACTTGGCTTACTCGTCAAGGATGAAAGGCTGCTGCTATAACTCCGTGCCCTTCTTCTTGGT
GCAACAGATCGGAAGGATCTCGGCGATAGTCGCAAAGTACGCTGGTCAGCAGCTGATGACCTTCCAAA
CGCCTTCGTCTTCCATGGTTGGAGGCTTTAGGGCCCAACCAGAACCAGACATGACACTAAATGACAGC
TCCAGAGGCGGAGAGGTTGTTTTCAGGCCTCACGATCGGCGAACCGAGATGGGAAGACGGACGGCATT
AGCATTAGCACAAGTCGCTGCGGAGGAGCTTCTCGCAATATCTCAGCTGGGCGAGCCTATATGGACTT
GGGGCTACGACGGTTTCCGTGAAACCTTGAACCAGGAAGAGTACGCTCGGGCATTTCCCGGAGGGCTC
GGGCCAAAGATGGAAGGCTTGAGAACCGAAGCAAGCCGCGAGACCGCAGTCGTTCGCATGCACACCGG
GAGATTAGTTGACATCCTAATGGATGTGGTACGCAACACATGATGATGATGATGATGATGATGATGTT
GTTGTTGTTCTTCTTCTGAAACTCTTACGAGTTGCATGGAATTCTACAGCAGTTGCATGAATTGACCG
GATTGTGGATTGTGTTCCTCTTGATGATCTGCAGAGTAAATGGAGTGTGTTCTTCTCCAGCATCGTCA
CAAGAGCGACCACCGTGGAAATGTTTGCGACTGGAGTAGGAGAGGGCGGCTACGACGGCACTCTGCAA

TTGGTATAGCTCCGTTACATGATCTCGCTGTAGAGTGCATCTATATGTCTCTGCGATCGGTATAGCCC
TGTTACAACAGAGACCACTCTGCTAACCTAGTTAATTGGCACTGGGAGCTCCGAGCTTAGTTCAGGTG
TTGTCAGTTCATACATTGATAAATGGTGAATTTGATTTGATTAATAGGATCTTTTATCGAGAAATTTG
ACATAGCAGAACCCTATTGTTCGCTTCTTAGCATTACTATTATCATCACCATTTTGTTCATTGGCTAT
GTGCATTCTCAGATGGCTGCTGAACTACAAGTTCCAACCCCTCTCGTTCCTCTCAGAGAGAGCTTGTT
TCTAAGATACTGCAAACGCCATGACGGACTCTGGGTGGTGGTTGATGTTTCTATGGAAGTACGTCTCA
ACCCGATGGCTCGGTACCGAAGAAGACCGTCGGGCTGCGTAATACAACAACTGCCAAATGATTACTCC
AAGGTAAATGAGCCGACAAAACATCGATCTCCACCTTGCAAGTTCCGTTAAACTCTTTCTCTTCCAGA
TCTGATCTCATGTGTCATGCTCGCTACTACGATGTAGGTGGTATGGATCGAACATGTCGAAGTGGATG
ACAGTGGTGTTCATGACATGTTCAAGTCGATGGTGAACTCCTGTCTAGCATTCGGAGCTAAAAGATGG
GTATCAACAATGACGAGGCAATGTGAGAGGTTAGCTTGCCTCTTAGCCACGAACAGCTCCAGTGCTGA
TATAGCCGGTGATGACCCTTTCCTTTCCCTGTCAATTCATACGTCGCCTTGTTCAGCTTTCTATTTTC
ACATCTCTCTTTTTTGTAGCAAATCCTGATGGAACGAGGAACATACTCAAGTTAGCTGAGAGGATGGT
GGTGAGCTTTTGCAGTGGTGTAAGTGGGTCTGTGGCTCACCACTGGACAAATATATCCGGGAACGTCG
CAGATCATGTTAGGATCAGAAGCAGAAACAACGATGGAGATCCAGGGAGGCCTCCCGGAGTTGTTCTG
AATGCAAGCACGTCCATCTGGTTGCCAATTACCCCGAAGAGAGTCTTTGAGCTCCTGCGGAGTGAGTC
CTTACGCAACGAGGTATTCAGGGTTCCAGAAATAGTGCGTTCTTTCATTCTCACCCCCCAAAAGAATT
AATTACTTTTGTTCTTCCTTTGAACAACACATCAATCACTTGGATTATGTTTTCATGTGGTTATAGCC
AGCCATTCCAAACTCTTTTTTATATTTACATGTTAGAATCTGAGCCAGTTCACGATGACATGACTTGC
TATTCCAAGGGACGACAGCATTAGCAGTGACGTATATGTTAGACGTTAGCAAATTACCATGAGATTTC
TTTCTTTCTGATGCAAAGCCAACTTAGTTTCTTAAACTATTTCTGACAACACAAACTTGTTTGGAAGA
AGATGTACCCCTTCATCCGGTCGTTCTGATTTCGATTCTGTAGTGTACCCATACATAATTACATTCAA
GATCAATTGATCTCTCTCTCTCTCTCTCTCTCTCTCTCATGCATGACATGCTGGCATCCAGTAGGTGG
TTGGATAGAAGATGTGTACATCAATCACATAGATAGATATTTGTAATTTTTCTTGCGTTTTATTGTTG
TATGAAACAGTGGGATATTCTATTGAAAGGAGGCATGATTCATGAAGCAGCTTGTATAGCCAATGGCC
AACAAGGAGGCAATTGTGTCTCCATATACACCGTCGGAGTAAGTCTTTTGTTGTTCCTTTTTTCGCCT
TTTTACTGTTTCTACATTTTCTACTCGAATGCCTTGAGCTGATAAAAATGCATGCTGATCAATGAATG
AATGGTAGCTCCCTGAAGAAGAGACCACCAACATGATGATACTTCAAGAGAGCTGCTCCGACCCAACT
TGCTCGTACGTGATTTATGCTCCTGTCGATGCTGCTGCCATCGCTGCGGTCCTCAGTGGTGGAGACCC
CAACCACGTAGCGCTGCTGCCTTCGGGCTTCGCCATACTCCCCGATAGGCCTTCTTCCGCGGTCGACC
ATGATGGGGAGACTGACTTGGGGGGGGTTCCCTTCTGACCGTGGCTTTTCAGATCCTAGTATTCTCG
GGTCCCGGTTCCAAGATATCCCGTGGGTTTATAGCGACAGTCGACGGTCTTCTATCCTGCACCTGTGA
CAGAATCAGAACCATTCTATCGGGCAACAGTGTTCGCTGACGCAGGAGGAAAAAGAGGATCCTTCATC
GTAAGTCGATCTTCCTTCCGAAGTGGAAGTTTGCCTGCCTCCGAAACTAGGAAGATCAAATCATCATC
ATCATCATCATCATCAGAGATTTCTGATTGGCTTTAATAATCAAGAACTGAATGGAATTGATGTTTCA
GGTCTATGATTCTGGAATCGTTTTTTGAATGCTTGCTTGCTCTCTAACTCGATTCATTTTCCCTTCAC
CTTGCAGGAGGAGAACCACTGGGACTTCAAAGATCAAAGATGTATTTTCCACTCTTCAAGCCATGGCG
GTCGTCCTTTGGTTTTCAGAACAGTAGTCCATGATAATGATAATTAGATGGTTACCGTATGTATGTTA
ACAACCGGAACAGTCCTTTTTCATTTTCAGCATGAGTTGAGGATATTTTTCTAATCCGAGCAATCTTC
TTCCACTGTTATTCTTCTTGTTGTTGACCTTAACATGTTGTTCATGATGGTGGGGATGAAGCAC

CDS

ATGTTAGAGGATCAAGAGCAAGATGATCTCCTGGATGATCAGATGATGGATCAGCTGATTCCCGACGTCGATGAA
GACTTCGCTAACACGCCAGGAAGTGAGCCCACGGAGGGAGCTTCCGAGGACGAGCTGCAAGATCATGGAAGCCGC
GGTAAGAGGAAGCGGCACCACAGGCATACTCCGGATCAAATTCAACGGCTGGAGGCGTTCTTCAAGGAGTGCCCG
CACCCTGATGACAGGCAGAGGAATGAGCTCGGCCGAGAGCTGGGATTGGAGCCTCTCCAAGTGAAGTTTTGGTTC
CAGAACAAGAGAACTCAAATAAAGAACCAGAGTGAGCGGGAGGAAAACCAAAGGCTCAAGGATGAGAACGACCGG
CTGCTATCCGAGAACTTAAGGCTCAAAGAGGCTCTGACCGGTGCCTGCTGTCCCAACTGCGGTGGCCCTCCTCAT
CTCGGTGAGATGTCGATCGACGAGCAAGAGCTGCAGGCCGAGAATGCTCGTTTACAGGACGAGATCGGAAGGATC
TCGGCGATAGTCGCAAAGTACGCTGGTCAGCAGCTGATGACCTTCCAAACGCCTTCGTCTTCCATGGTTGGAGGC
TTTAGGGCCCAACCAGAACCAGACATGACACTAAATGACAGCTCCAGAGGCGGAGAGGTTGTTTTCAGGCCTCAC
GATCGGCGAACCGAGATGGGAAGACGGACGGCATTAGCATTAGCACAAGTCGCTGCGGAGGAGCTTCTCGCAATA
TCTCAGCTGGGCGAGCCTATATGGACTTGGGGCTACGACGGTTTCCGTGAAACCTTGAACCAGGAAGAGTACGCT
CGGGCATTTCCCGGAGGGCTCGGGCCAAAGATGGAAGGCTTGAGAACCGAAGCAAGCCGCGAGACCGCAGTCGTT
CGCATGCACACCGGGAGATTAGTTGACATCCTAATGGATGTGAGTAAATGGAGTGTGTTCTTCTCCAGCATCGTC

ACAAGAGCGACCACCGTGGAAATGTTTGCGACTGGAGTAGGAGAGGGCGGCTACGACGGCACTCTGCAATTGATG
GCTGCTGAACTACAAGTTCCAACCCCTCTCGTTCCTCTCAGAGAGAGCTTGTTTCTAAGATACTGCAAACGCCAT
GACGGACTCTGGGTGGTGGTTGATGTTTCTATGGAAGTACGTCTCAACCCGATGGCTCGGTACCGAAGAAGACCG
TCGGGCTGCGTAATACAACAACTGCCAAATGATTACTCCAAGGTGGTATGGATCGAACATGTCGAAGTGGATGAC
AGTGGTGTTCATGACATGTTCAAGTCGATGGTGAACTCCTGTCTAGCATTCGGAGCTAAAAGATGGGTATCAACA
ATGACGAGGCAATGTGAGAGGTTAGCTTGCCTCTTAGCCACGAACAGCTCCAGTGCTGATATAGCCGCAAATCCT
GATGGAACGAGGAACATACTCAAGTTAGCTGAGAGGATGGTGGTGAGCTTTTGCAGTGGTGTAAGTGGGTCTGTG
GCTCACCACTGGACAAATATATCCGGGAACGTCGCAGATCATGTTAGGATCAGAAGCAGAAACAACGATGGAGAT
CCAGGGAGGCCTCCCGGAGTTGTTCTGAATGCAAGCACGTCCATCTGGTTGCCAATTACCCCGAAGAGAGTCTTT
GAGCTCCTGCGGAGTGAGTCCTTACGCAACGAGTGGGATATTCTATTGAAAGGAGGCATGATTCATGAAGCAGCT
TGTATAGCCAATGGCCAACAAGGAGGCAATTGTGTCTCCATATACACCGTCGGACTCCCTGAAGAAGAGACCACC
AACATGATGATACTTCAAGAGAGCTGCTCCGACCCAACTTGCTCGTACGTGATTTATGCTCCTGTCGATGCTGCT
GCCATCGCTGCGGTCCTCAGTGGTGGAGACCCCAACCACGTAGCGCTGCTGCCTTCGGGCTTCGCCATACTCCCC
GATAGGCCTTCTTCCGCGGTCGACCATGATGGGGAGACTGACTTGGGGGGGGGTTCCCTTCTGACCGTGGCTTTT
CAGATCCTAGTATTCTCGGGTCCCGGTTCCAAGATATCCCGTGGGTTTATAGCGACAGTCGACGGTCTTCTATCC
TGCACCTGTGACAGAATCAGAACCATTCTATCGGGCAACAGTGTTCGCTGA

GSMUA_Achr7P11310_001 (MaHDZIV12)

ATGGTTTGACCCCTTTGGATTTCCGGGTTTAGGGAGACAGTAGGAATAAGGGGGCAGGCAAAATCTTAGTTCTGT
GTTGCTGACATAAACGTTTCACTTCGGTACTGGGTCACTCCTGGTAACCATCTCCTTCTCCTTCCCTTCCCCTTC
CCCTTCCTCTTCATCCTTCTTCCACTGTTTGATCATGGCAACCGAGTGATGGACTGAGGGAAGACAACTACGGGT
ATTAAAGATCTCTGATCTCATAGCATTGAAGGGAGGGAAGCAGCGTCTCTTCAACCATCTGGTTTCGGTTCAGCA
TGGTGGACATCATAAATCTGTGCACCAGTAATTTCAGCTGCAAAGGGTAAAATGTACCACCATGCAACCATTATG
GCTCTGCTCAGCTGACATGTAATCTTGATTGCTAGCATTCACTACATGAGGAGGAGGAGGAGGAGGAGGAGGAGG
GGGAGGAGCACACCACAGTCGCTGATTGAATCACCATCGCTTCTCACTCACCCGACATGAGACTGTCTTCAAGTA
GATGACGATCATGGAGTTAGAGCAGAGGGCGCATGCTATCATGGTAAGCAGCAGTGTGTTGCAATCAGCGGGACA
CGTGAGAGAGAAAGGGTCGAAGGTGAGAGCTGGCAGAGTAATAAGCTCAGCACTCTTCATGCCTCATCTCTCTCT
CTCTCTCTGCTCCGCTGTCTCATCTTAAATTCTCGAAGACGAATCATCCCAACAGACAATGGTTCAAAATCAAAA
TGGAAGCTTTTTCGTGCAATTTCTGCCAAAATTTCATCTTTGTTTCAAGTACACCTTGTGGTTGCTAGAAATTTT
AGATCGTACGAAAATGCATCGCAAATAGGAAGAAGAATTAAAGTCGGAGAGCGAATCAACGAGAAGAAGAAAGCT
GGGAGTAGTATCAGATTCAGAAAACTAGCATGAGAAAAGCGAATAATCCTAAATCTCAGATGTATATTATTGTGT
AATCTGCTCCTAATAAATGTATAAAGATGTGGTTCGGGGAGGATAGAATAGCCCATGAAGCGAAATCCAACATTG
TTAAAGCCCTGACCTTTTACATCGCGTCAGTTACTGTTGGCTTTAAATACAGAGAGAGAAAGAGAGAGAGAGAGA
GAGAGCAGAGTGAGAGGGGGAGAAGAATTCTCGGTGCATCTTAATAAGGGGGAGAGATGGGGGCGAATCCGCCCG
GATTGCTGCCGATATCTGCGATATGCGCACCGCCGGTTTCTCATTTAGCTCGCAAATCCCGCTCCCCGACGAGAG
TCACAGAGGGAAACTGATGTGTGTTTTTGCAGCCGGATGTGAAAGGCGCCACCTTTGAGGACGAAACATAGGGGG
AAATAGCGTCGCCAAGCCTCGGAAATAGATAGCATGAACTGGCTAACCTCTGTAGCAGATTGTCGTGGGTACCGG
TAGTGACAGTAGTAACAGTATAAGCGGGAGGAGGAGCGGTGAGGGGGGGTTTTGGAGGCGTCACAGGTTGGGAAA
ATGGACTCCGGTGACGAACAGGACGTGCCCGACTCACAGGGGAGGAAGAAGCGTTACCACCGGCACACGCCGCGG
CAGATTCAGGAGCTCGAATCGTATGCTCTGCTCTCTTCTATCTCCTTCGCTCTCTCGCTCTGTTTGTTTGGTGTG
TCCGCAGAGGTTATGGTGCTGTTTTGTTGTGTGTTTTGGGGCTGTTCGGTGGCGGCTTCCTGATGGGTTTCTCTG
AGCTTGTTGGTAGGATGTTCAAGGTTTGCCCGCACCCTGACGAGAAGCAGAGGGCGCAGCTGAGCCGGGATCTGG
GGCTGGAGCCGCGGCAGATCAAGTTCTGGTTCCAGAACCGCAGGACGCAGATGAAGGCAACCGCTCGCCGCTCCC
TACTGTTCTTGTTCCTCTTTCGTTCGTGTTCTCGATGGAATGACTGGACTTTGATGCTACTGCTAGCAAAAAGGA
GCTACCTTGGCCTTGTTTGATCGATCCTGGCGCTTTGCAGGCTCAGCACGAGAGGGCGGACAACTGCTTGCTCCG
TGCTGAGAACGACAAGATCCGGTGCGAGAACATCGCGATGAGGGAGGCTATGAAGAACGTCATCTGCCCTTCCTG
CGGCGCCCCTCCGGCCAGTGACGACTCCTACTTTGACGAGCAGAAGCTGCGGATGGAGAATGCGAGGTTGAAGGA
GGAGGTAACGTCGACATCACTTTCCTCTAATTTGTTTGTTGGTGGTGATCCTTCCCGTGTGTAAGTATCCTGTTT
AGTAGTCTCTTCACGCATGCATTTGTCATATAGTTGTTATAGCTCCAAAGAGTTGCAGTTGAATCATTGTTCGAG
TGTCATAAATTGCTAAATTAATGGAAGCCTCAAGCATTTATACTGTAATAATAAGAGATGATTATATCCGATGCC
ATCAATCTACTTTGGTAGTAGTCTTCTTTTAGGAAGAGATAAAGAAAAGCAATCAGAAAAAACTTCAATCCAGTG
TGCTTTTATCATGATGGATGAAGCCTGCAATTGATACAATCTGTTTAGTTAGTCATCCCATTTTCATATACATTT
AGTTAGCTACTTATGCTTGGAGGAAGCAGATCACTATGGATCATTTTGATCGGTTTATGTTTGTCACTGATTTAT
CCAAGGACAATTATTTGCTGGTGTGGCATGTAAATGTGGATCATAAGAAGCCGCTGTTGTCTTAATTCTTTGGCT

TCAAGGCTGTCATCTGATAATTATTTTCCTATAGCGCACATGCGTTAACCTGATATAATTATAGATTATGTAAAC
ATAATTCATTTAATATGACACTGGTATCTTTACTCTAGTTAGTTGCTTGGTCATTGGTTGTTGGTTTACATTTGA
GGAGAGTAATCATTGCTACTTGTGATAATTAAGATTAATGATTGCATTACTCCATGTAATTCCTTGTAGCTCGAT
CGTGTTTCAAGTCTTGCATCAAAGTACCTTGGAAGGCCCATCACTCAGCTTCCCCCGGTTCAGCCATTATCTGTT
TCATCATTAGACTTATCGGTCGGAGGATACAGTAATCCAGGGATAAGCCCTTCGCTTGATCTTGATCTCCTATGC
CGGAATTCTTCTTCGGCTTTTCCATATACATTTCCTGCAGCAGTTTCTGAGCTTGAAAAACCTCTAATGATGGAG
ATGGCCACTGGTGCAATGGAGGAGGTTATCCGGCTTGTGCAGGCTGATGCACCCTTGTGGGTGAAGTCTGGAAGT
GACGGAAGGGACATACTTCAACTTGAAACCTACGACAGGATTTTCCAAAGGTCAAATCGGCAGCTCAGGTTCCCA
GATACTCAAACTGAGGCATCAAGAGACTCTGCTCTTGTTTTTATGAATGCTACGACATTGATCGATATGTTCATG
GATGCGGTAAAGTTTCATTCTTCTCAAAAAGAAATGCTTTCTCCTTATTCTACTTTTGAATTGATTTGGATTATG
TTGCAGAGCAAGTGGGCGGAGCTGTTTCCTACAATTGTTTCCAAGGCCAGGACCTTTGAAGTCCTTGCAGCTGGA
ATGGCGGGAAGCAGAAGCGGATCTTTGATCCTGGTAATGACAGTTCTTAAGCTCTTTTCATGAATCCAAACTTTC
GAACCTCCTCTGTGATACTGATAAATCCAAAGGCATGCAGATGTATGAGGAGCTACAGGTTCTTTCACCAGTTGT
TCCGACACGCGAATTCTGCTTTCTGCGGTATTGCCAGCAAATAGAGCCACATGTGTGGGCAGTAGCTGATGTTTC
AGTGGACTATCCCAGAGACAATCAGCTTGCTCCTTCCAGATCAAGGAAGCTTCCTTCTGGCTGCTTGATTGAGGA
AATGTCAAATGGCTATTCGAAGGTGATCAAAATATGGTTCTTGTGCTTGCACAATGTTCATAGTTACTGGTTTCT
CTCTGCTTGCATAACAGAATATCCGTGGTGATACTACTGTAGATAACTTGGGTCGAGCACATGGAAATTGAAGAA
AAGAATCCAATTCATATACTCTTCAGGGATCTGATAAATAGTGGAATGGCATTTGGAGCACAGCGCTGGCTCACC
ACCCTCCAGAGAATGTGCGAGAGGTTTGCTTGTTTAACCATCACTGGACTTCCAGCTAGAGACCTTGGAGGTAAA
CTGCAGTTTGCTTAAAAGTTGCAATCATGGGTATCAGAATCTTTATACAACTTGTTAAATTGCTGTTTGATTGTG
TTTTTAACTGTTGACATAGTGACACCAACACCTGATGGCAAGAAGAGCATGATGAAGCTTGCTCAGAGAATGGTT
AACAATTTCTGTGCCAATGTTGGTGCGGCAAATGGTCACAAGTGGACCACCCTCTCTGGGTTAAATGATGTTGGT
GTCAGGGTTACACTTCACAAAAGCACAGATGCTGGCCAGCCTAACGGAGTTGTTCTCAGTGCAGCAACCTCAATA
TGGCTTCCCATATCAACTGAGAGGGTCTTTAGTTTCTTCAAGGATGAACAAACACGAACTCAGGTTTCTGTTCAG
CTTCTCGAGATGACTATTTTAAGATACCTTTCTTTCCGCTGTATTTTTTCTTTTACAAAAAGAGCGTTAACTCTC
CATCTTAATCTCTTTCAGTGGGATGTTCTTGCAAATGGAAACACTGTACAAGAGGTGGCTCATATCACAAATGGA
TCACATCCGGGGAACTGCATCTCTCTTCTTCGTGTATGCTTTCTTTCTCTTGCCCTTGTCCTTGACCTTTAGATT
TATGACATCTTATGGAACGAAAATGTCTTCCATGTGGCTGGTCATGGTTTTTCTTTTTTTTGTAAATATTTATTA
GAATAGTATGCAAATCTAGTGAAAGAGTCTGCCTTCCAGCACCAATAATATTTTCTTTTCATATTTTCCATGAAT
TTGTATGCTTTGTACAATCGAGTTATAATTAATTTTGACCTGCAAGTGAATGCCAATCTGTGTCAGAAGTTTTTA
ATTGTCTATTTTTGTTGGTCATCAAATAGGGATTAAATTCCGGCCAGAACACCATGTTGATACTCCAAGAGTGCT
GCACTGATGCGTCAGGCTCAGTTGTGGTGTATTCTCCCATTGATTTACCGGCCATCAATATTGTCATGAGCGGTG
AGGATCCATCTTACGTACCCATTTTACCTTCAGGCTTCACCATATTACCAGATGGGCGATCTGCTGGGGGACAAG
GCGCATCATCCAGCTCAAATCCATTGGGTGGCTCCTCTGGCTCATTGGTGACTGTCGCATTTCAAATACTTATGA
GCAGCTTGCCATCAGCAAAACTCAATTTGGAGTCAGTAACGACAGTCAATAACTTGATCGGCACCACAGTTCAGC
AAATAAAGGCTGCCTTGAACTGTCCTGACGTCTGATGCATCTGAAGAGAATTAAAAGCCTATTTTTTCATCTCTT
CCCACCACCTCCCTTAACTTCTTCAGATCTGATGAACTCCTCTTAAGGCTTTTGATGCAGCATTCAAGATGGGGA
TTAAAGTGGGTCTTCTATTGTTGGTTGGTTGGCCTTGAATGCTTCCATCATAGGCTTGAATTGGAACTTTAAGAT
GATGCTGCTGCAGCTGCTGCTGGTGCCAGTCTCAGGACAACCCTTTGTTTTCTTACAGGAATCAATCAGGCTTTA
CATTTACAAGTACTTAATGAAGGAAGTCAAGAACGCACCATACCGGCCTCTGCTCTTCTGCTGTCATAACTTCTT
CTTGGCCTTTAGACTTTAGGTTTCTTCTTTTAGGTTTGCTTTAATTCTCTTCTCCTCCACACCTCAATCCCATTC
TTTCCTTTCTTCGTGCAGCAAGCGGTAATGGTTCGGGTATTGACTTCTGTCCTTGAACATTTATTAATGAGCATT
TACTTTGAAG

CDS

ATGGACTCCGGTGACGAACAGGACGTGCCCGACTCACAGGGGAGGAAGAAGCGTTACCACCGGCACACGCCGCGGCAG
ATTCAGGAGCTCGAATCGATGTTCAAGGTTTGCCCGCACCCTGACGAGAAGCAGAGGGCGCAGCTGAGCCGGGATCTGG
GGCTGGAGCCGCGGCAGATCAAGTTCTGGTTCCAGAACCGCAGGACGCAGATGAAGGCAACCGCTCGCCGCTCCCTACT
GGCGGACAACTGCTTGCTCCGTGCTGAGAACGACAAGATCCGGTGCGAGAACATCGCGATGAGGGAGGCTATGAAGAA
CGTCATCTGCCCCTTCCTGCGGCGCCCCTCCGGCCAGTGACGACTCCTACTTTGACGAGCAGAAGCTGCGGATGGAGAATG
CGAGGTTGAAGGAGGAGCTCGATCGTGTTTCAAGTCTTGCATCAAAGTACCTTGGAAGGCCCATCACTCAGCTTCCCCCG
GTTCAGCCATTATCTGTTTCATCATTAGACTTATCGGTCGGAGGATACAGTAATCCAGGGATAAGCCCTTCGCTTGATCTTG
ATCTCCTATGCCGGAATTCTTCTTCGGCTTTTCCATATACATTTCCTGCAGCAGTTTCTGAGCTTGAAAAACCTCTAATGATG

GAGATGGCCACTGGTGCAATGGAGGAGGTTATCCGGCTTGTGCAGGCTGATGCACCCTTGTGGGTGAAGTCTGGAAGTG
ACGGAAGGGACATACTTCAACTTGAAACCTACGACAGGATTTTCCAAAGGTCAAATCGGCAGCTCAGGTTCCCAGATACT
CAAACTGAGGCATCAAGAGACTCTGCTCTTGTTTTTATGAATGCTACGACATTGATCGATATGTTCATGGATGCGAGCAAG
TGGGCGGAGCTGTTTCCTACAATTGTTTCCAAGGCCAGGACCTTTGAAGTCCTTGCAGCTGGAATGGCGGGAAGCAGAA
GCGGATCTTTGATCCTGATGTATGAGGAGCTACAGGTTCTTTCACCAGTTGTTCCGACACGCGAATTCTGCTTTCTGCGGT
ATTGCCAGCAAATAGAGCCACATGTGTGGGCAGTAGCTGATGTTTCAGTGGACTATCCCAGAGACAATCAGCTTGCTCCT
TCCAGATCAAGGAAGCTTCCTTCTGGCTGCTTGATTGAGGAAATGTCAAATGGCTATTCGAAGATAACTTGGGTCGAGCA
CATGGAAATTGAAGAAAAGAATCCAATTCATATACTCTTCAGGGATCTGATAAATAGTGGAATGGCATTTGGAGCACAGC
GCTGGCTCACCACCCTCCAGAGAATGTGCGAGAGGTTTGCTTGTTTAACCATCACTGGACTTCCAGCTAGAGACCTTGGA
GTGACACCAACACCTGATGGCAAGAAGAGCATGATGAAGCTTGCTCAGAGAATGGTTAACAATTTCTGTGCCAATGTTGG
TGCGGCAAATGGTCACAAGTGGACCACCCTCTCTGGGTTAAATGATGTTGGTGTCAGGGTTACACTTCACAAAAGCACAG
ATGCTGGCCAGCCTAACGGAGTTGTTCTCAGTGCAGCAACCTCAATATGGCTTCCCATATCAACTGAGAGGGTCTTTAGTT
TCTTCAAGGATGAACAAACACGAACTCAGTGGGATGTTCTTGCAAATGGAAACACTGTACAAGAGGTGGCTCATATCACA
AATGGATCACATCCGGGGAACTGCATCTCTCTTCTTCGTGGATTAAATTCCGGCCAGAACACCATGTTGATACTCCAAGAG
TGCTGCACTGATGCGTCAGGCTCAGTTGTGGTGTATTCTCCCATTGATTTACCGGCCATCAATATTGTCATGAGCGGTGAG
GATCCATCTTACGTACCCATTTTACCTTCAGGCTTCACCATATTACCAGATGGGCGATCTGCTGGGGGACAAGGCGCATCA
TCCAGCTCAAATCCATTGGGTGGCTCCTCTGGCTCATTGGTGACTGTCGCATTTCAAATACTTATGAGCAGCTTGCCATCAG
CAAAACTCAATTTGGAGTCAGTAACGACAGTCAATAACTTGATCGGCACCACAGTTCAGCAAATAAAGGCTGCCTTGAAC
TGTCCTGACGTCTGA
